# Palladium‐Catalyzed *Gem*‐Diborylalkylation of Silyl Enol Ethers and *N*‐Vinylacetamide via Diboryl Carbon‐Centered Radicals

**DOI:** 10.1002/advs.202508566

**Published:** 2025-07-16

**Authors:** Xiao‐Yu Xie, Yi Wei, Xin‐Yi Chen, Ming Li, Kai Hong

**Affiliations:** ^1^ Shanghai Engineering Research Center of Molecular Therapeutics and New Drug Development School of Chemistry and Molecular Engineering East China Normal University 3663 N Zhongshan Road Shanghai 200062 China; ^2^ Shanghai Frontiers Science Center of Molecule Intelligent Syntheses School of Chemistry and Molecular Engineering East China Normal University 3663 N Zhongshan Road Shanghai 200062 China

**Keywords:** iododiboron, organoboron, palladium catalysis, silyl enol ether, synthetic methods

## Abstract

Herein, a palladium‐catalyzed coupling of *gem*‐iododiborylalkanes with electron‐rich olefins to access β,β‐diboryl ketones and aldehydes under mild conditions is reported. This method exhibits broad substrate scope and excellent functional group tolerance. Mechanistic studies support a distinctive pathway involving *gem*‐diboryl carbon‐centered radicals and a Pd(0)/Pd(I) catalytic cycle, without the formation of Pd(II) intermediates. This protocol provides a new platform for accessing synthetically valuable *gem*‐diboryl carbonyl compounds and expands the reactivity profile of organoboron‐based radical transformations.

## Graphical abstract

Unlocking new reactivity: a palladium‐catalyzed coupling of *gem*‐iododiborylalkanes with electron‐rich olefins enables streamlined access to β,β‐diboryl carbonyl compounds under mild conditions. Mechanistic insights reveal a Pd(0)/Pd(I) cycle and a diboryl‐stabilized radical intermediate, offering a distinct platform for advancing unconventional organoboron and radical chemistry.

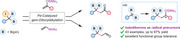

## 1 Introduction

The strategic incorporation of a boryl group to modulate the reactivity of α‐radicals has emerged as a powerful tool in developing new transformations over the past decade.^[^ ^1^, ^2^, ^3^ ^]^ In the case of neutral, three‐coordinate boronates, the vacant *p* orbital on the boron atom allows for resonance stabilization of the adjacent carbon‐centered radical.^[^ ^2^ ^]^ Recent advances have featured the use of geminal diboryl substitution at the α‐position, enabling dual stabilization and unlocking new reactivity paradigms. Notably, the groups of Masarwa,^[^ ^4^ ^]^ Renaud,^[^ ^5^ ^]^ and Sharma^[^ ^6^ ^]^ have developed a range of transformations involving *gem*‐diboryl radicals, which originated from 1,1‐diborylalkenes or *gem*‐diboryl cyclopropanes.

**Scheme 1:**
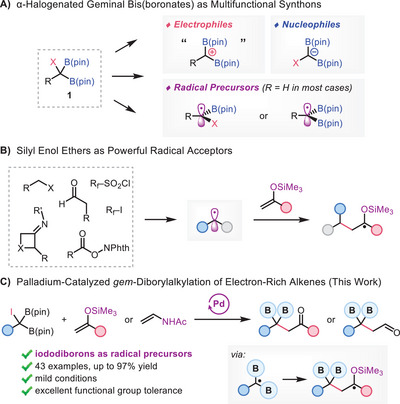
Radical‐Based Transformations of *gem*‐Halodiborylalkanes and Silyl Enol Ethers.

In recent years, several groups, including our own, have demonstrated that geminal halodiborylalkanes (**1**),^[^ ^7^ ^]^ a fusion of α‐haloboronates^[^ ^8^ ^]^ and geminal bis(boronates),^[^ ^9^ ^]^ can serve as multifunctional synthons, participating in reactions not only as electrophiles^[^ ^10^, ^11^, ^12^ ^]^ and nucleophiles,^[^ ^7^, ^13^, ^14^, ^15^ ^]^ but also as precursors to diboryl‐stabilized radicals^[^ ^16^, ^17^, ^18^, ^19^, ^20^ ^]^ (**Scheme** **1**A). In 2024, our group reported the first palladium‐catalyzed three component reaction of *gem*‐iododiboron compounds, styrenes, and aldehydes or imines, featuring the intermediacy of a Pd(I) species and *gem*‐diboryl radical intermediates.^[^ ^17^ ^]^ Concurrently, Molloy and co‐workers, building on their earlier research on monoboryl‐stabilized radicals,^[^ ^2k,l^ ^]^ developed an elegant, light‐driven system that uses only a Lewis base to directly activate the C─I bond of iododiborylmethane.^[^ ^18^ ^]^ More recently, Liu and co‐workers employed iododiborylmethane in a *N*‐heterocyclic carbene‐boryl radical‐mediated reaction to construct 1,1‐bis(boronates) from alkenes.^[^ ^19^ ^]^ Hu and Jing reported carboiodination of alkynes to forge γ‑iodo‐allylic *gem*‐bis(boronates) in the presence of visible light and a manganese catalyst.^[^ ^20^ ^]^

Despite these advances, the scope of the radical transformations described above are mainly confined to unsubstituted iododiborylmethane. While our method accommodates a variety of substituted iododiborylalkanes, the scope of the radical acceptor is currently restricted to mono‐substituted styrenes. Considering the resonance stabilization between the α‐radical and boron atoms enhances the electrophilicity of the radical center,^[^ ^21^, ^22^ ^]^ we reasoned that electron‐rich olefins, such as enol ethers, might engage more readily with *gem*‐diboryl radicals. Indeed, silyl enol ethers are not only powerful building blocks in classic transformations^[^ ^23^ ^]^ such as Mukaiyama aldol reaction^[^ ^24^ ^]^ and Danishefsky's diene cycloaddition,^[^ ^25^ ^]^ but have also found widespread application as radical acceptors,^[^ ^26^ ^]^ which has been extensively demonstrated by MacMillan,^[^ ^27^ ^]^ Melchiorre,^[^ ^28^ ^]^ Fu,^[^ ^29^ ^]^ and many other groups (Scheme 1B).^[^ ^30^ ^]^

In this article, we report a palladium‐catalyzed coupling of *gem*‐iododiborylalkanes with electron‐rich silyl enol ethers to afford β,β‐diboryl ketones in a highly efficient manner (Scheme 1C).^[^ ^4^, ^31^ ^]^ A complementary variant employing *N*‐vinylacetamide enables access to the corresponding β,β‐diboryl aldehydes. These reactions showed superb compatibility with various functional groups and heterocycles, significantly broadening the scope of *gem*‐diboryl radical chemistry and offering a new synthetic approach to access polyfunctionalized *gem*‐diborylalkanes, which are synthetically valuable motifs in organic synthesis.^[^ ^9^ ^]^ Importantly, mechanistic experiments provide evidence that supports a catalytic cycle involving Pd(I)/diboryl radical intermediates, notably proceeding without the formation of Pd(II) species. This mechanistic divergence from our previous work,^[^ ^17^ ^]^ in which β‐hydride elimination from a Pd(II) intermediate was rate‐determining, allows the present transformation to operate under milder conditions and expands the reactivity beyond Heck‐type reaction, therefore offering new opportunities for the design of radical‐mediated transformations in organoboron chemistry.

## 2 Results and Discussion

We began by establishing a set of standard conditions based on our previous study. A thorough investigation of critical reaction parameters was conducted, including the precatalyst, ligand, base, solvent, and other relevant factors (see Tables S1–S4, Supporting Information). Using a more electron‐rich silyl enol ether substrate (**3a**), we anticipated enhanced reactivity in this transformation compared to analogous reactions using styrenes. Indeed, at room temperature, we observed an 97% NMR yield of β,β‐diboryl ketone (**4a**) with 10 mol% Pd(OAc)_2_ and 20 mol% Xantphos in DCM after 8 h (**Table** **1**, entry 1), whereas our previously reported reaction with styrenes required elevated temperatures (80 °C). Remarkably, this reaction even proceeded at 0 °C, albeit at a much lower rate (entry 3). We were also delighted to find that several phosphine ligands could promote the reaction, among which Xantphos was the most effective (entries 4–7). The addition of water was found to be essential for turnover (entry 8). Conducting the reaction in the dark had a negligible impact on the yield, suggesting that a photochemical process was likely not involved (entry 9). Although a base was indispensable (entry 10), the reaction showcased a surprising tolerance to a range of organic and inorganic bases. Triethylamine, along with secondary and primary alkylamines, afforded comparable or slightly reduced yields (entry 11). Even inorganic bases, previously not very compatible with halodiborylalkanes, furnished the desired product in moderate to high yields (entry 12). The improved tolerance was partially attributed to the lower reaction temperature, and also suggested that the base may function merely as a proton scavenger in this transformation. Next, the variation on the halodiboron substrate revealed that the bromo‐analog remained reactive, providing **4a** in 85% yield (entry 13), whereas the corresponding chlorodiboron compound exhibited substantially reduced reactivity (entry 14). Lastly, we evaluated the effect of different silyl groups. While replacing trimethylsilyl (TMS) group with *tert*‐butyldimethylsilyl (TBS) group led to a slightly reduced yield, the reaction efficiency was significantly suppressed when a sterically bulkier triisopropylsilyl (TIPS) group was employed, affording the desired product in only 15% yield (entry 15). These observations suggest that steric hindrance around the silyl enol ether may impede key steps in the transformation.

**Table 1:** Optimization of Reaction Conditions.

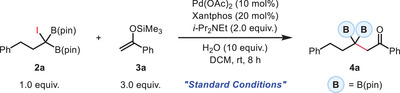		
Entry^a)^	variation from the standard conditions	NMR yield of 4a (%)
1	none	97 (97)^b)^
2	reaction for only 1 h	67
3	reaction at 0 °C	41
4	*N*‐Xantphos as ligand	44
5	PPh_3_ as ligand	53
6	other ligands (see Table S2, Supporting Information)	<10
7	no ligand	0
8	no water additive	9
9	reaction conducted in the dark	97
10	no base	0
11	Et_3_N/*i*‐Pr_2_NH/*i*‐PrNH_2_, instead of *i*‐Pr_2_NEt	96/96/83
12	CsF/Na_2_CO_3_/K_3_PO_4_, instead of *i*‐Pr_2_NEt	74/70/95
13	bromodiboron, instead of **2a**	85
14	chlorodiboron, instead of **2a**	10
15	TBS/TIPS, instead of TMS	87/15

^a)^ The standard conditions: iododiboron **2a** (0.1 mmol), silyl enol ether **3a** (0.3 mmol), Pd(OAc)_2_ (0.01 mmol), Xantphos (0.02 mmol), *i*‐Pr_2_NEt (0.2 mmol), H_2_O (1.0 mmol), DCM (1 mL), rt, 8 h. The crude yield was determined by ^1^H NMR using 1,3,5‐trimethoxybenzene as the internal standard;

^b)^ isolated yield in the parentheses.

With the optimal conditions in hand, we first explored the scope of silyl enol ethers, as shown in **Table** **2**A. To facilitate purification, the *para*‐methoxy‐substituted analogue of iododiboron **2a** was adopted. A broad range of silyl enol ethers bearing both electron‐donating and electron‐withdrawing substituents on the aryl ring, including halogens, methoxy, ester, and nitro groups, proved compatible, regardless of their position (*ortho*‐, *meta*‐, or *para*‐), furnishing the desired β,β‐diboryl ketones in excellent yields (**4b**–**4i**). Heteroaromatic motifs, such as furan, thiophene, and pyridine rings, were also well tolerated, delivering the corresponding products with high efficiency (**4j**–**4l**). While the alkynyl‐substituted substrate showed slightly diminished reactivity (**4m**), the presence of an alkenyl group did not compromise the reaction efficiency (**4n**). Notably, Danishefsky's diene was found to be a competent radical acceptor, albeit in a moderate 42% yield (**4o**). Lastly, alkyl‐substituents were evaluated. The acetone‐derived silyl enol ether successfully underwent the reaction to give **4p** in 58% yield under re‐optimized conditions. However, further extension to other alkyl variants proved challenging, likely due to a combination of substrate impurity and the inherent instability of the resulting alkyl radicals, and no desired product could be isolated under the standard conditions. Trisubstituted silyl enol ethers were also evaluated but found to be unreactive, likely due to steric hindrance.

**Table 2:** Substrate Scope of *gem*‐Diborylalkylation of Silyl Enol Ethers (see Table footnotes).

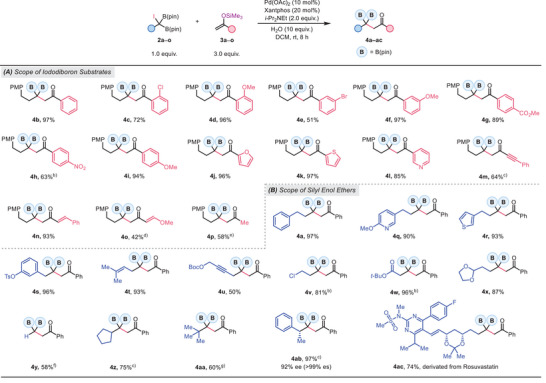

^a)^ Standard conditions: geminal iododiborylalkane (0.1 mmol), silyl enol ether (0.3 mmol), Pd(OAc)_2_ (0.01 mmol), Xantphos (0.02 mmol), *i*‐Pr_2_NEt (0.2 mmol), DCM (1 mL), rt, 8 h under an argon atmosphere. Isolated yields;

^b)^ 40 °C, 14 h;

^c)^ 40 °C;

^d)^ 1,4‐dioxane, 50 °C, 24 h;

^e)^ 6.0 equiv. of silyl enol ether, 1,4‐dioxane, 40 °C, 24 h;

^f)^ 0.2 mmol iododiborylmethane, 0.1 mmol silyl enol ether **3a**, 2 equiv. H_2_O, 50 °C, 14 h;

^g)^ Geminal bromodiborylalkane, 1,4‐dioxane, 50 °C, 24 h.

Next, the generality of *gem*‐iododiborylalkanes was evaluated (Table 2B). Heterocycles proceeded smoothly to deliver the desired products in high yields (**4q** and **4r**). Benzyl, allyl, and propargyl substituents (**4s**–**4u**), along with other synthetically useful functionalities (**4v**–**4x**), were also compatible. Iododiborylmethane, which was briefly demonstrated in Molloy's photocatalyzed system,^[^ ^18^ ^]^ was less reactive in our reaction. Since it's readily available,^[^ ^7^, ^16^ ^]^ by increasing its loading to 2.0 equivalents, we successfully improved the isolated yield of **4y** to 58%. Steric hindrance on halodiboron compounds was not a significant limitation for this transformation. A secondary alkyl‐substituted iododiboron compound only required a slightly elevated temperature to afford a good yield (**4z**). Even the sterically demanding *tert*‐butyl group was well accommodated (**4aa**). The bromodiboron analogue was used in this case due to constraints in substrate preparation.^[^ ^32^ ^]^ Furthermore, a substrate bearing an adjacent chiral center was smoothly converted to the desired product **4ab** in excellent yield. Importantly, erosion of enantiomeric excess was not detected (> 99% es). Finally, this method was successfully applied to a polyfunctionalized, bioactive fragment (**4ac**), showcasing its potential for late‐stage modification in medicinal chemistry.

Encouraged by the success of β,β‐diboryl ketones, we sought to expand this methodology to the preparation of the analogous aldehydes. Despite extensive optimization, our initial attempts using silyl vinyl ether as the radical acceptor only afforded moderate yields, with an NMR yield of up to 55% (see Tables S5 and S6, Supporting Information). To overcome this challenge, we explored alternative electron‐rich olefins, including vinyl acetate and *N*‐vinylacetamide. After further screening (see Tables S7–S10, Supporting Information), we identified a set of optimized conditions employing *N*‐vinylacetamide as the radical acceptor, Pd(dppf)Cl₂ as the precatalyst, and 1,4‐dioxane as the solvent at 80 °C, which delivered the desired product **5a** in 89% NMR yield and 73% isolated yield. Intriguingly, dppf and Xantphos were individually effective ligands for the reaction, and a slightly improved yield was observed when using Pd(dppf)Cl_2_ in combination with additional Xantphos (see Table S10, Supporting Information for details).

The scope of this transformation is illustrated in **Table** **3**. While the NMR yields were generally high for various substrates, these aldehydes exhibited lower stability on silica gel, suffering yield loss during purification. Nevertheless, the reaction tolerated a wide array of iododiborylalkane substrates, including those bearing heterocycles, secondary and tertiary alkyl side chains, and synthetically valuable functional groups. Moreover, the protocol was applicable to structurally complex iododiborons derived from bioactive molecules such as rosuvastatin (**5m**) and glucose (**5n**) with synthetically useful yields.

**Table 3:** Substrate Scope of *gem*‐Diborylalkylation of *N*‐Vinylacetamide (see Table footnotes).

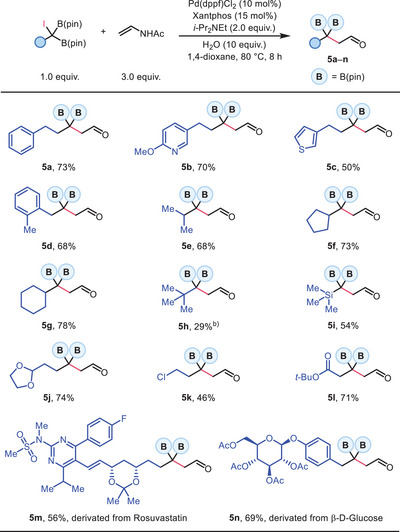

^a)^ Standard conditions: geminal iododiborylalkane (0.1 mmol), *N*‐vinylacetamide (0.3 mmol), Pd(dppf)Cl_2_ (0.01 mmol), Xantphos (0.015 mmol), *i*‐Pr_2_NEt (0.2 mmol), 1,4‐dioxane (1 mL), 80 °C, 8 h under an argon atmosphere. Isolated yields;

^b)^ Geminal bromodiborylalkane, 100 °C.

**Scheme 2:**
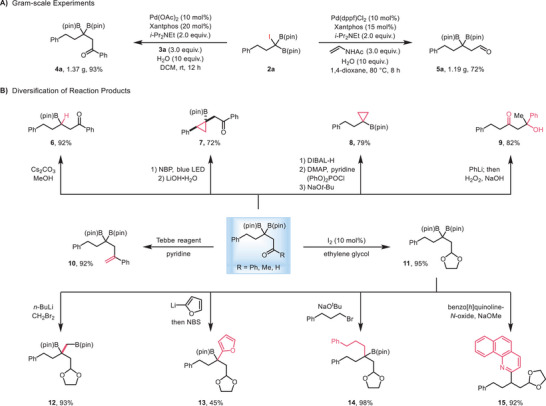
Gram‐scale Reaction and Synthetic Application of Products.

To demonstrate the practicality and robustness of this method, gram‐scale reactions were performed for both β,β‐diboryl ketone **4a** and aldehyde **5a** with comparable efficiency to the small‐scale reactions (**Scheme** **2**A). The synthetic potential of our products was showcased through a variety of diversifications (Scheme 2B). Notably, by leveraging our previously developed deborylative cyclization strategy,^[^ ^33^ ^]^ cyclopropane derivatives **7** and **8** were efficiently forged. Nucleophilic addition followed by H_2_O_2_ oxidation afforded β‐hydroxy ketone **9** in 82% yield. Exposure of **4a** to Tebbe reagent^[^ ^34^ ^]^ resulted in smooth olefination to deliver **10** in 92% yield. Furthermore, under acid‐free conditions,^[^ ^35^ ^]^ aldehyde **5a** was protected to give acetal **11**, which enabled selective downstream transformations of the diboron motif. Specifically, one of the B(pin) groups in **11** underwent transition metal‐free derivatization, including Matteson homologation,^[^ ^36^ ^]^ Aggarwal's arylation,^[^ ^37^ ^]^ and Morken's deborylative alkylation,^[^ ^38^ ^]^ to furnish products **12**, **13**, and **14**, respectively. Lastly, Cho's protocol^[^ ^39^ ^]^ was successfully applied to convert the diboryl unit into a heteroaryl group.

**Scheme 3:**
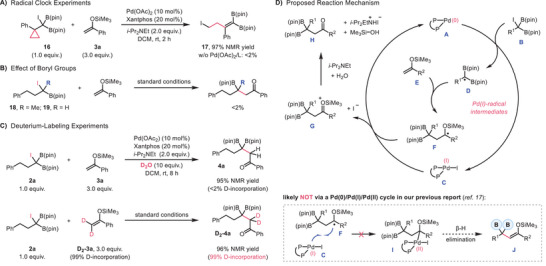
Brief Survey and Proposal of Reaction Mechanism.

Next, our efforts were devoted to elucidating the reaction mechanism. The exclusive formation of ring‐opening product **17** in the radical clock experiment strongly supports the involvement of a *gem*‐diboryl carbon‐centered radical, consistent with our previous study^[^ ^17^ ^]^ (**Scheme** **3**A). Dual boron stabilization proved essential for reactivity. When one of the B(pin) groups was substituted with either a hydrogen or methyl group, the reaction was completely prohibited (Scheme 3B). We also conducted deuterium‐labeling experiments (Scheme 3C). Performing the reaction in the presence of D_2_O led to product **4a** without any deuterium incorporation. Conversely, the reaction using D_2_‐**3a** furnished product D_2_‐**4a** without any loss of deuterium labeling. These outcomes excluded the involvement of an enolate intermediate during the catalytic cycle.

Based on these results and related proposals in the literature, a plausible mechanism was depicted in Scheme 3D. The catalytic cycle begins with iodine atom abstraction from substrate **B** by an active Pd(0) catalyst **A**, generating a Pd(I) species **C** and a diboryl‐stabilized radical **D**. This activation mode is commonly associated with elevated temperature or photoexcited Pd(0)^*^ catalysts under visible light irradiation.^[^ ^40^ ^]^ However, in our case, heating or photoinduction is not essential, likely due to the exceptional stability of radical **D** with dual boron stabilization. Radical **D** then adds to silyl enol ether **E**, forming a new radical intermediate **F**. This intermediate likely undergoes iodine atom abstraction from Pd(I) species **C**, leading to the regeneration of Pd(0) catalyst **A** and formation of intermediate **G**. Similar iodine‐abstraction from Pd(I) has been widely proposed in alkene carbohalogenation reactions via a Pd(0)/Pd(I) catalytic cycle.^[^ ^41^ ^]^ Lastly, desilylation of intermediate **G** in the presence of base and H_2_O provides the final product **H**.

It is worth mentioning that, unlike our previous study,^[^ ^17^ ^]^ no evidence supports the formation of a Pd(II) intermediate (**I**) via recombination of radical **F** and Pd(I). This alternative pathway would lead to β‐hydride elimination and formation of enolate **J**, a formal Heck coupling product,^[^ ^29^, ^42^ ^]^ which has been ruled out by our deuterium‐labeling experiments.

## 3 Conclusion

In conclusion, we have developed a palladium‐catalyzed coupling reaction of *gem*‐iododiboron compounds with silyl enol ethers or *N*‐vinylacetamide. High efficiency, broad functional group tolerance, and diverse utilization were demonstrated. Mechanistic investigation supported the intermediacy of a *gem*‐diboryl carbon‐centered radical and a distinctive Pd(0)/Pd(I) catalytic cycle without photoinduction.

## Conflict of Interest

The authors declare no conflict of interest.

## Supporting information



Supporting Information

## Data Availability

The data that support the findings of this study are available in the supplementary material of this article.
