# Supplementary material for: Palladium‐Catalyzed Gem‐Diborylalkylation of Silyl Enol Ethers and N‐Vinylacetamide via Diboryl Carbon‐Centered Radicals
Source: Adv Sci (Weinh). 2025 Jul 16;12(38):e08566. doi: 10.1002/advs.202508566 (PMC12520485; doi:10.1002/advs.202508566)
Supplement: Supplementary file 1 — Supporting Information [file ADVS-12-e08566-s001.pdf]

ADVANCED SCIENCE
Open Access

Supporting Information

for *Adv. Sci.*, DOI 10.1002/advs.202508566

Palladium-Catalyzed *Gem*-Diborylalkylation of Silyl Enol Ethers and *N*-Vinylacetamide via Diboryl Carbon-Centered Radicals

*Xiao-Yu Xie, Yi Wei, Xin-Yi Chen, Ming Li and Kai Hong**

Palladium-Catalyzed *gem*-Diborylalkylation of Silyl Enol Ethers and *N*-Vinylacetamide via Diboryl Carbon-Centered Radicals

Xiao-Yu Xie,[a] Yi Wei,[a] Xin-Yi Chen,[a] Ming Li,[a] and Kai Hong*[a,b]

[a] Shanghai Engineering Research Center of Molecular Therapeutics and New Drug Development, School of Chemistry and Molecular Engineering, East China Normal University, 3663 N Zhongshan Road, Shanghai 200062, China.

[b] Shanghai Frontiers Science Center of Molecule Intelligent Syntheses, School of Chemistry and Molecular Engineering, East China Normal University, 3663 N Zhongshan Road, Shanghai 200062, China

Supporting Information

Table of Contents

General Information 2
Experimental Section 3
I. Preparation of Substrates 3
Scope of Silyl Enol Ethers 3
Scope of Iododiboron Substrates 5
II. Palladium-Catalyzed *gem*-Diborylalkylation of Silyl Enol Ethers 13
Optimization of Reaction Conditions 13
Experimental Procedures and Spectral Data 17
III. Palladium-Catalyzed *gem*-Diborylalkylation of *N*-Vinylacetamide 31
Optimization of Reaction Conditions 31
Experimental Procedures and Spectral Data 35
IV. Synthetic Utilization 41
Gram-scale Experiments 41
Diversification of Products 41
V. Mechanistic Studies 49
Radical-clock Experiment 49
Effect of Boryl Groups 50
Isotope-Labeling Experiments 50
References 53
NMR Spectra 54

General Information

Unless otherwise noted, all commercial materials were purchased from Alfa-Aesar, Laajoo (SINOCOMPOUND), Leyan, Macklin, Adamas, Energy Chemical, and Aladdin, and used without further purification. THF, dichloromethane, and toluene were purified using a solvent purification system from Vigor Technologies Co., Ltd. Anhydrous chlorobenzene, 1,4-dioxane, DCE, DME, and MeCN were purchased from Adamas or Energy Chemical. Anhydrous ethyl acetate was prepared by distillation over anhydrous $MgSO_4$. *N*-vinylacetamide was purchased from Macklin. All air- and water-sensitive reactions were carried out in oven-dried glassware under an argon atmosphere by using standard Schlenk manifold techniques or in a glovebox. The photoinduced bromination experiments were carried out in a Parallel Light Reactor (WP-TEC-1020HSL, WATTCAS, China).

Flash column chromatographic purification of products was accomplished using forced-flow chromatography on silica gel (300-400 mesh). The *pre-dried silica gel* was prepared by baking in the oven at 105 °C for 24 hours prior to use. All mixed solvent eluents are reported as v/v solutions.

^{1}H NMR spectra were recorded on Bruker DPX-500 instrument (500 MHz) or Bruker DRX-600 instrument (600 MHz). Chemical shifts were quoted in parts per million (ppm) referenced to 0.0 ppm for tetramethylsilane in $CDCl_3$. The following abbreviations (or combinations thereof) were used to explain multiplicities: s = singlet, d = doublet, t = triplet, q = quartet, m = multiplet, br = broad. Coupling constants, *J*, were reported in Hertz unit (Hz). ^{13}C NMR spectra were recorded on Bruker DPX-500 instrument (125 MHz) or Bruker DRX-600 instrument (150 MHz), and were fully decoupled by broadband proton decoupling. Chemical shifts were reported in ppm referenced to the center line of a triplet at 77.00 ppm of $CDCl_3$. The resonances of the carbon next to the boron atom were not observed or broadened due to quadrupolar relaxation. High resolution mass spectra (HRMS) were recorded on the Bruker maXis impact, and Waters G2-XS qtof equipped with ESI ionization source. Analytical chiral supercritical fluid chromatography (SFC) was performed on the Waters ACQUITY UPC^2. Chiral SFC was performed using Diacel Chiralpak IG-3 (4.6 × 250 mm × 3 μm) and Chiralcel OD-3 columns (4.6 × 250 mm × 3 μm), and monitored by DAD (diode array detector).

Experimental Section

I. Preparation of Substrates

Scope of Silyl Enol Ethers

The following silyl enol ethers **3n-3o** were purchased from commercial sources:

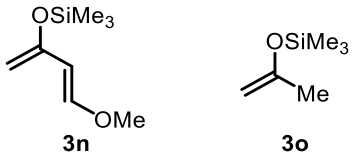

The following silyl enol ethers **3a-3m**[1] have been previously synthesized and characterized:

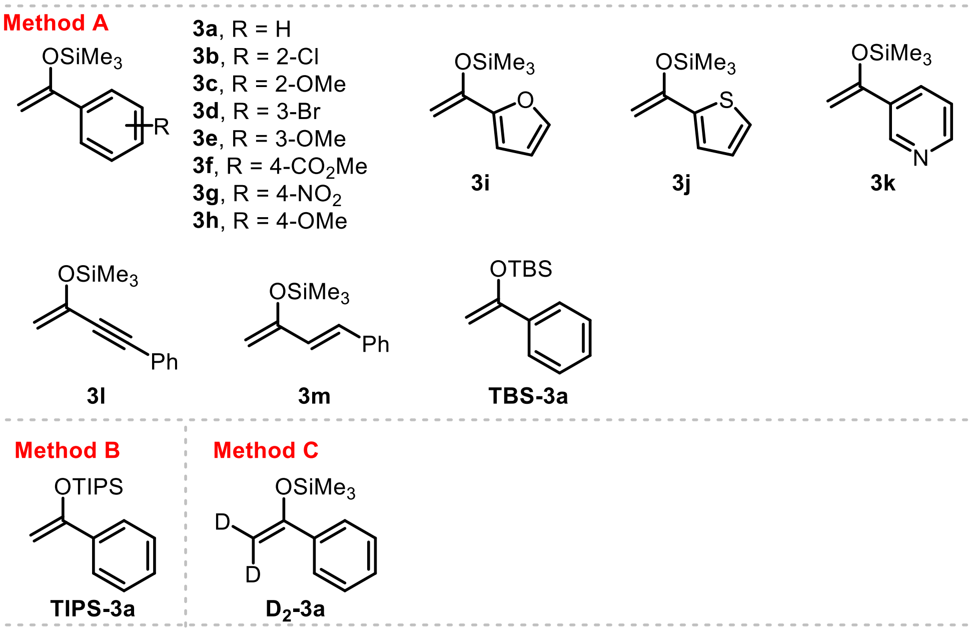

Method A:

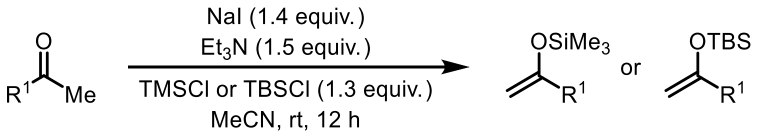

The reaction was performed according to the procedure in the literature with slight modifications.[1] An oven-dried 40 mL vial with a magnetic stir bar was charged with NaI (944 mg, 6.3 mmol, 1.4 equiv.). The vial was sealed with a polypropylene open-top cap with PTFE/silicone septum, evacuated and backfilled with argon three times, then anhydrous MeCN (10 mL), ketone (4.5 mmol, 1.0 equiv.), Et_3N (0.94 mL, 6.8 mmol, 1.5 equiv.) and TMSCl or TBSCl (5.9 mmol, 1.3 equiv.) were successively added. The mixture was vigorously stirred overnight at room temperature while monitored by TLC. After the reaction was completed, ice-water (20 mL) and cold pentane (20 mL) were successively added. After decantation, the aqueous layer was extracted with pentane (2 × 20 mL). The combined organic layers were dried over $MgSO_4$, filtered, and concentrated *in vacuo*. After determining the purity by NMR analysis,

the obtained silyl enol ether was used directly in the next step without further purification.

Silyl enol ether **TIPS-3a**[1] has been previously synthesized and characterized:

Method B:

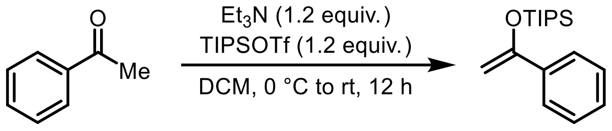

TIPS-3a

The reaction was performed according to the procedure in the literature with slight modifications.[1] Acetophenone (0.58 mL, 5.0 mmol, 1.0 equiv.) was added to a flame-dried round-bottom flask at 0 °C under argon. Anhydrous dichloromethane (20 mL) was added to the flask, followed by Et_3N (0.83 mL, 6.0 mmol, 1.2 equiv.). Then TIPSOTf (1.6 mL, 6.0 mmol, 1.2 equiv.) was added dropwise via syringe. The mixture was vigorously stirred overnight at room temperature while monitored by TLC. Upon completion, the reaction was quenched with sat. aq. $NaHCO_3$ and diluted with cold DCM. The phases were separated and the aqueous layer was extracted with DCM (3 × 20 mL). The combined organic layers were dried over $MgSO_4$, filtered, and concentrated *in vacuo*. After determining the purity by NMR analysis, the obtained silyl enol ether was used directly in the next step without further purification.

Silyl enol ether **D_2-3a**[1] has been previously synthesized and characterized:

Method C:

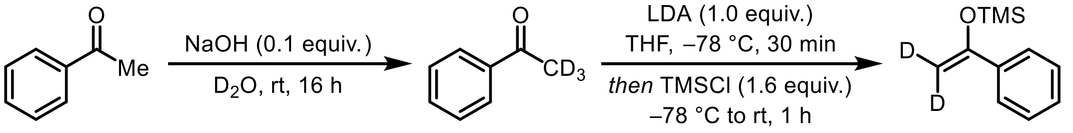

D_2-3a

STEP I: [D_3]Acetophenone was prepared according to the procedure in the literature with slight modifications.[1] A solution of acetophenone (0.47 mL, 4 mmol), NaOH (16 mg, 0.4 mmol) and D_2O [99% D] (2.4 mL, 130 mmol) was stirred at room temperature for 16 h under argon. The reaction mixture was diluted with anhydrous Et_2O (2 mL) and separated. The organic layer was dried over $MgSO_4$, filtered, concentrated *in vacuo*. After determining the purity by NMR analysis, the obtained silyl enol ether was used directly in the next step without further purification. [D_3]Acetophenone [99% D-incorporation] was obtained in 91% yield.

STEP II: To a solution of 2.0 M LDA (1.6 mL, 3.2 mmol) in anhydrous THF (3 mL) prepared at –78 ℃, [D_3]acetophenone (394 mg, 3.2 mmol) was added dropwise and stirred at –78 ℃ for 30 minutes. TMSCl (0.66 mL, 5.1 mmol) was added slowly at –78 ℃, and the reaction mixture was allowed to warm to room temperature and stirring was continued for 1 hour. The reaction mixture was diluted with anhydrous hexane (4 mL) and filtration of white solid (LiCl). The organic layer was dried ($MgSO_4$), filtered, concentrated *in vacuo*. After determining the purity by NMR analysis, the obtained silyl enol ether was used directly in the next step without further purification. [D_2]-α-trimethylsiloxystyrene (99% D-incorporation) was obtained in 87% yield.

Scope of Iododiboron Substrates

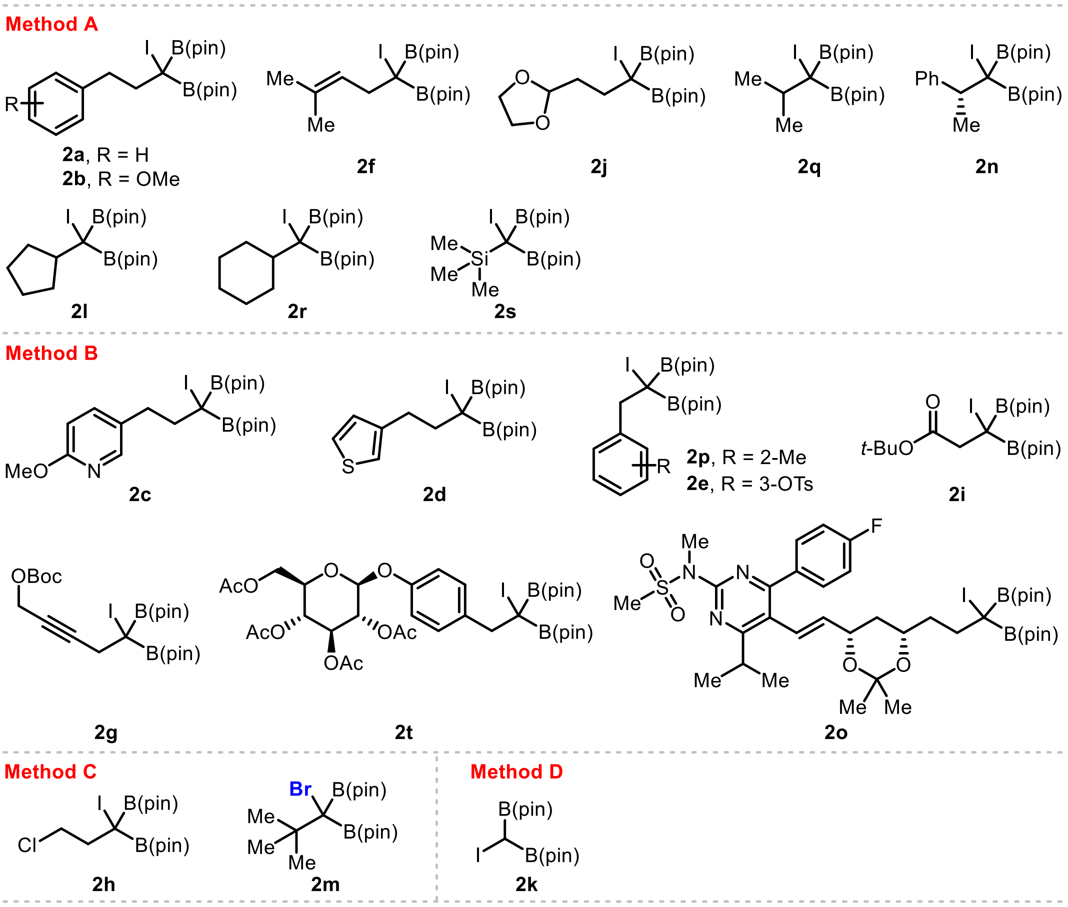

Method A

Iododiboron substrates **2a-2b, 2q, 2l** and **2s** were prepared according to **Method A** and have been previously synthesized and characterized.[2]

Procedure:

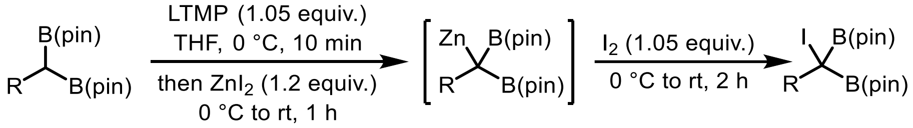

Preparation of Lithium 2,2,6,6-tetramethylpiperidide (LTMP): A 50 mL over-dried round-bottom flask with a magnetic stir bar was charged with anhydrous 2,2,6,6-tetramethylpiperidine (311 mg, 2.2 mmol, 1.1 equiv.). The vessel was evacuated and refilled with argon for three cycles, followed by the addition of THF (4 mL). After cooling the reaction vessel to 0 °C, *n*-BuLi (2.5 M in *n*-hexane, 0.84 mL, 2.1 mmol) was added via syringe slowly and carefully, resulting in a light-yellow solution, and the reaction was allowed to stir for 30 minutes at 0 °C. Upon completion, the LTMP solution was used immediately.

Preparation of Iododiboron Substrates: A 50 mL over-dried round-bottom flask with a magnetic stir bar was charged with 1,1-diborylalkanes (2 mmol, 1.0 equiv.). The flask was evacuated and refilled with argon three times, followed by the addition of THF (6 mL). To the solution was added the freshly prepared LTMP solution dropwise at 0 °C. After stirring for 10

minutes, ZnI_2 solution (766 mg, 2.4 mmol, in 2 mL THF) was added dropwise. The reaction mixture was allowed to warm to room temperature and stirred for 1 hour, then cooled back to 0 °C again, and I_2 solution (533 mg, 2.1 mmol, in 2 mL THF) was added dropwise. The reaction mixture was warmed to room temperature and stirred for 2 hours. Upon completion, the mixture was diluted with petroleum ether (30 mL), filtered through celite, and concentrated *in vacuo*. The resulting mixture was dissolved again with petroleum ether, filtered through a pad of silica gel, washed with 50 mL eluent (Et_2O: petroleum ether = 1:1), and concentrated under reduced pressure. The crude mixture was purified by column chromatography on silica gel (PE/EA) to obtain the corresponding iododiboron substrate.

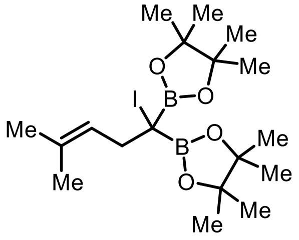

2,2'-(1-iodo-4-methylpent-3-ene-1,1-diyl)bis(4,4,5,5-tetramethyl-1,3,2-dioxaborolane) (2f)
The compound **2f** was prepared according to **Method A** with 2,2'-(4-methylpent-3-ene-1,1-diyl)bis(4,4,5,5-tetramethyl-1,3,2-dioxaborolane) (6.4 mmol) and was purified by flash column chromatography using the eluent (PE:EA = 20:1) to afford a light yellow solid (2.31 g, 78%).
^{1}H NMR ($CDCl_3$, 600 MHz): δ 5.16 (t, J = 6.9 Hz, 1H), 2.64 (d, J = 7.0 Hz, 2H), 1.63 (s, 3H), 1.60 (s, 3H), 1.25 (s, 12H), 1.24 (s, 12H); **^{13}C NMR** ($CDCl_3$, 150 MHz): δ 133.2, 124.5, 84.1, 35.1, 25.9, 24.4, 24.3, 18.5; **^{11}B NMR** ($CDCl_3$, 193 MHz): δ 32.6; **HRMS** (ESI-TOF): *m/z* calculated for $C_{18}H_{33}B_2INaO_4^+$ $[M+Na]^+$ 485.1502, found 485.1511.

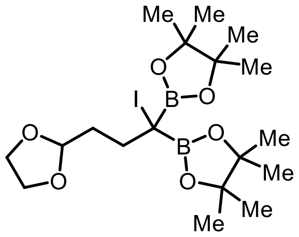

2,2'-(3-(1,3-dioxolan-2-yl)-1-iodopropane-1,1-diyl)bis(4,4,5,5-tetramethyl-1,3,2-dioxaborolane) (2j)
The compound **2j** was prepared according to **Method A** with 2,2'-(3-(1,3-dioxolan-2-yl)propane-1,1-diyl)bis(4,4,5,5-tetramethyl-1,3,2-dioxaborolane) (2.0 mmol) and was purified by flash column chromatography using the eluent (PE:EA = 6:1 to 4:1, stain in CAM) to afford a white solid (730 mg, 74%).
^{1}H NMR ($CDCl_3$, 600 MHz): δ 4.89 (t, J = 4.8 Hz, 1H), 3.99-3.94 (m, 2H), 3.86-3.81F (m, 2H), 1.98-1.96 (m, 2H), 1.84-1.81 (m, 2H), 1.25 (s, 12H), 1.24 (s, 12H); **^{13}C NMR** ($CDCl_3$, 150 MHz): δ 104.3, 84.2, 64.7, 35.2, 31.2, 24.5, 24.3; **^{11}B NMR** ($CDCl_3$, 193 MHz):δ 32.8; **HRMS** (ESI-TOF): *m/z* calculated for $C_{18}H_{33}B_2INaO_6^+$ $[M+Na]^+$ 517.1400, found 517.1402.

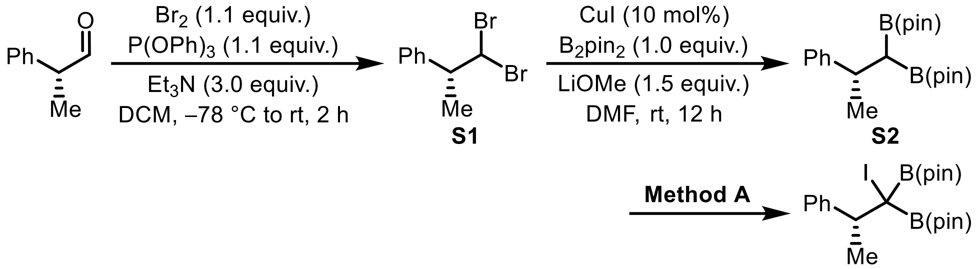

2n

STEP I: The reaction was performed according to the literature procedure with slight modifications.[3] To a stirred solution of triphenyl phosphite (7.51 g, 24.2 mmol) in anhydrous DCM (220 mL), bromine (3.87 g, 24.2 mmol) was added dropwise at –78 °C under argon. Freshly distilled triethylamine (9.2 mL, 66 mmol) and (*R*)-2-phenylpropanal (2.95 g, 22 mmol) were added at –78 °C. The reaction mixture was allowed to warm to room temperature and stirred for 2 hours. Upon completion, the solvent was evaporated *in vacuo* and the crude reaction mixture was purified on silica gel (100% hexanes) to afford (*R*)-(1,1-dibromopropan-2-yl)benzene **S1** (1.11 g, 18%).

STEP II: In the glove box, an oven-dried 50 mL round-bottom flask with a magnetic stir bar was charged with CuI (9.5 mg, 0.2 mmol), LiOMe (228 mg, 6.0 mmol) and $B_2(pin)_2$ (2.03 g, 8.0 mmol). The flask was sealed with a rubber septum, removed from the glove box, followed by the addition of DMF (8 mL) under argon. After stirring at room temperature for 10 minutes, a solution of **S1** (1.11 g, 4.0 mmol) was added via syringe at room temperature. The reaction mixture was allowed to stir at room temperature for 12 hours. Upon completion, 20 mL diethyl ether was added. The slurry was filtered through a silica gel plug, rinsed with diethyl ether, and concentrated *in vacuo*. The crude reaction mixture (DMF solution) was directly purified on silica gel (petroleum ether/EA) to afford the *gem*-diboron product **S2** as a white solid (796 mg, 54%).

STEP III: The compound **2n** was prepared according to **Method A** with **S2** (2.0 mmol) and was purified by flash column chromatography using the eluent (PE:EA = 20:1 to 18:1) to afford a white solid (355 mg, 36%).

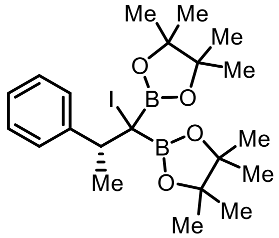

(*R*)-2,2'-(1-iodo-2-phenylpropane-1,1-diyl)bis(4,4,5,5-tetramethyl-1,3,2-dioxaborolane) (2n)

^{1}H NMR ($CDCl_3$, 500 MHz): δ 7.47-7.44 (m, 2H), 7.25-7.22 (m, 2H, overlapped with $CHCl_3$ signal), 7.19-7.16 (m, 1H), 3.10 (q, J = 7.0 Hz, 1H), 1.52 (d, J = 7.0 Hz, 3H), 1.25 (s, 12H), 1.20 (s, 6H), 1.16 (s, 6H); **^{13}C NMR** ($CDCl_3$, 150 MHz): δ 145.9, 128.4, 127.6, 126.4, 84.2, 84.1, 44.6, 24.5, 24.43, 24.41, 24.3, 22.7; **^{11}B NMR** ($CDCl_3$, 193 MHz): δ 32.4; **HRMS** (ESI-TOF): *m/z* calculated for $C_{21}H_{33}B_2INaO_4^+$ $[M+Na]^+$ 521.1502, found 521.1515.

Chiral SFC (IG-3, Chiralcel, 2 mL/min, 3% MeOH, 2000 psi, 35 °C), λ = 220 nm, tr = 3.63 min (major), 5.28 min (minor): 92% ee.

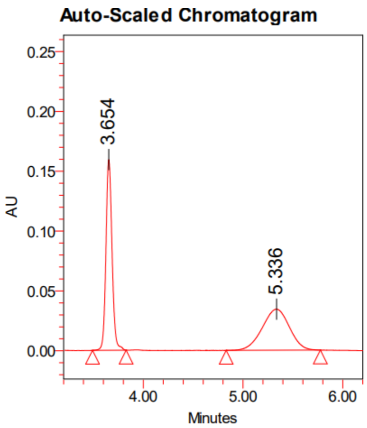

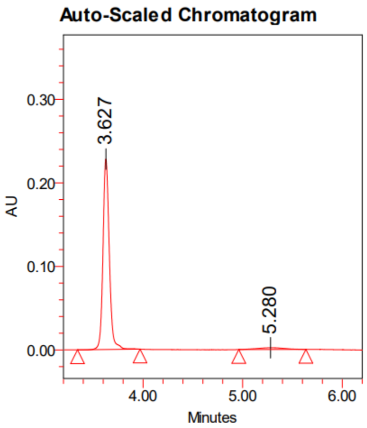

Peak Results

	RT	Area	Height	% Area
1	3.654	676223	159401	50.96
2	5.336	650710	34319	49.04

Peak Results

	RT	Area	Height	% Area
1	3.627	1005075	228020	96.15
2	5.280	40194	2088	3.85

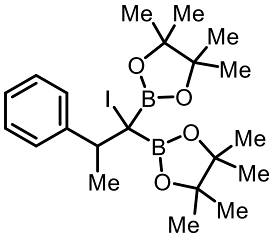

2,2'-(1-iodo-2-phenylpropane-1,1-diyl)bis(4,4,5,5-tetramethyl-1,3,2-dioxaborolane)

^{1}**H NMR**($CDCl_3$, 600 MHz): δ 7.48-7.43 (m, 2H), 7.26-7.22 (m, 2H), 7.20-7.16 (m, 1H), 3.10 (q, J = 7.0 Hz, 1H), 1.52 (d, J = 7.0 Hz, 3H), 1.25 (s, 12H), 1.20 (s, 6H), 1.16 (s, 6H)

Racemates of **2n** was prepared according to **Method B** and have been previously synthesized and characterized.[2]

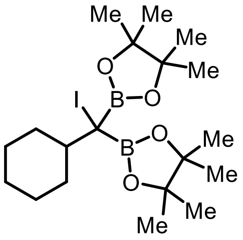

2,2'-(cyclohexyliodomethylene)bis(4,4,5,5-tetramethyl-1,3,2-dioxaborolane) (**2r**)

The compound **2r** was prepared according to **Method A** with 2,2'-(cyclohexylmethylene)bis(4,4,5,5-tetramethyl-1,3,2-dioxaborolane) (2.0 mmol) and was purified by flash column chromatography using the eluent (PE:EA = 30:1, stain in CAM) to afford a white solid (797 mg, 84%).

^{1}**H NMR** ($CDCl_3$, 500 MHz): δ 1.89-1.87 (m, 2H), 1.72-1.68 (m, 2H), 1.60-1.57 (m, 1H, overlapped with H_2O signal), 1.42-1.21 (m, 29H), 1.15-1.07 (m, 1H); ^{13}**C NMR** ($CDCl_3$, 150 MHz): δ 84.0, 43.5, 33.0, 26.7, 26.3, 24.5, 24.4; ^{11}**B NMR** ($CDCl_3$, 193 MHz): δ 32.5; **HRMS** (ESI-TOF): m/z calculated for $C_{19}H_{35}B_2INaO_4^+$ $[M+Na]^+$ 499.1658, found 499.1662.

Method B

Iododiboron substrates **2d-2e**, **2g**, **2j** and **2p** were prepared according to **Method B** and have been previously synthesized and characterized.[2][4]

Procedure:

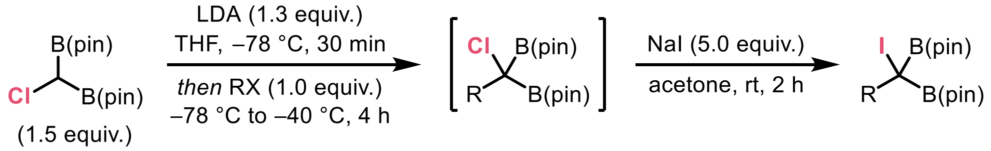

STEP I: The reaction was performed according to the procedure in the literature with slight modifications.[4] A 100 mL over-dried round-bottom flask with a magnetic stir bar was charged with 2,2'-(chloromethylene)bis(4,4,5,5-tetramethyl-1,3,2-dioxaborolane) (907.2 mg, 3.0 mmol, 1.5 equiv.). The vessel was evacuated and refilled with argon for three cycles, followed by the addition of THF (0.1 M). The reaction mixture was cooled to –78 °C, and 2.0 M LDA (1.3 mL, 1.3 equiv.) was added via syringe slowly. After stirring at –78 °C for 30 minutes, alkyl halide (X = Br or I, 2.0 mmol, 1.0 equiv.) was added via syringe (if the halide was a solid, it was added as a THF solution), then warmed to –40 °C and stirred for 4 hours. Upon completion, the reaction was quenched with sat. NH_4Cl solution (20 mL) and extracted with Et_2O (3 × 20 mL). The combined organic layers were dried over Na_2SO_4, filtered, and concentrated *in vacuo*. The crude product was used directly in the iodide exchange.

STEP II: A 100 mL over-dried round-bottom flask with a magnetic stir bar was charged with the chlorodiboron crude product from Step I, and NaI (1.50 g, 5.0 equiv.). The flask was sealed with a rubber septum, evacuated, and refilled with argon (three cycles), and acetone (0.1 M) was added via syringe. The resulting reaction mixture was stirred at 25 ºC for 2 hours, then the solvent was removed under vacuum, and the crude reaction mixture was purified by column chromatography on silica gel (petroleum ether/EA) to afford the desired product.

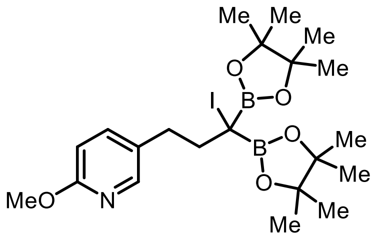

5-(3-iodo-3,3-bis(4,4,5,5-tetramethyl-1,3,2-dioxaborolan-2-yl)propyl)-2-methoxypyridine (2c)

The compound **2c** was prepared according to **Method A** with 5-(3,3-bis(4,4,5,5-tetramethyl-1,3,2-dioxaborolan-2-yl)propyl)-2-methoxypyridine (2.0 mmol) and was purified by flash column chromatography using the eluent (PE:EA = 15:1 to 12:1) to afford a white solid (577 mg, 55%).

^{1}H NMR ($CDCl_3$, 500 MHz): δ 7.99 (d, J = 2.4 Hz, 1H), 7.44 (dd, J = 8.4, 2.5 Hz, 1H), 6.66 (d, J = 8.4 Hz, 1H), 3.90 (s, 3H), 2.66-2.63 (m, 2H), 2.08-2.05 (m, 2H), 1.27 (s, 12H), 1.26 (s, 12H); **^{13}C NMR** ($CDCl_3$, 125 MHz): δ 162.6, 146.2, 139.1, 130.1, 110.3, 84.3, 53.2, 38.8, 34.0, 24.5, 24.3; **^{11}B NMR** ($CDCl_3$, 161 MHz): δ 32.8; **HRMS** (ESI-TOF): *m/z* calculated for $C_{21}H_{34}B_2INNaO_5^+$ $[M+Na]^+$ 552.1560, found 552.1574.

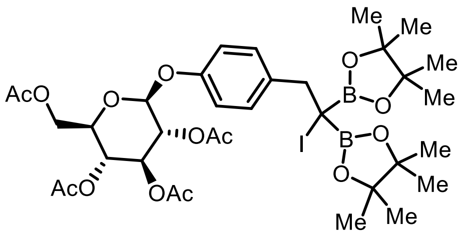

(2*R*, 3*R*, 4*S*, 5*R*, 6*S*)-2-(acetoxymethyl)-6-(4-(2-iodo-2,2-bis(4,4,5,5-tetramethyl-1,3,2-dioxaborolan-2-yl)ethyl)phenoxy)tetrahydro-2*H*-pyran-3,4,5-triyl triacetate (2t)

The compound **2t** was prepared according to **Method B** with (2*R*,3*R*,4*S*,5*R*,6*S*)-2-(acetoxymethyl)-6-(4-(2,2-bis(4,4,5,5-tetramethyl-1,3,2-dioxaborolan-2-yl)ethyl)phenoxy)tetrahydro-2H-pyran-3,4,5-triyl triacetate (1.2 mmol) and was purified by flash column chromatography using the eluent (PE:EA = 3:1 to 2:1) to afford a white solid (667.6 mg, 67%).

^{1}H NMR ($CDCl_3$, 600 MHz): δ 7.31 (d, *J* = 8.6 Hz, 2H), 6.87 (d, *J* = 8.6 Hz, 2H), 5.30-5.24 (m, 2H), 5.18-5.14 (m, 1H), 5.04 (d, *J* = 7.4 Hz, 1H), 4.29 (dd, *J* = 12.3, 5.3 Hz, 1H), 4.16 (dd, *J* = 12.3, 2.4 Hz, 1H), 3.86-3.83 (m, 1H), 3.24 (s, 2 H), 2.08 (s, 3H), 2.06 (s, 3H), 2.05 (s, 3H), 2.03 (s, 3H), 1.25 (s, 12H), 1.24 (s, 12H); **^{13}C NMR** ($CDCl_3$, 150 MHz): δ 170.5, 170.2, 169.4, 169.3, 155.5, 136.2, 130.9, 116.1, 99.2, 84.3, 72.7, 71.9, 71.1, 68.3, 61.9, 40.6, 24.4, 20.6, 20.58, 20.55, 20.52; **^{11}B NMR** ($CDCl_3$, 193 MHz): δ 32.6; **HRMS** (ESI-TOF): *m/z* calculated for $C_{34}H_{49}B_2INaO_{14}^+$ $[M+Na]^+$ 853.2245, found 853.2260.

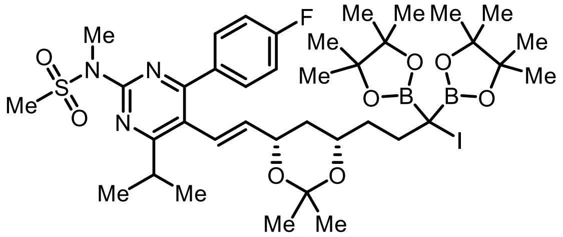

***N*-(4-(4-fluorophenyl)-5-((*E*)-2-((4*S*, 6*S*)-6-(3-iodo-3,3-bis(4,4,5,5-tetramethyl-1,3,2-dioxaborolan-2-yl)propyl)-2,2-dimethyl-1,3-dioxan-4-yl)vinyl)-6-isopropylpyrimidin-2-yl)-*N*-methylmethanesulfonamide (2o)**

The compound **2o** was prepared according to **Method B** with *N*-(5-((*E*)-2-((4*S*, 6*S*)-6-(3,3-bis(4,4,5,5-tetramethyl-1,3,2-dioxaborolan-2-yl)propyl)-2,2-dimethyl-1,3-dioxan-4-yl)vinyl)-4-(4-fluorophenyl)-6-isopropylpyrimidin-2-yl)-*N*-methylmethanesulfonamide (1.45 mmol) and was purified by flash column chromatography using the eluent (PE:EA = 8:1 to 7:1) to afford a pale yellow solid (624.2 mg, 49%).

^{1}H NMR ($CDCl_3$, 500 MHz): δ 7.69-7.66 (m, 2H), 7.12-7.08 (m, 2H), 6.49 (dd, *J* = 16.3, 1.5 Hz, 1H), 5.52 (dd, *J* = 16.2, 5.3 Hz, 1H), 4.40-4.36 (m, 1H), 3.86-3.81 (m, 1H), 3.57 (s, 3H), 3.52 (s, 3H), 3.44-3.38 (m, 1H), 1.96-1.91 (m, 1H), 1.82-1.77 (m, 1H), 1.71-1.65 (m, 1H, overlapped with H_2O signal), 1.54-1.50 (m, 2H), 1.45 (s, 3H), 1.42 (s, 3H), 1.28-1.24 (m, 30H), 1.16-1.10 (m, 1H); **^{13}C NMR** ($CDCl_3$, 150 MHz): δ 174.8, 163.4, 163.2 (d, *J* = 250.0 Hz), 157.2, 137.8, 134.3 (d, *J* = 3.3 Hz), 132.2 (d, *J* = 8.5 Hz), 122.9, 121.2, 114.9 (d, *J* = 21.6 Hz), 98.6, 84.21, 84.19, 69.4, 68.7, 42.4, 37.8, 36.4, 33.0, 32.1, 31.8, 30.1, 24.4, 24.3, 24.2, 21.77, 21.71, 19.8; **^{11}B NMR** ($CDCl_3$, 193 MHz): δ 32.0; **^{19}F NMR** ($CDCl_3$, 565 MHz): δ –111.4; **HRMS** (ESI-TOF): *m/z* calculated for $C_{38}H_{57}B_2FIN_3NaO_8S^+$ $[M+Na]^+$ 906.2973, found 906.2998.

Method C

Bromodiboron substrates **2m** was prepared according to **Method C** and have been previously synthesized and characterized.[5]

Procedure:

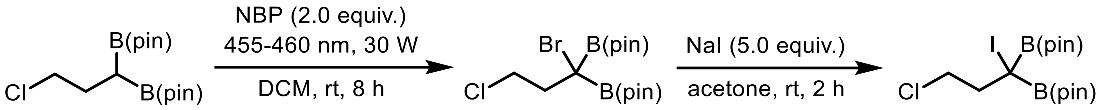

STEP I: The following iododiboron substrate **2h** was prepared using our previously reported method[5] with slight modifications. An oven-dried 20 mL vial with a magnetic stir bar was charged with 2,2'-(3-chloropropane-1,1-diyl)bis(4,4,5,5-tetramethyl-1,3,2-dioxaborolane) (330.5 mg, 1.0 mmol) and 2-bromoisoindoline-1,3-dione (452.1 mg, 2.0 mmol). The vial was sealed with a polypropylene open-top cap with PTFE/silicone septum, evacuated, and backfilled with argon three times, then anhydrous DCM (5 mL) was added. The reaction mixture was then irradiated under blue light (450-455 nm, 30 W) 8 h at 25 ºC. Upon completion, the solvent was removed under vacuum. The resulting mixture was filtered through celite, rinsed with petroleum ether (2 mL×3), and the combined filtrates were concentrated *in vacuo*.
STEP II: A 50 mL over-dried round-bottom flask with a magnetic stir bar was charged with the bromodiboron crude product, and NaI (749.5 mg, 5.0 mmol). The flask was sealed with a rubber septum, evacuated, and refilled with argon (three cycles), and acetone (10 mL) was added via syringe. The resulting reaction mixture was stirred at 25 ºC for 2 hours, then the solvent was removed under vacuum, and the crude reaction mixture was purified by column chromatography on silica gel (petroleum ether/EA) to afford the desired product as a white solid (168.9 mg, 37%).

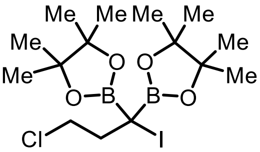

2,2'-(3-chloro-1-iodopropane-1,1-diyl)bis(4,4,5,5-tetramethyl-1,3,2-dioxaborolane) (2h)
^{1}H NMR ($CDCl_3$, 500 MHz): δ 3.68-3.65 (m, 2H), 2.29-2.26 (m, 2H), 1.26 (s, 12H), 1.25 (s, 12H); **^{13}C NMR** ($CDCl_3$, 150 MHz): δ 84.5, 46.1, 38.8, 24.4, 24.3; **^{11}B NMR** ($CDCl_3$, 193 MHz): δ 32.5; **HRMS** (ESI-TOF): *m/z* calculated for $C_{15}H_{28}B_2ClINaO_4^+$ $[M+Na]^+$ 479.0799, found 479.0810.

Method D

Iododiboron substrates **2k** was prepared according to **Method D** and have been previously synthesized and characterized.[2]

Procedure:

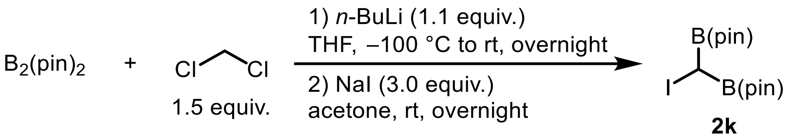

The reaction was performed according to the procedure in the literature with slight modifications.[6] An oven-dried 200 mL round-bottom flask equipped with a stir bar was added DCM (3 mL, 45 mmol) and anhydrous THF (60 mL) under argon atmosphere, then the tube was cooled to –100 °C followed by dropwise addition of *n*-BuLi (22.8 mL, 33 mmol, 1.45 mol/L in *n*-hexane) via syringe. The reaction mixture was stirred at the same temperature for 45 min, then a solution of $B_2(pin)_2$ (7.62 g, 30 mmol) in THF (15 mL) was added at the same temperature. Then the mixture was allowed to slowly warm to room temperature and stirred overnight. After the reaction was completed, a large amount of DCM was added to precipitate LiCl and the solution was filtered and concentrated. Then the residue was dissolved in acetone (150 mL) and NaI (13.49 g, 90 mmol) was added. The mixture was stirred at room temperature overnight. Upon completion, the mixture was quenched with water, extracted with PE three times, and the combined organic layers were washed with saturated brine and dried over anhydrous Na_2SO_4. After the solvent was evaporated, the crude product was purified by recrystallization from *n*-hexane at –30 °C to afford the desired product (9.02 g, 76% yield).

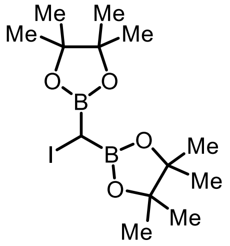

2,2'-(iodomethylene)bis(4,4,5,5-tetramethyl-1,3,2-dioxaborolane) (2k)

^{1}H NMR (600 MHz, $CDCl_3$) δ 2.07 (s, 1H), 1.263 (s, 12H), 1.265 (s, 12H).

The spectral data are in accord with the literature.[6]

II. Palladium-Catalyzed *gem*-Diborylalkylation of Silyl Enol Ethers

Optimization of Reaction Conditions

In the glove box, an oven-dried 10 mL vial with a magnetic stir bar was charged with palladium catalyst (0.01 mmol), ligand (0.02 mmol), and anhydrous solvent (1 mL). The reaction vial was sealed and allowed to stir at ambient temperature for 10 minutes. Then the base (0.2 mmol), trimethyl((1-phenylvinyl)oxy)silane (**3a**, 0.3 mmol) and 2,2'-(1-iodo-3-phenylpropane-1,1-diyl)bis(4,4,5,5-tetramethyl-1,3,2-dioxaborolane) (**2a**, 0.1 mmol) were successively added. The vial was sealed with a polypropylene open-top cap with PTFE/silicone septum, removed from the glove box, charged with H_2O (1 mmol), and stirred at room temperature for 8 hours. Upon completion, the reaction mixture was diluted with ethyl acetate, filtered through a silica gel plug using ethyl acetate as eluent, and concentrated *in vacuo* to afford the crude reaction mixture. The crude yield was determined by ^1H NMR using 1,3,5-trimethoxybenzene as the internal standard.

Table S1. Optimization of Palladium Precatalyst and ligand[a]

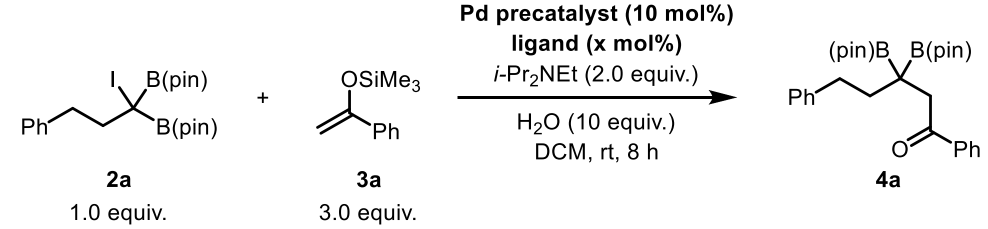

entry	cat.	ligand (x mol%)	remaining **2a** (%)	NMR yield of **4a** (%)
1	$Pd(OAc)_2$	Xantphos (20 mol%)	0	97
2	$Pd(TFA)_2$	Xantphos (20 mol%)	6	93
3	$Pd(dba)_2$	Xantphos (10 mol%)	26	69
4	$Pd(dba)_2$	Xantphos (20 mol%)	96	3
5	$Pd(PPh_3)_4$	/	91	1
6	$Pd(PPh_3)_4$	Xantphos (10 mol%)	20	68
7	$Pd(OAc)_2$	Xantphos (20 mol%) + PPh_3 (40 mol%)	75	18
8	/	/	99	0

[a] Conditions: iododiboron **2a** (0.1 mmol), silyl enol ether **3a** (0.3 mmol), **palladium precatalyst** (0.01 mmol), ligand, *i*-Pr_2NEt (0.2 mmol), H_2O (1.0 mmol), DCM (1 mL), rt, 8 h. The crude yield was determined by ^1H NMR using 1,3,5-trimethoxybenzene as the internal standard.

Table S2. Optimization of Ligand[a]

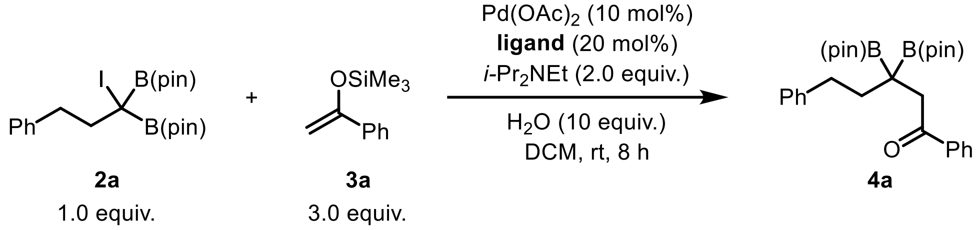

entry	ligand	remaining **2a** (%)	NMR yield of **4a** (%)
1	/	98	0
2	Xantphos	0	97
3	*N*-Xantphos	51	44
4	Cy-Xantphos	80	6
5	(±)-BINAP	93	0
6	dppe	87	0
7	dppf	76	2
8	XPhos	87	7
9	PPh_3	30	53

[a] Conditions: iododiboron **2a** (0.1 mmol), silyl enol ether **3a** (0.3 mmol), $Pd(OAc)_2$ (0.01 mmol), **ligand** (0.02 mmol), *i*-Pr_2NEt (0.2 mmol), H_2O (1.0 mmol), DCM (1 mL), rt, 8 h. The crude yield was determined by ^1H NMR using 1,3,5-trimethoxybenzene as the internal standard.

Table S3. Optimization of Solvent[a]

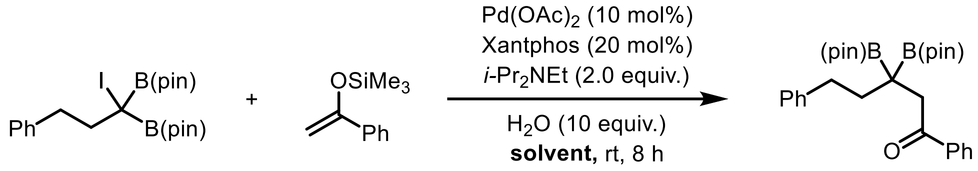

2a 1.0 equiv. **3a** 3.0 equiv. **4a**

entry	solvent	remaining **2a** (%)	NMR yield of **4a** (%)
1	DCM	0	97 (97)[b]
2	1,4-dioxane	10	84
3	THF	6	83
4	DME	22	61
5	EA	56	26
6	toluene	73	7
7	MeCN	62	3

[a] Conditions: iododiboron **2a** (0.1 mmol), silyl enol ether **3a** (0.3 mmol), $Pd(OAc)_2$ (0.01 mmol), Xantphos (0.02 mmol), *i*-Pr_2NEt (0.2 mmol), H_2O (1.0 mmol), **solvent** (1 mL), rt, 8 h. The crude yield was determined by ^1H NMR using 1,3,5-trimethoxybenzene as the internal standard. [b] isolated yield in the parentheses.

Table S4. Optimization of Base[a]

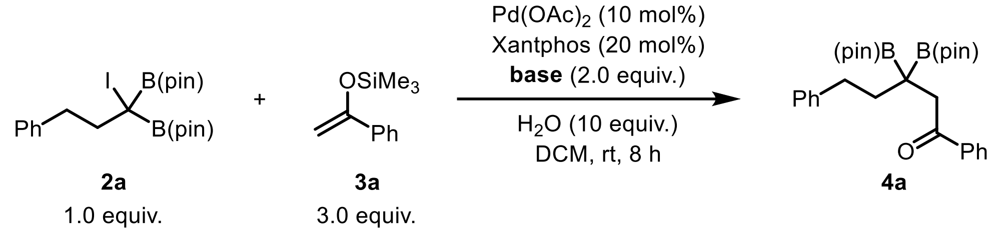

entry	base	remaining **2a** (%)	NMR yield of **4a** (%)
1	/	96	0
2	*i*-Pr_2NEt	0	97
3	*i*-Pr_2NEt	0	97[b]
4	*i*-Pr_2NEt	42	54[c]
5	Et_3N	0	96
6	*i*-Pr_2NH	0	96
7	*i*-$PrNH_2$	0	83
8	CsF	23	74
9	Na_2CO_3	30	70
10	K_3PO_4	4	95

[a] Conditions: iododiboron **2a** (0.1 mmol), silyl enol ether **3a** (0.3 mmol), $Pd(OAc)_2$ (0.01 mmol), Xantphos (0.02 mmol), **base** (0.2 mmol), H_2O (1.0 mmol), DCM (1 mL), rt, 8 h. The crude yield was determined by ^{1}H NMR using 1,3,5-trimethoxybenzene as the internal standard. [b] *i*-Pr_2NEt (0.1 mmol). [c] *i*-Pr_2NEt (0.05 mmol).

Experimental Procedures and Spectral Data

General Procedure A:

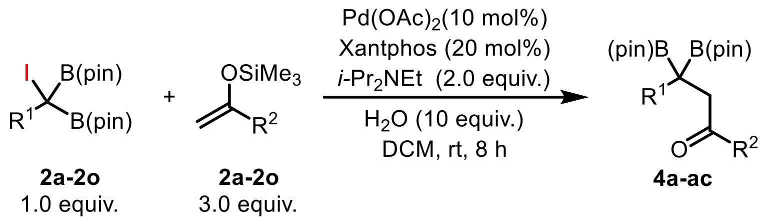

In the glove box, an oven-dried 10 mL vial with a magnetic stir bar was charged with $Pd(OAc)_2$ (2.2 mg, 0.01 mmol), Xantphos (11.6 mg, 0.02 mmol) and anhydrous DCM (1 mL). The vial was sealed and allowed to stir at ambient temperature for 10 minutes. Then, *i*-Pr_2NEt (35 µL, 0.2 mmol), silyl enol ether (0.3 mmol), and iododiboron substrate (0.1 mmol) were successively added. The vial was sealed with a polypropylene open-top cap with PTFE/silicone septum, removed from the glove box, charged with H_2O (18 µL, 1 mmol), and stirred at room temperature for 8 hours. Upon completion, the reaction mixture was diluted with ethyl acetate, filtered through a silica gel plug using ethyl acetate as eluent, and concentrated *in vacuo*. The crude reaction mixture was purified by column chromatography on silica gel using petroleum ether/ethyl acetate as the eluent to afford the desired product.

Spectral Data:

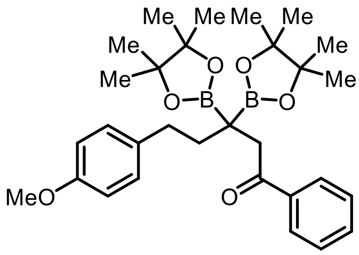

5-(4-methoxyphenyl)-1-phenyl-3,3-bis(4,4,5,5-tetramethyl-1,3,2-dioxaborolan-2-yl)pentan-1-one (4b)

The title compound was prepared according to ***General Procedure A*** with **2b** (52.8 mg, 0.1 mmol) and **3a** (57.7 mg, 0.3 mmol). The crude reaction mixture was purified by column chromatography (PE:EA = 12:1, stain in CAM) to afford a white solid (50.4 mg, 97%).

^{1}H NMR ($CDCl_3$, 600 MHz): δ 7.98-7.97 (m, 2H), 7.54-7.51 (m, 1H), 7.44-7.42 (m, 2H), 7.00 (d, J = 8.6 Hz, 2H), 6.71 (d, J = 8.6 Hz, 2H), 3.72 (s, 3H), 3.42 (s, 2H), 2.45-2.42 (m, 2H), 1.99-1.96 (m, 2H), 1.28 (s, 12H), 1.25 (s, 12H); **^{13}C NMR** ($CDCl_3$, 150 MHz): δ 200.0, 157.4, 137.2, 135.3, 132.6, 129.2, 128.3, 128.1, 113.5, 83.1, 55.2, 40.3, 33.5, 32.6, 24.8, 24.7; **^{11}B NMR** ($CDCl_3$, 193 MHz): δ 34.6; **HRMS** (ESI-TOF): *m/z* calculated for $C_{30}H_{42}B_2NaO_6^+$ $[M+Na]^+$ 543.3060, found 543.3080.

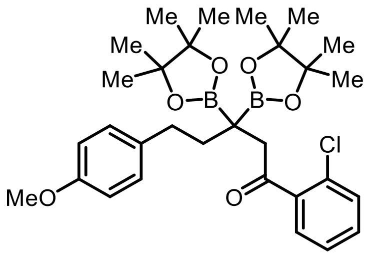

1-(2-chlorophenyl)-5-(4-methoxyphenyl)-3,3-bis(4,4,5,5-tetramethyl-1,3,2-dioxaborolan-2-yl)pentan-1-one (**4c**)

The title compound was prepared according to ***General Procedure A*** with **2b** (52.8 mg, 0.1 mmol) and **3b** (68.0 mg, 0.3 mmol). The crude reaction mixture was purified by column chromatography (PE:EA = 12:1, stain in CAM) to afford a white solid (39.7 mg, 72%).

^{1}H NMR ($CDCl_3$, 600 MHz): δ 7.40-7.38 (m, 2H), 7.35-7.32 (m, 1H), 7.30-7.27 (m, 1H), 7.06 (d, J = 8.5 Hz, 2H), 6.76 (d, J = 8.5 Hz, 2H), 3.75 (s, 3H), 3.35 (s, 2H), 2.48-2.46 (m, 2H), 2.03-2.00 (m, 2H), 1.27 (s, 12H), 1.25 (s, 12H); **^{13}C NMR** ($CDCl_3$, 150 MHz): δ 203.5, 157.5, 139.9, 135.3, 131.0, 130.5, 130.2, 129.3, 128.4, 126.6, 113.6, 83.2, 55.2, 44.7, 33.5, 32.6, 24.8, 24.7; **^{11}B NMR** ($CDCl_3$, 193 MHz): δ 34.2; **HRMS** (ESI-TOF): *m/z* calculated for $C_{30}H_{41}B_2ClNaO_6^+$ $[M+Na]^+$ 577.2670, found 577.2680.

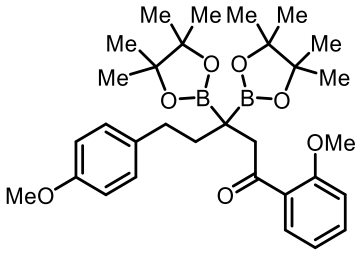

1-(2-methoxyphenyl)-5-(4-methoxyphenyl)-3,3-bis(4,4,5,5-tetramethyl-1,3,2-dioxaborolan-2-yl)pentan-1-one (**4d**)

The title compound was prepared according to ***General Procedure A*** with **2b** (52.8 mg, 0.1 mmol) and **3c** (66.7 mg, 0.3 mmol). The crude reaction mixture was purified by column chromatography (PE:EA = 10:1 to 8:1, stain in CAM) to afford a reddish-brown solid (53.0 mg, 96%).

^{1}H NMR ($CDCl_3$, 500 MHz): δ 7.56-7.55 (m, 1H), 7.42-7.39 (m, 1H), 7.03 (d, J = 8.6 Hz, 2H), 6.97-6.92 (m, 2H), 6.73 (d, J = 8.6 Hz, 2H), 3.88 (s, 3H), 3.74 (s, 3H), 3.41 (s, 2H), 2.46-2.43 (m, 2H), 1.96-1.92 (m, 2H), 1.28 (s, 12H), 1.26 (s, 12H); **^{13}C NMR** ($CDCl_3$, 125 MHz): δ 203.2, 158.1, 157.4, 135. 7, 132.6, 129.6, 129.3, 129.2, 120.4, 113.4, 111.3, 83.0, 55.4, 55.2, 45.3, 33.4, 32.9, 24.8, 24.7; **^{11}B NMR** ($CDCl_3$, 193 MHz): δ 34.4; **HRMS** (ESI-TOF): *m/z* calculated for $C_{31}H_{44}B_2NaO_7^+$ $[M+Na]^+$ 573.3165, found 573.3170.

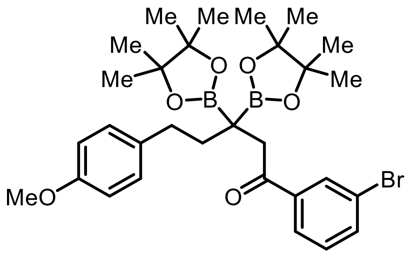

1-(3-bromophenyl)-5-(4-methoxyphenyl)-3,3-bis(4,4,5,5-tetramethyl-1,3,2-dioxaborolan-2-yl)pentan-1-one (**4e**)

The title compound was prepared according to ***General Procedure A*** with **2b** (52.8 mg, 0.1 mmol) and **3d** (81.4 mg, 0.3 mmol). The crude reaction mixture was purified by column chromatography (PE:EA = 12:1, stain in CAM) to afford a light yellow solid (30.6 mg, 51%). **^{1}H NMR** ($CDCl_3$, 600 MHz): δ 8.08-8.07 (m, 1H), 7.89-7.87 (m, 1H), 7.66-7.64 (m, 1H), 7.32-7.29 (m, 1H), 7.00 (d, J = 8.5 Hz, 2H), 6.71 (d, J = 8.5 Hz, 2H), 3.72 (s, 3H), 3.36 (s, 2H), 2.45-2.42 (m, 2H), 1.99-1.96 (m, 2H), 1.28 (s, 12H), 1.25 (s, 12H); **^{13}C NMR** ($CDCl_3$, 150 MHz): δ 198.7, 157.5, 138.9, 135.5, 135.2, 131.0, 129.9, 129.2, 126.6, 122.7, 113.5, 83.2, 55.2, 40.3, 33.5, 32.4, 24.8, 24.7; **^{11}B NMR** ($CDCl_3$, 193 MHz): δ 33.7; **HRMS** (ESI-TOF): *m/z* calculated for $C_{30}H_{41}B_2Br_2NaO_6^+$ $[M+Na]^+$ 621.2165, found 621.2170.

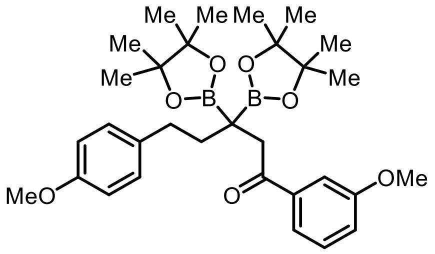

1-(3-methoxyphenyl)-5-(4-methoxyphenyl)-3,3-bis(4,4,5,5-tetramethyl-1,3,2-dioxaborolan-2-yl)pentan-1-one (**4f**)

The title compound was prepared according to ***General Procedure A*** with **2b** (52.8 mg, 0.1 mmol) and **3e** (66.7 mg, 0.3 mmol). The crude reaction mixture was purified by column chromatography (PE:EA = 10:1 to 6:1, stain in CAM) to afford a white solid (53.4mg, 97%). **^{1}H NMR** ($CDCl_3$, 600 MHz): δ 7.59-7.57 (m, 1H), 7.49-7.48 (m, 1H), 7.35-7.33 (m, 1H), 7.09-7.07 (m, 1H), 7.00 (d, J = 8.6 Hz, 2H), 6.71 (d, J = 8.6 Hz, 2H), 3.85 (s, 3H), 3.72 (s, 3H), 3.40 (s, 2H), 2.44-2.41 (m, 2H), 1.98-1.95 (m, 2H), 1.28 (s, 12H), 1.25 (s, 12H); **^{13}C NMR** ($CDCl_3$, 150 MHz): δ199.8, 159.6, 157.4, 138.6, 135.3, 129.3, 129.2, 120.8, 119.0, 113. 5, 112.4, 83.1, 55.4, 55.1, 40.4, 33.4, 32.6, 24.8, 24.7; **^{11}B NMR** ($CDCl_3$, 193 MHz): δ 34.1; **HRMS** (ESI-TOF): *m/z* calculated for $C_{31}H_{44}B_2NaO_7^+$ $[M+Na]^+$ 573.3165, found 573.3168.

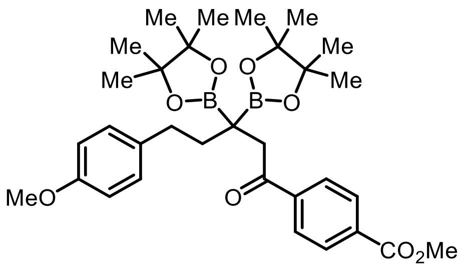

methyl 4-(5-(4-methoxyphenyl)-3,3-bis(4,4,5,5-tetramethyl-1,3,2-dioxaborolan-2-yl)pentanoyl)benzoate (4g)

The title compound was prepared according to ***General Procedure A*** with **2b** (52.8 mg, 0.1 mmol) and **3f** (75.1mg, 0.3 mmol). The crude reaction mixture was purified by column chromatography (PE:EA = 8:1, stain in CAM) to afford a white solid (51.3 mg, 89%).

^{1}H NMR ($CDCl_3$, 600 MHz): δ 8.09 (d, *J* = 8.4 Hz, 2H), 8.00 (d, *J* = 8.4 Hz, 2H), 7.00 (d, *J* = 8.5 Hz, 2H), 6.70 (d, *J* = 8.5 Hz, 2H), 3.95 (s, 3H), 3.71 (s, 3H), 3.42 (s, 2H), 2.45-2.43 (m, 2H), 2.00-1.97 (m, 2H), 1.28 (s, 12H), 1.26 (s, 12H); **^{13}C NMR** ($CDCl_3$, 150 MHz): δ 199.6, 166.3, 157.5, 140.5, 135.2, 133.4, 129.6, 129.2, 127.9, 113.5, 83.2, 55.1, 52.3, 40.6, 33.5, 32.4, 24.8, 24.7; **^{11}B NMR** ($CDCl_3$, 193 MHz): δ 34.1; **HRMS** (ESI-TOF): *m/z* calculated for $C_{32}H_{44}B_2NaO_8^+$ $[M+Na]^+$ 601.3115, found 601.3134.

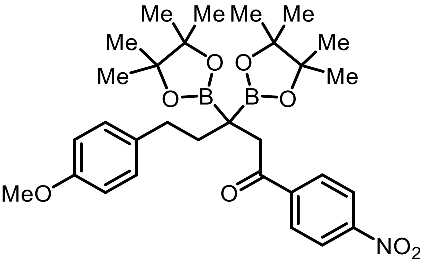

5-(4-methoxyphenyl)-1-(4-nitrophenyl)-3,3-bis(4,4,5,5-tetramethyl-1,3,2-dioxaborolan-2-yl)pentan-1-one (4h)

The title compound was prepared according to ***General Procedure A*** with **2b** (52.8 mg, 0.1 mmol) and **3g** (71.2 mg, 0.3 mmol) at 40 °C for 14 hours. The crude reaction mixture was purified by column chromatography (PE:EA:DCM = 40:16:1 to 20:10:1, stain in CAM) to afford a pale yellow solid (34.5 mg, 63%).

^{1}H NMR ($CDCl_3$, 500 MHz): δ 8.26 (d, *J* = 8.6 Hz, 2H), 8.07 (d, *J* = 8.6 Hz, 2H), 7.00 (d, *J* = 8.6 Hz, 2H), 6.69 (d, *J* = 8.6 Hz, 2H), 3.70 (s, 3H), 3.40 (s, 2H), 2.47-2.43 (m, 2H), 2.02-1.99 (m, 2H), 1.28 (s, 12H), 1.26 (s, 12H); **^{13}C NMR** ($CDCl_3$, 150 MHz): δ 198.6, 157.5, 150.1, 141.7, 135.0, 129.2, 129.0, 123.5, 113.5, 83.3, 55.1, 40.8, 33.6, 32.2, 24.8, 24.7; **^{11}B NMR** ($CDCl_3$, 193 MHz): δ 34.0; **HRMS** (ESI-TOF): *m/z* calculated for $C_{30}H_{41}B_2NNaO_8^+$ $[M+Na]^+$ 588.2910, found 588.2931.

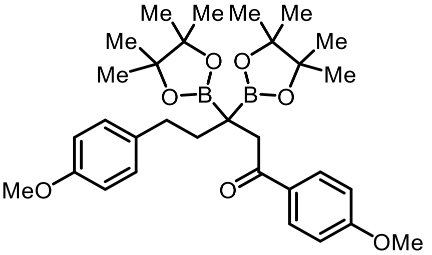

1,5-bis(4-methoxyphenyl)-3,3-bis(4,4,5,5-tetramethyl-1,3,2-dioxaborolan-2-yl)pentan-1-one (4i)

The title compound was prepared according to ***General Procedure A*** with **2b** (52.8 mg, 0.1 mmol) and **3h** (66.7 mg, 0.3 mmol). The crude reaction mixture was purified by column chromatography (PE:EA = 8:1 to 6:1, stain in CAM) to afford a reddish-brown solid (52.0 mg,

94%).
^{1}H NMR ($CDCl_3$, 600 MHz): δ 7.97 (d, *J* = 8.6 Hz, 2H), 6.99 (d, *J* = 8.3 Hz, 2H), 6.90 (d, *J* = 8.5 Hz, 2H), 6.70 d, *J* = 8.2 Hz, 2H), 3.86 (s, 3H), 3.72 (s, 3H), 3.37 (s, 2H), 2.44-2.41 (m, 2H), 1.96-1.93f (m, 2H), 1.28 (s, 12H), 1.25(s, 12H); **^{13}C NMR** ($CDCl_3$, 150 MHz): δ 198.5, 163.1, 157.4, 135.4, 130.3, 130.2, 129.2, 113.44, 113.40, 83.0, 55.4, 55.2, 39.9, 33.5, 32.7, 24.8, 24.7; **^{11}B NMR** ($CDCl_3$, 193 MHz): δ 34.8; **HRMS** (ESI-TOF): *m/z* calculated for $C_{31}H_{44}B_2NaO_7^+$ $[M+Na]^+$ 573.3165, found 573.3181.

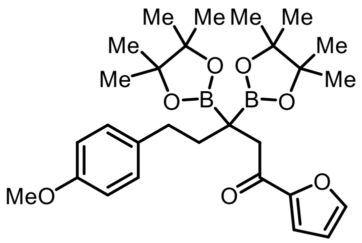

1-(furan-2-yl)-5-(4-methoxyphenyl)-3,3-bis(4,4,5,5-tetramethyl-1,3,2-dioxaborolan-2-yl)pentan-1-one (**4j**)
The title compound was prepared according to ***General Procedure A*** with **2b** (52.8 mg, 0.1 mmol) and **3i** (54.7 mg, 0.3 mmol). The crude reaction mixture was purified by column chromatography (PE:EA = 9:1 to 6:1, stain in CAM) to afford a pale pink solid (49.1 mg, 96%).
^{1}H NMR ($CDCl_3$, 500 MHz): δ 7.56 (d, *J* = 1.6 Hz, 1H), 7.18 (d, *J* = 3.4 Hz, 1H), 7.01 (d, *J* = 8.6 Hz, 2H), 6.72 (d, *J* = 8.6 Hz, 2H), 6.50 (dd, *J* = 3.6, 1.7 Hz, 1H), 3.73 (s, 3H), 3.28 (s, 2H), 2.46-2.42 (m, 2H), 1.96-1.92 (m, 2H), 1.27 (s, 12H), 1.26 (s, 12H); **^{13}C NMR** ($CDCl_3$, 150 MHz): δ 189.1, 157.4, 152.6, 146.0, 135.2, 129.2, 116.7, 113.5, 111.8, 83.2, 55.2, 39.5, 33.4, 32.5, 24.9, 24.6; **^{11}B NMR** ($CDCl_3$, 193 MHz): δ 33.6; **HRMS** (ESI-TOF): *m/z* calculated for $C_{28}H_{40}B_2NaO_7^+$ $[M+Na]^+$ 533.2852, found 533.2870.

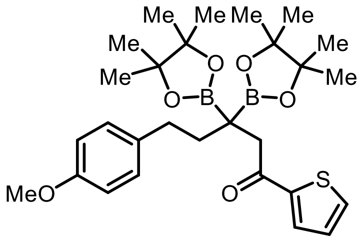

5-(4-methoxyphenyl)-3,3-bis(4,4,5,5-tetramethyl-1,3,2-dioxaborolan-2-yl)-1-(thiophen-2-yl)pentan-1-one (**4k**)
The title compound was prepared according to ***General Procedure A*** with **2b** (52.8 mg, 0.1 mmol) and **3j** (59.5 mg, 0.3 mmol). The crude reaction mixture was purified by column chromatography (PE:EA = 8:1 to 6:1, stain in CAM) to afford a white solid (50.9 mg, 97%).
^{1}H NMR ($CDCl_3$, 500 MHz): δ 7.76 (dd, *J* = 3.8, 1.2 Hz, 1H), 7.58 (dd, *J* = 5.0, 1.1 Hz, 1H), 7.10 (dd, *J* = 5.0, 3.7 Hz, 1H), 7.00 (d, *J* = 8.6 Hz, 2H), 6.71 (d, *J* = 8.7 Hz, 2H), 3.73 (s, 3H), 3.36 (s, 2H), 2.47-2.44 (m, 2H), 1.98-1.94 (m, 2H), 1.27 (s, 12H), 1.25 (s, 12H); **^{13}C NMR** ($CDCl_3$, 150 MHz): δ 192.9, 157.4, 144.2, 135.2, 132.8, 131.7, 129.2, 127.8, 113.5, 83.2, 55.2, 40.6, 33.4, 32.5, 24.8, 24.7; **^{11}B NMR** ($CDCl_3$, 193 MHz): δ 33.4; **HRMS** (ESI-TOF): *m/z* calculated for $C_{28}H_{40}B_2NaO_6S^+$ $[M+Na]^+$ 549.2624, found 549.2636.

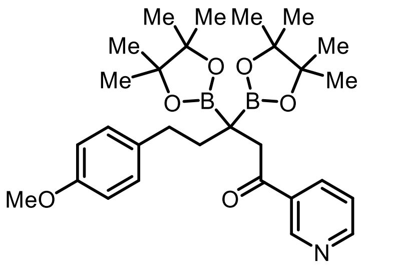

5-(4-methoxyphenyl)-1-(pyridin-3-yl)-3,3-bis(4,4,5,5-tetramethyl-1,3,2-dioxaborolan-2-yl)pentan-1-one (**4l**)

The title compound was prepared according to ***General Procedure A*** with **2b** (52.8 mg, 0.1 mmol) and **3k** (58.0 mg, 0.3 mmol). The crude reaction mixture was purified by column chromatography (PE:EA = 2:1 to 1:1, stain in CAM) to afford a white solid (44.5 mg, 85%). **^{1}H NMR** ($CDCl_3$, 500 MHz): δ 9.16 (s, 1H), 8.75 (d, *J* = 4.0 Hz, 1H), 8.20 (d, *J* = 7.9 Hz, 1H), 7.38 (dd, *J* = 8.0, 4.8 Hz, 1H), 7.01 (d, *J* = 8.6 Hz, 2H), 6.70 (d, *J* = 8.6 Hz, 2H), 3.72 (s, 3H), 3.40 (s, 2H), 2.47-2.44 (m, 2H), 2.01-1.98 (m, 2H), 1.28 (s, 12H), 1.25 (s, 12H); **^{13}C NMR** ($CDCl_3$, 150 MHz): δ 198.9, 157.5, 153.0, 149.5, 135.3, 135.1, 132.3, 129.2, 123.3, 113.5, 83.2, 55.1, 40.5, 33.5, 32.3, 24.8, 24.74, 24.67; **^{11}B NMR** ($CDCl_3$, 193 MHz): δ 33.8; **HRMS** (ESI-TOF): *m/z* calculated for $C_{29}H_{41}B_2NNaO_6^+$ $[M+Na]^+$ 544.3012, found 544.3026.

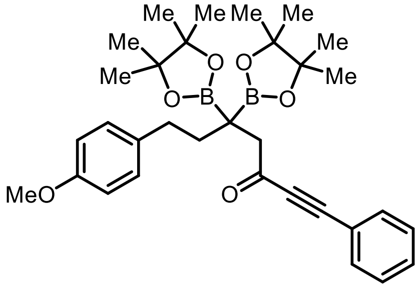

7-(4-methoxyphenyl)-1-phenyl-5,5-bis(4,4,5,5-tetramethyl-1,3,2-dioxaborolan-2-yl)hept-1-yn-3-one (**4m**)

The title compound was prepared according to ***General Procedure A*** with **2b** (52.8, 0.1 mmol) and **3c** (64.9 mg, 0.3 mmol) at 40 °C. The crude reaction mixture was purified by column chromatography (PE:EA = 12:1 to 10:1, stain in CAM) to afford a white solid (34.6 mg, 64%). **^{1}H NMR** ($CDCl_3$, 500 MHz): δ 7.58-7.56 (m, 2H), 7.46-7.42 (m, 1H), 7.39-7.36 (m, 2H), 7.08 (d, *J* = 8.6 Hz, 2H), 6.77 (d, *J* = 8.6 Hz, 2H), 3.74 (s, 3H), 3.19 (s, 2H), 2.49-2.45 (m, 2H), 1.97-1.94 (m, 2H), 1.26 (s, 12H), 1.25 (s, 12H); **^{13}C NMR** ($CDCl_3$, 150 MHz): δ 187.7, 157.5, 135.1, 133.0, 130.4, 129.3, 128.5, 120.2, 113.6, 90.1, 88.0, 83.3, 55.2, 46.9, 33.4, 32.4, 24.8, 24.6; **^{11}B NMR** ($CDCl_3$, 193 MHz): δ 33.8; **HRMS** (ESI-TOF): *m/z* calculated for $C_{32}H_{42}B_2NaO_6^+$ $[M+Na]^+$ 567.3060, found 567.3075.

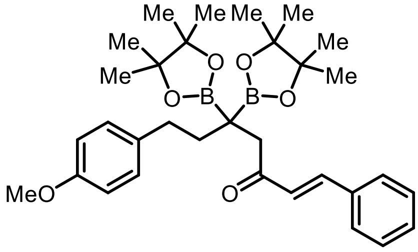

(*E*)-7-(4-methoxyphenyl)-1-phenyl-5,5-bis(4,4,5,5-tetramethyl-1,3,2-dioxaborolan-2-yl)hept-1-en-3-one (4n)

The title compound was prepared according to ***General Procedure A*** with **2b** (52.8 mg, 0.1 mmol) and **3m** (65.5 mg, 0.3 mmol). The crude reaction mixture was purified by column chromatography (PE:EA = 12:1 to 10:1, stain in CAM) to afford a white solid (50.7 mg, 93%).

^{1}H NMR ($CDCl_3$, 600 MHz): δ 7.55-7.52 (m, 3H), 7.39-7.37 (m, 3H), 7.04 (d, *J* = 8.6 Hz, 2H), 6.74-6.69 (m, 3H), 3.70 (s, 3H), 3.14 (s, 2H), 2.46-2.44 (m, 2H), 1.95-1.92 (m, 2H), 1.28 (s, 12H), 1.26 (s, 12H); **^{13}C NMR** ($CDCl_3$, 150 MHz): δ 200.0, 157.4, 142.0, 135.3, 134.7, 130.1, 129.3, 128.8, 128.1, 126.4, 113.5, 83.1, 55.1, 41.9, 33.5, 32.6, 24.9, 24.6; **^{11}B NMR** ($CDCl_3$, 193 MHz): δ 34.2; **HRMS** (ESI-TOF): *m/z* calculated for $C_{32}H_{44}B_2NaO_6^+$ [M+Na]$^+$ 569.3216, found 569.3215.

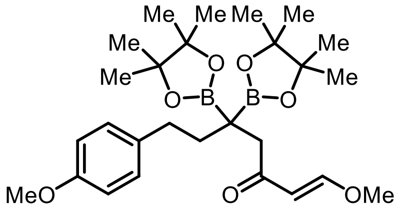

(*E*)-1-methoxy-7-(4-methoxyphenyl)-5,5-bis(4,4,5,5-tetramethyl-1,3,2-dioxaborolan-2-yl)hept-1-en-3-one (4o)

The title compound was prepared according to ***General Procedure A*** with **2b** (52.8 mg, 0.1 mmol), **3n** (51.7 mg, 0.3 mmol) and 1,4-dioxane (1 mL) at 50 °C for 24 hours. The crude reaction mixture was purified by column chromatography (PE:EA = 6:1 to 4:1, stain in CAM) to afford a pale pink solid (21.2 mg, 42%).

^{1}H NMR ($CDCl_3$, 500 MHz): δ 7.59 (d, *J* = 12.8 Hz, 1H), 7.05 (d, *J* = 8.6 Hz, 2H), 6.75 (d, *J* = 8.6 Hz, 2H), 5.56 (d, *J* = 12.8 Hz, 1H), 3.75 (s, 3H), 3.67 (s, 3H), 2.93 (s, 2H), 2.44-2.40 (m, 2H), 1.90-1.86 (m, 2H), 1.26 (s, 12H), 1.24 (s, 12H); **^{13}C NMR** ($CDCl_3$, 125 MHz): δ 199.1, 162.2, 157.5, 135.5, 129.3, 113.5, 105.6, 83.0, 57.2, 55.2, 42.1, 33.5, 32.7, 24.9, 24.7; **^{11}B NMR** ($CDCl_3$, 161 MHz): δ 33.4; **HRMS** (ESI-TOF): *m/z* calculated for $C_{27}H_{42}B_2NaO_7^+$ [M+Na]$^+$ 523.3009, found 523.3028.

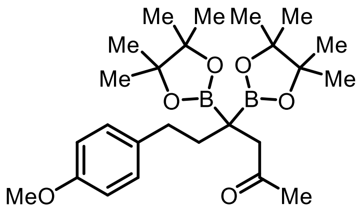

6-(4-methoxyphenyl)-4,4-bis(4,4,5,5-tetramethyl-1,3,2-dioxaborolan-2-yl)hexan-2-one (4p)

The title compound was prepared according to ***General Procedure A*** with **2b** (52.8 mg, 0.1 mmol), **3o** (86.9 mg, 0.6 mmol) and 1,4-dioxane (1 mL) at 40 °C for 24 hours. The crude reaction mixture was purified by column chromatography (PE:EA = 8:1 to 6:1, stain in CAM) to afford a white solid (22.5 mg, 58%).

^{1}H NMR ($CDCl_3$, 500 MHz): δ 7.06 (d, *J* = 8.6 Hz, 2H), 6.77 (d, *J* = 8.5 Hz, 2H), 3.76 (s, 3H), 2.88 (s, 2H), 2.43-2.40 (m, 2H), 2.07 (s, 3H), 1.90-1.87 (m, 2H), 1.25 (s, 12H), 1.23 (s, 12H);

¹³C NMR ($CDCl_3$, 150 MHz): δ 208.6, 157.5, 135.3, 129.3, 113.6, 83.1, 55.3, 45.0, 33.4, 32.6, 29.5, 24.8, 24.6; **¹¹B NMR** ($CDCl_3$, 193 MHz): δ 33.8; **HRMS** (ESI-TOF): *m/z* calculated for $C_{25}H_{40}B_2NaO_6^+$ $[M+Na]^+$ 481.2903, found 481.2917.

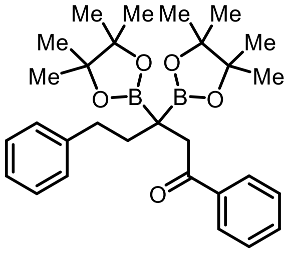

1,5-diphenyl-3,3-bis(4,4,5,5-tetramethyl-1,3,2-dioxaborolan-2-yl)pentan-1-one (**4a**)

The title compound was prepared according to ***General Procedure A*** with **2a** (49.8 mg, 0.1 mmol) and **3a** (57.7 mg, 0.3 mmol). The crude reaction mixture was purified by column chromatography (PE:EA = 15:1, stain in CAM) to afford a white solid (47.4 mg, 97%).

¹H NMR ($CDCl_3$, 500 MHz): δ 8.00-7.98 (m, 2H), 7.55-7.52 (m, 1H), 7.45-7.42 (m, 2H), 7.18-7.15 (m, 2H), 7.10-7.05 (m, 3H), 3.44 (s, 2H), 2.50-2.47 (m, 2H), 2.02-1.98 (m, 2H), 1.28 (s, 12H), 1.26 (s, 12H); **¹³C NMR** ($CDCl_3$, 125 MHz): δ 200.0, 143.1, 137.1, 132.7, 128.4, 128.3, 128.1, 128.0, 125.3, 83.1, 40.3, 34.4, 32.5, 24.8, 24.7; **¹¹B NMR** ($CDCl_3$, 161 MHz): δ 34.0; **HRMS** (ESI-TOF): *m/z* calculated for $C_{29}H_{40}B_2NaO_5^+$ $[M+Na]^+$ 513.2954, found 513.2971.

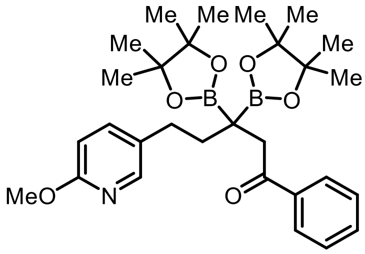

5-(6-methoxypyridin-3-yl)-1-phenyl-3,3-bis(4,4,5,5-tetramethyl-1,3,2-dioxaborolan-2-yl)pentan-1-one (**4q**)

The title compound was prepared according to ***General Procedure A*** with **2c** (52.9 mg, 0.1 mmol) and **3a** (57.7 mg, 0.3 mmol). The crude reaction mixture was purified by column chromatography (PE:EA = 6:1 to 5:1, stain in CAM) to afford a white solid (46.9 mg, 90%).

¹H NMR ($CDCl_3$, 500 MHz): δ 8.00-7.98 (m, 2H), 7.86 (d, J = 2.4 Hz, 1H), 7.55-7.52 (m, 1H), 7.45-7.42 (m, 2H), 7.33 (dd, J = 8.5, 2.4 Hz, 1H), 6.56 (d, J = 8.5 Hz, 1H), 3.85 (s, 3H), 3.44 (s, 2H), 2.42-2.39 (m, 2H), 1.96-1.93 (m, 2H), 1.28 (s, 12H), 1.25 (s, 12H); **¹³C NMR** ($CDCl_3$, 150 MHz): δ 199.8, 162.3, 145.8, 138.9, 137.0, 132.7, 131.0, 128.3, 128.0, 110.1, 83.1, 53.1, 40.2, 32.6, 30.5, 24.8, 24.7; **¹¹B NMR** ($CDCl_3$, 193 MHz): δ 34.0; **HRMS** (ESI-TOF): *m/z* calculated for $C_{29}H_{41}B_2NNaO_6^+$ $[M+Na]^+$ 544.3012, found 544.3028.

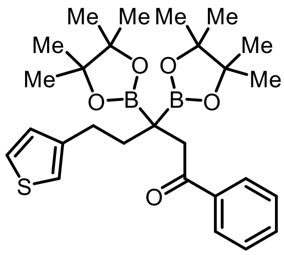

1-phenyl-3,3-bis(4,4,5,5-tetramethyl-1,3,2-dioxaborolan-2-yl)-5-(thiophen-3-yl)pentan-1-one (4r)

The title compound was prepared according to ***General Procedure A*** with **2d** (50.4 mg, 0.1 mmol) and **3a** (57.7 mg, 0.3 mmol). The crude reaction mixture was purified by column chromatography (PE:EA = 15:1, stain in CAM) to afford a white solid (46.1 mg, 93%).

^{1}H NMR ($CDCl_3$, 600 MHz): δ 7.99-7.97 (m, 2H), 7.54-7.51 (m, 1H), 7.44-7.41 (m, 2H), 7.12-7.10 (m, 1H), 6.86-684 (m, 2H), 3.41 (s, 2H), 2.54-2.51 (m, 2H), 2.04-2.01 (m, 2H), 1.28 (s, 12H), 1.25 (s, 12H); **^{13}C NMR** ($CDCl_3$, 150 MHz): δ 199.9, 143.4, 137.1, 132.7, 128.4, 128.3, 128.1, 124.7, 119.7, 83.1, 40.3, 31.3, 28.6, 24.8, 24.76, 24.73, 24.71; **^{11}B NMR** ($CDCl_3$, 193 MHz): δ 34.2; **HRMS** (ESI-TOF): *m/z* calculated for $C_{27}H_{38}B_2NaO_5S^+$ $[M+Na]^+$ 519.2518, found 519.2527.

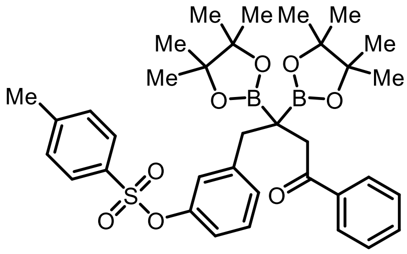

3-(4-oxo-4-phenyl-2,2-bis(4,4,5,5-tetramethyl-1,3,2-dioxaborolan-2-yl)butyl)phenyl 4-methylbenzenesulfonate (4s)

The title compound was prepared according to ***General Procedure A*** with **2e** (65.4 mg, 0.1 mmol) and **3a** (57.7 mg, 0.3 mmol). The crude reaction mixture was purified by column chromatography (PE:EA = 9:1 to 6:1, stain in CAM) to afford a reddish-brown solid (62.3 mg, 96%).

^{1}H NMR ($CDCl_3$, 500 MHz): δ 7.89-7.87 (m, 2H), 7.58 (d, J = 8.4 Hz, 2H), 7.55-7.51 (m, 1H), 7.43-7.40 m, 2H), 7.21 (d, J = 8.1 Hz, 2H), 7.01-6.98 (m, 1H), 6.86-6.84 (m, 1H), 6.76-6.74 (m, 2H), 3.14 (s, 2H), 3.00 (s, 2H), 2.41 (s, 3H), 1.29 (s, 12H), 1.23 (s, 12H); **^{13}C NMR** ($CDCl_3$, 150 MHz): δ 200.4, 149.4, 145.0, 144.1, 136.8, 132.8, 132.5, 129.6, 128.8, 128.5, 128.34, 128.27, 128.1, 123.2, 119.4, 83.4, 39.7, 35.1, 24.9, 24.8, 21.6; **^{11}B NMR** ($CDCl_3$, 193 MHz): δ 33.2; **HRMS** (ESI-TOF): *m/z* calculated for $C_{35}H_{44}B_2NaO_8S^+$ $[M+Na]^+$ 669.2835, found 669.2852.

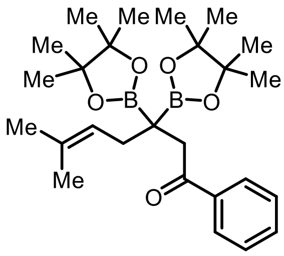

6-methyl-1-phenyl-3,3-bis(4,4,5,5-tetramethyl-1,3,2-dioxaborolan-2-yl)hept-5-en-1-one (4t)

The title compound was prepared according to ***General Procedure A*** with **2f** (46.2 mg, 0.1 mmol) and **3a** (57.7 mg, 0.3 mmol). The crude reaction mixture was purified by column chromatography (PE:EA = 15:1, stain in CAM) to afford a white solid (42.1 mg, 93%).

^{1}H NMR ($CDCl_3$, 600 MHz): δ 7.97-7.95 (m, 2H), 7.52-7.49 (m, 1H), 7.42-7.40 (m, 2H), 5.02 (t, J = 8.3 Hz, 1H), 3.31 (s, 2H), 2.38 (d, J = 7.8 Hz, 2H), 1.58 (s, 3H), 1.30 (s, 3H), 1.28 (s, 12H), 1.25 (s, 12H); **^{13}C NMR** ($CDCl_3$, 150 MHz): δ 200.6, 137.2, 132.7, 132.5, 128.2, 128.1, 123.8, 83.1, 40.2, 28.7, 25.9, 24.7, 17.7; **^{11}B NMR** ($CDCl_3$, 193 MHz): δ 33.7; **HRMS** (ESI-TOF): *m/z* calculated for $C_{26}H_{40}B_2NaO_5^+$ $[M+Na]^+$ 477.2954, found 477.2972.

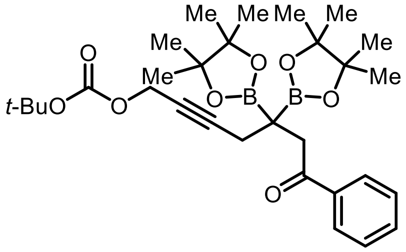

tert-butyl (7-oxo-7-phenyl-5,5-bis(4,4,5,5-tetramethyl-1,3,2-dioxaborolan-2-yl)hept-2-yn-1-yl) carbonate (4u)

The title compound was prepared according to ***General Procedure A*** with **2g** (56.2 mg, 0.1 mmol) and **3a** (57.7 mg, 0.3 mmol). The crude reaction mixture was purified by column chromatography (PE:EA = 12:1 to 9:1, stain in CAM) to afford a white solid (27.5 mg, 50%).

^{1}H NMR ($CDCl_3$, 500 MHz): δ 8.03-8.01 (m, 2H), 7.55-7.51 (m, 1H), 7.45-7.42 (m, 2H), 4.58 (t, J = 2.2 Hz, 2H), 3.53 (s, 2H), 2.59 (t, J = 2.2 Hz, 2H), 1.45 (s, 9H), 1.27 (s, 12H), 1.24 (s, 12H); **^{13}C NMR** ($CDCl_3$, 150 MHz): δ 200.2, 152.9, 136.9, 132.8, 128.31, 128.30, 88.5, 84.5, 83.4, 82.3, 74.0, 55.2, 40.6, 27.7, 24.7, 24.6, 20.4; **^{11}B NMR** ($CDCl_3$, 193 MHz): δ 33.5; **HRMS** (ESI-TOF): *m/z* calculated for $C_{30}H_{44}B_2NaO_8^+$ $[M+Na]^+$ 577.3115, found 577.3130.

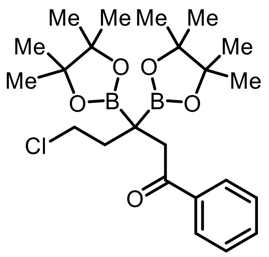

5-chloro-1-phenyl-3,3-bis(4,4,5,5-tetramethyl-1,3,2-dioxaborolan-2-yl)pentan-1-one (4v)

The title compound was prepared according to ***General Procedure A*** with **2h** (45.6 mg, 0.1 mmol) and **3a** (57.7 mg, 0.3 mmol) at 40 °C for 14 hours. The crude reaction mixture was purified by column chromatography (PE:EA = 20:1 to 15:1, irradiation with UV light) to afford a white solid (36.5 mg, 81%).

^{1}H NMR ($CDCl_3$, 500 MHz): δ 8.00-7.98 (m, 2H), 7.56-7.52 (m, 1H), 7.45-7.42 (m, 2H), 3.52 (t, J = 7.5 Hz, 2H), 3.43 (s, 2H), 2.21 (t, J = 7.5 Hz, 2H), 1.27 (s, 12H), 1.24 (s, 12H); **^{13}C NMR** ($CDCl_3$, 150 MHz): δ 199.8, 136.8, 132.9, 128.4, 128.1, 83.3, 44.2, 40.6, 33.7, 24.76, 24.72; **^{11}B NMR** ($CDCl_3$, 193 MHz): δ 33.8; **HRMS** (ESI-TOF): *m/z* calculated for $C_{23}H_{35}B_2ClNaO_5^+$ $[M+Na]^+$ 471.2251, found 471.2260.

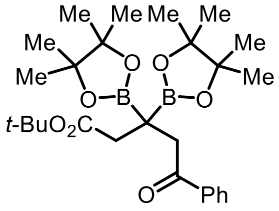

***tert*-butyl 5-oxo-5-phenyl-3,3-bis(4,4,5,5-tetramethyl-1,3,2-dioxaborolan-2-yl)pentanoate** (**4w**)

The title compound was prepared according to ***General Procedure A*** with **2i** (50.8 mg, 0.1 mmol) and **3a** (57.7 mg, 0.3 mmol) at 40 °C for 14 hours. The crude reaction mixture was purified by column chromatography (PE:EA = 15:1 to 13:1, stain in CAM) to afford a white solid (48.1 mg, 96%).

^{1}H NMR ($CDCl_3$, 500 MHz): δ 8.01-7.98 (m, 2H), 7.53-7.50 (m, 1H), 7.43-7.40 (m, 2H), 3.53 (s, 2H), 2.70 (s, 2H), 1.35 (s, 9H), 1.26 (s, 12H), 1.25 (s, 12H); **^{13}C NMR** ($CDCl_3$, 150 MHz): δ 200.3, 174.3, 136.9, 132.7, 128.3, 128.2, 83.2, 79.8, 41.5, 37.1, 28.1, 24.8, 24.6; **^{11}B NMR** ($CDCl_3$, 193 MHz): δ 33.7; **HRMS** (ESI-TOF): *m/z* calculated for $C_{27}H_{42}B_2NaO_7^+$ $[M+Na]^+$ 523.3009, found 523.3023.

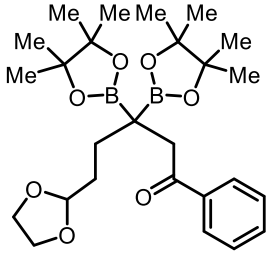

5-(1,3-dioxolan-2-yl)-1-phenyl-3,3-bis(4,4,5,5-tetramethyl-1,3,2-dioxaborolan-2-yl)pentan-1-one (**4x**)

The title compound was prepared according to ***General Procedure A*** with **2j** (49.4 mg, 0.1 mmol) and **3a** (57.7 mg, 0.3 mmol). The crude reaction mixture was purified by column chromatography (PE:EA = 6:1 to 5:1, stain in CAM) to afford a reddish-brown solid (42.5 mg, 87%).

^{1}H NMR ($CDCl_3$, 500 MHz): δ 7.99-7.97 (m, 2H), 7.53-7.50 (m, 1H), 7.43-7.40 (m, 2H), 4.74 (t, J = 4.9 Hz, 1H), 3.89-3.81 (m, 2H), 3.77-3.69 (m, 2H), 3.36 (s, 2H), 1.82-1.78 (m, 2H), 1.59-1.54 (m, 2 H), 1.26 (s, 12H), 1.23 (s, 12H); **^{13}C NMR** ($CDCl_3$, 150 MHz): δ 199.8, 137.1, 132.6,

128.2, 128.0, 105.0, 83.1, 64.6, 40.4, 31.9, 24.76, 24.69; **^{11}B NMR** ($CDCl_3$, 193 MHz): δ 33.8. **HRMS** (ESI-TOF): *m/z* calculated for $C_{26}H_{40}B_2NaO_7^+$ [M+Na]$^+$ 509.2852, found 509.2858.

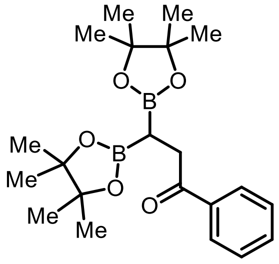

1-phenyl-3,3-bis(4,4,5,5-tetramethyl-1,3,2-dioxaborolan-2-yl)propan-1-one (4y)

The title compound was prepared according to ***General Procedure A*** with **2k** (78.8 mg, 0.2 mmol), **3a** (20.5 μL, 0.1 mmol), and H_2O (4 μL, 0.2 mmol) at 50 °C for 14 hours. The crude reaction mixture was purified by column chromatography (PE:EA = 10:1, stain in CAM) to afford a light yellow solid (22.5 mg, 58%).

^{1}H NMR ($CDCl_3$, 500 MHz): δ 8.00-7.98 (m, 2H), 7.54-7.50 (m, 1H), 7.44-7.41 (m, 2H), 3.30 (d, *J* = 7.9 Hz, 2H), 1.26 (s, 12H), 1.23 (s, 12H), 1.13 (t, *J* = 7.9 Hz, 1H); **^{13}C NMR** ($CDCl_3$, 150 MHz): δ 200.5, 137.0, 132.6, 128.3, 128.1, 83.1, 36.1, 24.8, 24.5; **^{11}B NMR** ($CDCl_3$, 193 MHz): δ 33.8; **HRMS** (ESI-TOF): *m/z* calculated for $C_{21}H_{32}B_2NaO_5^+$ [M+Na]$^+$ 409.2328, found 409.2336.

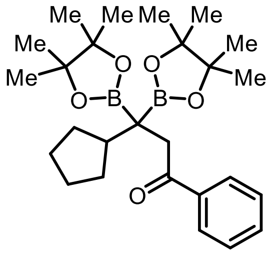

3-cyclopentyl-1-phenyl-3,3-bis(4,4,5,5-tetramethyl-1,3,2-dioxaborolan-2-yl)propan-1-one (4z)

The title compound was prepared according to ***General Procedure A*** with **2l** (46.2 mg, 0.1 mmol) and **3a** (57.7 mg, 0.3 mmol) at 40 °C. The crude reaction mixture was purified by column chromatography (PE:EA = 15:1, stain in CAM) to afford a white solid (34.1 mg, 75%).

^{1}H NMR ($CDCl_3$, 600 MHz): δ 7.99-7.98 (m, 2H), 7.53-7.50 (m, 1H), 7.44-7.41 (m, 2H), 3.32 (s, 2H), 2.21-2.15 (m, 1H), 1.79-1.74 (m, 2H), 1.53-1.42 (m, 4H), 1.25 (s, 12H), 1.24 (s, 12H), 1.18-1.12 (m, 2H); **^{13}C NMR** ($CDCl_3$, 150 MHz): δ 200.9, 137.3, 132.4, 128.2, 128.1, 82.7, 41.9, 40.9, 30.5, 25.1, 24.8, 24.7; **^{11}B NMR** ($CDCl_3$, 193 MHz): δ 33.7; **HRMS** (ESI-TOF): *m/z* calculated for $C_{26}H_{40}B_2NaO_5^+$ [M+Na]$^+$ 477.2954, found 477.2978.

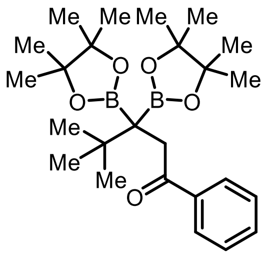

4,4-dimethyl-1-phenyl-3,3-bis(4,4,5,5-tetramethyl-1,3,2-dioxaborolan-2-yl)pentan-1-one (4aa)

The title compound was prepared according to ***General Procedure A*** with **2m** (40.3 mg, 0.1 mmol), **3a** (57.7 mg, 0.3 mmol) and 1,4-dioxane (1 mL) at 50 °C for 24 hours. The crude reaction mixture was purified by column chromatography (PE:EA = 20:1, stain in CAM) to afford a white solid (26.5 mg, 60%).

^{1}H NMR ($CDCl_3$, 500 MHz): δ 7.95-7.93 (m, 2H), 7.52-7.49 (m, 1H), 7.44-7.41 (m, 2H), 3.32 (s, 2H), 1.23 (s, 12H), 1.19 (s, 12H), 1.07 (s, 9H); **^{13}C NMR** ($CDCl_3$, 150 MHz): δ 202.0, 138.1, 132.0, 128.2, 127.8, 82.7, 41.5, 33.1, 29.7, 25.0, 24.6; **^{11}B NMR** ($CDCl_3$, 193 MHz): δ 33.8; **HRMS** (ESI-TOF): *m/z* calculated for $C_{25}H_{40}B_2NaO_5^+$ $[M+Na]^+$ 465.2954, found 465.2972.

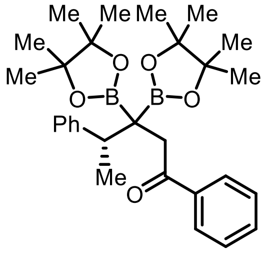

(*S*)-1,4-diphenyl-3,3-bis(4,4,5,5-tetramethyl-1,3,2-dioxaborolan-2-yl)pentan-1-one (4ab)

The title compound was prepared according to ***General Procedure A*** with **2n** (49.8 mg, 0.1 mmol, 92% ee) and **3a** (57.7 mg, 0.3 mmol) at 40 °C. The crude reaction mixture was purified by column chromatography (PE:EA = 15:1, stain in CAM) to afford a white solid (47.7 mg, 97%, 92% ee, >99% es).

^{1}H NMR ($CDCl_3$, 600 MHz): δ 7.78-7.77 (m, 2H), 7.48-7.46 (m, 1H), 7.36-7.34 (m, 2H), 7.14-7.06 (m, 5H), 3.29 (q, J = 7.2 Hz, 1H), 3.20 (d, J = 18.9 Hz, 1H), 3.07 (d, J = 18.9 Hz, 1H), 1.49 (d, J = 7.3 Hz, 3H), 1.30 (s, 6H), 1.29 (s, 6H), 1.27 (s, 6H), 1.21 (s, 6H); **^{13}C NMR** ($CDCl_3$, 150 MHz): δ 201.0, 147.4, 137.2, 132.3, 128.11, 128.10, 128.0, 127.9, 125.7, 83.0, 82.9, 41.8, 41.1, 25.1, 24.9, 24.6, 20.8; **^{11}B NMR** ($CDCl_3$, 193 MHz): δ 33.7; **HRMS** (ESI-TOF): *m/z* calculated for $C_{29}H_{40}B_2NaO_5^+$ $[M+Na]^+$ 513.2945, found 513.2969.

Chiral SFC (OD-3, Chiralcel, 2 mL/min, 5% MeOH, 2000 psi, 35 °C), λ = 220 nm, tr = 4.24 min (major), 8.72 min (minor): 92% ee.

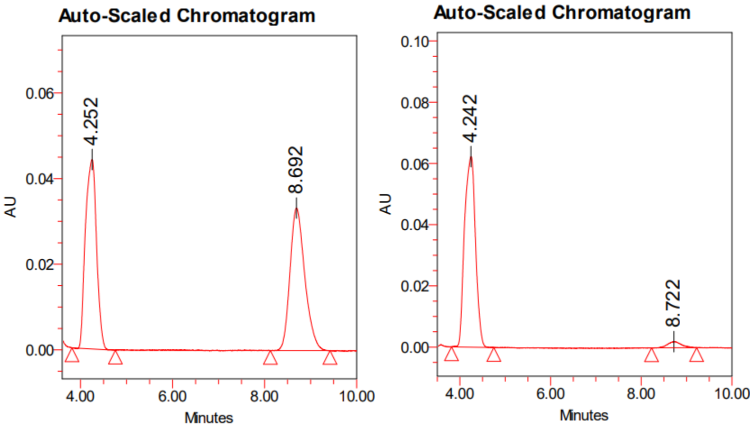

Peak Results

	RT	Area	Height	% Area
1	4.252	740806	44247	49.96
2	*8.692*	*742100*	*33252*	*50.04*

Peak Results

	RT	Area	Height	% Area
1	4.242	1046316	62284	96.04
2	*8.722*	*43131*	*2016*	*3.96*

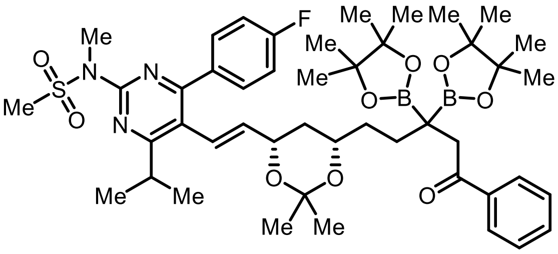

***N*-(5-((*E*)-2-((4*S*,6*S*)-2,2-dimethyl-6-(5-oxo-5-phenyl-3,3-bis(4,4,5,5-tetramethyl-1,3,2-dioxaborolan-2-yl)pentyl)-1,3-dioxan-4-yl)vinyl)-4-(4-fluorophenyl)-6-isopropylpyrimidin-2-yl)-*N*-methylmethanesulfonamide** (**4ac**)

The title compound was prepared according to ***General Procedure A*** with **2o** (88.3 mg, 0.1 mmol) and **3a** (57.7 mg, 0.3 mmol). The crude reaction mixture was purified by column chromatography (PE:EA = 6:1 to 4:1, stain in CAM) to afford a pink solid (64.9 mg, 74%).

^{1}H NMR ($CDCl_3$, 500 MHz): δ 8.00-7.98 (m, 2H), 7.68-7.64 (m, 2H), 7.54-7.51 (m, 1H), 7.44-7.41 (m, 2H), 7.10-7.06 (m, 2H), 6.43 (dd, J = 16.3, 1.4 Hz, 1H), 5.49 (dd, J = 16.2, 5.3 Hz, 1H), 4.34-4.29 (m, 1H), 3.75-3.69 (m, 1H), 3.57 (s, 3H), 3.52 (s, 3H), 3.43-3.35 (m, 3H), 1.77-1.66 (m, 2H), 1.63-1.60 (m, 1H, overlapped with H_2O signal), 1.47-1.40 (m, 1H), 1.37 (s, 3H), 1.31 (s, 3H), 1.27-1.21 (m, 32H), 1.03-0.96 (m, 1H); **^{13}C NMR** ($CDCl_3$, 150 MHz): δ 199.9, 174.8, 163.3, 163.2 (d, J = 250.0 Hz), 157.1, 137.8, 136.9, 134.3 (d, J = 3.2 Hz), 132.7, 132.2 (d, J = 8.3 Hz), 128.3, 128.0, 123.0, 121.2, 114.9 (d, J = 21.6 Hz), 98.5, 83.13, 83.09, 69.5, 69.4, 42.3, 39.8, 36.3, 34.5, 33.0, 31.7, 30.0, 24.8, 24.7, 24.64, 24.60, 21.8, 21.7, 19.7; **^{11}B NMR** ($CDCl_3$, 193 MHz): δ 33.3; **^{19}F NMR** ($CDCl_3$, 565 MHz): δ –111.3; **HRMS** (ESI-TOF): m/z calculated for $C_{46}H_{64}B_2FN_3NaO_9S^+$ $[M+Na]^+$ 898.4426, found 898.4460.

III. Palladium-Catalyzed *gem*-Diborylalkylation of *N*-Vinylacetamide

Optimization of Reaction Conditions

Table S5. Optimization of Solvent with Trimethyl(vinyloxy)silane[a]

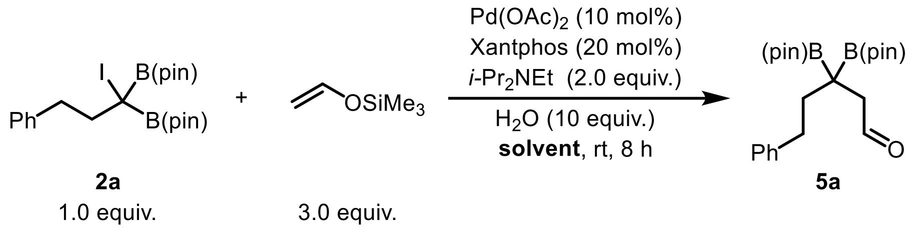

entry	solvent	NMR yield of **5a** (%)
1	DCM	32
2	1,4-dioxane	26
3	THF	16
4	DME	10
5	EA	15
6	MeCN	<2

[a] Conditions: **2a** (0.1 mmol), trimethyl(vinyloxy)silane (0.3 mmol), $Pd(OAc)_2$ (0.01 mmol), Xantphos (0.02 mmol), *i*-Pr_2NEt (0.2 mmol), H_2O (1.0 mmol), **solvent** (1 mL), rt, 8 h. The crude yield was determined by ^1H NMR using 1,3,5-trimethoxybenzene as the internal standard.

Table S6. Optimization of Temperature with Trimethyl(vinyloxy)silane[a]

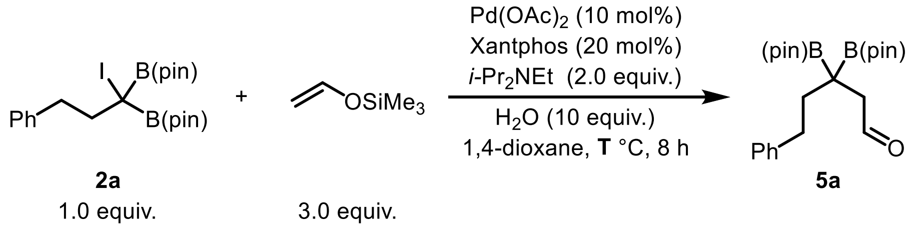

entry	T (°C)	NMR yield of **5a** (%)
1	25	26
2	50	46
3	60	55
4	80	39

[a] Conditions: **2a** (0.1 mmol), trimethyl(vinyloxy)silane (0.3 mmol), $Pd(OAc)_2$ (0.01 mmol), Xantphos (0.02 mmol), *i*-Pr_2NEt (0.2 mmol), H_2O (1.0 mmol), 1,4-dioxane (1 mL), **T** °C, 8 h. The crude yield was determined by ^1H NMR using 1,3,5-trimethoxybenzene as the internal standard.

Table S7. Optimization of Solvent with Vinyl Acetate [a]

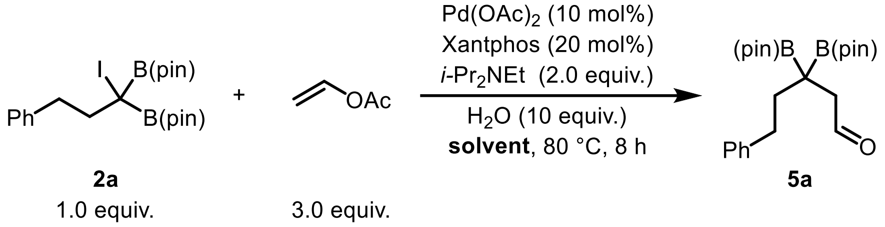

entry	solvent	NMR yield of **5a** (%)
1	DCE	28
2	1,4-dioxane	43
3	THF	28
4	DME	37
5	EA	44
6	MeCN	37

[a] Conditions: **2a** (0.1 mmol), vinyl acetate (0.3 mmol), $Pd(OAc)_2$ (0.01 mmol), Xantphos (0.02 mmol), *i*-Pr_2NEt (0.2 mmol), H_2O (1.0 mmol), **solvent** (1 mL), 80 °C, 8 h. The crude yield was determined by ^1H NMR using 1,3,5-trimethoxybenzene as the internal standard.

Table S8. Optimization of Temperature with *N*-vinylacetamide[a]

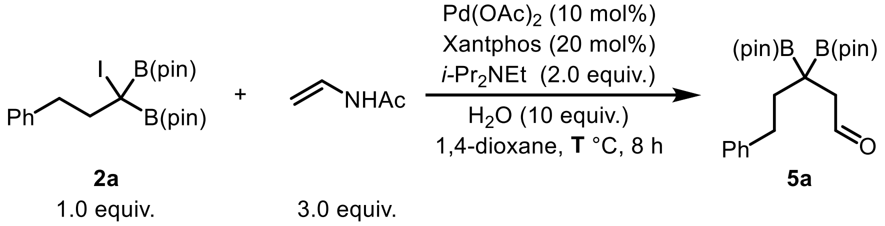

entry	T (°C)	NMR yield of **5a** (%)
1	25	10
2	50	67
3	60	77
4	80	80

[a] Conditions: **2a** (0.1 mmol), *N*-vinylacetamide (0.3 mmol), $Pd(OAc)_2$ (0.01 mmol), Xantphos (0.02 mmol), *i*-Pr_2NEt (0.2 mmol), H_2O (1.0 mmol), 1,4-dioxane (1 mL), **T** °C, 8 h. The crude yield was determined by ^1H NMR using 1,3,5-trimethoxybenzene as the internal standard.

Table S9. Optimization of Solvent with *N*-vinylacetamide[a]

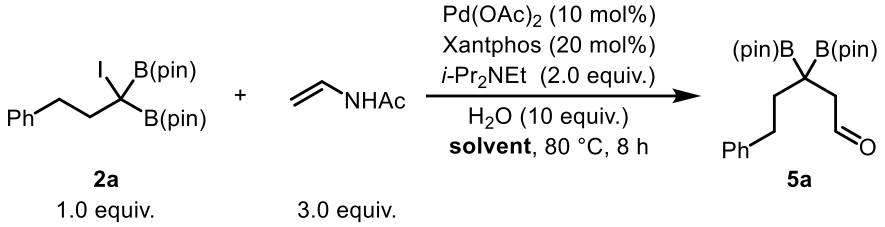

entry	solvent	NMR yield of **5a** (%)
1	DCE	60
2	1,4-dioxane	80
3	THF	59
4	DME	68
5	EA	67
6	MeCN	61

[a] Conditions: **2a** (0.1 mmol), *N*-vinylacetamide (0.3 mmol), $Pd(OAc)_2$ (0.01 mmol), Xantphos (0.02 mmol), *i*-Pr_2NEt (0.2 mmol), H_2O (1.0 mmol), **solvent** (1 mL), 80 °C, 8 h. The crude yield was determined by ^1H NMR using 1,3,5-trimethoxybenzene as the internal standard.

Table S10. Optimization of Palladium Precatalyst and ligand with *N*-vinylacetamide[a]

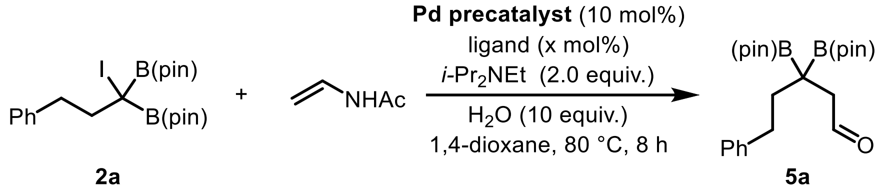

entry	palladium precatalyst	ligand (x mol%)	NMR yield of **5a** (%)
1	$Pd(OAc)_2$	Xantphos (15 mol%)	81
2	$Pd(OAc)_2$	Xantphos (20 mol%)	80
3	$Pd(OAc)_2$	dppf (15 mol%)	78
4	$Pd(dppf)Cl_2$	/	77
**5**	**$Pd(dppf)Cl_2$**	**Xantphos (15 mol%)**	**89**
6	$Pd(dppf)Cl_2$	Xantphos (20 mol%)	80
7	$Pd(P\textit{t}\text{-}Bu_3)_2$	Xantphos (15 mol%)	82
8	$Pd(P\textit{t}\text{-}Bu_3)_2$	Xantphos (20 mol%)	80
9	$Pd(dba)_2$	Xantphos (20 mol%)	72

[a] Conditions: **2a** (0.1 mmol), *N*-vinylacetamide (0.3 mmol), **palladium precatalyst** (0.01 mmol), ligand, *i*-Pr_2NEt (0.2 mmol), H_2O (1.0 mmol), 1,4-dioxane (1 mL), 80 °C, 8 h. The crude yield was determined by ^1H NMR using 1,3,5-trimethoxybenzene as the internal standard.

Experimental Procedures and Spectral Data

General Procedure B

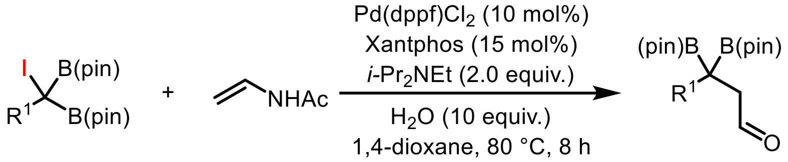

In the glove box, an oven-dried 10 mL vial with a magnetic stir bar was charged with Pd(dppf)Cl_2 (7.3 mg, 0.01 mmol), Xantphos (8.7 mg, 0.015 mmol) and anhydrous 1,4-dioxane (1 mL). The reaction vial was sealed and allowed to stir at ambient temperature for 10 minutes. Then, *i*-Pr_2NEt (35 µL, 0.2 mmol), *N*-vinylacetamide (25.5 mg, 0.3 mmol), and iododiboron substrate (0.1 mmol) were successively added. The vial was sealed with a polypropylene open-top cap with PTFE/silicone septum, removed from the glove box, charged with H_2O (18 µL, 1 mmol), and stirred at 80 °C for 8 hours. Upon completion, the reaction mixture was cooled to room temperature, diluted with ethyl acetate, filtered through a silica gel plug using ethyl acetate as eluent, and concentrated *in vacuo*. The crude reaction mixture was purified by column chromatography on *pre-dried silica gel* using petroleum ether/ethyl acetate as the eluent to afford the desired product.

***Note**: Flash column chromatography on pre-dried silica gel should generally be performed rapidly and preferably completed within 20 minutes; otherwise, product decomposition may occur, leading to lower yields.*

Spectral Data:

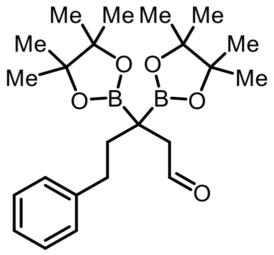

5-phenyl-3,3-bis(4,4,5,5-tetramethyl-1,3,2-dioxaborolan-2-yl)pentanal (5a)
The title compound was prepared according to ***General Procedure B*** with **2a** (49.8 mg, 0.1 mmol). The crude reaction mixture was purified by column chromatography on *pre-dried silica gel* (PE:EA = 10:1 to 9:1, stain in CAM) to afford a white solid (30.4 mg, 73%).
^{1}H NMR ($CDCl_3$, 500 MHz): δ 9.75 (t, *J* = 1.2 Hz, 1H), 7.24-7.21 (m, 2H), 7.16-7.12 (m, 3H), 2.84 (d, *J* = 1.2 Hz, 2H), 2.51-2.48 (m, 2H), 1.95-1.92 (m, 2H), 1.25 (s, 12H), 1.24 (s, 12H); **^{13}C NMR** ($CDCl_3$, 150 MHz): δ 203.0, 142.9, 128.4, 128.1, 125.5, 83.4, 45.3, 34.1, 33.0, 24.7, 24.6; **^{11}B NMR** ($CDCl_3$, 193 MHz): δ 33.8; **HRMS** (ESI-TOF): *m/z* calculated for $C_{23}H_{37}B_2O_5^+$ $[M+H]^+$ 415.2822, found 415.2833.

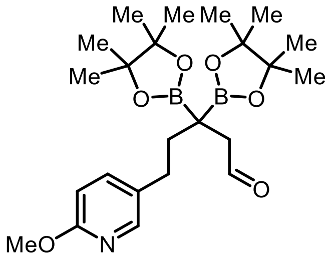

5-(6-methoxypyridin-3-yl)-3,3-bis(4,4,5,5-tetramethyl-1,3,2-dioxaborolan-2-yl)pentanal (5b)

The title compound was prepared according to ***General Procedure B*** with **2c** (52.9 mg, 0.1 mmol). The crude reaction mixture was purified by column chromatography on *pre-dried silica gel* (PE:EA = 4:1 to 2:1, stain in CAM) to afford a pale yellow solid (31.0 mg, 70%).

^{1}H NMR ($CDCl_3$, 500 MHz): δ 9.76 (t, J = 1.2 Hz, 1H), 7.92 (d, J = 2.4 Hz, 1H), 7.39 (dd, J = 8.4, 2.5 Hz, 1H), 6.63 (d, J = 8.4 Hz, 1H), 3.89 (s, 3H), 2.85 (s, 2H), 2.43-2.39 (m, 2H), 1.89-1.86 (m, 2H), 1.25 (s, 12H), 1.23 (s, 12H); **^{13}C NMR** ($CDCl_3$, 125 MHz): δ 202.8, 162.5, 145.9, 139.0, 130.8, 110.2, 83.5, 53.2, 45.3, 33.0, 30.3, 24.7, 24.6; **^{11}B NMR** ($CDCl_3$, 193 MHz): δ 33.9; **HRMS** (ESI-TOF): *m/z* calculated for $C_{23}H_{37}B_2NNaO_6^+$ $[M+Na]^+$ 468.2699, found 468.2706.

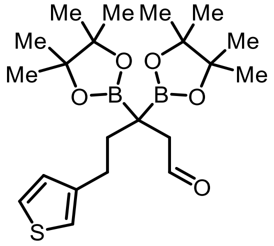

3,3-bis(4,4,5,5-tetramethyl-1,3,2-dioxaborolan-2-yl)-5-(thiophen-3-yl)pentanal (5c)

The title compound was prepared according to ***General Procedure B*** with **2d** (50.4 mg, 0.1 mmol). The crude reaction mixture was purified by column chromatography on *pre-dried silica gel* (PE:EA = 10:1 to 9:1, stain in CAM) to afford a white solid (21.2 mg, 50%).

^{1}H NMR ($CDCl_3$, 600 MHz): δ 9.73 (t, J = 1.2 Hz, 1H), 7.19 (dd, J = 4.9, 3.0 Hz, 1H), 6.93-6.90 (m, 2H), 2.82 (d, J = 1.1 Hz, 2H), 2.55-2.52 (m, 2H), 1.97-1.94 (m, 2H), 1.24 (s, 12H), 1.23 (s, 12H); **^{13}C NMR** ($CDCl_3$, 150 MHz): δ 202.9, 143.1, 128.4, 124.9, 119.9, 83.4, 45.4, 31.7, 28.4, 24.73, 24.65; **^{11}B NMR** ($CDCl_3$, 193 MHz): δ 34.1; **HRMS** (ESI-TOF): *m/z* calculated for $C_{21}H_{34}B_2NaO_5S^+$ $[M+Na]^+$ 443.2205, found 443.2212.

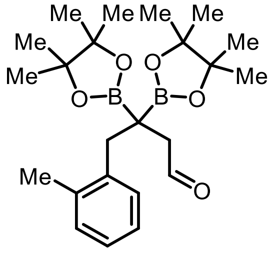

3,3-bis(4,4,5,5-tetramethyl-1,3,2-dioxaborolan-2-yl)-4-(o-tolyl)butanal (5d)

The title compound was prepared according to ***General Procedure B*** with **2p** (49.8 mg, 0.1 mmol). The crude reaction mixture was purified by column chromatography on *pre-dried silica gel* (PE:EA = 18:1 to 15:1, stain in CAM) to afford a colorless oil (27.2 mg, 68%).

^{1}H NMR ($CDCl_3$, 500 MHz): δ 9.59 (t, J = 1.2 Hz, 1H), 7.27-7.25 (m, 1H, overlapped with $CHCl_3$ signal), 7.08-7.02 (m, 3H), 3.05 (s, 2H), 2.75 (s, 2H), 2.19 (s, 3H), 1.27 (s, 12H), 1.23 (s, 12H); **^{13}C NMR** ($CDCl_3$, 150 MHz): δ 202.9, 139.7, 137.0, 130.2, 129.2, 125.7, 125.4, 83.6, 45.7, 31.8, 24.9, 24.7, 20.1; **^{11}B NMR** ($CDCl_3$, 193 MHz): δ 33.7; **HRMS** (ESI-TOF): *m/z* calculated for $C_{23}H_{36}B_2NaO_5^+$ $[M+Na]^+$ 437.2641, found 437.2640.

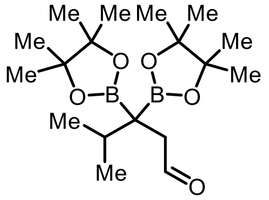

4-methyl-3,3-bis(4,4,5,5-tetramethyl-1,3,2-dioxaborolan-2-yl)pentanal (5e)

The title compound was prepared according to ***General Procedure B*** with **2q** (43.6 mg, 0.1 mmol). The crude reaction mixture was purified by column chromatography on *pre-dried silica gel* (PE:EA = 10:1, stain in CAM) to afford a white solid (24.0 mg, 68%).

^{1}H NMR ($CDCl_3$, 500 MHz): δ 9.78 (t, J = 1.8 Hz, 1H), 2.59 (d, J = 1.8 Hz, 2H), 2.29-2.20 (m, 1H), 1.23 (s, 24H), 0.90 (d, J = 6.9 Hz, 6H); **^{13}C NMR** ($CDCl_3$, 150 MHz): δ 204.9, 83.3, 42.5, 29.8, 24.73, 24.67, 21.1; **^{11}B NMR** ($CDCl_3$, 193 MHz): δ 33.7; **HRMS** (ESI-TOF): *m/z* calculated for $C_{18}H_{34}B_2NaO_5^+$ $[M+Na]^+$ 375.2485, found 375.2490.

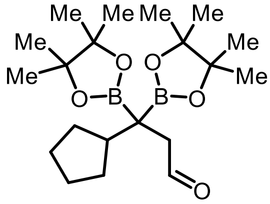

3-cyclopentyl-3,3-bis(4,4,5,5-tetramethyl-1,3,2-dioxaborolan-2-yl)propanal (5f)

The title compound was prepared according to ***General Procedure B*** with **2l** (46.2 mg, 0.1 mmol). The crude reaction mixture was purified by column chromatography on *pre-dried silica gel* (PE:EA = 15:1 to 12:1, stain in CAM) to afford a white solid (27.6 mg, 73%).

^{1}H NMR ($CDCl_3$, 500 MHz): δ 9.76 (t, J = 1.1 Hz, 1H), 2.63 (d, J = 1.6 Hz, 2H), 2.23-2.16 (m, 1H), 1.74-1.68 (m, 2H), 1.57-1.43 (m, 4H), 1.27-1.13 (m, 26H); **^{13}C NMR** ($CDCl_3$, 150 MHz): δ 204.4, 83.2, 44.6, 42.0, 30.1, 25.3, 24.66, 24.65; **^{11}B NMR** ($CDCl_3$, 193 MHz): δ 33.6; **HRMS** (ESI-TOF): *m/z* calculated for $C_{20}H_{36}B_2NaO_5^+$ $[M+Na]^+$ 401.2641, found 401.2653.

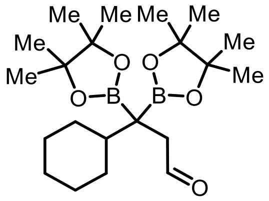

3-cyclohexyl-3,3-bis(4,4,5,5-tetramethyl-1,3,2-dioxaborolan-2-yl)propanal (5g)

The title compound was prepared according to ***General Procedure B*** with **2r** (47.6 mg, 0.1 mmol). The crude reaction mixture was purified by column chromatography on *pre-dried silica gel* (PE:EA = 25:1 to 20:1, stain in CAM) to afford a white solid (30.4 mg, 78%).

^{1}H NMR ($CDCl_3$, 500 MHz): δ 9.76 (t, *J* = 1.2 Hz, 1H), 2.59 (d, *J* = 1.9 Hz, 2H), 1.91-1.85 (m, 1H), 1.70-1.57 (m, 4H, overlapped with H_2O signal), 1.36-1.23 (m, 27H), 1.10-0.99 (m, 3H). **^{13}C NMR** ($CDCl_3$, 150 MHz): δ 205.1, 83.4, 42.8, 40.5, 31.6, 27.0, 26.3, 24.75, 24.67; **^{11}B NMR** ($CDCl_3$, 193 MHz): δ 33.7; **HRMS** (ESI-TOF): *m/z* calculated for $C_{21}H_{38}B_2NaO_5^+$ $[M+Na]^+$ 415.2798, found 415.2807.

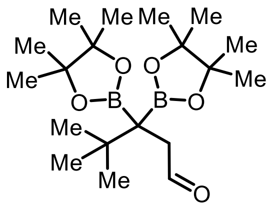

4,4-dimethyl-3,3-bis(4,4,5,5-tetramethyl-1,3,2-dioxaborolan-2-yl)pentanal (5h)

The title compound was prepared according to ***General Procedure B*** with **2m** (40.3 mg, 0.1 mmol) at 100 °C . The crude reaction mixture was purified by column chromatography on *pre-dried silica gel* (PE:EA = 25:1 to 20:1, stain in CAM) to afford a white solid (10.5 mg, 29%).

^{1}H NMR ($CDCl_3$, 500 MHz): δ 9.74 (t, *J* = 1.2 Hz, 1H), 2.68 (d, *J* = 1.5 Hz, 2H), 1.22 (s, 12H), 1.21 (s, 12H), 1.03 (s, 9H); **^{13}C NMR** ($CDCl_3$, 125 MHz): δ 204.4, 83.1, 45.6, 33.3, 29.4, 24.9, 24.6; **^{11}B NMR** ($CDCl_3$, 193 MHz): δ 33.6; **HRMS** (ESI-TOF): *m/z* calculated for $C_{19}H_{36}B_2NaO_5^+$ $[M+Na]^+$ 389.2641, found 389.2648.

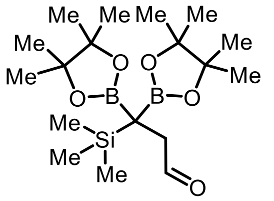

3,3-bis(4,4,5,5-tetramethyl-1,3,2-dioxaborolan-2-yl)-3-(trimethylsilyl)propanal (5i)

The title compound was prepared according to ***General Procedure B*** with **2s** (46.6 mg, 0.1 mmol). The crude reaction mixture was purified by column chromatography on *pre-dried silica gel* (PE:EA = 25:1 to 20:1, stain in CAM) to afford a white solid (20.8 mg, 54%).

^{1}H NMR ($CDCl_3$, 500 MHz): δ 9.66 (t, *J* = 1.2 Hz, 1H), 2.61 (s, 2H), 1.20 (s, 12H), 1.18 (s, 12H), 0.10 (s, 9H); **^{13}C NMR** ($CDCl_3$, 150 MHz): δ 203.3, 82.9, 42.9, 24.7, 24.6, –1.6; **^{11}B NMR** ($CDCl_3$, 193 MHz): δ 33.5; **HRMS** (ESI-TOF): *m/z* calculated for $C_{18}H_{36}B_2NaO_5Si^+$ $[M+Na]^+$ 405.2410, found 405.2424.

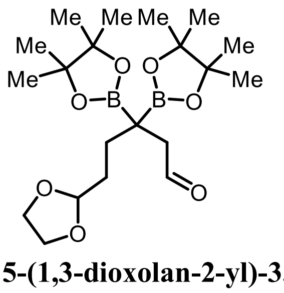

5-(1,3-dioxolan-2-yl)-3,3-bis(4,4,5,5-tetramethyl-1,3,2-dioxaborolan-2-yl)pentanal (5j)

The title compound was prepared according to ***General Procedure B*** with **2j** (49.4 mg, 0.1 mmol). The crude reaction mixture was purified by column chromatography on ***pre-dried silica gel*** (PE:EA = 4:1 to 3:1, stain in CAM) to afford a reddish-brown solid (30.2 mg, 74%).
^{1}H NMR ($CDCl_3$, 500 MHz): δ 9.71 (t, *J* = 1.1 Hz, 1H), 4.79 (t, *J* = 4.9 Hz, 1H), 3.95-3.89 (m, 2H), 3.84-3.77 (m, 2H), 2.76 (d, *J* = 1.0 Hz, 2H), 1.77-1.73 (m, 2H), 1.59-1.55 (m, 2H), 1.23 (s, 12H), 1.21 (s, 12H); **^{13}C NMR** ($CDCl_3$, 150 MHz): δ 202.7, 104.8, 83.3, 64.7, 45.4, 31.7, 25.0, 24.67, 24.61; **^{11}B NMR** ($CDCl_3$, 193 MHz): δ 33.7; **HRMS** (ESI-TOF): *m/z* calculated for $C_{20}H_{36}B_2NaO_7^+$ $[M+Na]^+$ 433.2539, found 433.2548.

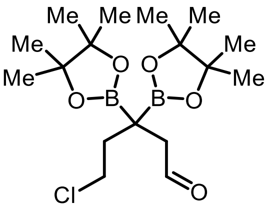

5-chloro-3,3-bis(4,4,5,5-tetramethyl-1,3,2-dioxaborolan-2-yl)pentanal (5k)
The title compound was prepared according to ***General Procedure B*** with **2h** (45.6 mg, 0.1 mmol). The crude reaction mixture was purified by column chromatography on *pre-dried silica gel* (PE:EA = 12:1, stain in CAM) to afford a white solid (17.0 mg, 46%).
^{1}H NMR ($CDCl_3$, 500 MHz): δ 9.73 (t, *J* = 1.2 Hz, 1H), 3.52 (t, *J* = 7.6 Hz, 2H), 2.87 (s, 2H), 2.14 (t, *J* = 7.6 Hz, 2H), 1.24 (s, 12H), 1.22 (s, 12H); **^{13}C NMR** ($CDCl_3$, 150 MHz): δ 202.2, 83.6, 45.7, 43.7, 33.7, 24.69, 24.65; **^{11}B NMR** ($CDCl_3$, 193 MHz): δ 33.5; **HRMS** (ESI-TOF): *m/z* calculated for $C_{17}H_{31}B_2ClNaO_5^+$ $[M+Na]^+$ 395.1938, found 395.1949.

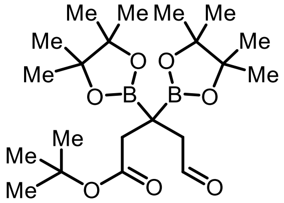

***tert*-butyl 5-oxo-3,3-bis(4,4,5,5-tetramethyl-1,3,2-dioxaborolan-2-yl)pentanoate (5l)**
The title compound was prepared according to ***General Procedure B*** with **2i** (50.8 mg, 0.1 mmol). The crude reaction mixture was purified by column chromatography on *pre-dried silica gel* (PE:EA = 10:1 to 9:1, stain in CAM) to afford a colorless oil (30.2 mg, 71%).
^{1}H NMR ($CDCl_3$, 500 MHz): δ 9.70 (t, *J* = 1.2 Hz, 1H), 2.98 (s, 2H), 2.64 (s, 2H), 1.39 (s, 9H), 1.23 (s, 24H); **^{13}C NMR** ($CDCl_3$, 150 MHz): δ 202.9, 173.7, 83.5, 80.1, 46.6, 36.9, 28.1, 24.7, 24.6; **^{11}B NMR** ($CDCl_3$, 193 MHz): δ 33.6; **HRMS** (ESI-TOF): *m/z* calculated for $C_{21}H_{38}B_2N_aO_7^+$ $[M+Na]^+$ 447.2696, found 447.2711.

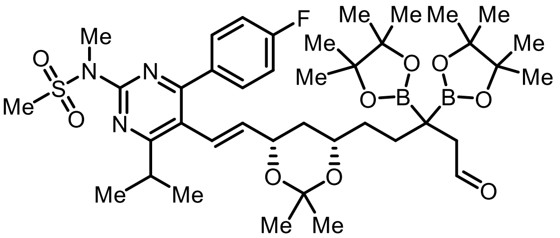

***N*-(5-((*E*)-2-((4*S*,6*S*)-2,2-dimethyl-6-(5-oxo-3,3-bis(4,4,5,5-tetramethyl-1,3,2-dioxaborolan-2-yl)pentyl)-1,3-dioxan-4-yl)vinyl)-4-(4-fluorophenyl)-6-isopropylpyrimidin-2-yl)-*N*-methylmethanesulfonamide (5m)**

The title compound was prepared according to ***General Procedure B*** with **2o** (88.3 mg, 0.1 mmol). The crude reaction mixture was purified by column chromatography on *pre-dried silica gel* (PE:EA = 5:1 to 3:1, stain in CAM) to afford a reddish brown oil (46.3 mg, 56%).

^{1}H NMR ($CDCl_3$, 600 MHz): δ 9.72 (t, *J* = 1.2 Hz, 1H), 7.68-7.65 (m, 2H), 7.12-7.08 (m, 2H), 6.47 (dd, *J* = 16.3, 1.4 Hz, 1H), 5.50 (dd, *J* = 16.3, 5.4 Hz, 1H), 4.37-4.33 (m, 1H), 3.77-3.72 (m, 1H), 3.57 (s, 3H), 3.52 (s, 3H), 3.43-3.38 (m, 1H), 2.81 (s, 2H), 1.67-1.63 (m, 2H), 1.59-1.56 (m, 1H), 1.46-1.37 (m, 7H), 1.28-1.22 (m, 31H), 1.05-0.99 (m, 1H); **^{13}C NMR** ($CDCl_3$, 150 MHz): δ 202.8, 174.8, 163.4, 163.3 (d, *J* = 249.9 Hz), 157.2, 137.8, 134.3 (d, *J* = 3.1 Hz), 132.2 (d, *J* = 8.3 Hz), 123.0, 121.2, 114.9 (d, *J* = 21.6 Hz), 98.6, 83.41, 83.39, 69.5, 69.3, 45.0, 42.4, 36.3, 34.3, 33.0, 31.8, 30.1, 24.75, 24.66, 24.59, 24.56, 21.8, 21.7, 19.8; **^{19}F NMR** ($CDCl_3$, 565 MHz) δ –111.5; **^{11}B NMR** ($CDCl_3$, 193 MHz): δ 33.6; **HRMS** (ESI-TOF): *m/z* calculated for $C_{40}H_{60}B_2FN_3NaO_9S^+$ $[M+Na]^+$ 822.4113, found 824.4122.

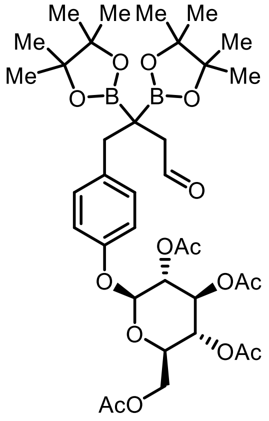

(2*R*, 3*R*, 4*S*, 5*R*, 6*S*)-2-(acetoxymethyl)-6-(4-(4-oxo-2,2-bis(4,4,5,5-tetramethyl-1,3,2-dioxaborolan-2-yl)butyl)phenoxy)tetrahydro-2*H*-pyran-3,4,5-triyl triacetate (5n)

The title compound was prepared according to ***General Procedure B*** with **2t** (83.0 mg, 0.1 mmol). The crude reaction mixture was purified by column chromatography on *pre-dried silica gel* (PE:EA = 3:1 to 2:1, stain in CAM) to afford a pale reddish brown solid (51.8 mg, 69%).

^{1}H NMR ($CDCl_3$, 500 MHz): δ 9.66 (t, *J* = 1.2 Hz, 1H), 7.04 (d, *J* = 8.6 Hz, 2H), 6.83 (d, *J* = 8.6 Hz, 2H), 5.30-5.23 (m, 2H), 5.17-5.14 (m, 1H), 5.01 (d, *J* = 7.4 Hz, 1H), 4.28 (dd, *J* = 12.3, 5.3 Hz, 1H), 4.16 (dd, *J* = 12.3, 2.5 Hz, 1H), 3.85-3.82 (m, 1H), 2.99 (s, 2H), 2.64 (s, 2H), 2.07 (s, 3H), 2.05 (s, 3H), 2.04 (s, 3H), 2.03 (s, 3H), 1.27 (s, 12H), 1.22 (s, 12H); **^{13}C NMR** ($CDCl_3$, 150 MHz): δ 202.9, 170.5, 170.2, 169.3, 169.2, 155.1, 136.2, 130.6, 116.5, 99.2, 83.5, 72.7, 71.8, 71.1, 68.2, 61.9, 44.8, 34.6, 24.82, 24.80, 24.64, 20.59, 20.55, 20.52, 20.50; **^{11}B NMR** ($CDCl_3$, 193 MHz): δ 34.2; **HRMS** (ESI-TOF): *m/z* calculated for $C_{36}H_{52}B_2NaO_{15}^+$ $[M+Na]^+$ 769.3385, found 769.3405.

IV. Synthetic Utilization

Gram-scale Experiments

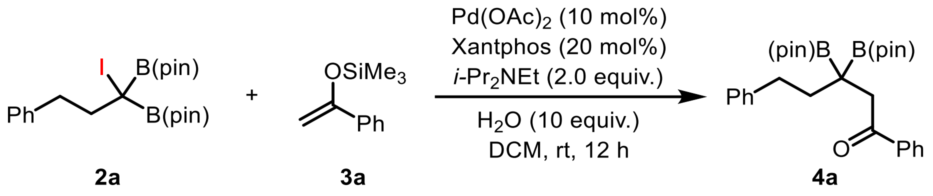

In the glove box, an oven-dried 100 mL round-bottom flask with a magnetic stir bar was charged with $Pd(OAc)_2$ (67.4 mg, 0. 3 mmol), Xantphos (347.2 mg, 0.6 mmol) and anhydrous DCM (30 mL). The reaction flask was sealed and allowed to stir at ambient temperature for 10 minutes. Then, *i*-Pr_2NEt (1.0 mL, 6.0 mmol), **3a** (1.8 mL, 9.0 mmol) and **2a** (1.5 g, 3.0 mmol) were successively added. The flask was sealed, removed from the glove box, charged with H_2O (540 μL, 30 mmol) and stirred at rt for 12 hours. Upon completion, the reaction mixture was cooled to room temperature, diluted with ethyl acetate, filtered through a silica gel plug using ethyl acetate as eluent, and concentrated *in vacuo*. The crude reaction mixture was purified by column chromatography on silica gel using petroleum ether/ethyl acetate as the eluent to afford a white solid (1.37 g, 93%).

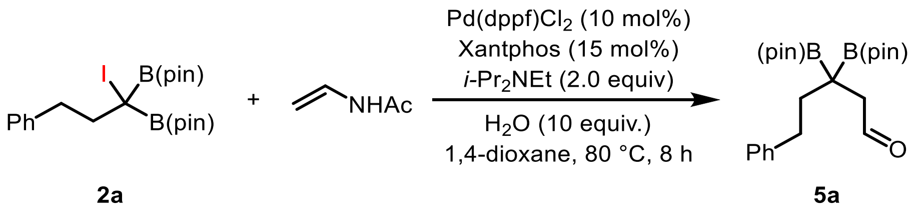

In the glove box, an oven-dried 100 mL round-bottom flask with a magnetic stir bar was charged with $Pd(dppf)Cl_2$ (292.7 mg, 0.4 mmol), Xantphos (347.2 mg, 0.6 mmol) and anhydrous 1,4-dioxane (40 mL). The reaction flask was sealed and allowed to stir at ambient temperature for 10 minutes. Then, *i*-Pr_2NEt (1.4 mL, 8.0 mmol), *N*-vinylacetamide (1.0 g, 12.0 mmol), and **2a** (2.0 g, 4.0 mmol) were successively added. The flask was sealed, removed from the glove box, charged with H_2O (720 μL, 40 mmol), and stirred at 80 °C for 8 hours. Upon completion, the reaction mixture was cooled to room temperature, diluted with ethyl acetate, filtered through a silica gel plug using ethyl acetate as eluent, and concentrated *in vacuo*. The crude reaction mixture was purified by column chromatography on silica gel using petroleum ether/ethyl acetate as the eluent to afford a white solid (1.19 g, 72%).

Diversification of Products

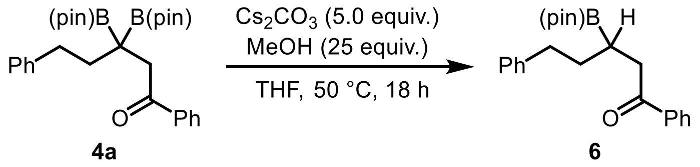

The reaction was performed according to the literature procedure with slight modifications.[7] An oven-dried 10 mL vial with a magnetic stir bar was charged with **4a** (49 mg, 0.1 mmol) and Cs_2CO_3 (163 mg, 0.5 mmol). The vial was sealed with a polypropylene open-top cap with PTFE/silicone septum, evacuated and backfilled with argon three times, then anhydrous THF (1 mL) and anhydrous MeOH (0.1 mL) were added, and the mixture was stirred at 50 °C for 18 hours. Next, the reaction was quenched with H_2O and extracted with EA (3 × 5 mL). The organic layers were dried over Na_2SO_4, filtered, and concentrated *in vacuo*. The crude reaction mixture was purified by column chromatography (PE/EA = 18:1, stain in CAM) to afford a colorless oil (33.4 mg, 92%).

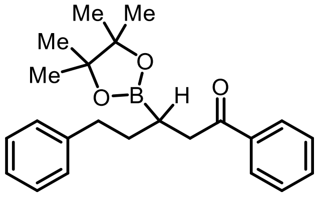

1,5-diphenyl-3-(4,4,5,5-tetramethyl-1,3,2-dioxaborolan-2-yl)pentan-1-one (6)
^{1}H NMR (500 MHz, $CDCl_3$): δ 7.96-7.94 (m, 2H), 7.55-7.52 (m, 1H), 7.45-7.42 (m, 2H), 7.28-7.25 (m, 2H, overlapped with $CHCl_3$ signal), 7.20-7.15 (m, 3H), 3.21-3.12 (m, 2H), 2.74-2.65 (m, 2H), 1.91-1.83 (m, 1H), 1.74-1.66 (m, 1H), 1.50-1.44 (m, 1H), 1.28 (s, 6H), 1.25 (s, 6H); **^{13}C NMR** (150 MHz, $CDCl_3$): δ 200.2, 142.6, 137.0, 132.8, 128.41, 128.37, 128.2, 128.0, 125.6, 83.0, 41.0, 35.4, 32.6, 24.8, 24.7; **^{11}B NMR** (193 MHz, $CDCl_3$) δ 33.9; **HRMS** (ESI-TOF): *m/z* calculated for $C_{23}H_{29}BNaO_3^+$ $[M+Na]^+$ 387.2102, found 387.2112.

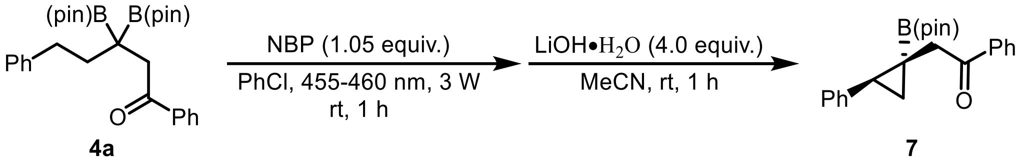

The reaction was performed according to the literature procedure with slight modifications.[8] An oven-dried 10 mL vial with a magnetic stir bar was charged with **4a** (98.1 mg, 0.2 mmol) and 2-bromoisoindoline-1,3-dione (47.5 mg, 0.21 mmol). The vial was sealed with a polypropylene open-top cap with PTFE/silicone septum, evacuated and backfilled with argon three times, then chlorobenzene (2 mL) was added. The reaction mixture was then irradiated with a 3 W 455-460 nm LED lamp with cooling from circulating cold water at 25 °C for 1 hour. Upon completion, the reaction mixture was concentrated *in vacuo*. $LiOH\cdot H_2O$ (33.6 mg, 0.8 mmol) was added to the vial and then purged with argon three times. MeCN (2 mL) was then added to the mixture as solvent. The reaction mixture was stirred at room temperature for 12 hours. Upon completion, the reaction mixture was filtered through a silica gel plug using EA as eluent. After evaporation under reduced pressure, the crude reaction mixture was purified by column chromatography (PE:EA = 20:1, stain in CAM) to afford a light yellow solid (52.0 mg, 72%, > 50:1 dr).

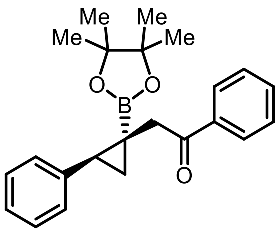

1-phenyl-2-((1*R*,2*R*)-2-phenyl-1-(4,4,5,5-tetramethyl-1,3,2-dioxaborolan-2-yl)cyclopropyl)ethan-1-one (7)

^{1}H NMR (600 MHz, $CDCl_3$): δ 7.69-7.68 (m, 2H), 7.46-7.43 (m, 1H), 7.33-7.31 (m, 2H), 7.21-7.19 (m, 4H), 7.13-7.10 (m, 1H), 2.78 (d, J = 18.4 Hz, 1H), 2.65 (d, J = 18.4 Hz, 1H), 2.51 (dd, J = 8.0, 6.1 Hz, 1H), 1.34-1.32 (m, 1H), 1.26 (s, 12H), 1.00-0.98 (m, 1H); **^{13}C NMR** (150 MHz, $CDCl_3$): δ 199.8, 138.6, 137.3, 132.4, 129.5, 128.2, 127.9, 127.7, 126.0, 83.3, 40.4, 25.7, 24.54, 24.50, 14.4; **^{11}B NMR** (193 MHz, $CDCl_3$) δ 33.0; **HRMS** (ESI-TOF): *m/z* calculated for $C_{23}H_{27}BNaO_3^+$ $[M+Na]^+$ 385.1945, found 385.1956.

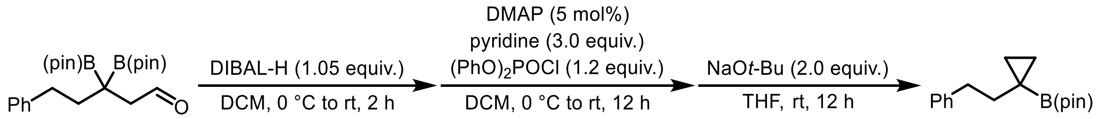

5a **8**

STEP I: The reaction was performed according to the literature procedure with slight modifications.[9] To a solution of **5a** (165.7 mg, 0.4 mmol) in CH_2Cl_2 (4 mL) at 0 °C was added dropwise DIBAL-H (1.0 M in THF, 0.42 mL, 0.42 mmol). The reaction mixture was warmed to room temperature and stirred for 2 hours. Upon completion, the reaction mixture was cooled to 0 °C before being quenched by the addition of MeOH (1.5 mL) followed by sodium potassium tartrate (2.0 mL). The mixture was diluted with Et_2O, the phases were separated, and the aqueous phase was extracted with Et_2O (3 × 10 mL). The combined organic extracts were washed with brine, dried over Na_2SO_4, filtered, and concentrated *in vacuo*. The obtained crude mixture was used for the next step without purification.

STEP II: The reaction was performed according to the literature procedure with slight modifications.[10] An oven-dried 20 mL vial with a magnetic stir bar was charged with the crude mixture and anhydrous CH_2Cl_2 (4.0 mL) under argon atmosphere. To this solution, pyridine (0.1 mL, 1.2 mmol) and 4-(dimethylamino)pyridine (2.4 mg, 0.02 mmol) were sequentially added. The mixture was cooled at 0 °C, and diphenyl chlorophosphate (0.1 mL, 0.48 mmol) was added dropwise. The reaction mixture was warmed to room temperature and stirred for 12 hours. The reaction mixture was cooled to 0 °C and quenched with H_2O (2 mL). The aqueous layer was extracted with EA (3 × 10 mL). The combined organic layers were dried over Na_2SO_4, filtered, and concentrated under reduced pressure. The crude mixture was purified by column chromatography on silica gel (PE:EA = 5:1 to 4:1, stain in CAM) to give the desired product as a colorless oil (240.8 mg, 93%).

STEP III: An oven-dried 20 mL vial with a magnetic stir bar was charged with the product from Step II (240.8 mg, 0.37 mmol) and NaO*t*-Bu (71.1 mg, 0.74 mmol), then purged with argon three times. THF (4 mL) was added to the mixture as solvent. The reaction mixture was stirred at room temperature for 12 hours. Upon completion, the reaction mixture was passed through a silica gel plug using EA as eluent. After evaporation under reduced pressure, the

crude reaction mixture was purified by column chromatography on *pre-dried silica gel* (PE:EA = 15:1, stain in CAM) to afford a colorless oil (86.1 mg, 79% over 3 steps).

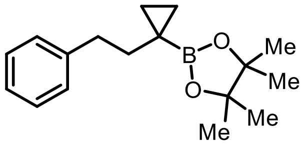

4,4,5,5-tetramethyl-2-(1-phenethylcyclopropyl)-1,3,2-dioxaborolane (8)
^{1}H NMR (600 MHz, $CDCl_3$): δ 7.26-7.24 (m, 2H, overlapped with $CHCl_3$ signal), 7.19-7.13 (m, 3H), 2.73-2.71 (m, 2H), 1.52-1.50 (m, 2H), 1.22 (s, 12H), 0.67-0.65 (m, 2H), 0.29-0.28 (m, 2H); **^{13}C NMR** (150 MHz, $CDCl_3$): δ 143.2, 128.4, 128.1, 125.4, 82.9, 38.7, 35.8, 24.7, 11.5; **^{11}B NMR** (193 MHz, $CDCl_3$) δ 33.9; **HRMS** (ESI-TOF): *m/z* calculated for $C_{17}H_{25}BNaO_2^+$ $[M+Na]^+$ 295.1840, found 295.1846.

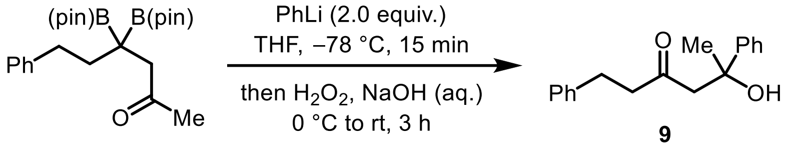

9

An oven-dried 10 mL vial with a magnetic stir bar was charged with 6-phenyl-4,4-bis(4,4,5,5-tetramethyl-1,3,2-dioxaborolan-2-yl)hexan-2-one (95.6 mg, 0.2 mmol) and anhydrous THF (2.0 mL) under argon, then cooled to –78 °C. PhLi (1.0 M in Et_2O, 0.4 mL, 0.4 mmol) was added to this solution dropwise with stirring for 15 minutes. The reaction mixture was warmed to 0 °C in an ice-cold water bath, and then 3 M NaOH (2.0 mL) was added, followed by 30% H_2O_2 (1.0 mL) dropwise. The reaction mixture was allowed to warm up to room temperature and was stirred for 3 hours, then cooled back to 0 °C, and saturated aq. $Na_2S_2O_3$ (3.0 mL) was added dropwise. The reaction was warmed to room temperature and the aqueous layer was extracted with EA (3 × 10 mL). The combined organic layers were dried over Na_2SO_4, filtered and concentrated *in vacuo*. After evaporation under reduced pressure, the crude reaction mixture was purified by column chromatography on silica gel (PE:EA = 9:1 to 8:1) to afford a colorless oil (44.2 mg, 82%).

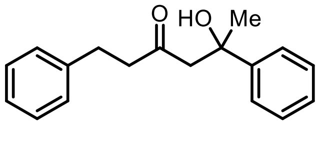

5-hydroxy-1,5-diphenylhexan-3-one (9)
^{1}H NMR (500 MHz, $CDCl_3$): δ 7.40-7.38 (m, 2H), 7.34-7.30 (m, 2H), 7.26-7.16 (m, 4H, overlapped with $CHCl_3$ signal), 7.07-7.04 (m, 2H), 4.52 (s, 1H), 3.14 (d, J = 16.7 Hz, 1H), 2.82-2.54 (m, 5H), 1.50 (s, 3H); **^{13}C NMR** (150 MHz, $CDCl_3$): δ 211.7, 147.1, 140.4, 128.4, 128.2, 128.1, 126.7, 126.1, 124.3, 73.3, 53.5, 45.9, 30.6, 29.0; **HRMS** (ESI-TOF): *m/z* calculated for $C_{18}H_{20}NaO_2^+$ $[M+Na]^+$ 291.1356, found 291.1366.

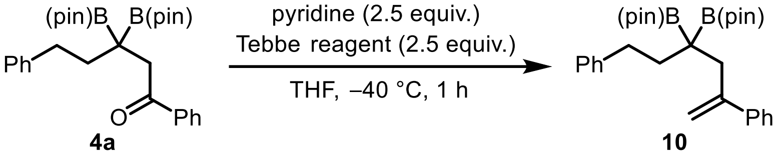

The reaction was performed according to the literature procedure with slight modifications.[11] A solution of **4a** (50.6 mg, 0.1 mmol) and pyridine (20 μL, 0.25 mmol) in THF (1.2 mL) was cooled to −40 °C and treated with Tebbe reagent (0.5 M in toluene, 0.5 mL, 0.25 mmol). After being stirred at −40 °C for 1 hour, the reaction mixture was diluted with Et_2O (2 mL), 15% aqueous NaOH and solid Na_2SO_4 was added to this mixture. The resulting mixture was stirred at room temperature for 1.5 hours and filtered through Celite. Concentration and flash column chromatography (PE:EA = 30:1 to 25:1, stain in CAM) provided **10** as a white solid (46.5 mg, 92%)

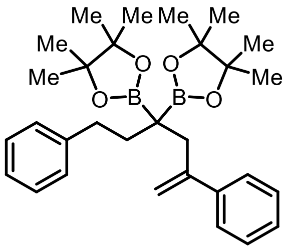

2,2'-(1,5-diphenylhex-5-ene-3,3-diyl)bis(4,4,5,5-tetramethyl-1,3,2-dioxaborolane) (10)
^{1}H NMR (500 MHz, $CDCl_3$): δ 7.44-7.41 (m, 2H), 7.31-7.22 (m, 3H, overlapped with $CHCl_3$ signal), 7.17-7.14 (m, 2H), 7.09-7.06 (m, 1H), 6.92-6.90 (m, 2H), 5.23 (d, J = 1.6 Hz, 1H), 5.16 (d, J = 1.8 Hz, 1H), 2.97 (s, 2H), 2.43-2.40 (m, 2H), 1.87-1.84 (m, 2H), 1.21 (s, 12H), 1.18 (s, 12H); **^{13}C NMR** (150 MHz, $CDCl_3$): δ 149.2, 143.9, 143.5, 128.4, 128.0, 127.9, 126.97, 126.95, 125.2, 114.9, 83.2, 34.1, 33.9, 32.1, 25.1, 24.6; **^{11}B NMR** (193 MHz, $CDCl_3$) δ 33.9; **HRMS** (ESI-TOF): m/z calculated for $C_{30}H_{42}B_2NaO_4^+$ $[M+Na]^+$ 511.3161, found 511.3166.

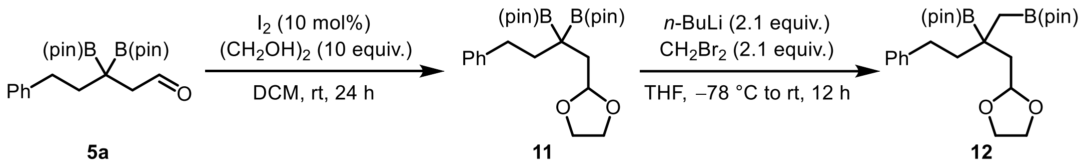

STEP I: The reaction was performed according to literature with slight modifications.[12] An oven-dried 20 mL vial with a magnetic stir bar was charged with **5a** (124.3 mg, 0.3 mmol, 1.0 equiv.), iodine (7.6 mg, 0.03 mmol), ethylene glycol (186.2 mg, 3.0 mmol) and anhydrous DCM (1.5 mL), and was allowed to stir at room temperature for 24 hours. After TLC analysis showed that starting material was completely consumed, diethyl ether (10 mL) was added. The reaction mixture was washed with $Na_2S_2O_3$ (5 mL), washed with saturated $NaHCO_3$ (5 mL), dried over Na_2SO_4 and the solvent gets evaporated. Finally, the crude mixture was purified by column chromatography on silica gel (PE:EA = 10:1 to 9:1, stain in CAM) to give the desired product **11** as a white solid (129.9 mg, 95%).
STEP II: An oven-dried 10 mL vial with a magnetic stir bar was charged with **11** (458 mg, 1.0 mmol, 1.0 equiv.), CH_2Br_2 (147 μL, 2.1 mmol) and THF (10 mL) under argon. The mixture was cooled to –78 °C, and n-BuLi (1.6 M in n-hexane, 1.25 mL, 2.1 mmol) was added to this

solution carefully and stirred for 30 minutes. The reaction mixture was then warmed to room temperature and stirred for 12 hours. Upon completion, the crude mixture was diluted with Et_2O, washed with brine, dried over Na_2SO_4, filtered, and concentrated *in vacuo*. After evaporation under reduced pressure, the crude reaction mixture was purified by column chromatography on silica gel (PE:EA:DCM = 12:1:1 to 10:1:1) to afford a white solid (436.9 mg, 93%).

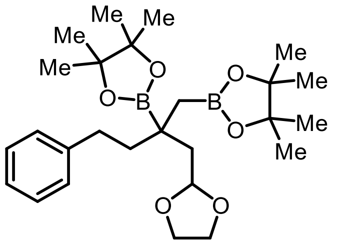

2,2'-(2-((1,3-dioxolan-2-yl)methyl)-4-phenylbutane-1,2-diyl)bis(4,4,5,5-tetramethyl-1,3,2-dioxaborolane) (12)

^{1}H NMR (500 MHz, $CDCl_3$): δ 7.26-7.12 (m, 5H, overlapped with $CHCl_3$ signal), 5.03 (t, J = 5.1 Hz, 1H), 3.97-3.90 (m, 2H), 3.84-3.77 (m, 2H), 2.64-2.53 (m, 2H), 1.89 (d, J = 5.1 Hz, 2H), 1.85-1.79 (m, 1H), 1.76-1.70 (m, 1H), 1.26 (s, 12H), 1.23 (s, 12H), 1.09-1.00 (m, 2H); **^{13}C NMR** (125 MHz, $CDCl_3$): δ 143.9, 128.5, 128.1, 125.3, 103.4, 83.0, 82.7, 64.5, 64.4, 41.1, 40.3, 31.5, 25.0, 24.9, 24.88, 24.86; **^{11}B NMR** (193 MHz, $CDCl_3$) δ 33.6; **HRMS** (ESI-TOF): *m/z* calculated for $C_{26}H_{43}B_2O_6^+$ $[M+H]^+$ 473.3240, found 473.3247.

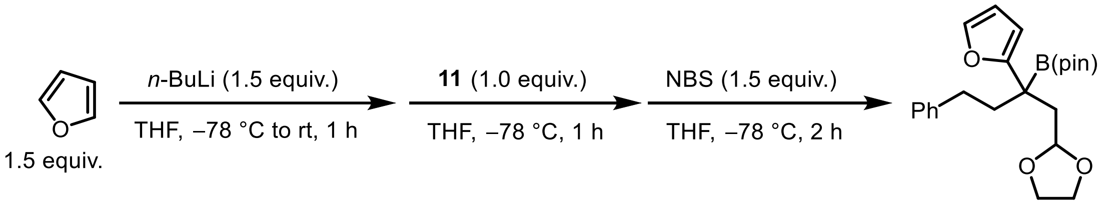

13

The reaction was performed according to the literature procedure with slight modifications.[13] An oven-dried 10 mL vial with a magnetic stir bar was charged with furan (22 μL,0.3 mmol) and anhydrous THF (0.8 mL) under argon, then cooled to –78 °C. *n*-BuLi (1.6 M in *n*-hexane, 0.19 mL, 0.3 mmol) was added to this solution dropwise. The reaction mixture was warmed to room temperature and stirred for 1 hour, then cooled to –78 °C. A solution of **11** (91.6 mg, 0.2 mmol) in THF (0.4 mL) was added and stirred for 1 hour, then a solution of NBS (53.4 mg, 0.3 mmol) in THF (0.8 mL) was added to the mixture and stirred for 2 hours under –78 °C. Upon completion, the reaction was quenched with sat. $Na_2S_2O_3$ solution (2 mL) and the reaction mixture was allowed to warm to room temperature. The crude mixture was diluted with Et_2O, washed with brine, dried over Na_2SO_4, filtered, and concentrated *in vacuo*. After evaporation under reduced pressure, the crude reaction mixture was purified by column chromatography on silica gel (PE:EA:DCM = 15:1:1 to 12:1;1) to afford a white solid (36.1 mg, 45%).

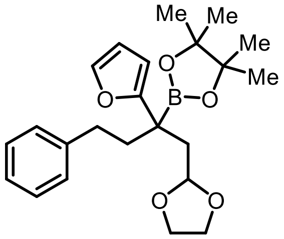

2-(1-(1,3-dioxolan-2-yl)-2-(furan-2-yl)-4-phenylbutan-2-yl)-4,4,5,5-tetramethyl-1,3,2-dioxaborolane (13)

^{1}H NMR (500 MHz, $CDCl_3$): δ 7.35 (d, *J* = 1.8 Hz, 1H), 7.25-7.22 (m, 2H), 7.15-7.11 (m, 3H), 6.35 (d, *J* = 3.2 Hz, 1H), 6.29 (dd, *J* = 3.2, 1.8 Hz, 1H), 4.81 (t, *J* = 5.1 Hz, 1H), 3.97-3.87 (m, 2H), 3.81-3.76 (m, 2H), 2.44-2.33 (m, 2H), 2.24 (dd, *J* = 13.8, 4.7 Hz, 1H), 2.18-2.03 (m, 3H), 1.29 (s, 6H), 1.27 (s, 6H); **^{13}C NMR** (150 MHz, $CDCl_3$): δ 157.9, 143.0, 140.7, 128.4, 128.2, 125.5, 109.8, 106.7, 103.1, 83.6, 64.7, 64.5, 39.0, 38.2, 32.1, 24.9, 24.8; **^{11}B NMR** (193 MHz, $CDCl_3$) δ 33.0; **HRMS** (ESI-TOF): *m/z* calculated for $C_{23}H_{31}BNaO_5^+$ $[M+Na]^+$ 421.2157, found 421.2164.

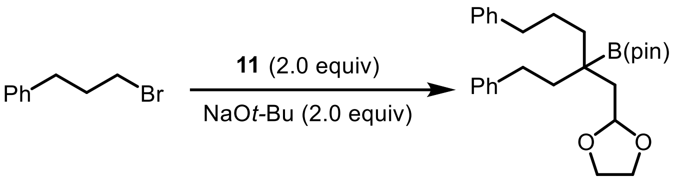

14

The reaction was performed according to literature with slight modifications.[3] In a glove box, an oven-dried 10 mL vial with a magnetic stir bar was charged with the product **11** from Step I (91.6 mg, 0.2 mmol), (3-bromopropyl)benzene (19.9 mg, 0.1 mmol) and anhydrous THF (1.0 mL), followed by NaO*t*-Bu (28.8 mg, 0.3 mmol). The vial was sealed with a polypropylene open-top cap with PTFE/silicone septum, removed from the glove box, and allowed to stir at room temperature for 12 hours. Upon completion, the reaction mixture was diluted with diethyl ether, filtered through a silica gel plug, rinsed with diethyl ether, and concentrated *in vacuo*. The crude mixture was purified by column chromatography on silica gel (PE:EA = 11:1 to 10:1, stain in CAM) to give the desired product **14** as a colorless oil (44.0 mg, 98%).

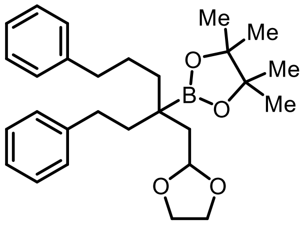

2-(3-((1,3-dioxolan-2-yl)methyl)-1,6-diphenylhexan-3-yl)-4,4,5,5-tetramethyl-1,3,2-dioxaborolane (14)

^{1}H NMR (500 MHz, $CDCl_3$): δ 7.29-7.13 (m, 10H, overlapped with $CHCl_3$ signal), 4.97 (t, *J* = 5.1 Hz, 1H), 3.97-3.90 (m, 2H), 3.84-3.77 (m, 2H), 2.65-2.44 (m, 4H), 1.80 (d, *J* = 5.1 Hz, 2H), 1.76-1.51 (m, 6H, overlapped with H_2O signal), 1.24 (s, 12H); **^{13}C NMR** (150 MHz, $CDCl_3$): δ 143.5, 142.8, 128.4, 128.19, 128.17, 125.6, 125.4, 103.2, 83.1, 64.5, 38.8, 36.7, 36.6, 33.9,

30.8, 26.4, 25.0; **^{11}B NMR** (193 MHz, $CDCl_3$) δ 34.1; **HRMS** (ESI-TOF): *m/z* calculated for $C_{28}H_{39}BNaO_4^+$ $[M+Na]^+$ 473.2834, found 473.2844.

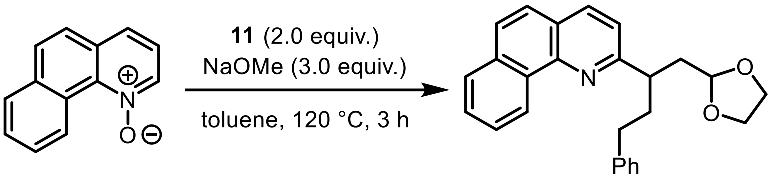

15

The reaction was performed according to the literature procedure with slight modifications.[15] In a glove box, an oven-dried 10 mL vial with a magnetic stir bar was charged with quinoline-*N*-oxide (19.5 mg, 0.1 mmol), NaOMe (16.2 mg, 0.3 mmol), **11** (91.6 mg, 0.2 mmol) and anhydrous toluene (1.0 mL). The vial was sealed with a cap, removed from the glove box, and allowed to stir at 120 °C for 3 hours. The reaction mixture was filtered through celite and washed with DCM (10 mL). The filtrate was concentrated under reduced pressure. The crude reaction mixture was purified by column chromatography on silica gel (PE:EA:DCM = 15:1:1 to 12:1:1) to afford a white solid (35.4 mg, 92%).

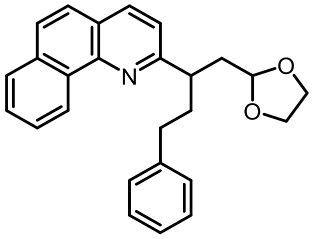

2-(1-(1,3-dioxolan-2-yl)-4-phenylbutan-2-yl)benzo[*h*]quinoline (15)

^{1}H NMR (500 MHz, $CDCl_3$): δ 9.40 (dd, *J* = 8.1, 1.5 Hz, 1H), 8.08 (d, *J* = 8.2 Hz, 1H), 7.89 (dd, *J* = 7.7, 1.5 Hz, 1H), 7.77-7.66 (m, 4H), 7.39 (d, *J* = 8.1 Hz, 1H), 7.26-7.21 (m, 2H, overlapped with $CHCl_3$ signal), 7.15-7.10 (m, 3H), 4.72 (dd, *J* = 6.7, 3.5 Hz, 1H), 3.97-3.87 (m, 2H), 3.78-3.70 (m, 2H), 3.34-3.29 (m, 1H), 2.62-2.47 (m, 3H), 2.39-2.31 (m, 1H), 2.19-2.08 (m, 2H); **^{13}C NMR** (150 MHz, $CDCl_3$): δ 162.7, 146.1, 142.4, 135.7, 133.6, 131.6, 128.4, 128.2, 127.9, 127.6, 126.8, 126.7, 125.6, 125.2, 124.7, 124.6, 122.2, 103.3, 64.71, 64.68, 43.6, 39.6, 38.1, 33.6; **HRMS** (ESI-TOF): *m/z* calculated for $C_{26}H_{26}NO_2^+$ $[M+H]^+$ 384.1958, found 384.1969.

V. Mechanistic Studies

Radical-clock Experiment

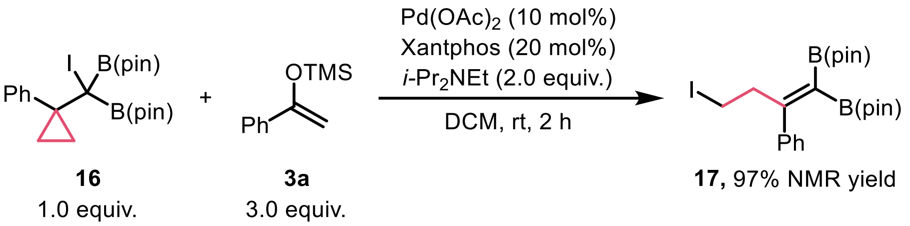

In the glove box, an oven-dried 10 mL vial with a magnetic stir bar was charged with $Pd(OAc)_2$ (2.2 mg, 0.01 mmol), Xantphos (11.6 mg, 0.02 mmol) and anhydrous DCM (1 mL). The reaction vial was sealed and allowed to stir at ambient temperature for 10 minutes. Then, *i*-Pr_2NEt (35 μL, 0.2 mmol), **3a** (62 μL, 0.3 mmol) and **16** (51.0 mg, 0.1 mmol) were successively added. The vial was sealed with a cap, removed from the glove box, and stirred for 2 hours at room temperature. Upon completion, the reaction mixture was cooled to room temperature, diluted with Et_2O, filtered through a silica gel plug using Et_2O as eluent, and concentrated *in vacuo* to give the mixture, which was tested by ^1H NMR using 1,3,5-trimethoxybenzene (7.4 mg, 0.044 mmol) as internal standard. The ^1H NMR spectrum of the mixture is shown below.

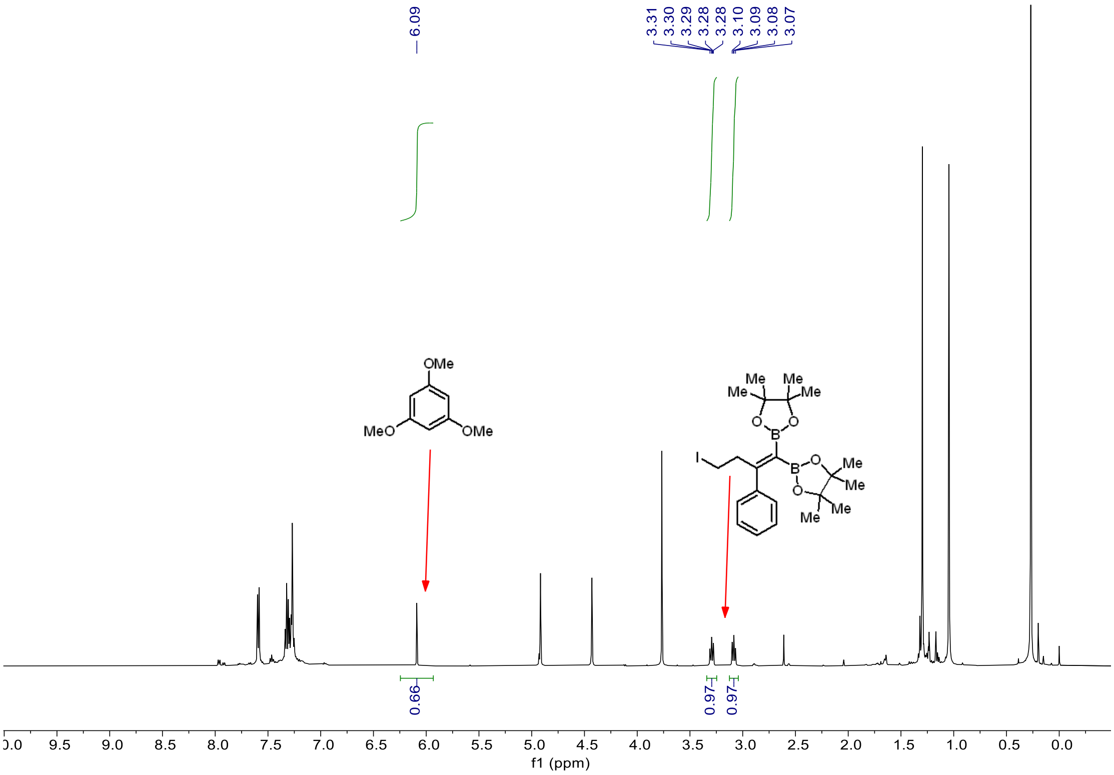

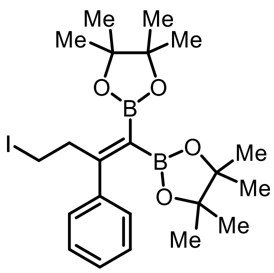

2,2'-(4-iodo-2-phenylbut-1-ene-1,1-diyl)bis(4,4,5,5-tetramethyl-1,3,2-dioxaborolane) (17)
^{1}H NMR (600 MHz, $CDCl_3$) δ 7.28-7.25 (m, 5H), 3.30-3.28 (m, 2H), 3.10-3.07 (m, 2H), 1.30 (s, 12H), 1.04 (s, 12H).
The spectral data are in accord with the literature.[2]

Effect of Boryl Groups

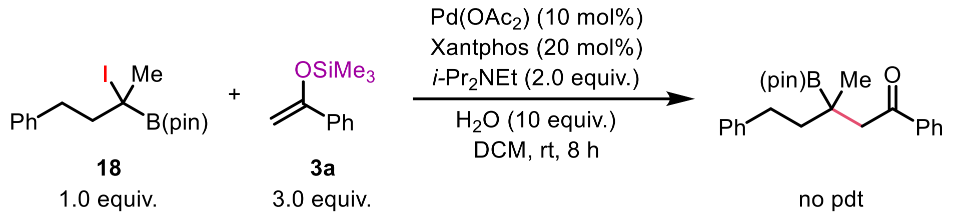

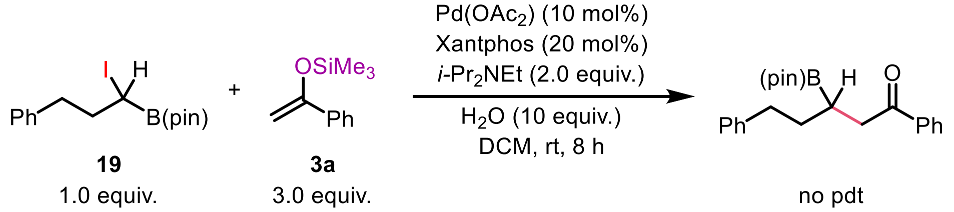

The reaction followed ***General Procedure A*** with **18** (38.6 mg, 0.1 mmol) or **19** (37.2 mg, 0.1 mmol). The amounts of the product were determined by ^{1}H NMR of the crude mixture.

Isotope-Labeling Experiments

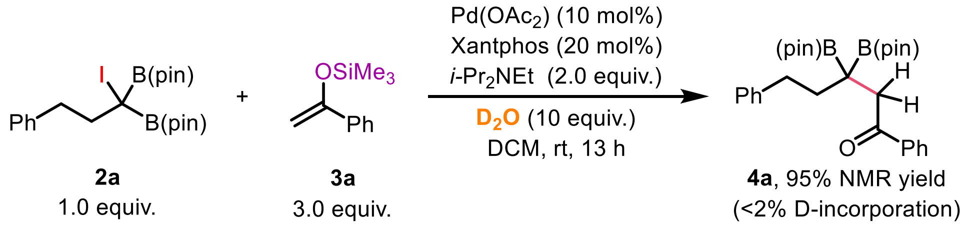

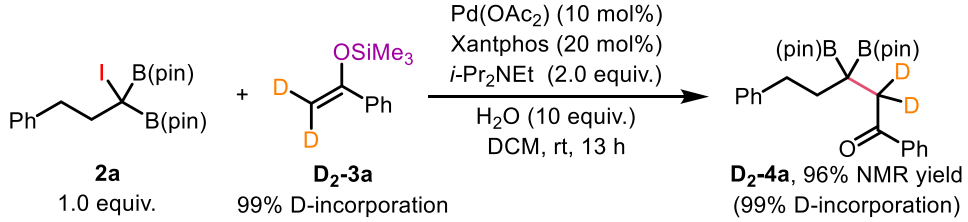

The reaction followed ***General Procedure A*** with D_2O (18 µL, 1.0 mmol) or **D$_2$-3a** (58.3 mg, 0.3 mmol). The amounts of the product and the remaining starting material were determined by

^{1}H NMR of the crude mixture (see below).

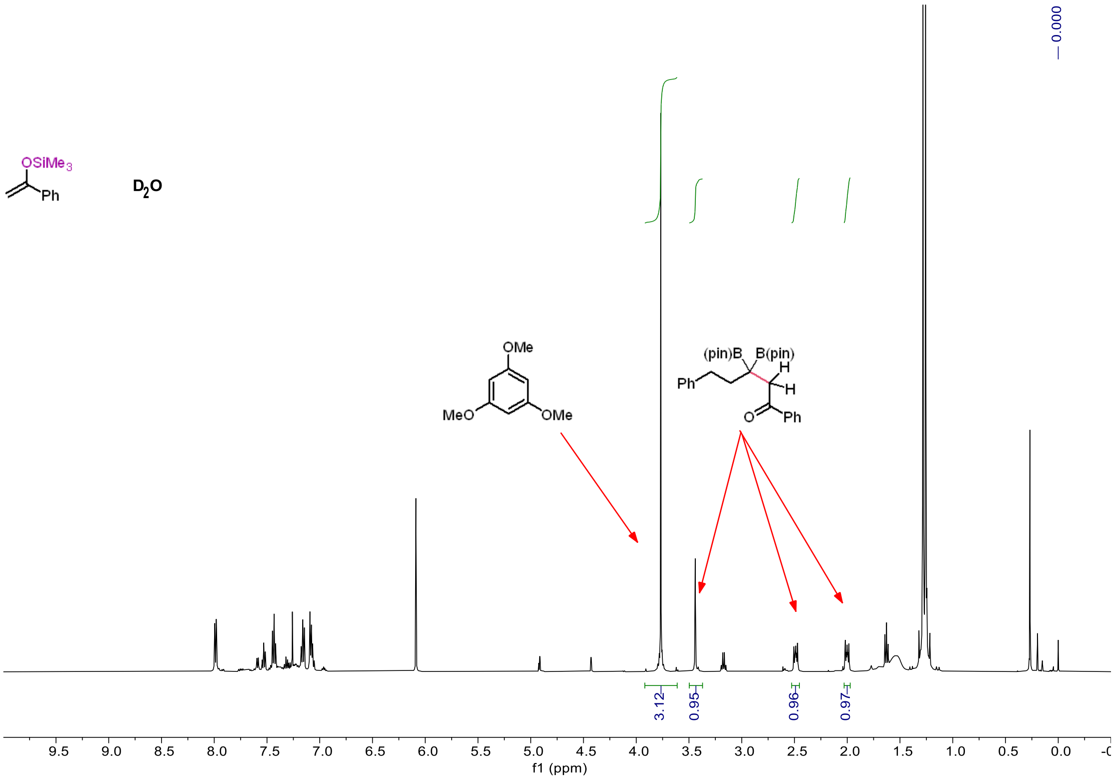

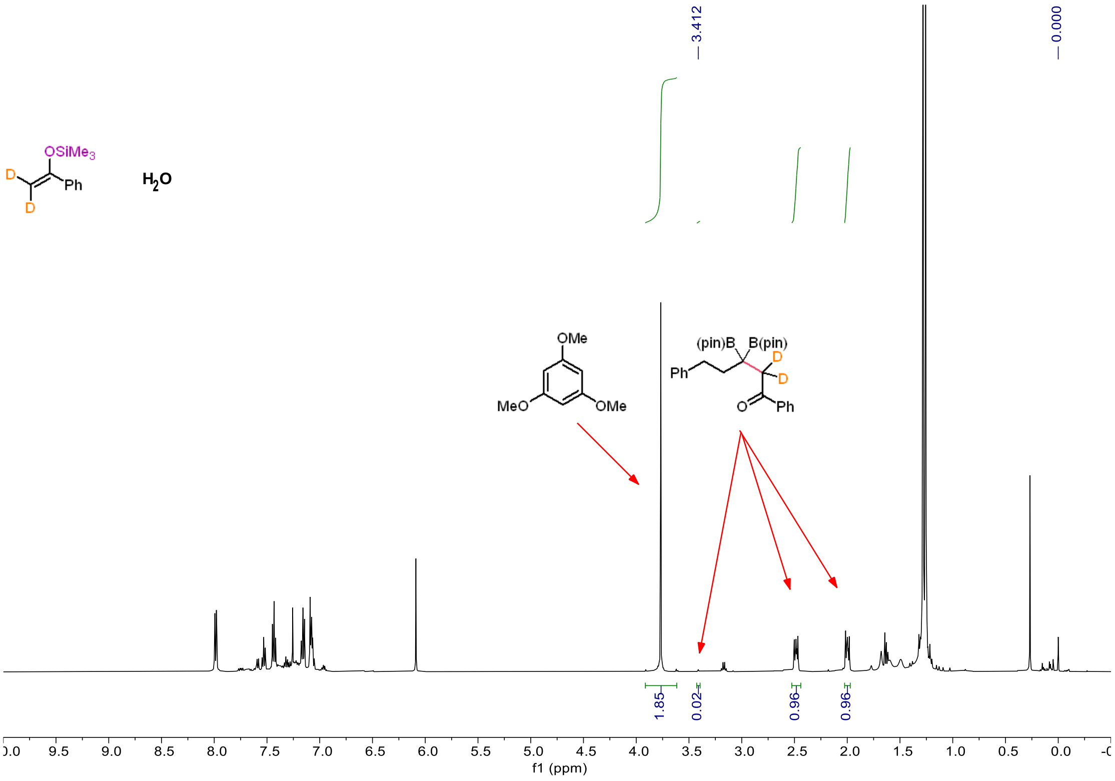

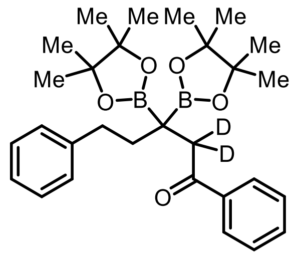

1,5-diphenyl-3,3-bis(4,4,5,5-tetramethyl-1,3,2-dioxaborolan-2-yl)pentan-1-one-2,2-*d*$_2$ (D$_2$-4a)

The title compound was prepared according to ***General Procedure A*** with **D$_2$-3a** (58.3 mg, 0.3 mmol). The crude reaction mixture was purified by column chromatography (PE:EA = 20:1 to 15:1, stain in CAM) to afford a white solid (47.4 mg, 96%).

^{1}H NMR ($CDCl_3$, 500 MHz): δ 7.99-7.98 (m, 2H), 7.55-7.52 (m, 1H), 7.45-7.42 (m, 2H), 7.17-7.14 (m, 2H), 7.09-7.05 (m, 3H), 2.50-2.47 (m, 2H), 2.01-1.98 (m, 2H), 1.28 (s, 12H), 1.26 (s, 12H); **^{13}C NMR** ($CDCl_3$, 125 MHz): δ 200.1, 143.2, 137.2, 132.7, 128.4, 128.3, 128.1, 128.0, 125.3, 83.1, 34.4, 32.5, 24.9, 24.7; **^{11}B NMR** ($CDCl_3$, 161 MHz): δ 34.2; **HRMS** (ESI-TOF): *m/z* calculated for $C_{29}H_{38}D_2B_2NaO_5^+$ $[M+Na]^+$ 515.3080, found 515.3097.

References

[1] (a) Cao, H.-Y.; Ma, S.-S.; Feng, Y.-H.; Guo, Y.-W.; Peng, J. *Chem. Commun*. **2022**, *58*, 1780–1783; (b) Horino, Y.; Kimura, M.; Tanaka, S.; Okajima, T.; Tamaru, Y. *Chem. Eur. J.* **2003**, *9*, 2419–2438.

[2] (a) Wei, Y.; Xie, X.-Y.; Liu, J.; Liu, X.; Zhang, B.; Chen, X.-Y.; Li, S.-J.; Lan, Y.; Hong, K. *Angew. Chem. Int. Ed.* **2024**, *63*, e202401050; (b) Li, M.; Wei, Y.; Chen, D.; Ning, P.-F.; Yang, Y.-F.; Xie, X.-Y.; Liu, C.; Hong, K. *Chin. J. Chem.* **2025**, *43*, 949–955.

[3] Hong, K.; Liu, X.; Morken, J. P. *J. Am. Chem. Soc*. **2014**, *136*, 10581–10584.

[4] Y, Y.-F.; Ning, P.-F.; Zhang, B.; Li, M.; Xie, X.-Y.; Hong, K. *Org. Lett.* **2024**, *26*, 10285–10290.

[5] Gao, F.-C.; Li, M.; Gu, H.-Y.; Chen, X.-Y.; Xu, S.; Wei, Y.; Hong, K. *J. Org. Chem.* **2023**, *88*, 14246–14254.

[6] Fang, T.-C.; Xu, L.-X.; Qin, Y.-C.; Jiang, N.-Q.; Liu, C. *Chin. J. Org. Chem.* **2023**, *43*, 777–780.

[7] Nagaraju, A.; Saiaede, T.; Eghbarieh, N.; Masarwa, A. *Chem. Eur. J.* **2023**, *29*, e202202646.

[8] Chen, X.-Y.; Gao, F.-C.; Ning, P.-F.; Wei, Y.; Hong, K. *Angew. Chem. Int. Ed.* **2023**, *62*, e202302638.

[9] Paterson, I.; Paquet, T. *Org. Lett.* **2010**, *12*, 2158–2161.

[10] Kim, G.; Kim, M.; Ryu, C.; Choi, J.; Cho, S. H. *Org. Lett.* **2023**, *25*, 4130–4134.

[11] Inoue, M.; Carson, M. W.; Frontier, A. J.; Danishefsky, S. J. *J. Am. Chem. Soc*. **2001**, *123*, 1878–1889.

[12] Banik, B. K.; Chapa, M.; Marquez, J.; Cardona, M. *Tetrahedron Lett.* **2005**, *46*, 2341–2343.

[13] Sonawane, R. P.; Jheengut, V.; Rabalakos, C.; Larouche-Gauthier, R.; Scott, H. K.; Aggarwal, V. K. *Angew. Chem. Int. Ed*. **2011**, *50*, 3760–3763.

[14] Bonet, A.; Odachowski, M.; Leonori, D.; Essafi, S.; Aggarwal, V. K. *Nat. Chem.* **2014**, *6*, 584–589.

[15] Hwang, C.; Jo, W.; Cho, S. H. *Chem. Commun*. **2017**, *53*, 7573–7576.

NMR Spectra

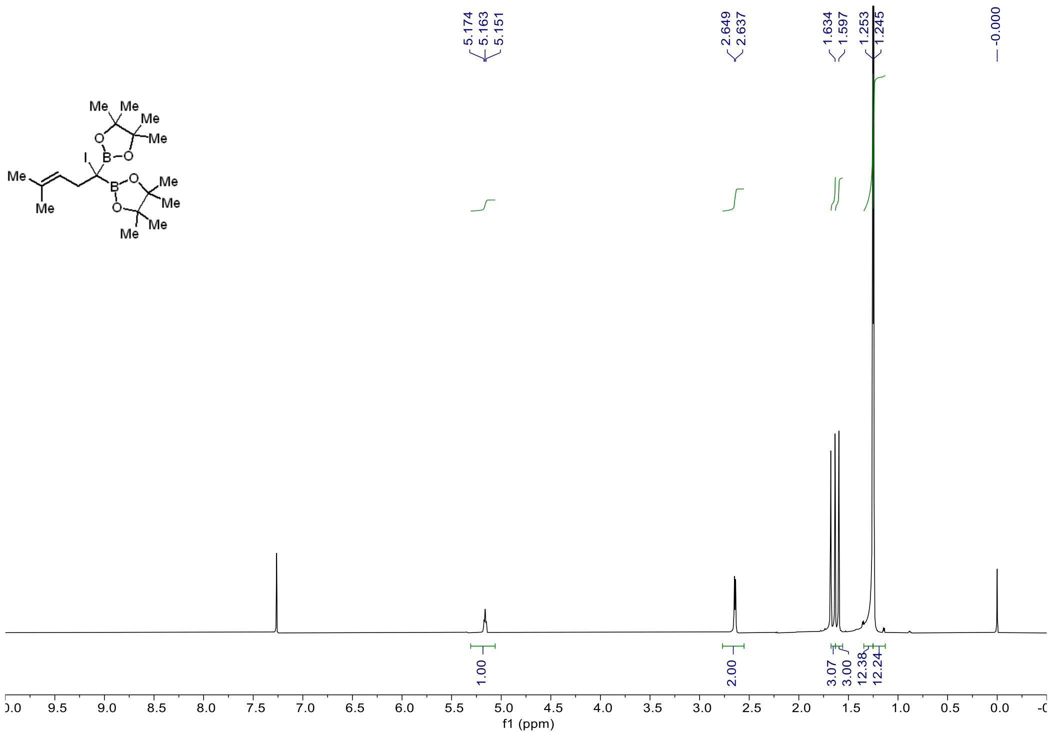

^{1}H NMR (600 MHz, $CDCl_3$) spectrum of compound **2f**

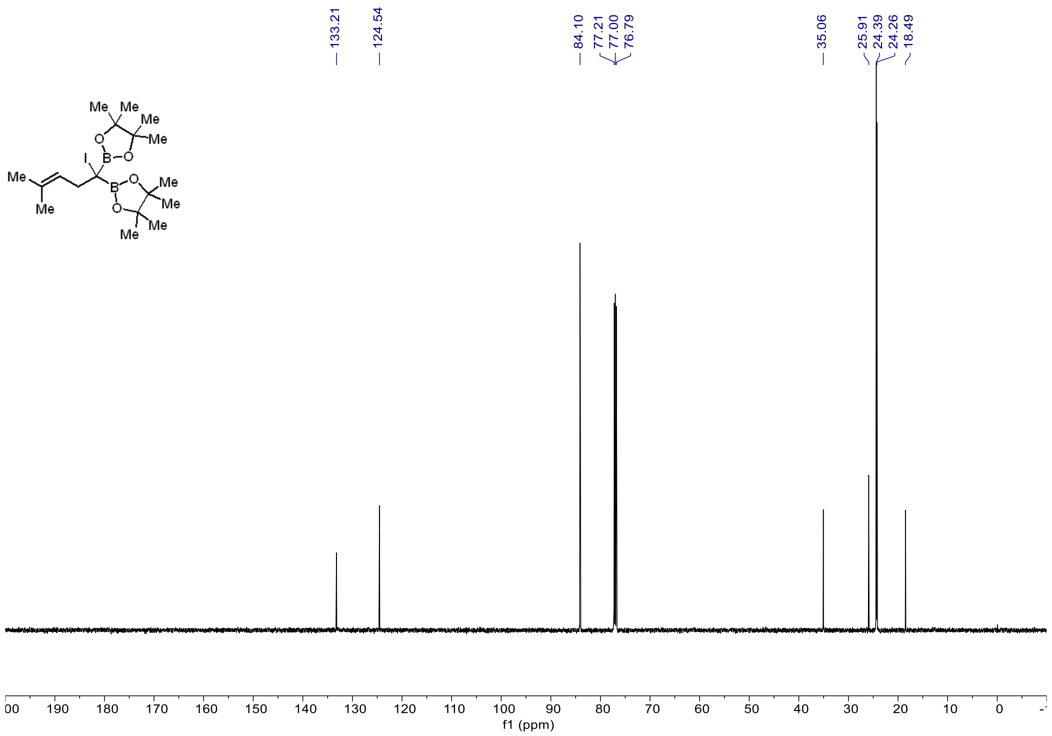

^{13}C NMR (150 MHz, $CDCl_3$) spectrum of compound **2f**

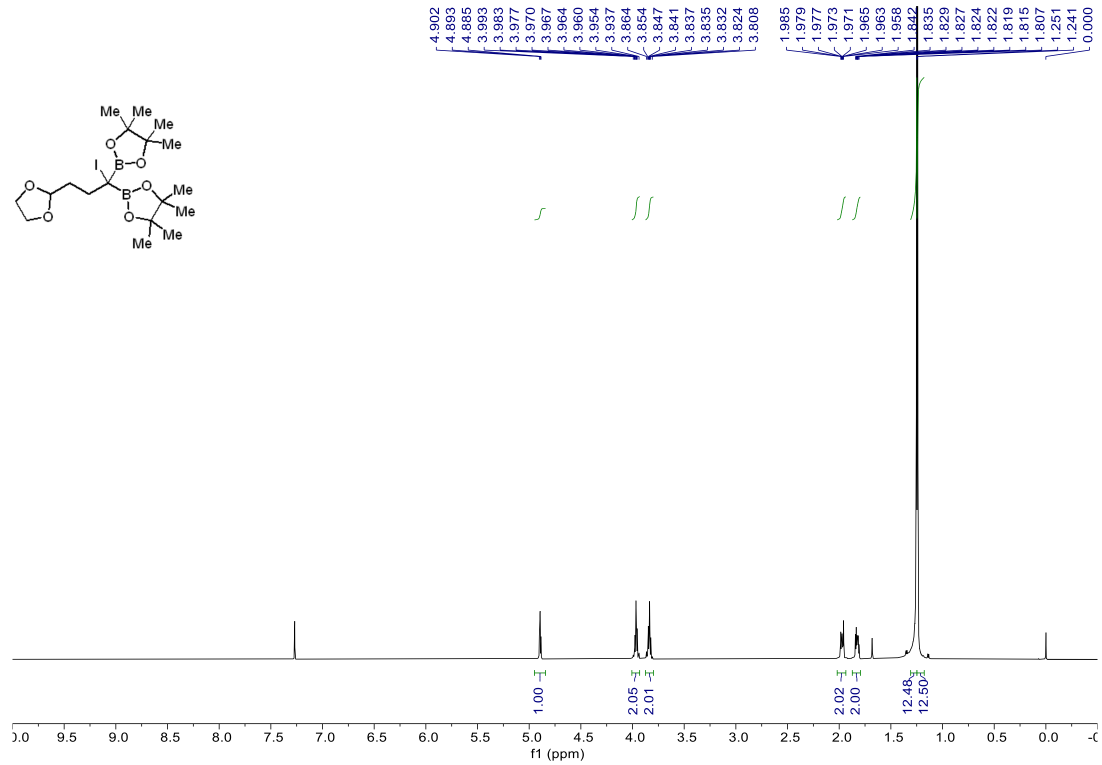

^{1}H NMR (600 MHz, $CDCl_3$) spectrum of compound **2j**

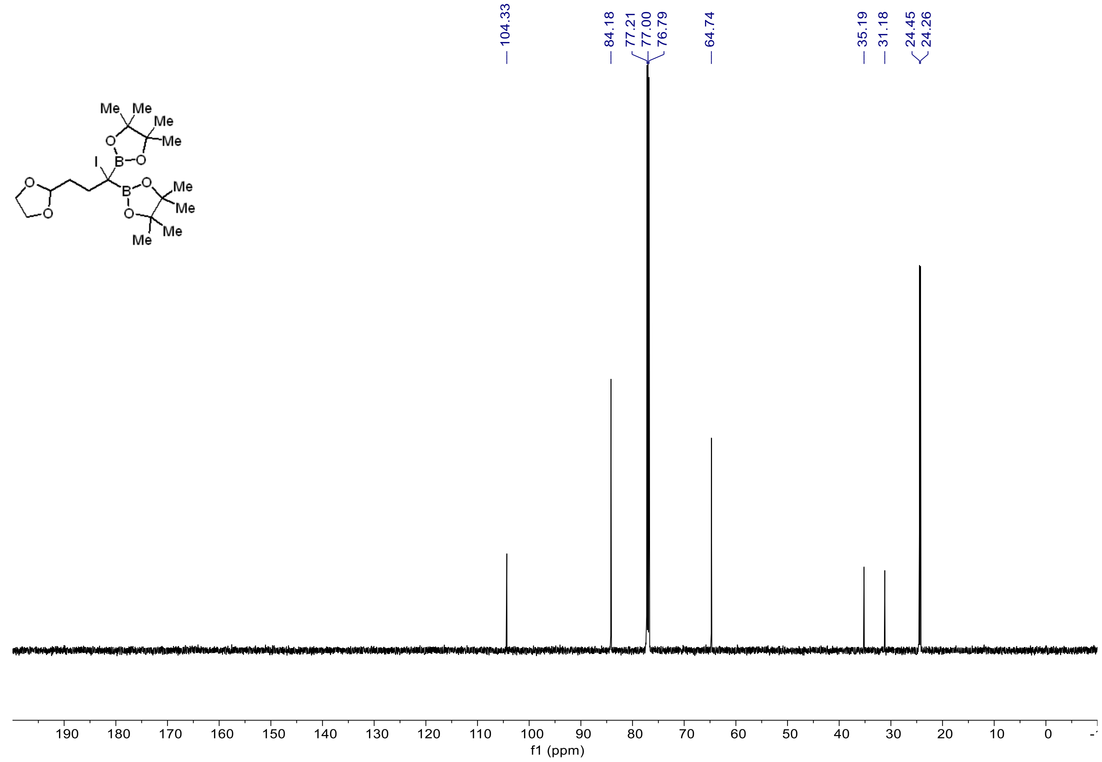

^{13}C NMR (150 MHz, $CDCl_3$) spectrum of compound **2j**

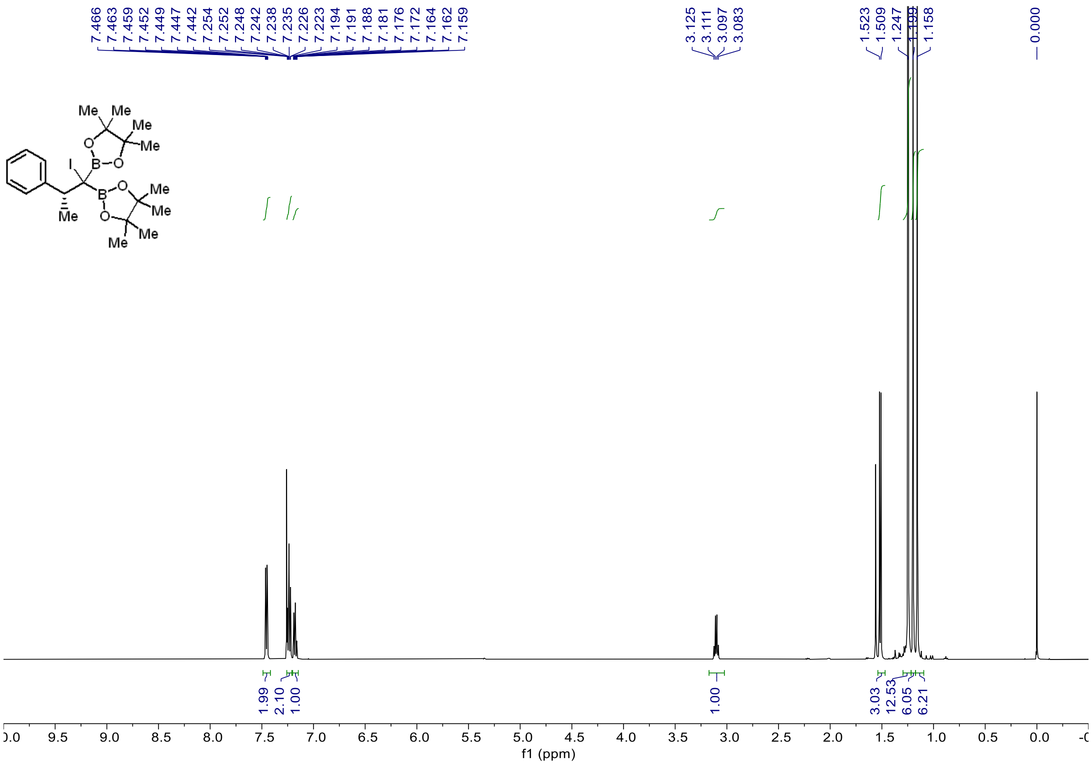

^{1}H NMR (500 MHz, $CDCl_3$) spectrum of compound **2n**

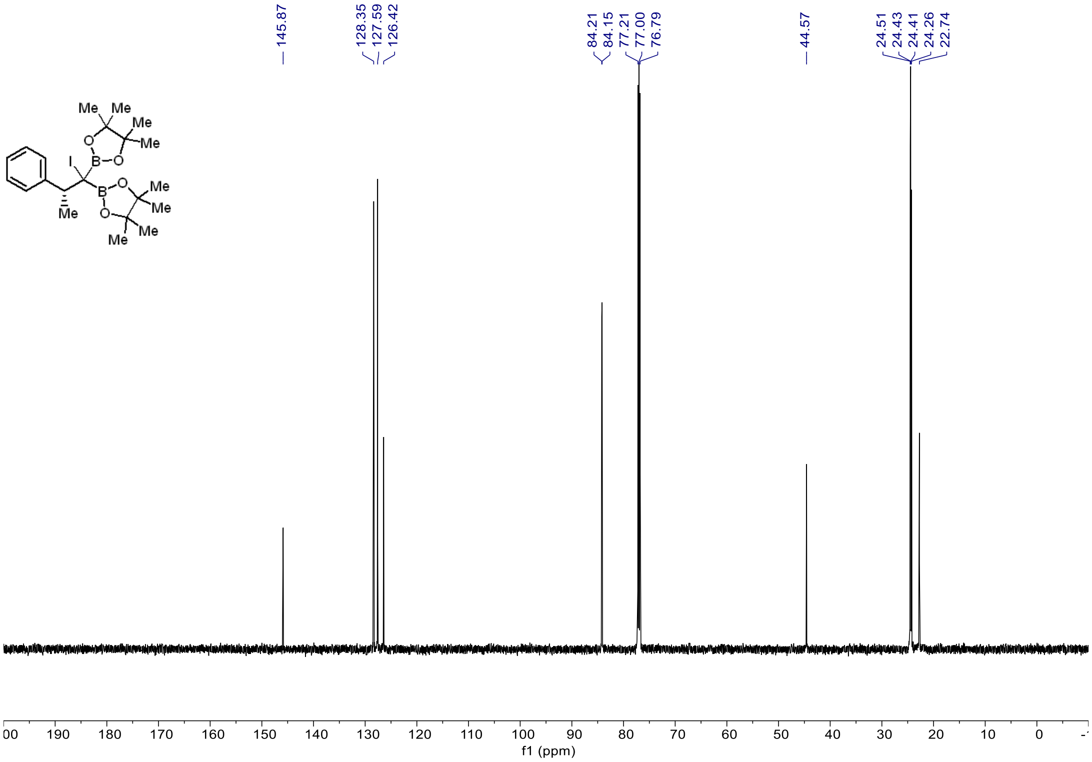

^{13}C NMR (150 MHz, $CDCl_3$) spectrum of compound **2n**

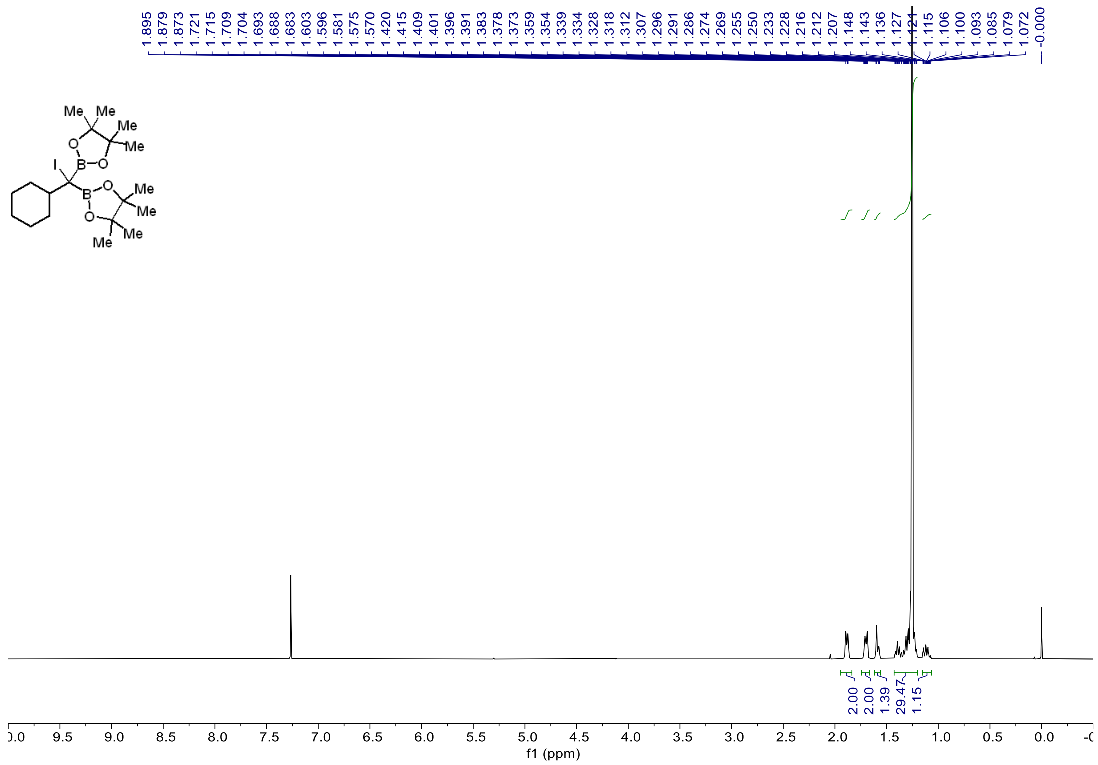

^{1}H NMR (500 MHz, $CDCl_3$) spectrum of compound **2r**

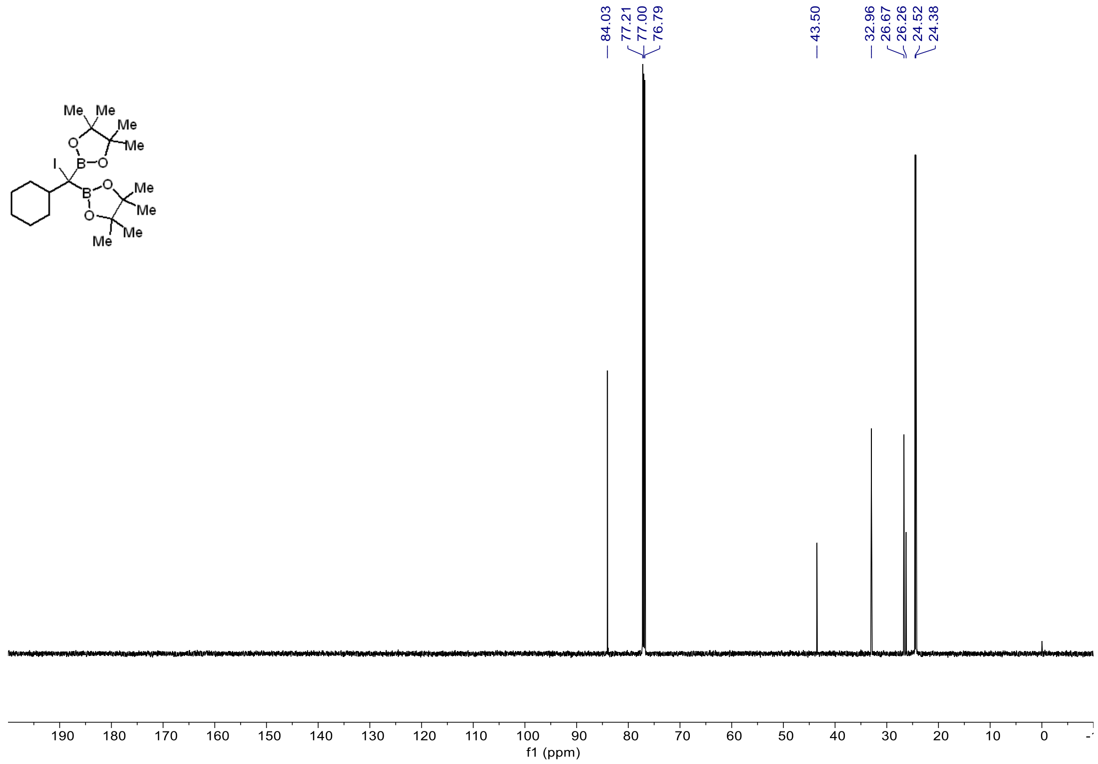

^{13}C NMR (150 MHz, $CDCl_3$) spectrum of compound **2r**

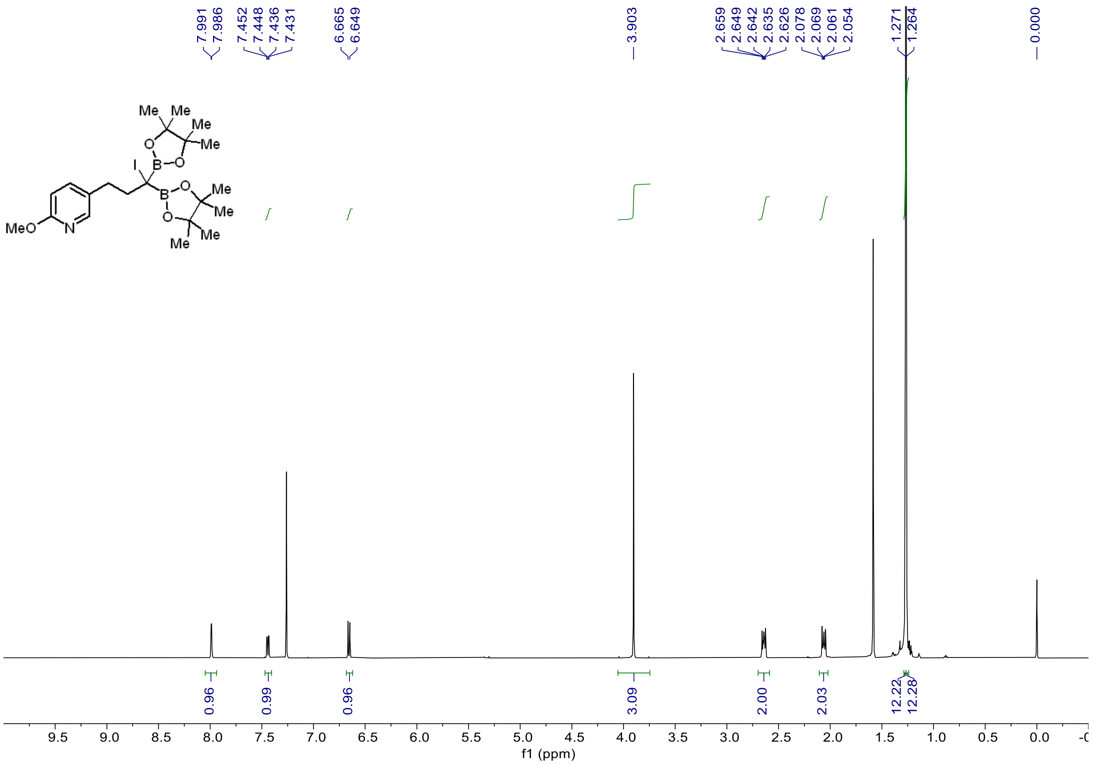

^{1}H NMR (500 MHz, $CDCl_3$) spectrum of compound **2c**

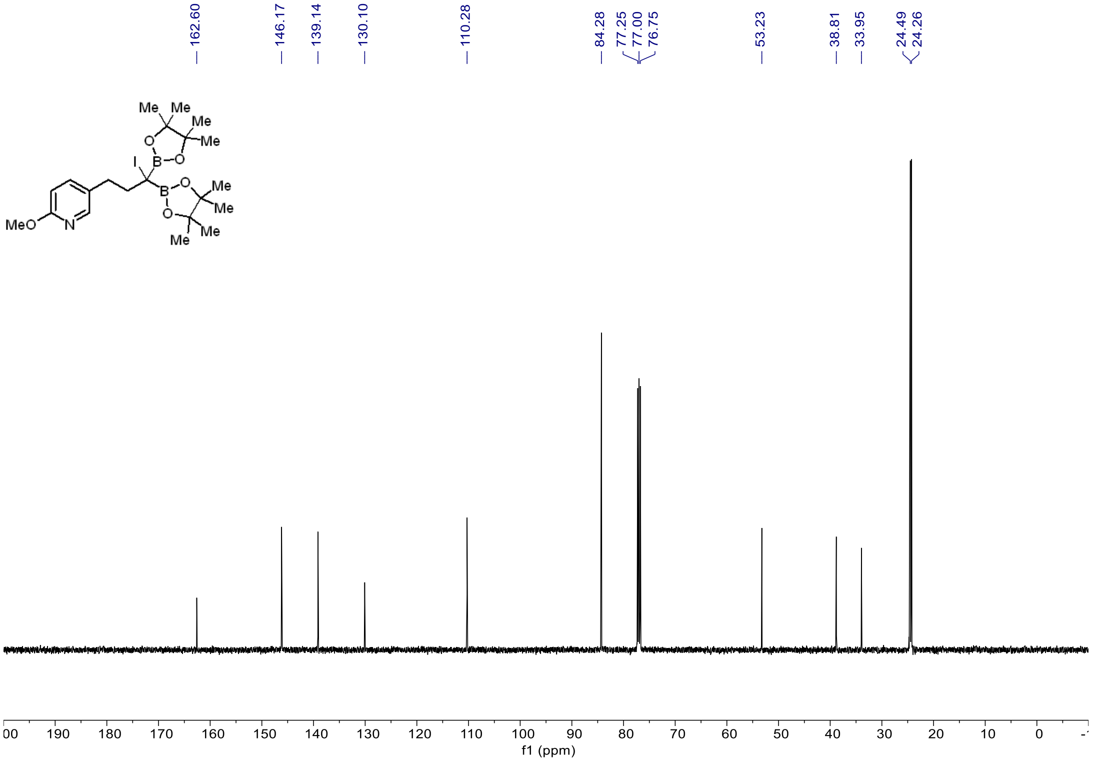

^{13}C NMR (125 MHz, $CDCl_3$) spectrum of compound **2c**

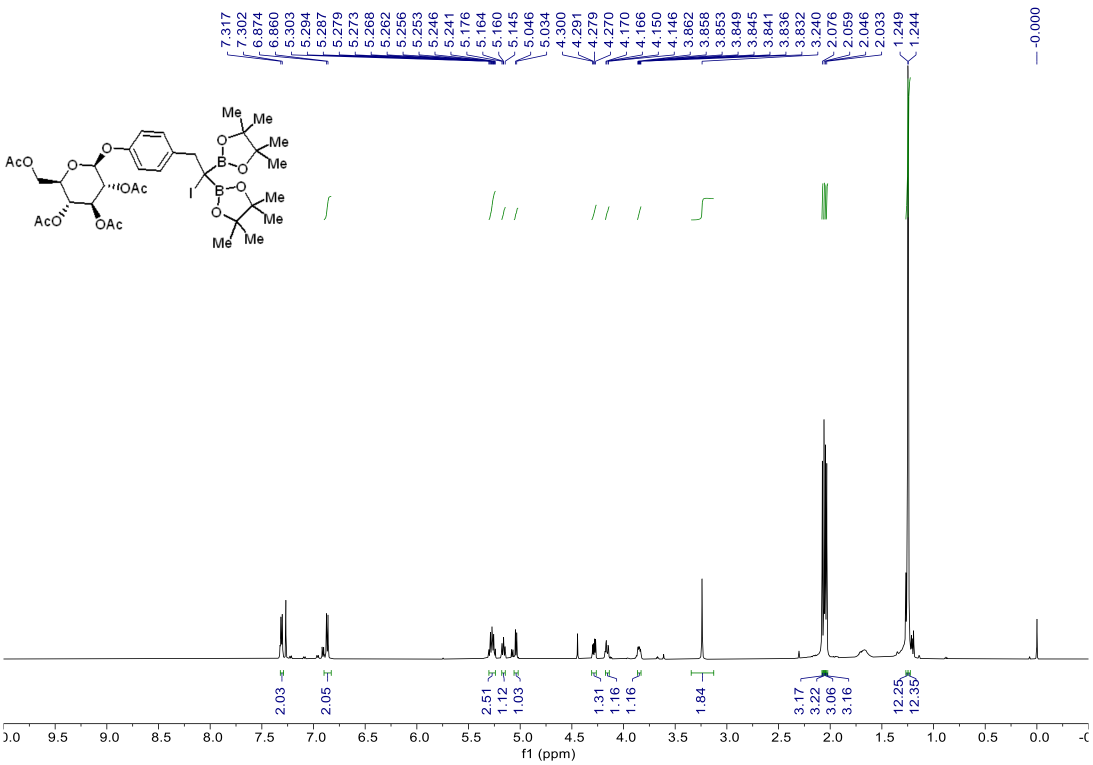

^{1}H NMR (600 MHz, $CDCl_3$) spectrum of compound **2t**

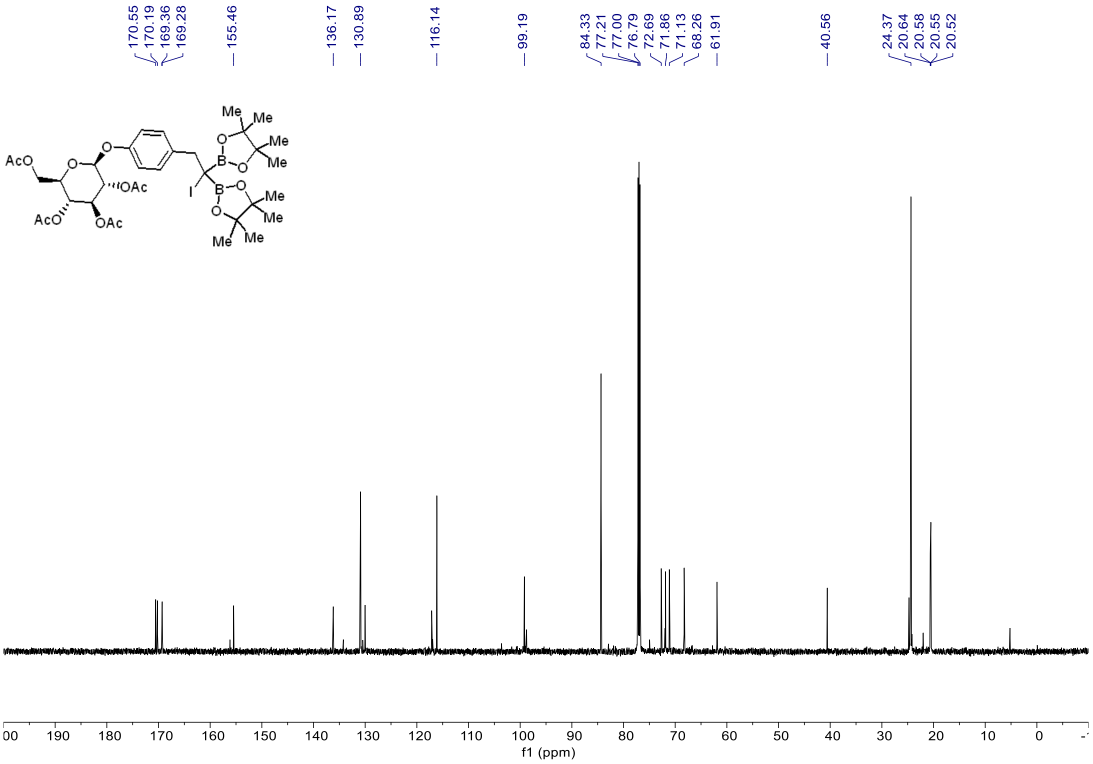

^{13}C NMR (150 MHz, $CDCl_3$) spectrum of compound **2t**

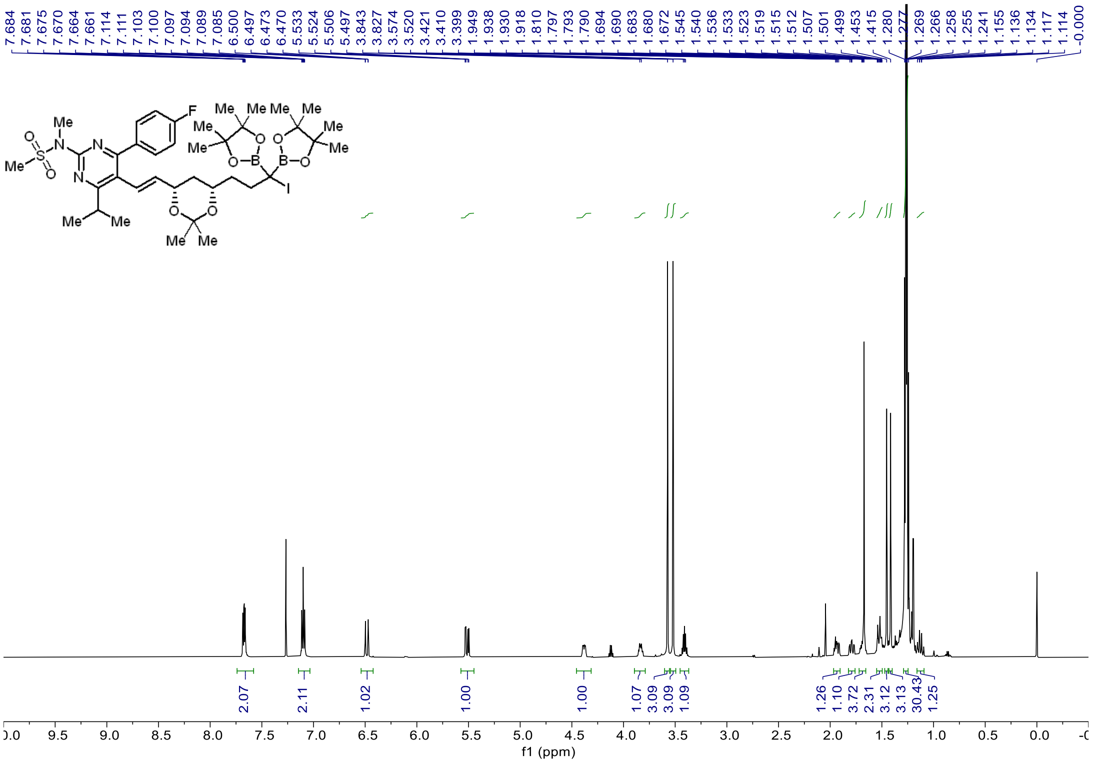

^{1}H NMR (500 MHz, $CDCl_3$) spectrum of compound **2o**

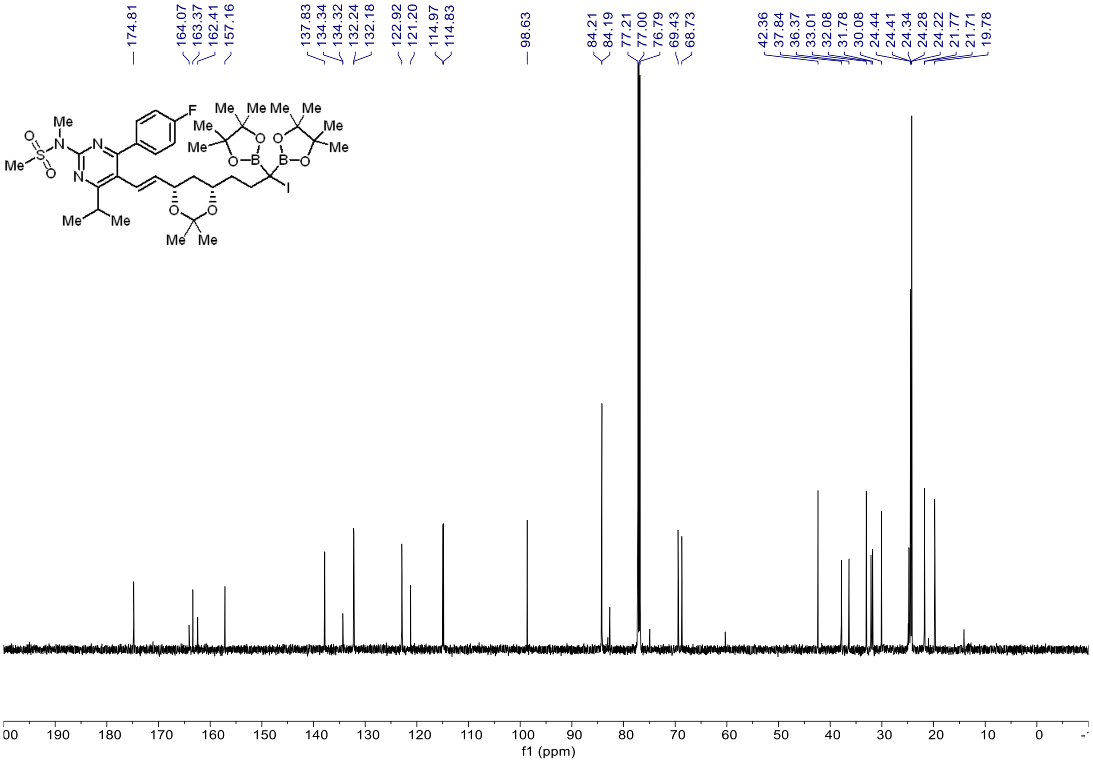

^{13}C NMR (150 MHz, $CDCl_3$) spectrum of compound **2o**

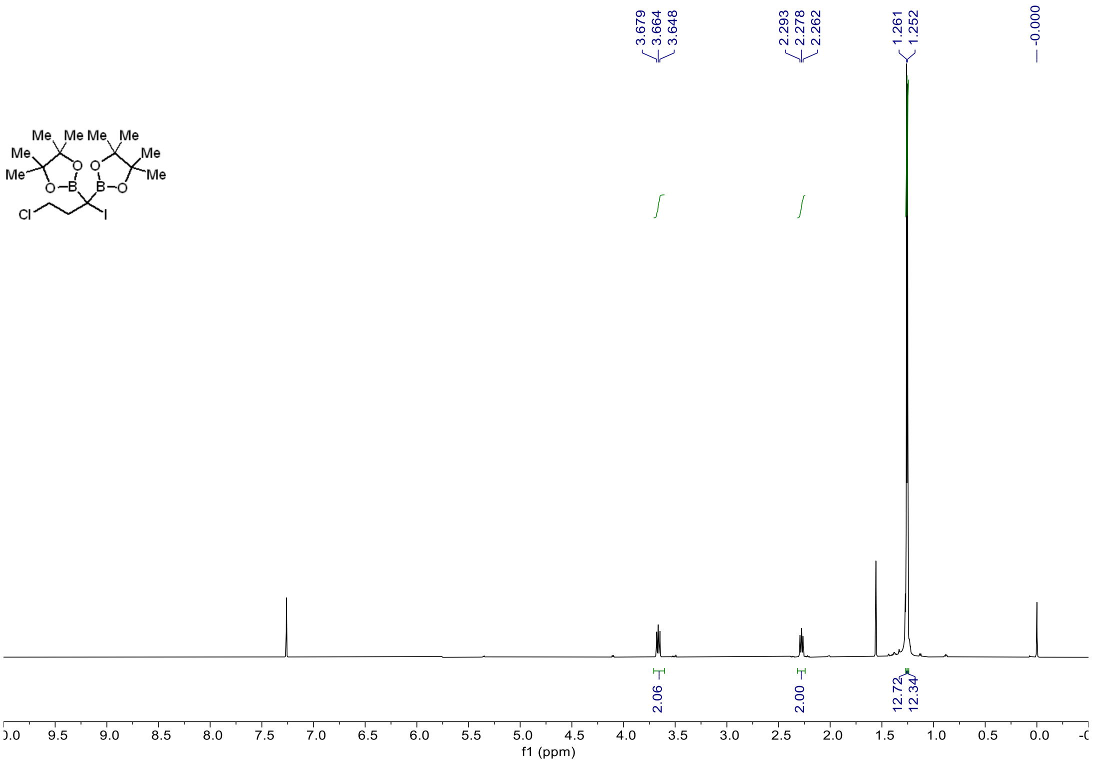

^{1}H NMR (500 MHz, $CDCl_3$) spectrum of compound **2h**

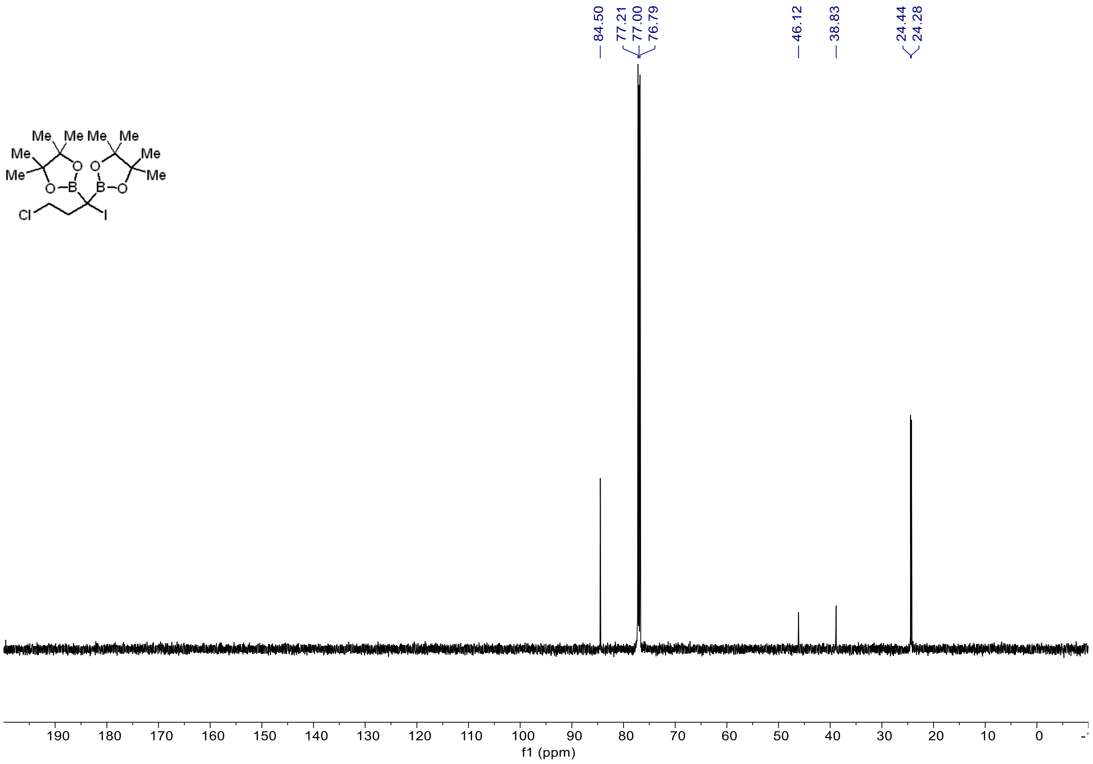

^{13}C NMR (150 MHz, $CDCl_3$) spectrum of compound **2h**

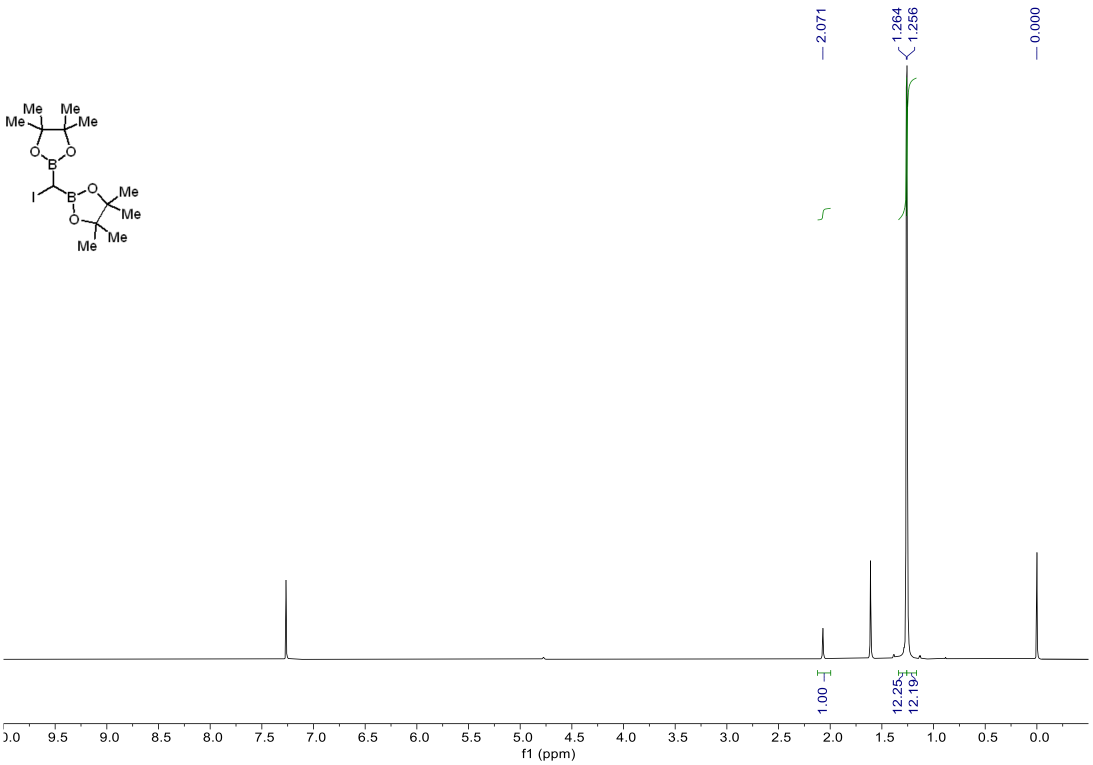

^{1}H NMR (500 MHz, $CDCl_3$) spectrum of compound **2k**

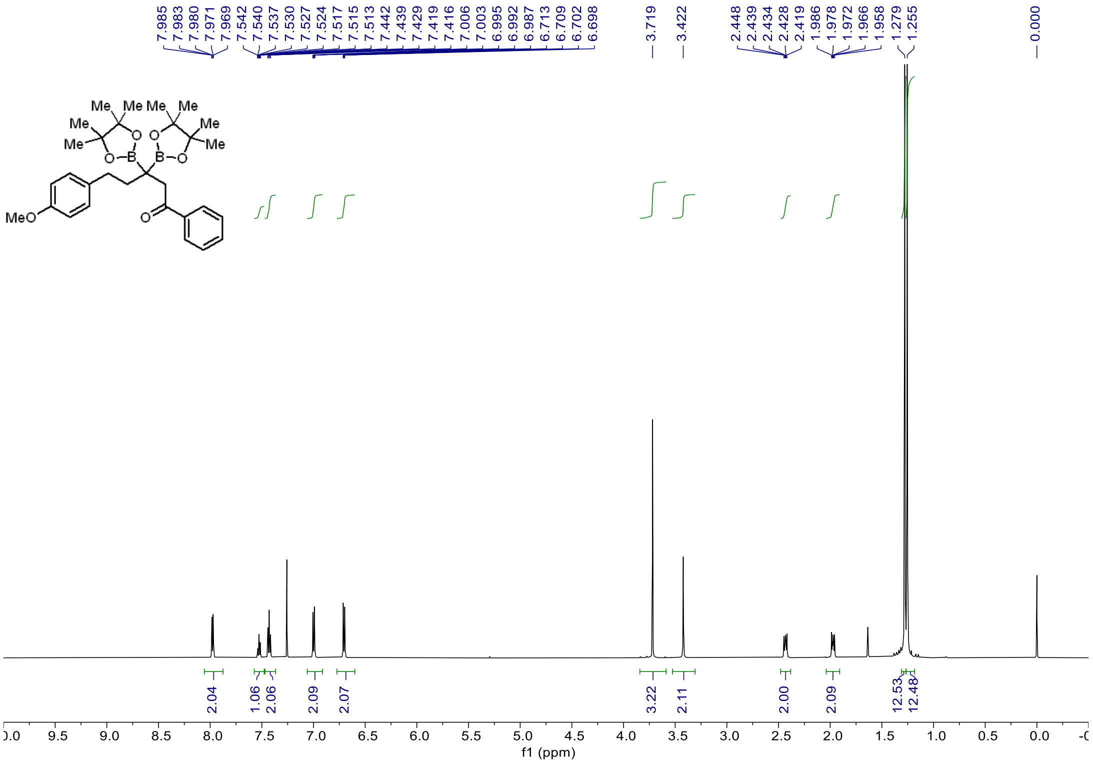

^{1}H NMR (600 MHz, $CDCl_3$) spectrum of compound **4b**

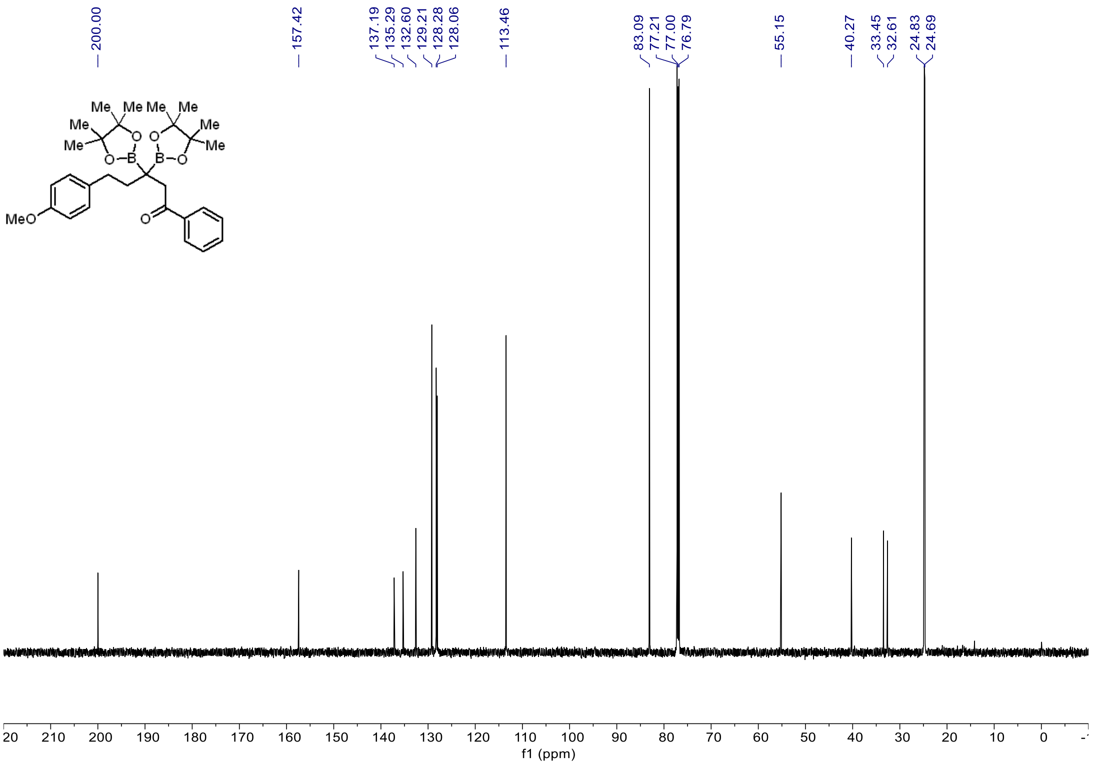

^{13}C NMR (150 MHz, $CDCl_3$) spectrum of compound **4b**

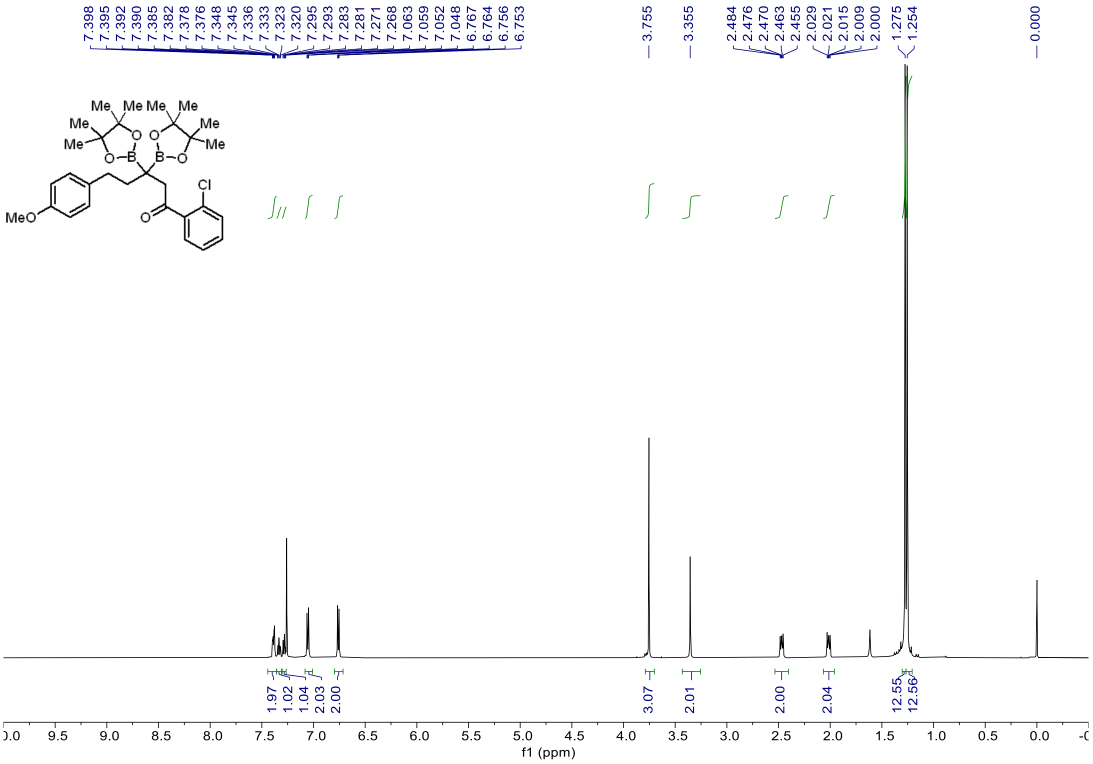

^{1}H NMR (600 MHz, $CDCl_3$) spectrum of compound **4c**

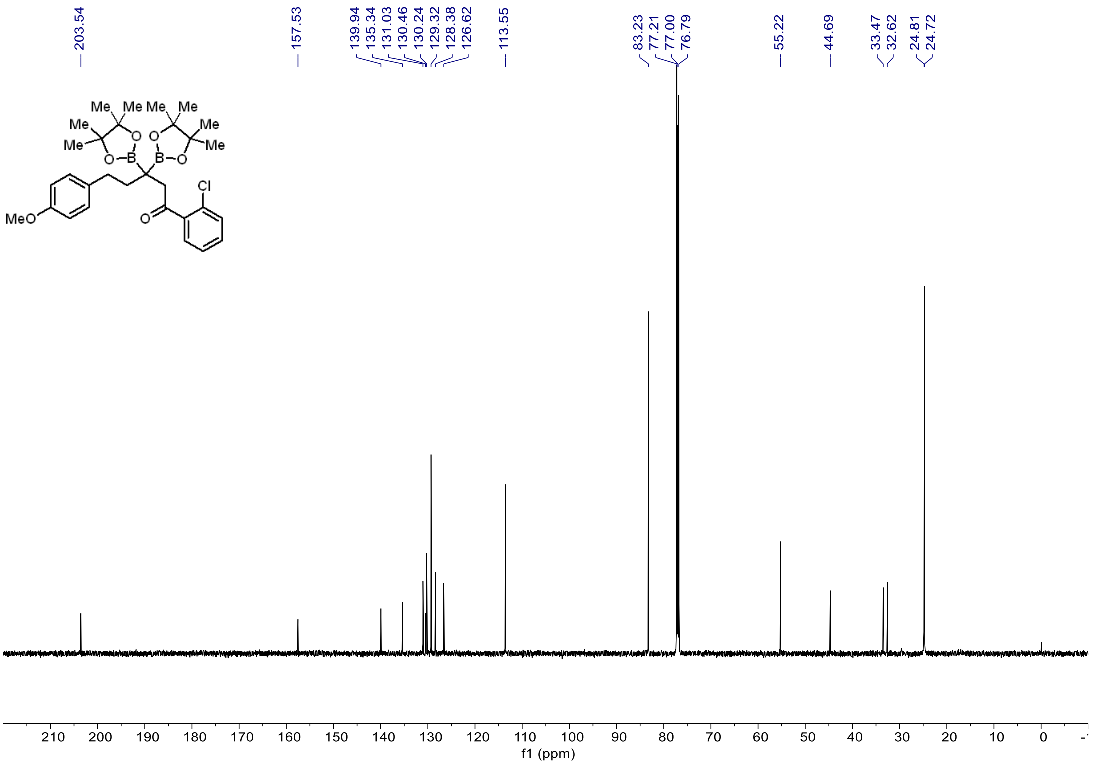

^{13}C NMR (150 MHz, $CDCl_3$) spectrum of compound **4c**

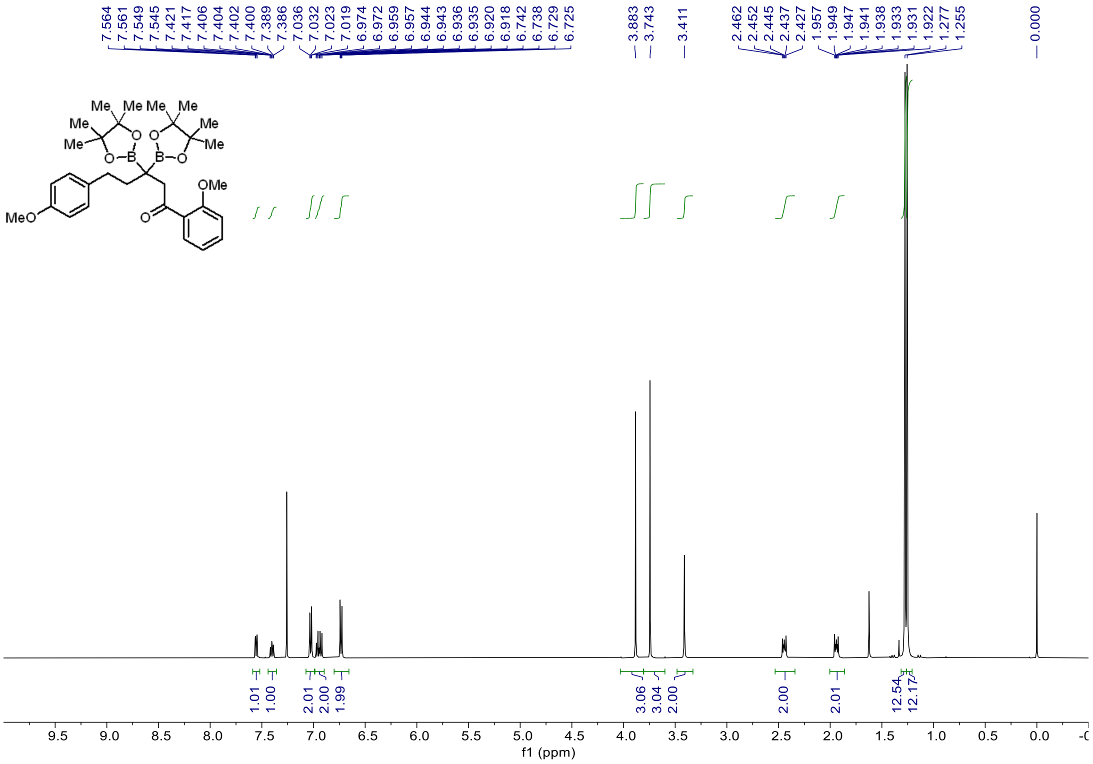

^{1}H NMR (500 MHz, $CDCl_3$) spectrum of compound **4d**

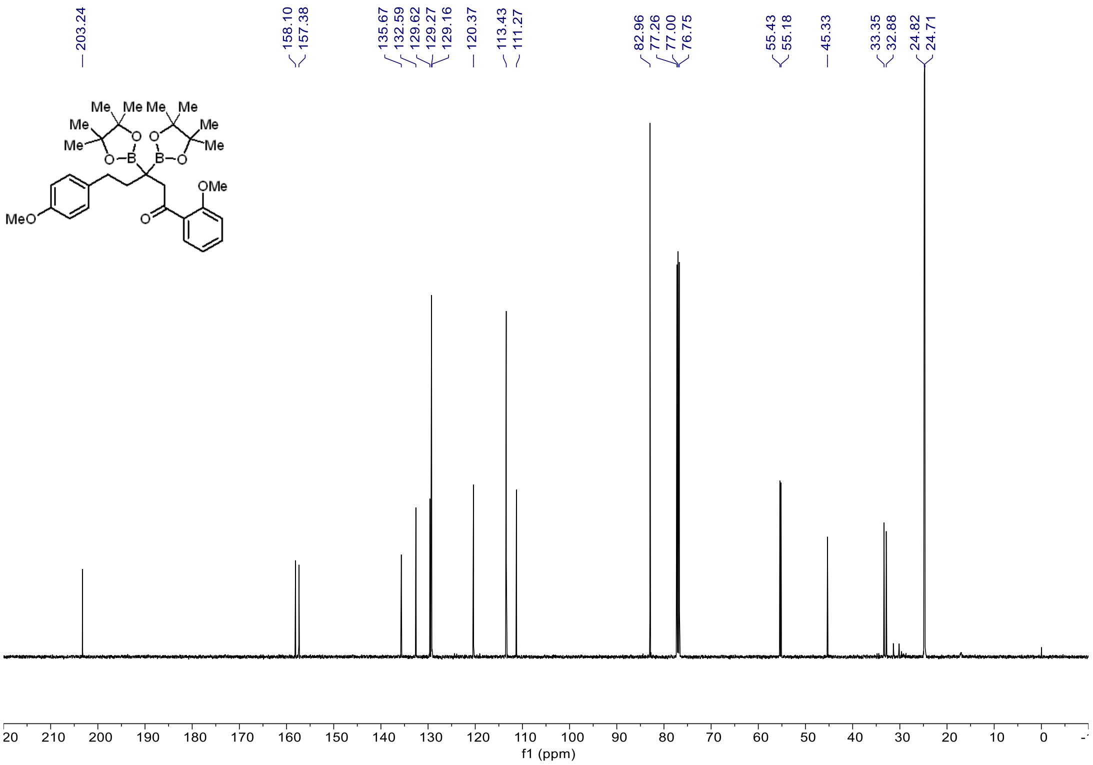

^{13}C NMR (125 MHz, $CDCl_3$) spectrum of compound **4d**

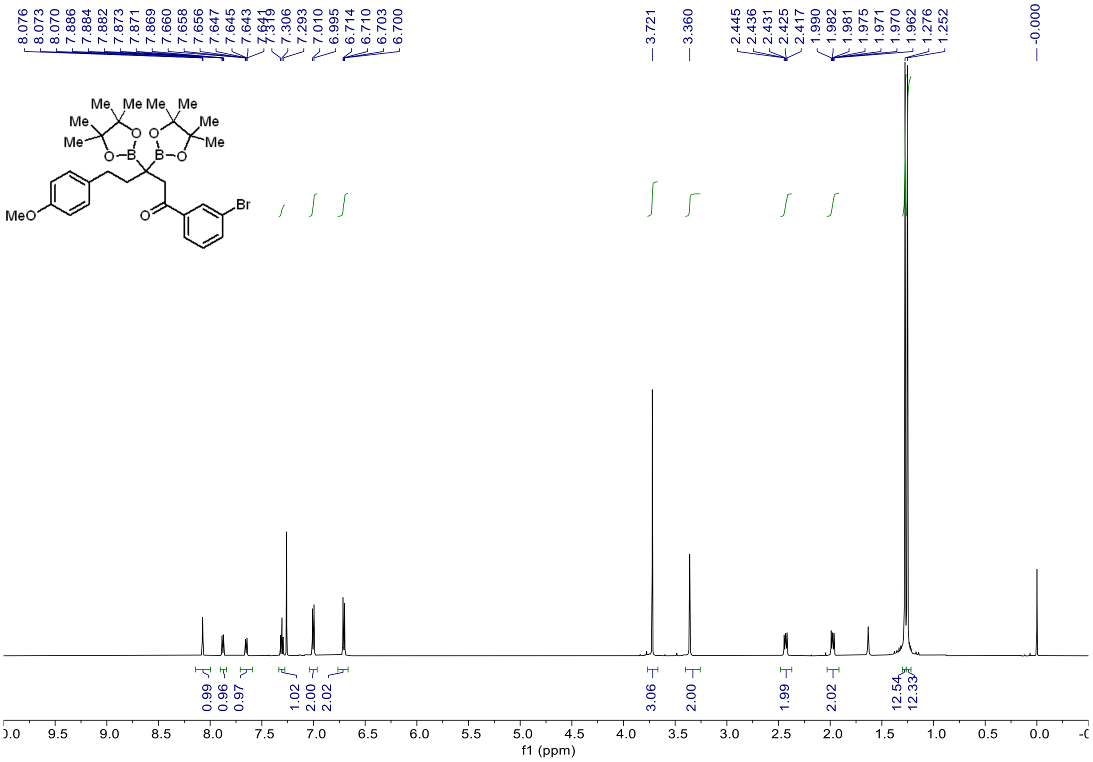

^{1}H NMR (600 MHz, $CDCl_3$) spectrum of compound **4e**

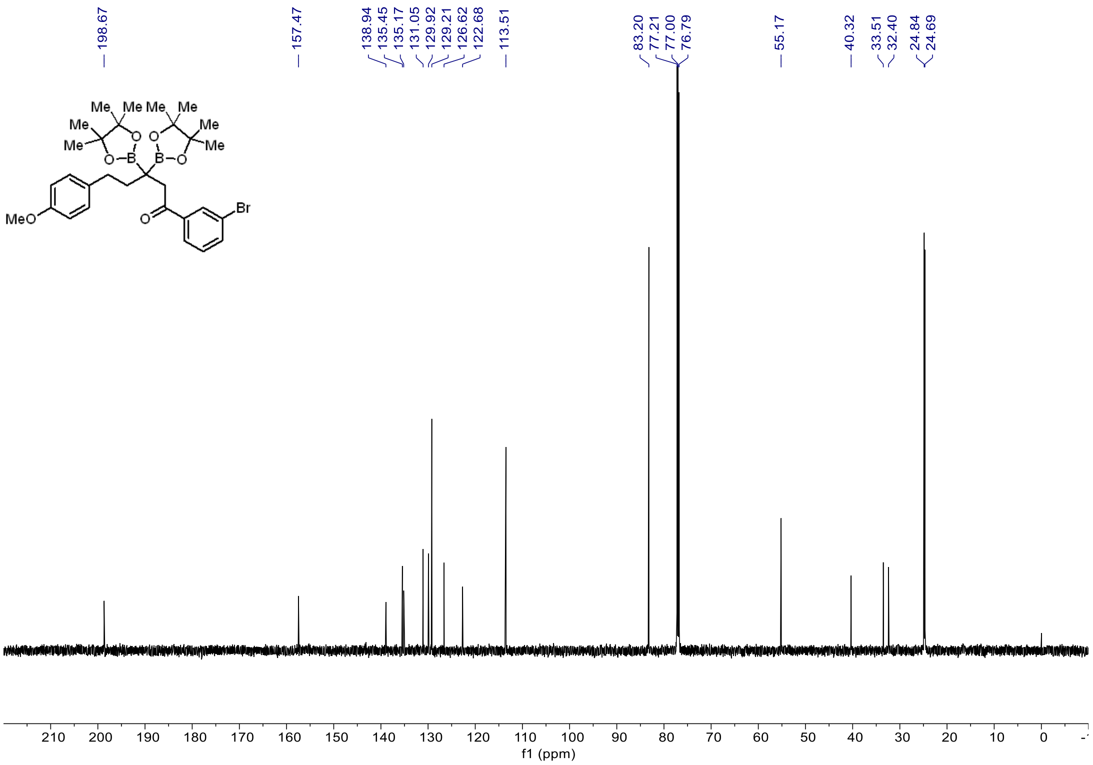

^{13}C NMR (150 MHz, $CDCl_3$) spectrum of compound **4e**

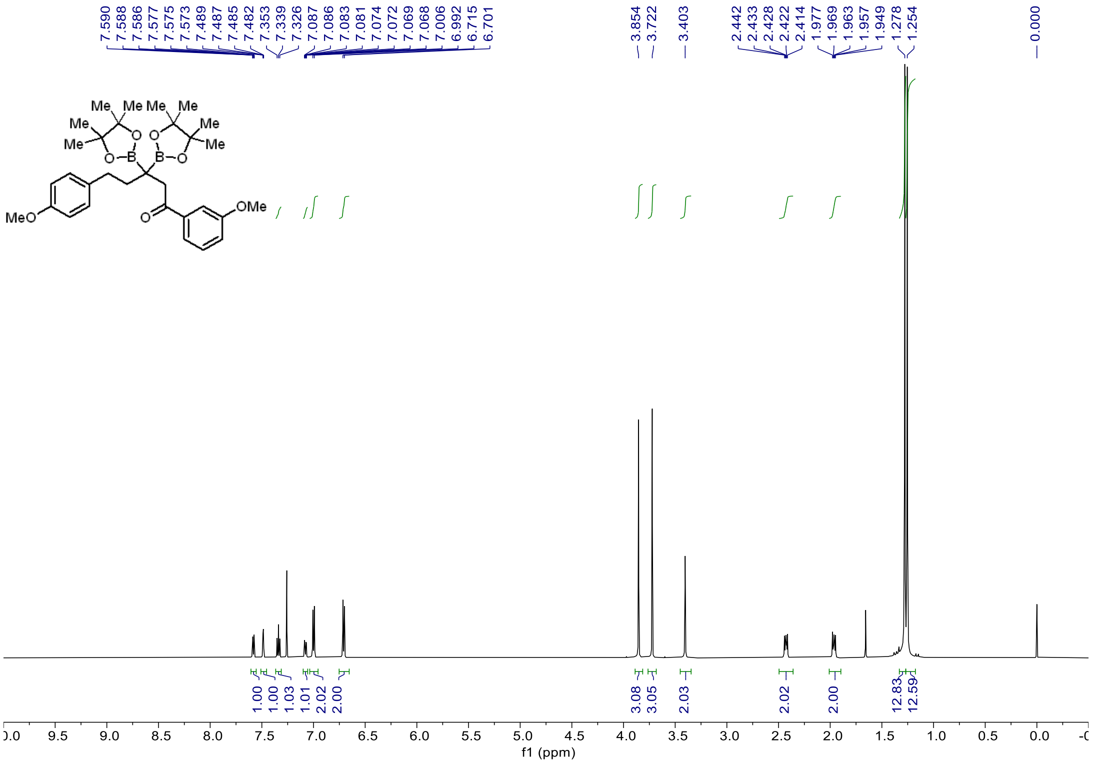

^{1}H NMR (600 MHz, $CDCl_3$) spectrum of compound **4f**

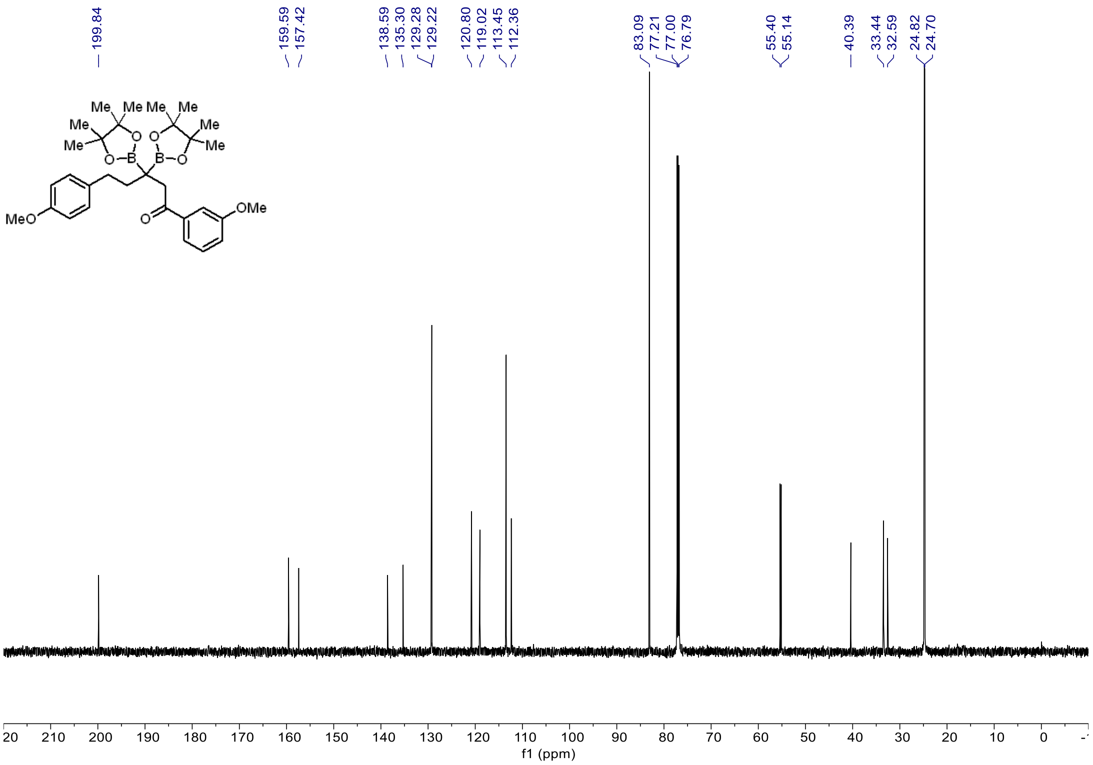

^{13}C NMR (150 MHz, CDCl$_3$) spectrum of compound **4f**

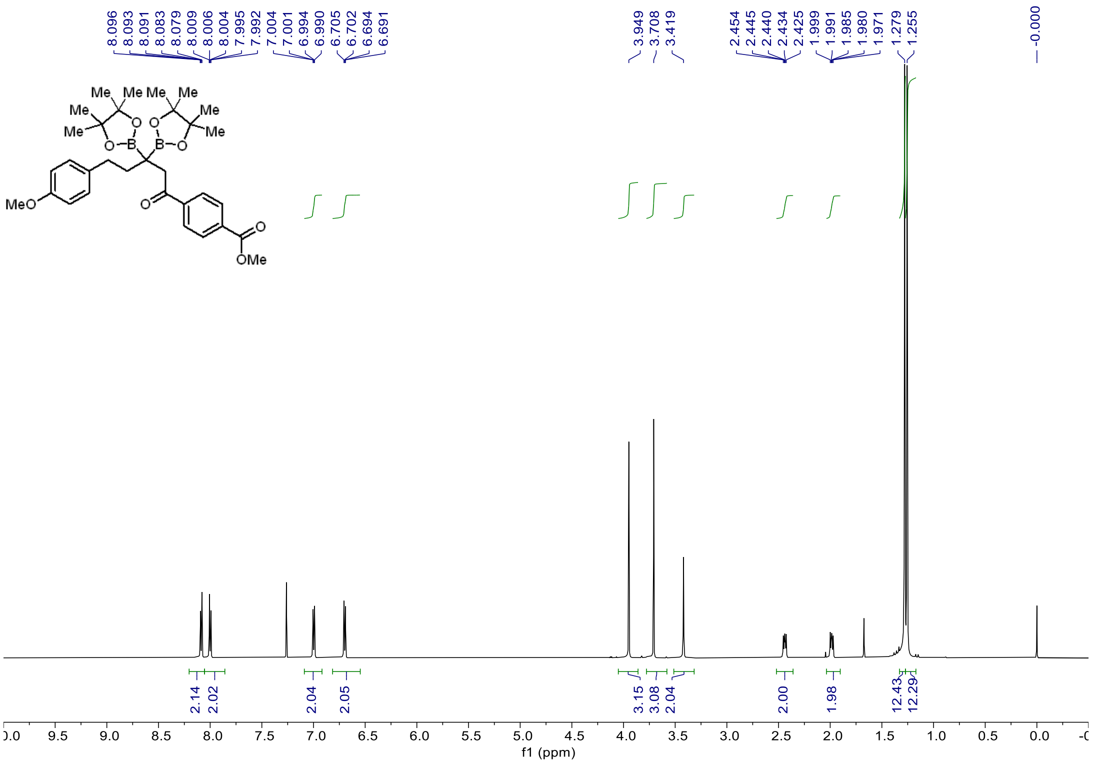

^{1}H NMR (600 MHz, CDCl$_3$) spectrum of compound **4g**

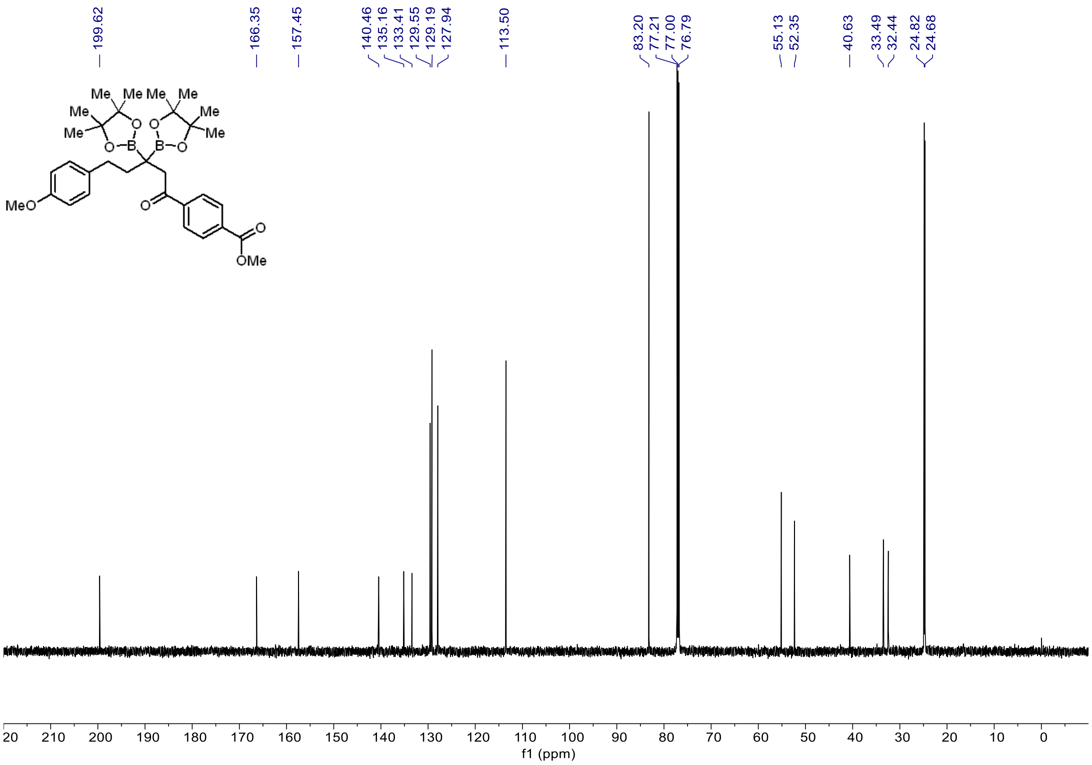

^{13}C NMR (150 MHz, $CDCl_3$) spectrum of compound **4g**

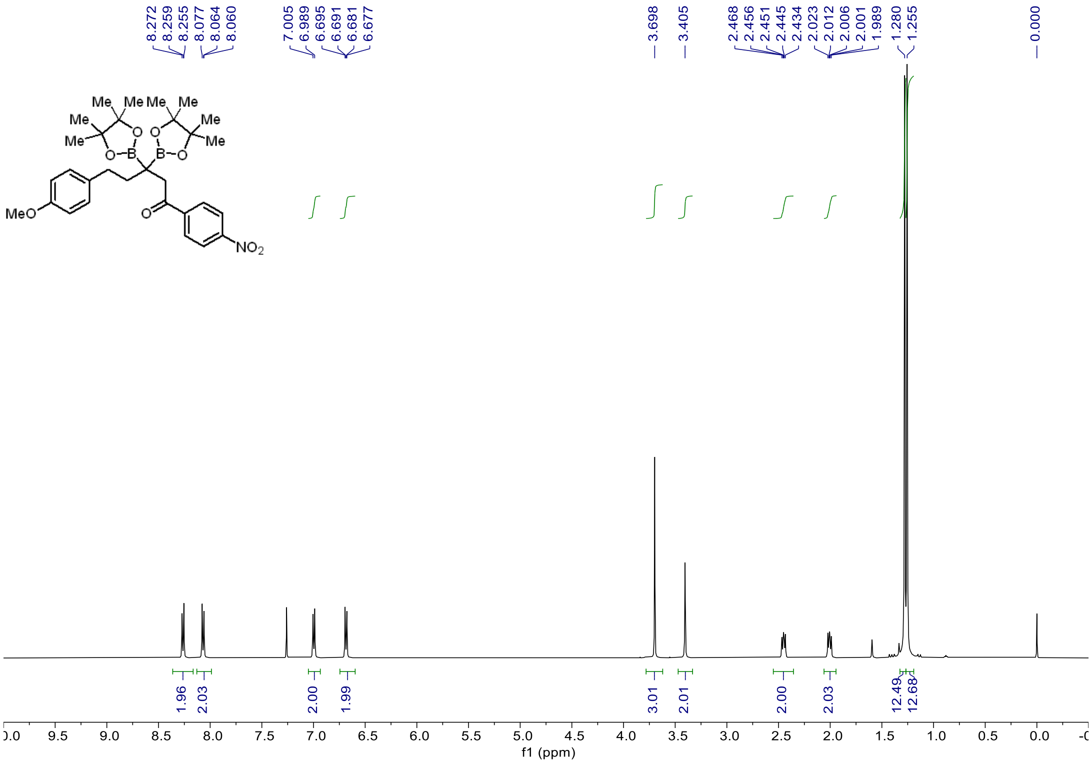

^{1}H NMR (500 MHz, $CDCl_3$) spectrum of compound **4h**

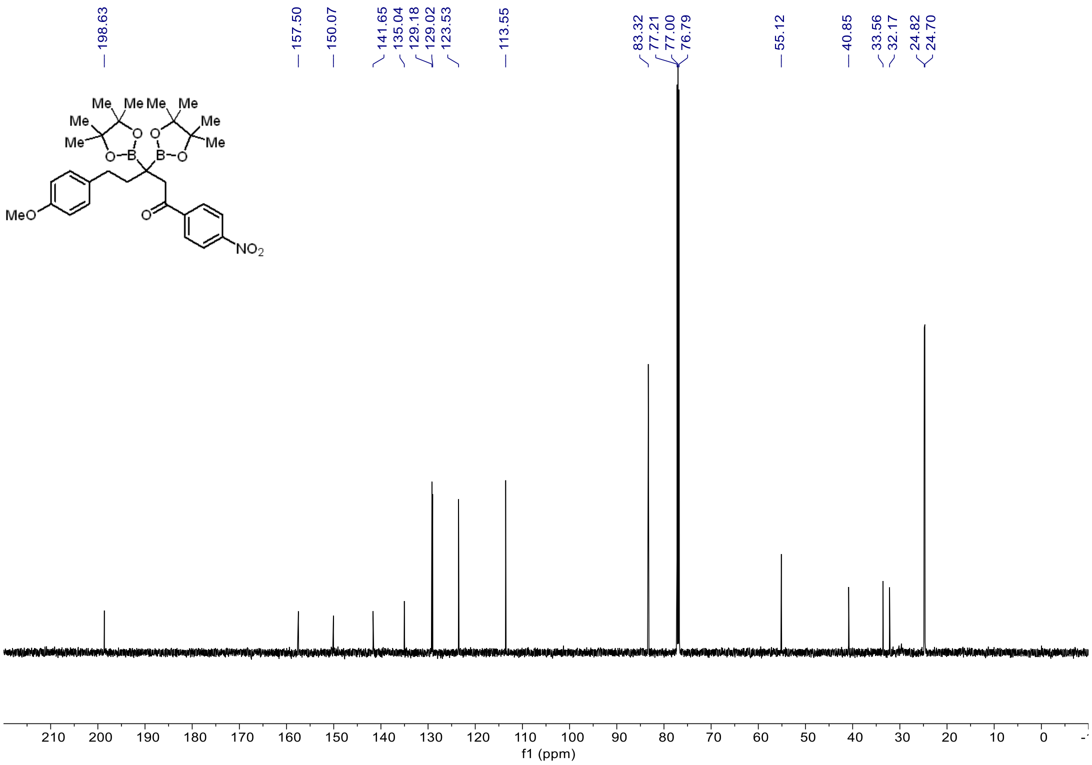

^{13}C NMR (150 MHz, $CDCl_3$) spectrum of compound **4h**

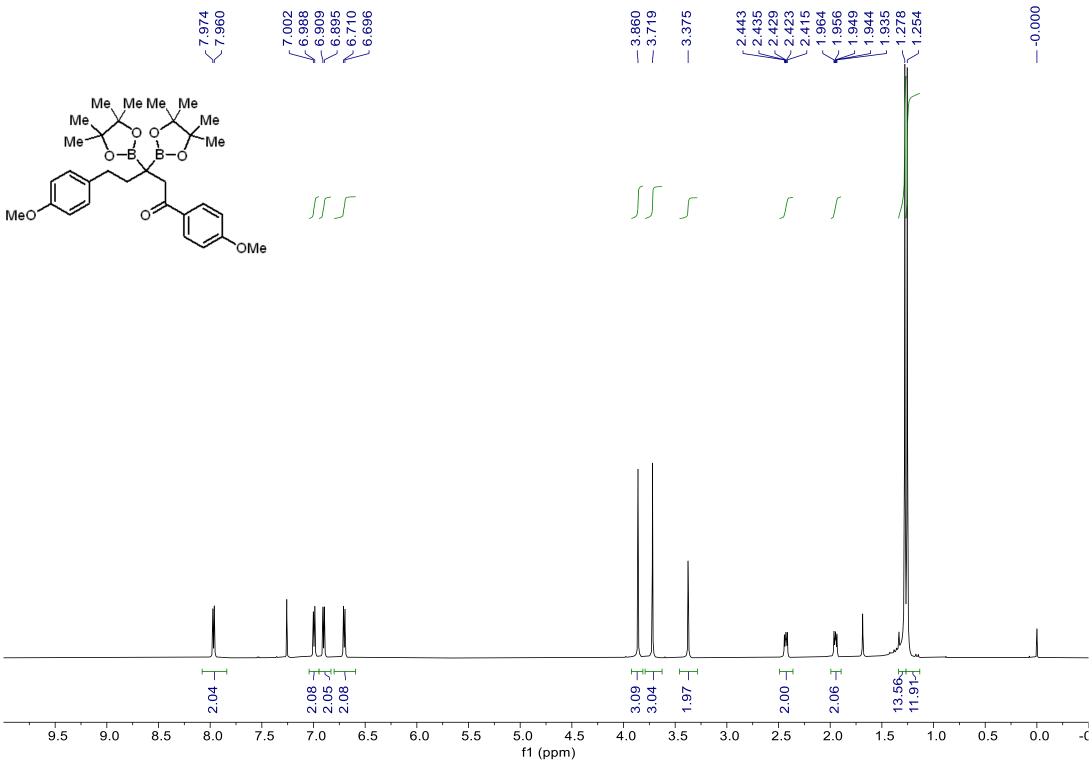

^{1}H NMR (600 MHz, $CDCl_3$) spectrum of compound **4i**

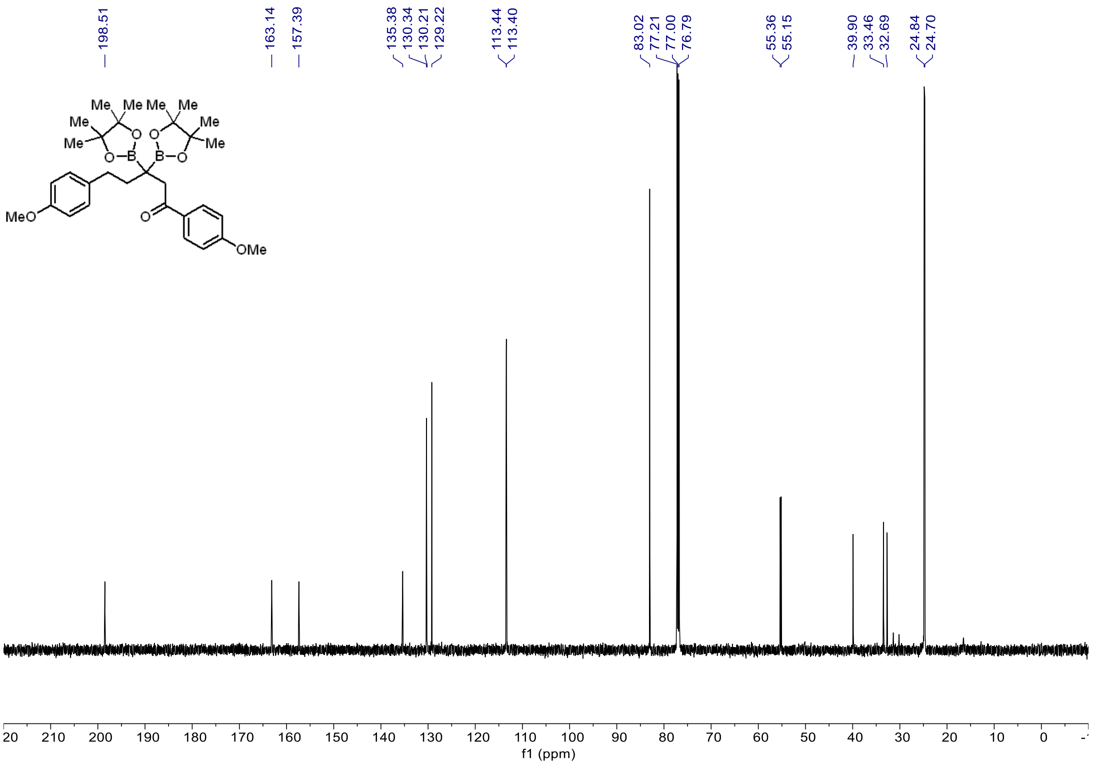

^{13}C NMR (150 MHz, $CDCl_3$) spectrum of compound **4i**

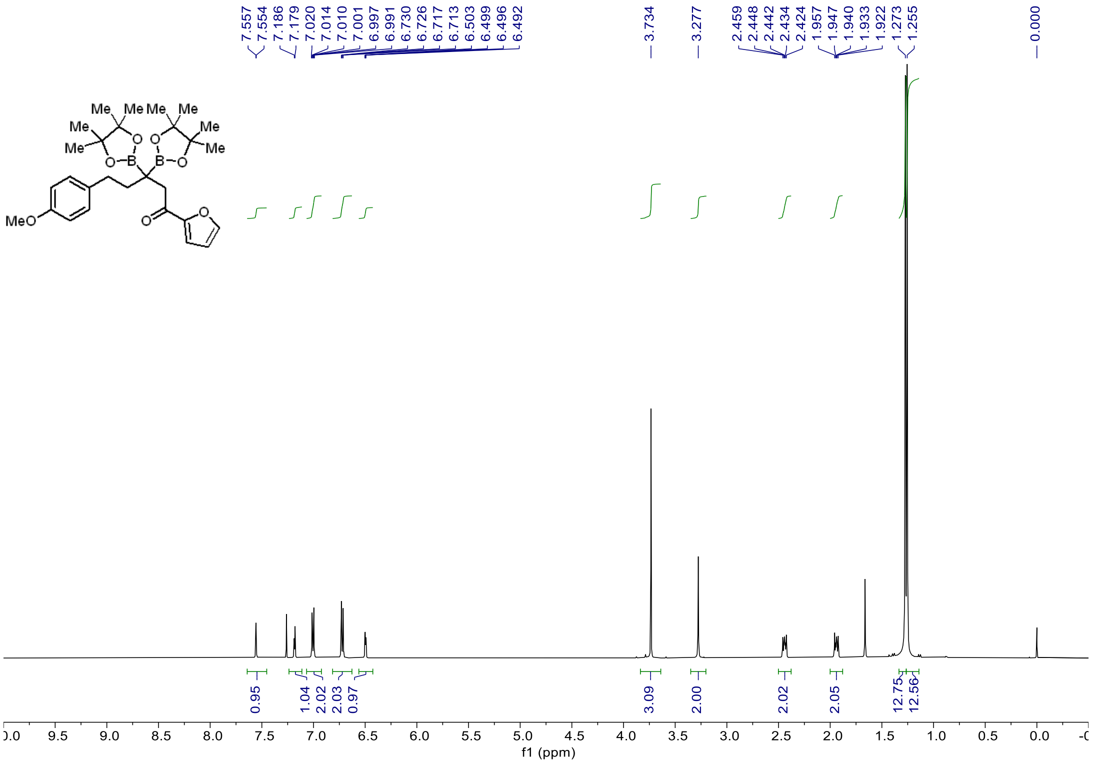

^{1}H NMR (500 MHz, $CDCl_3$) spectrum of compound **4j**

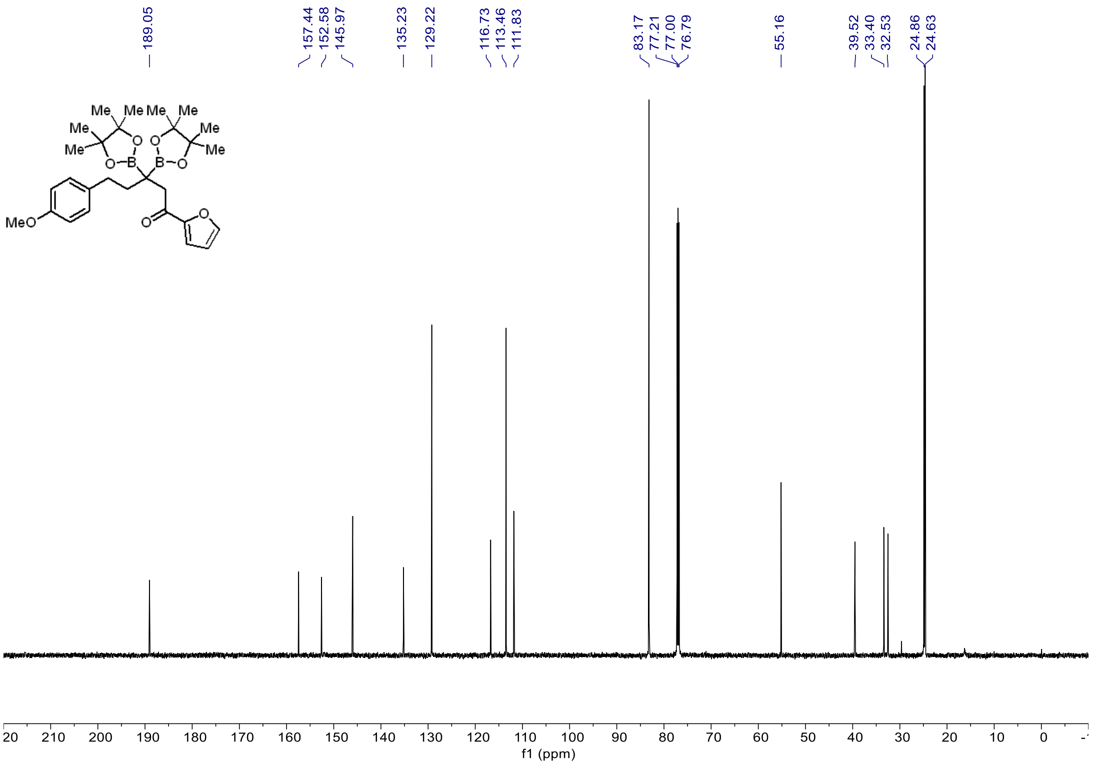

^{13}C NMR (150 MHz, $CDCl_3$) spectrum of compound **4j**

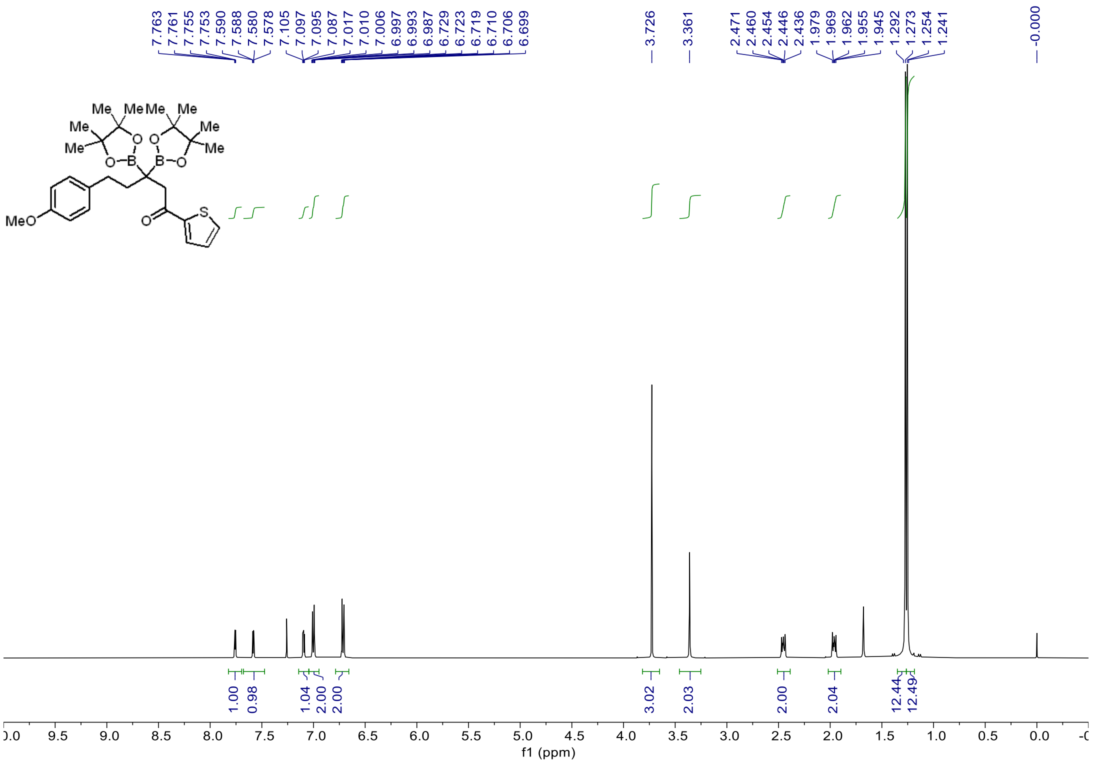

^{1}H NMR (500 MHz, $CDCl_3$) spectrum of compound **4k**

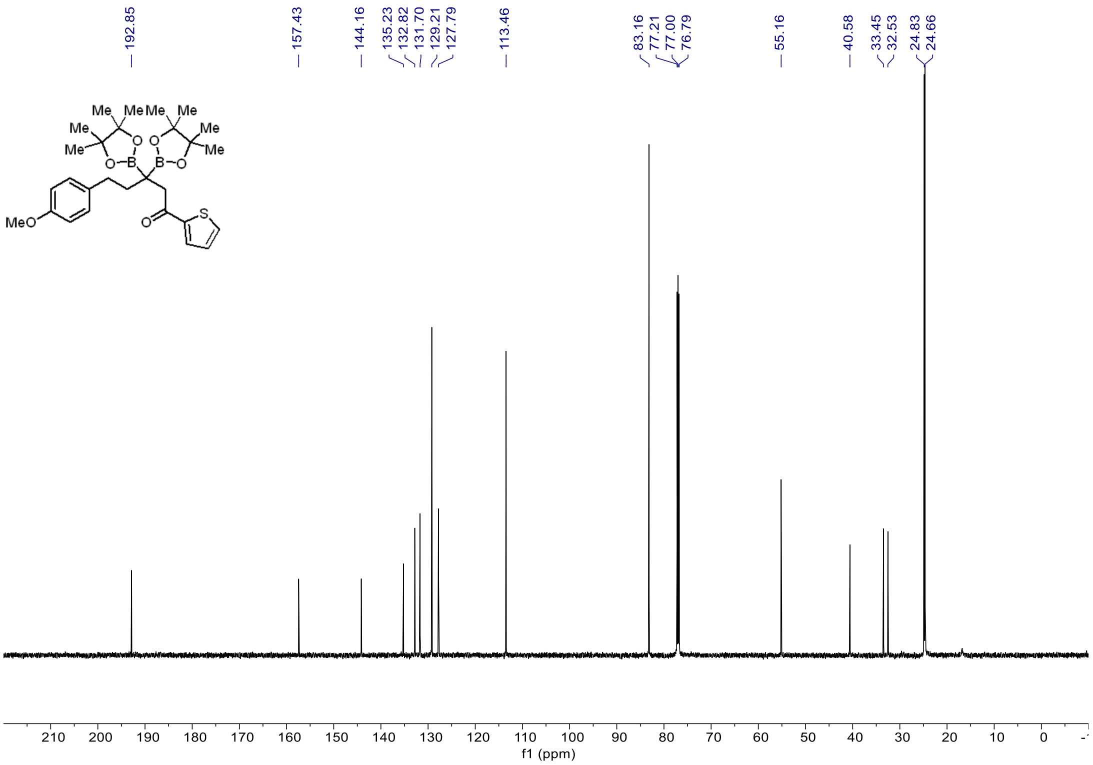

^{13}C NMR (150 MHz, CDCl$_3$) spectrum of compound **4k**

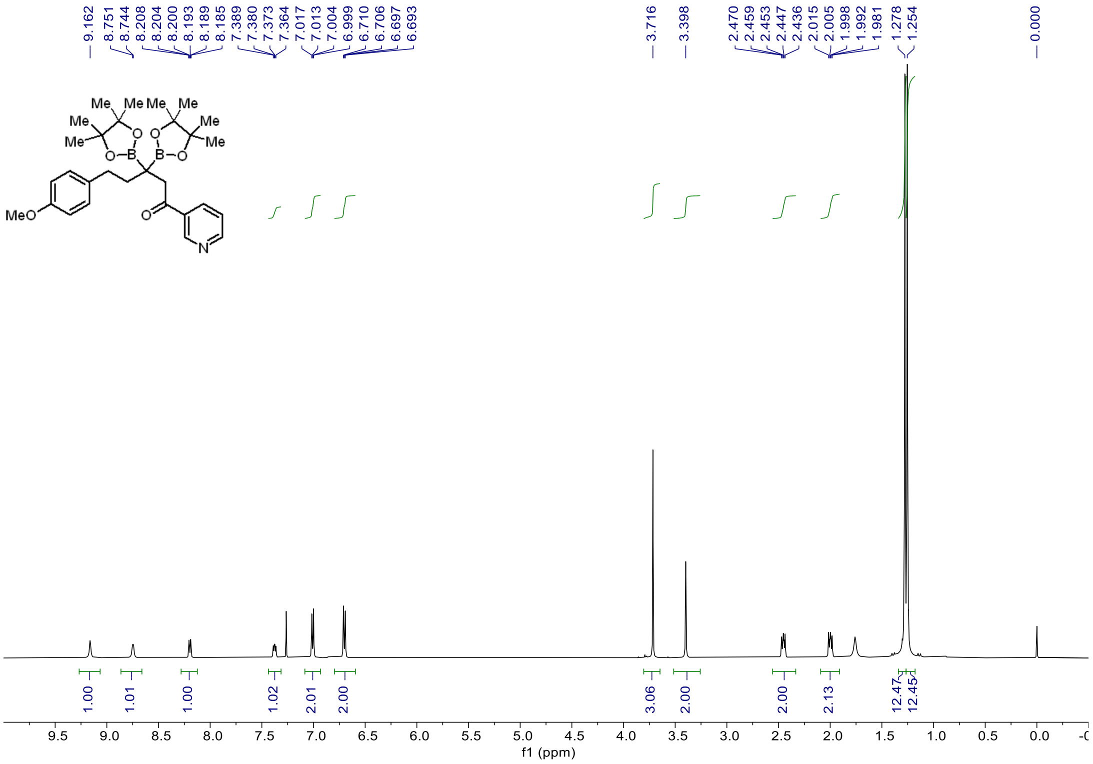

^{1}H NMR (500 MHz, CDCl$_3$) spectrum of compound **4l**

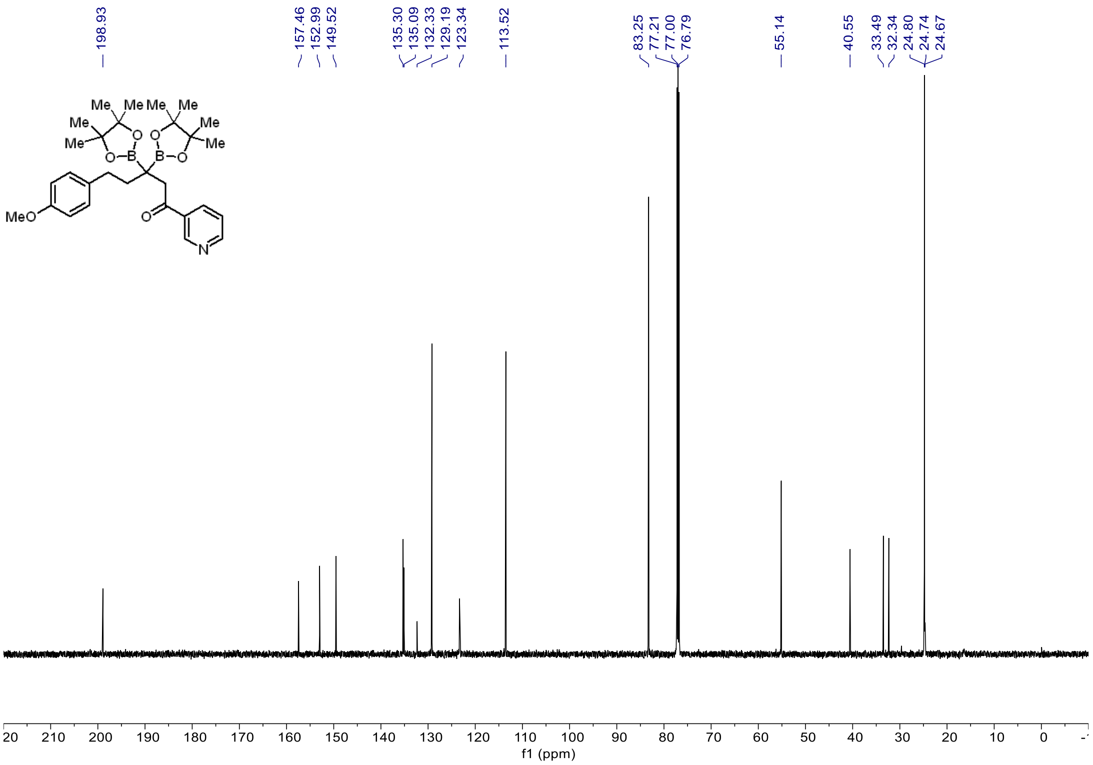

^{13}C NMR (150 MHz, $CDCl_3$) spectrum of compound **4l**

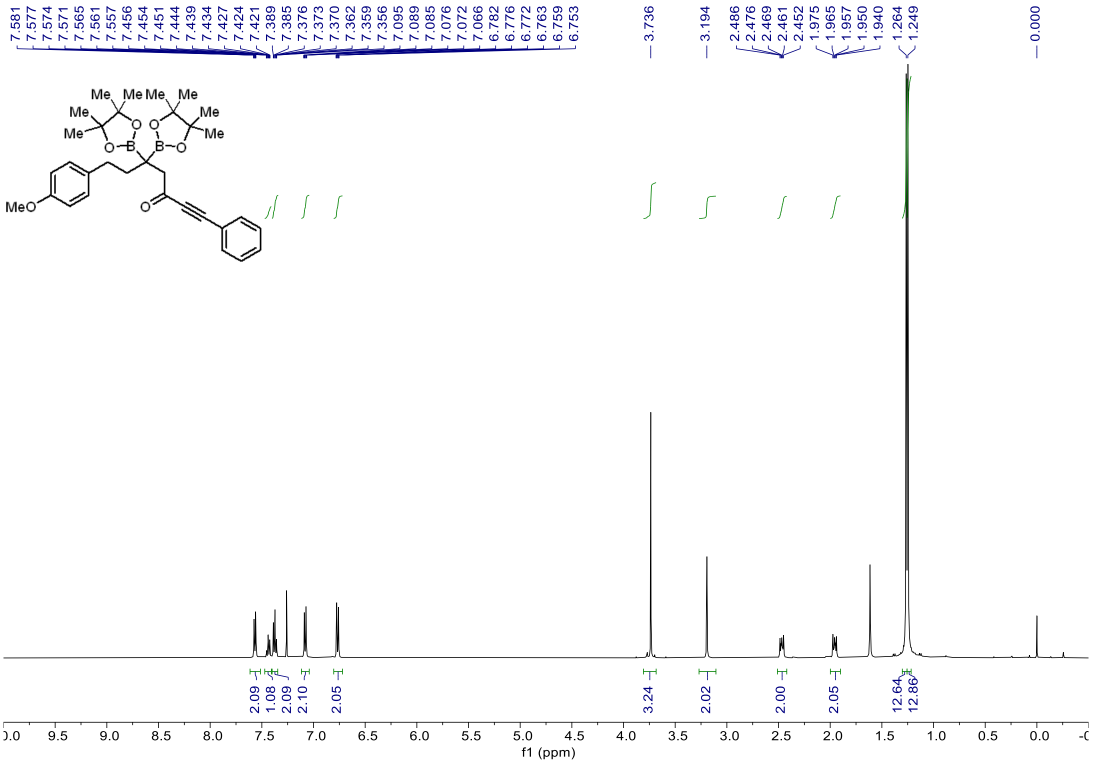

^{1}H NMR (500 MHz, $CDCl_3$) spectrum of compound **4m**

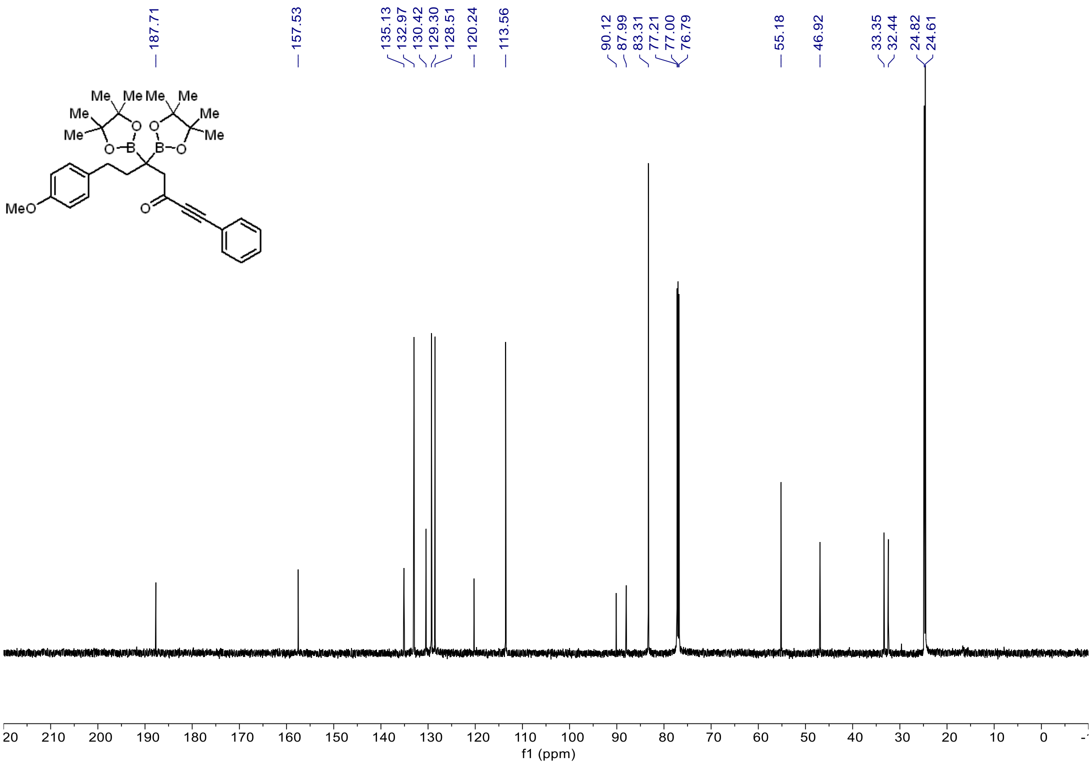

^{13}C NMR (150 MHz, $CDCl_3$) spectrum of compound **4m**

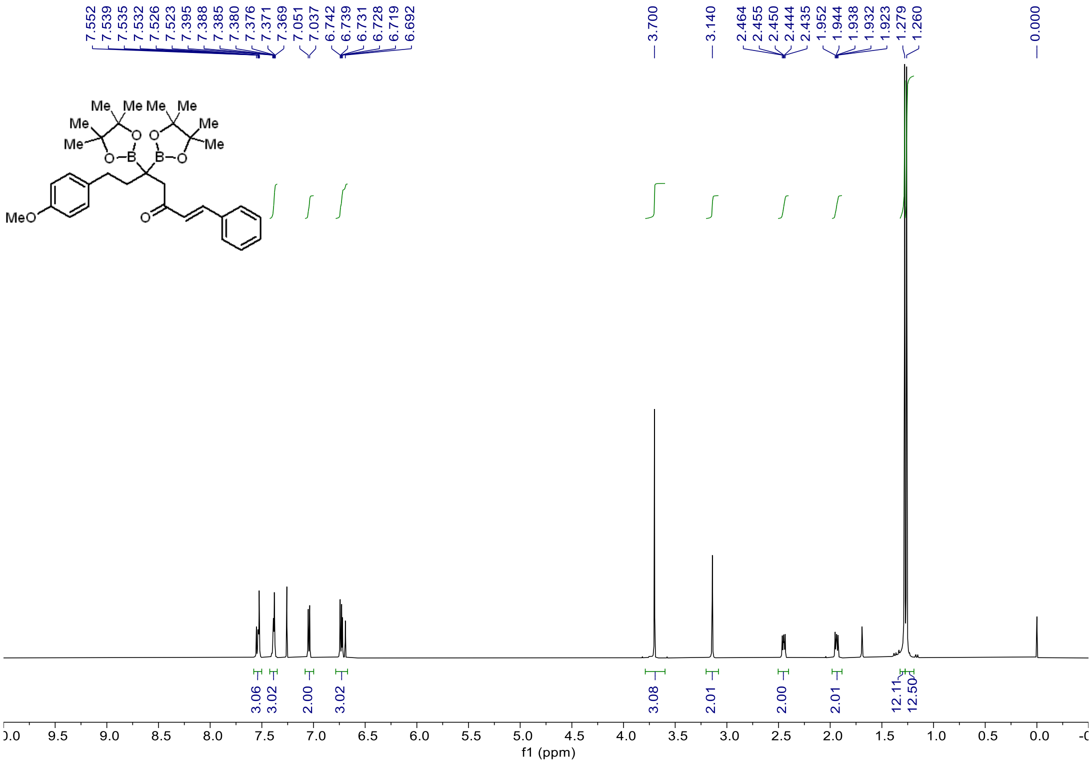

^{1}H NMR (600 MHz, $CDCl_3$) spectrum of compound **4n**

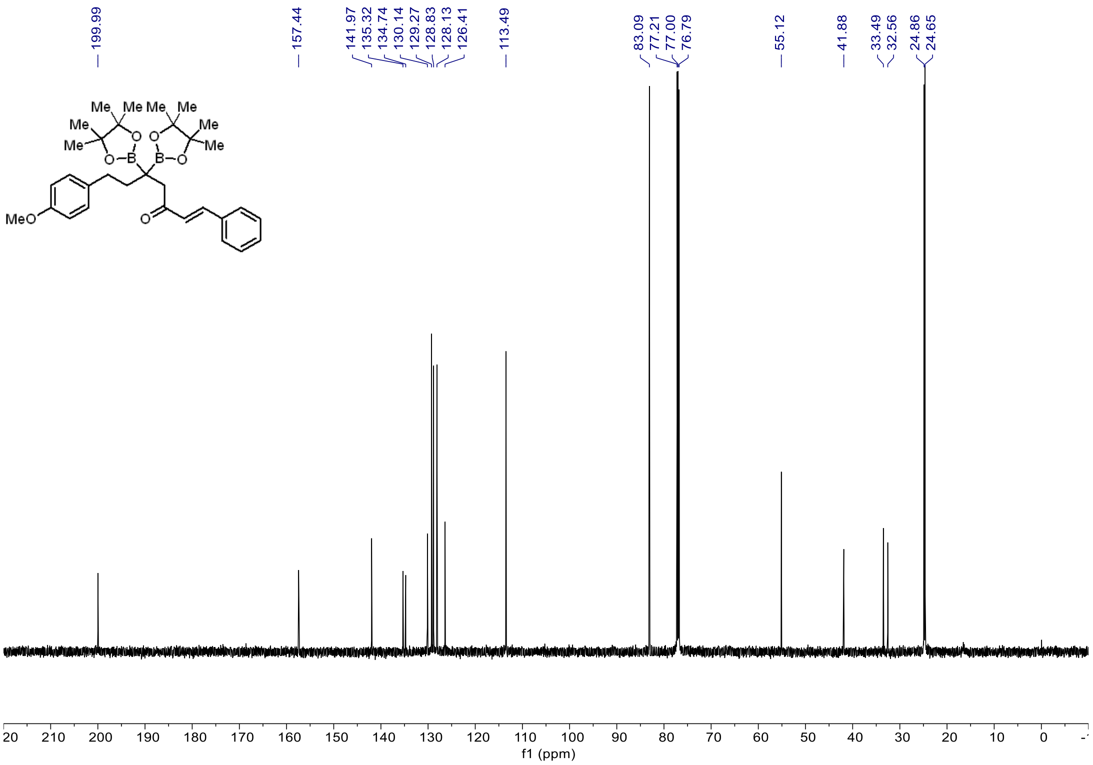

^{13}C NMR (150 MHz, $CDCl_3$) spectrum of compound **4n**

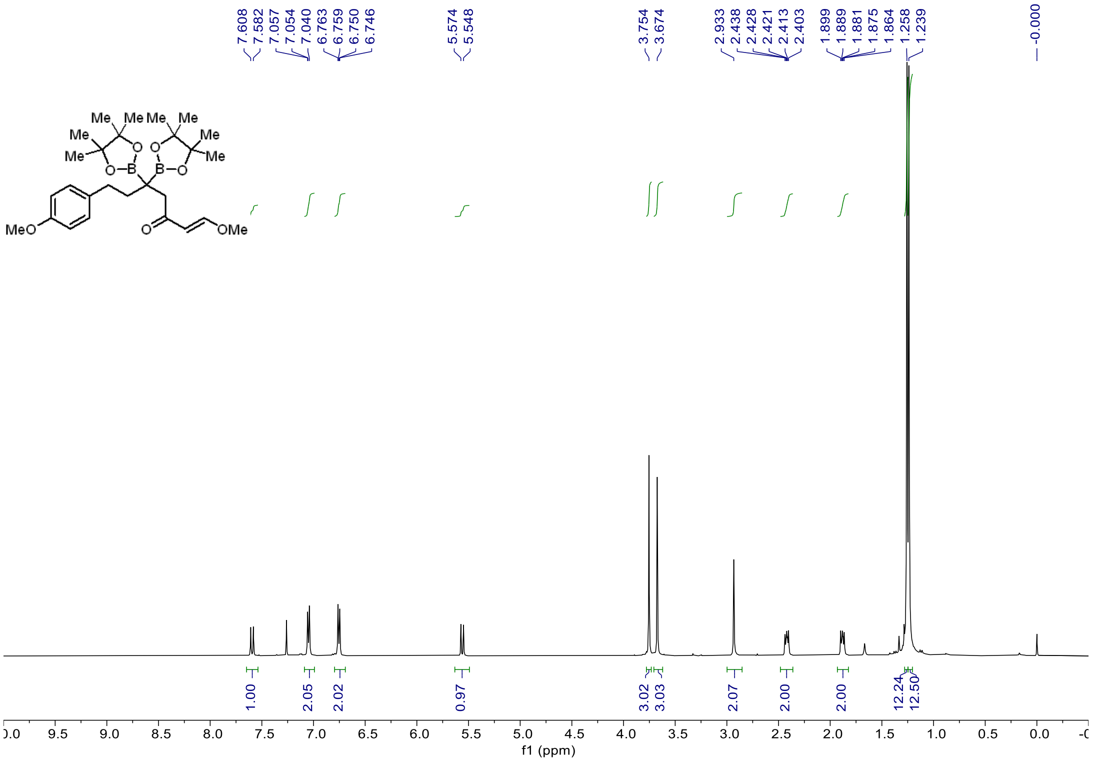

^{1}H NMR (500 MHz, $CDCl_3$) spectrum of compound **4o**

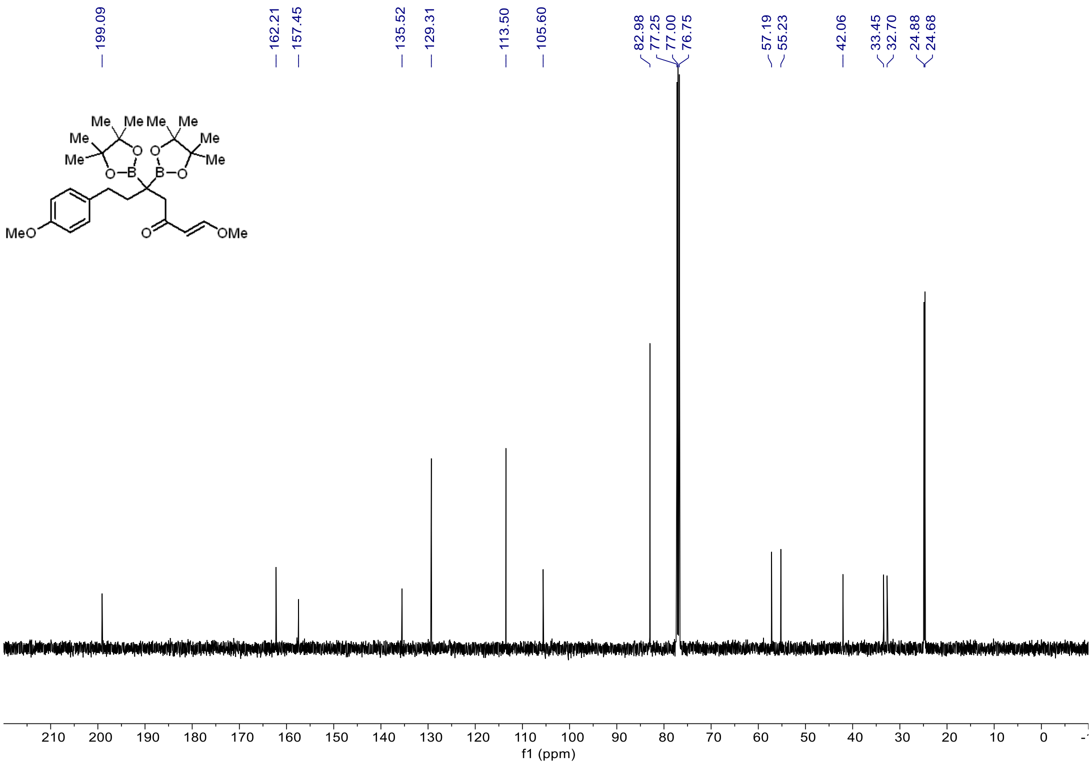

^{13}C NMR (125 MHz, $CDCl_3$) spectrum of compound **4o**

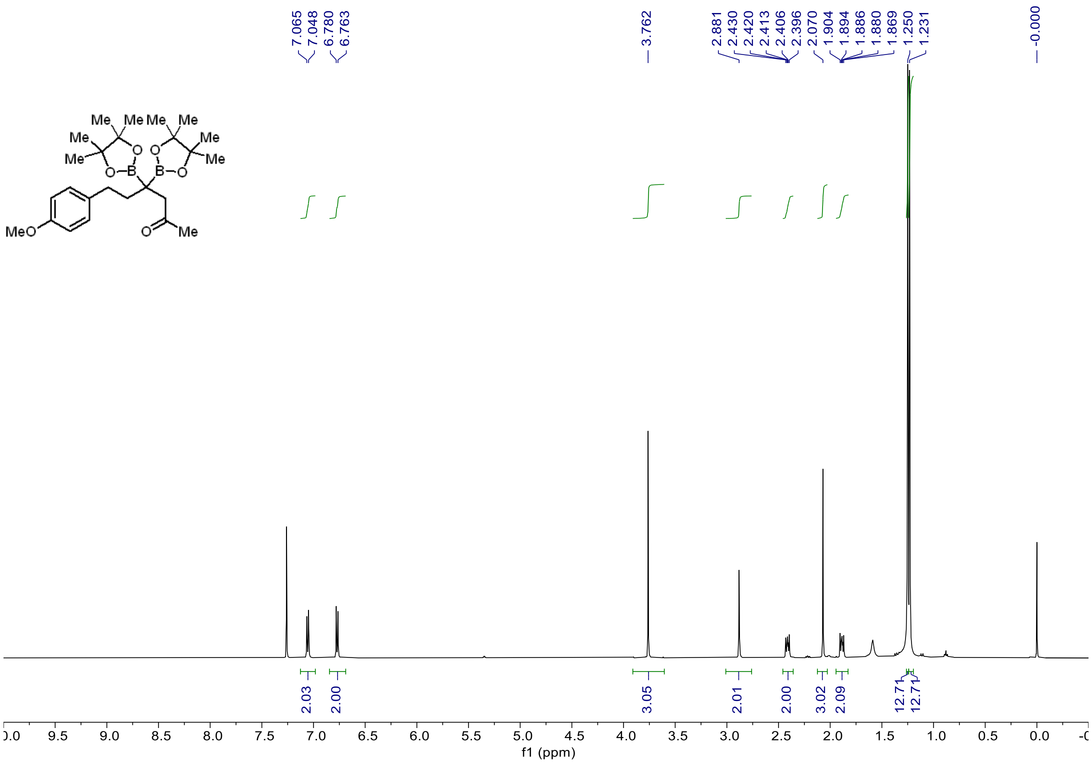

^{1}H NMR (500 MHz, $CDCl_3$) spectrum of compound **4p**

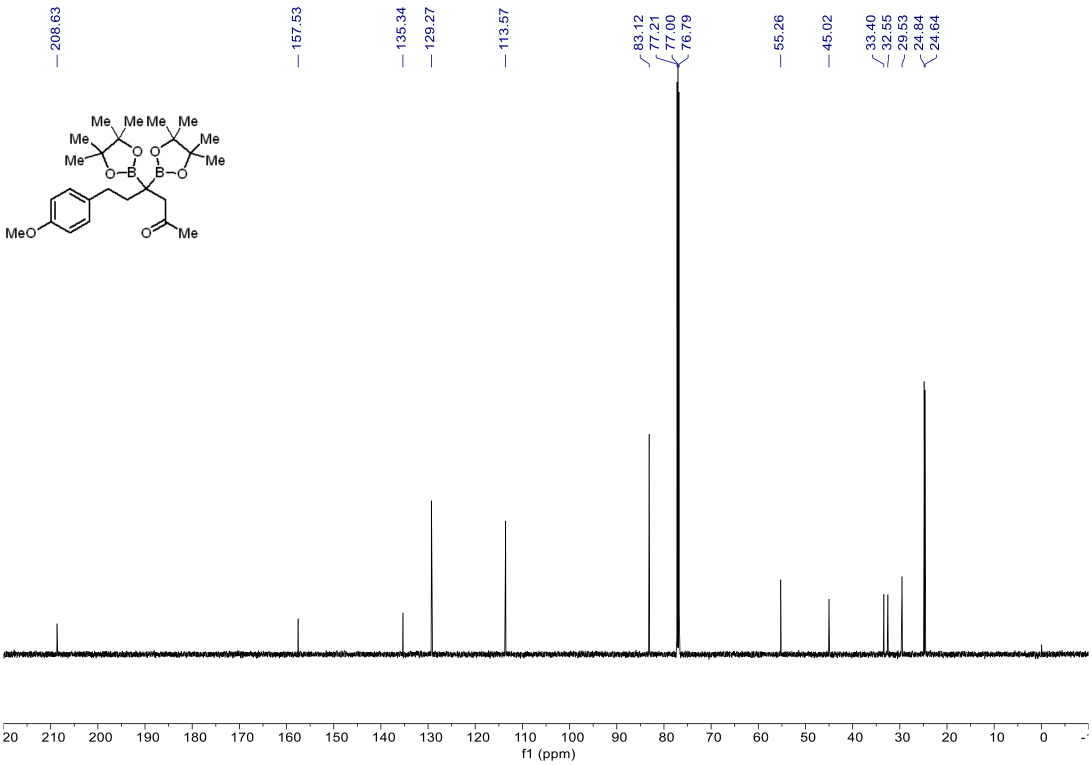

^{13}C NMR (150 MHz, $CDCl_3$) spectrum of compound **4p**

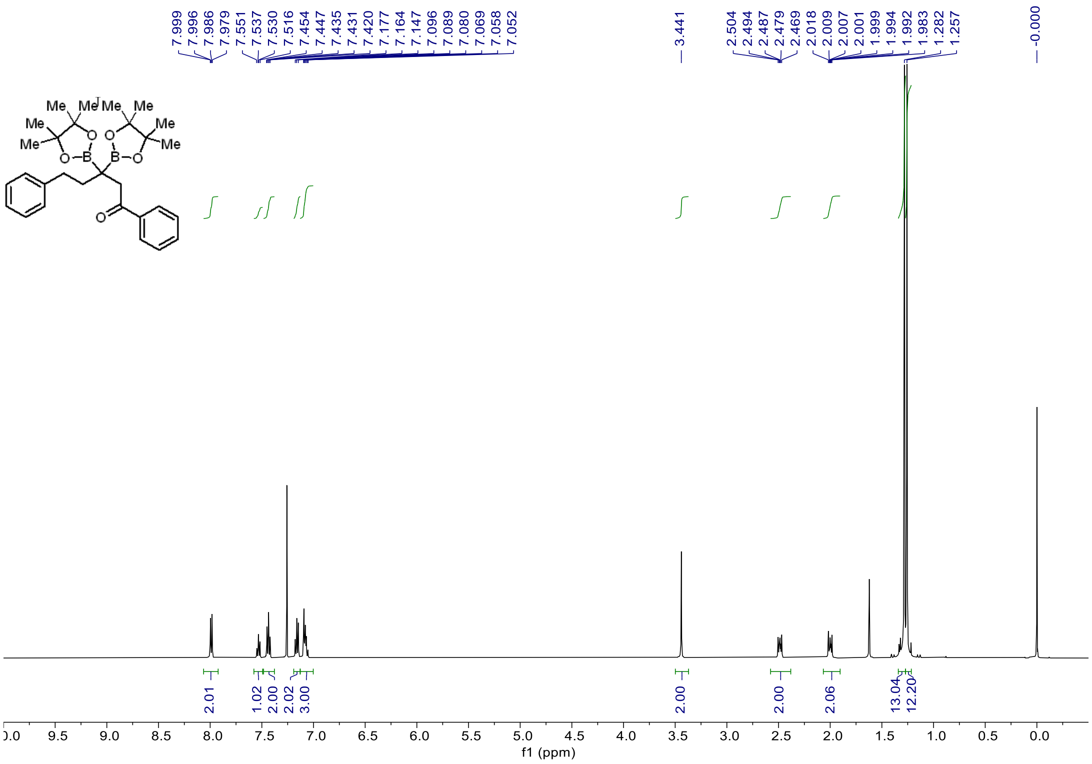

^{1}H NMR (500 MHz, $CDCl_3$) spectrum of compound **4a**

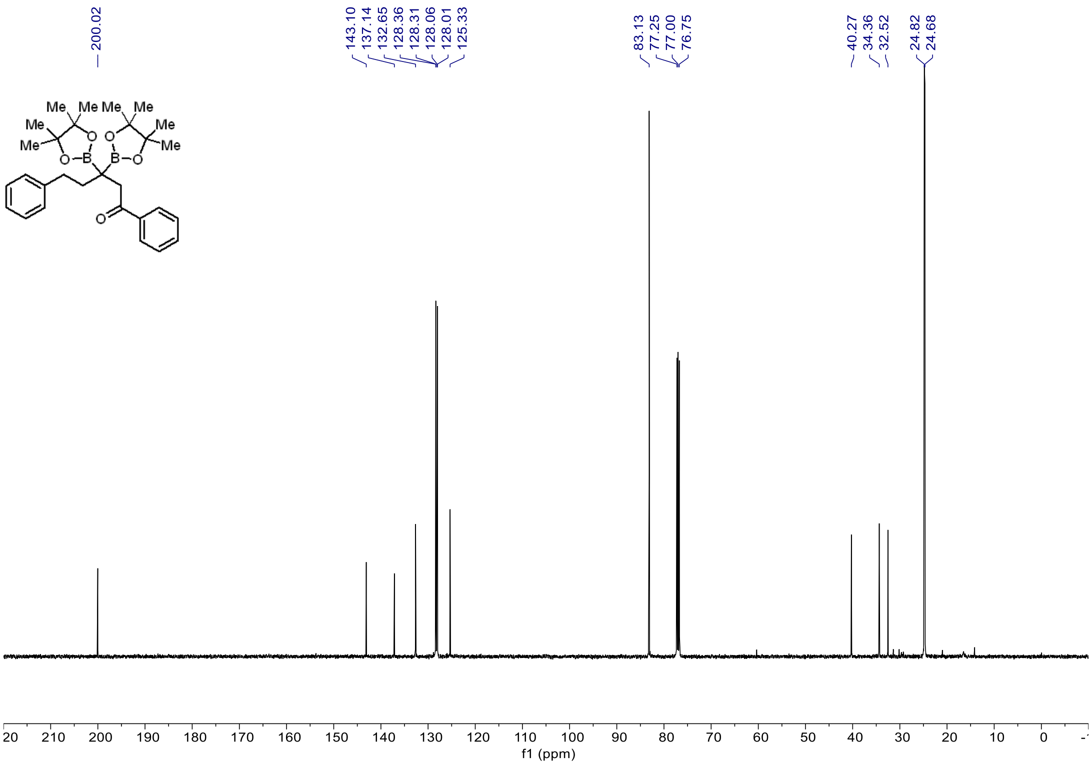

^{13}C NMR (125 MHz, $CDCl_3$) spectrum of compound **4a**

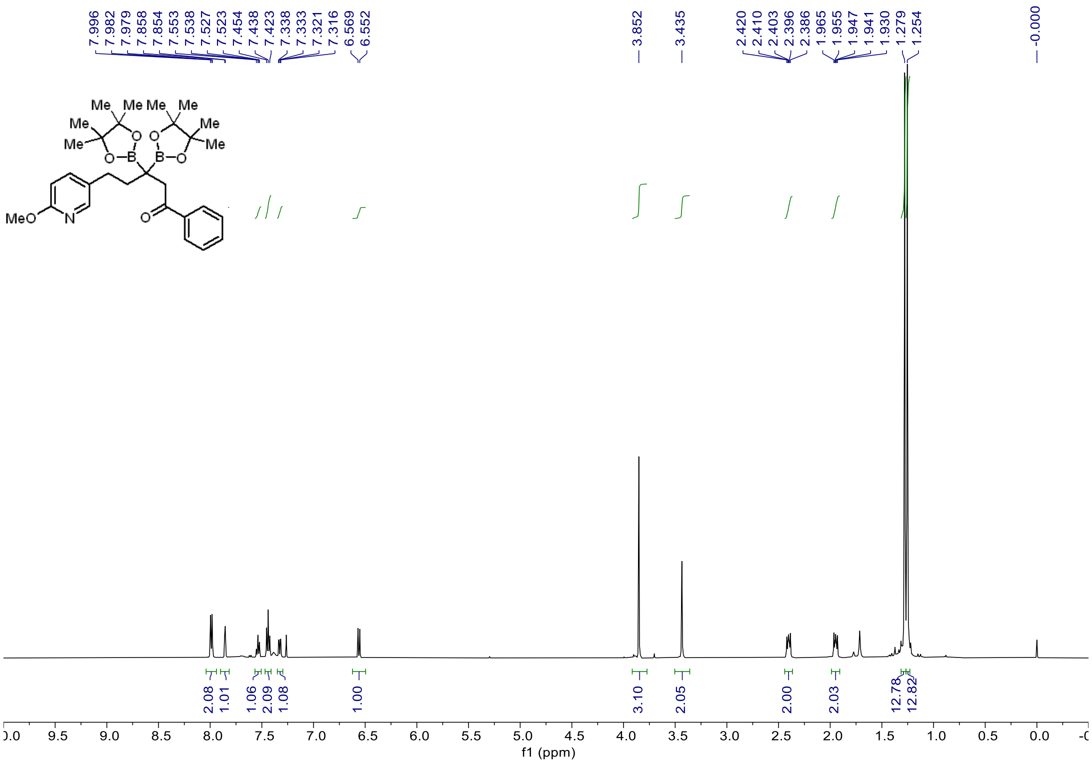

^{1}H NMR (500 MHz, $CDCl_3$) spectrum of compound **4q**

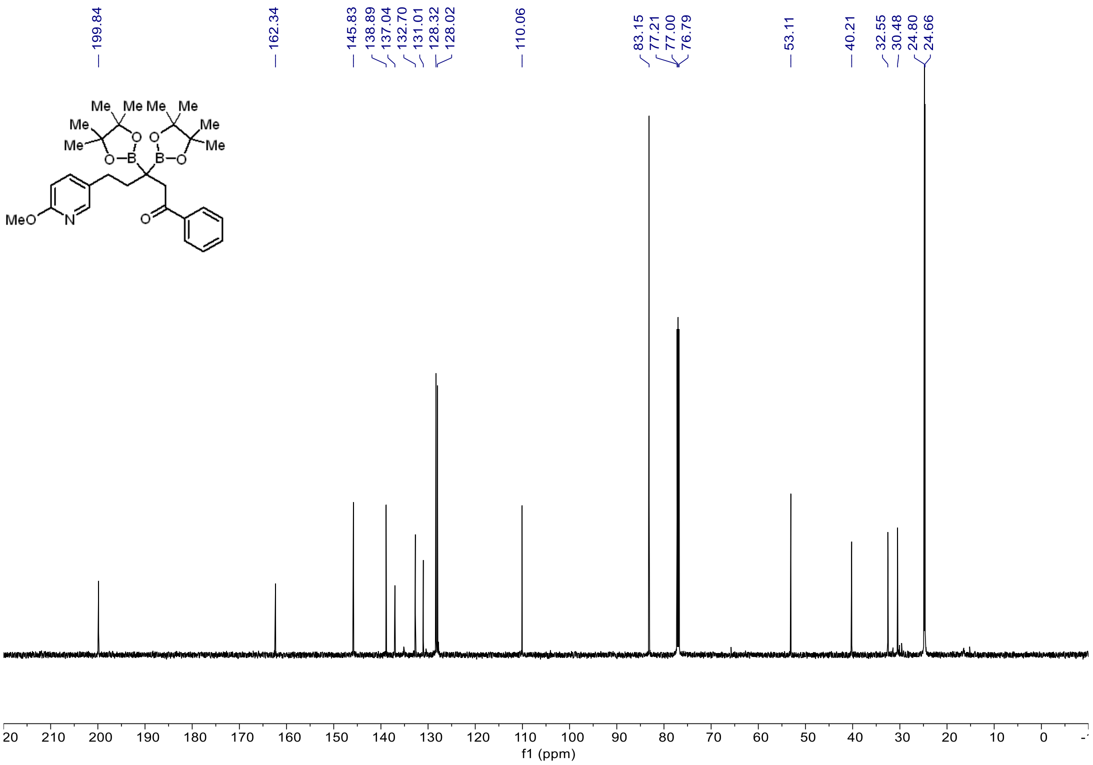

^{13}C NMR (150 MHz, $CDCl_3$) spectrum of compound **4q**

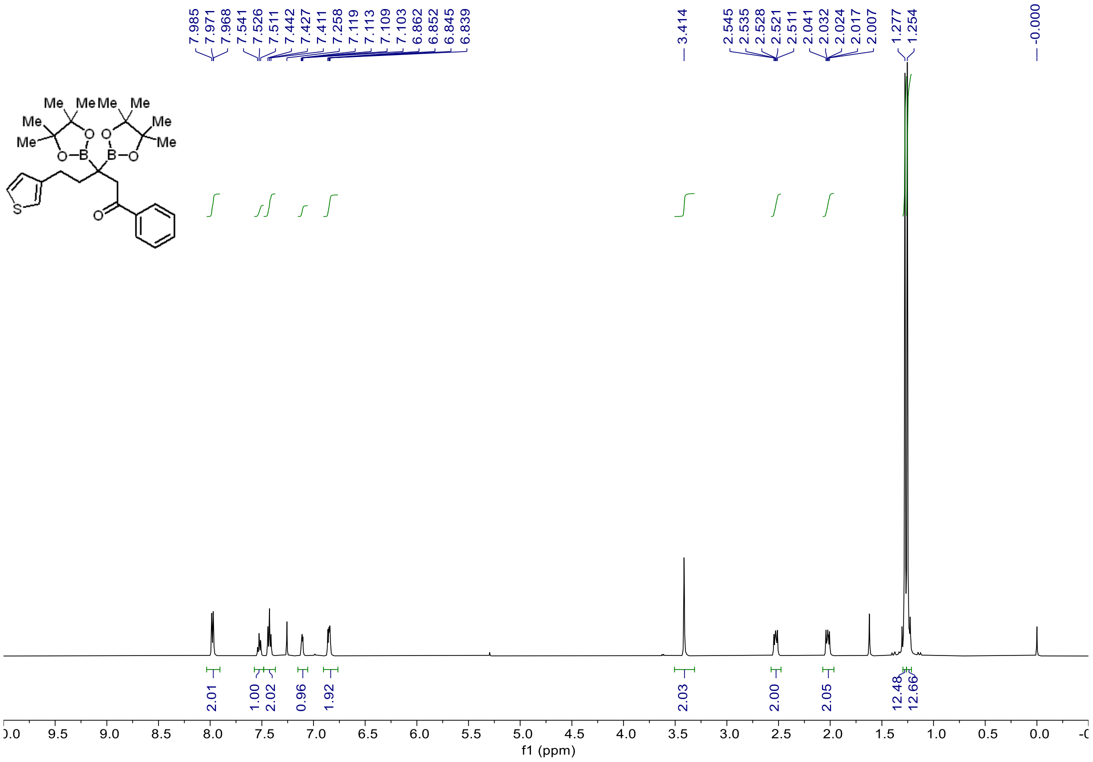

^{1}H NMR (600 MHz, $CDCl_3$) spectrum of compound **4r**

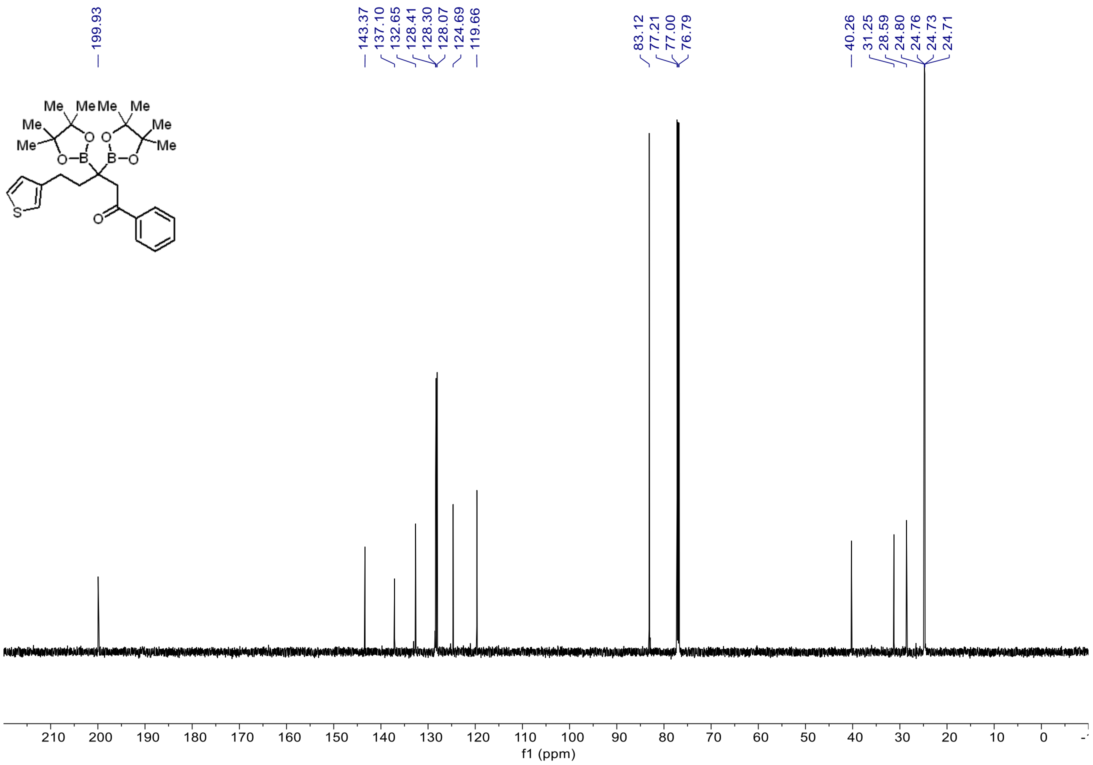

^{13}C NMR (150 MHz, CDCl$_3$) spectrum of compound **4r**

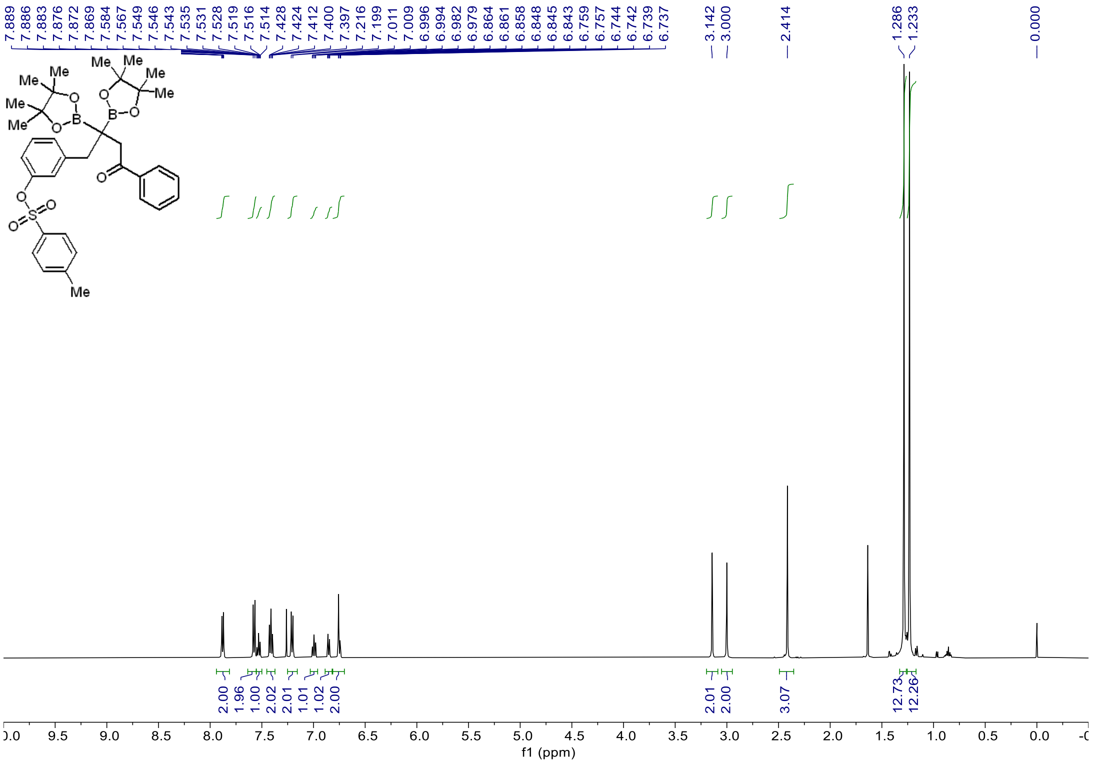

^{1}H NMR (500 MHz, CDCl$_3$) spectrum of compound **4s**

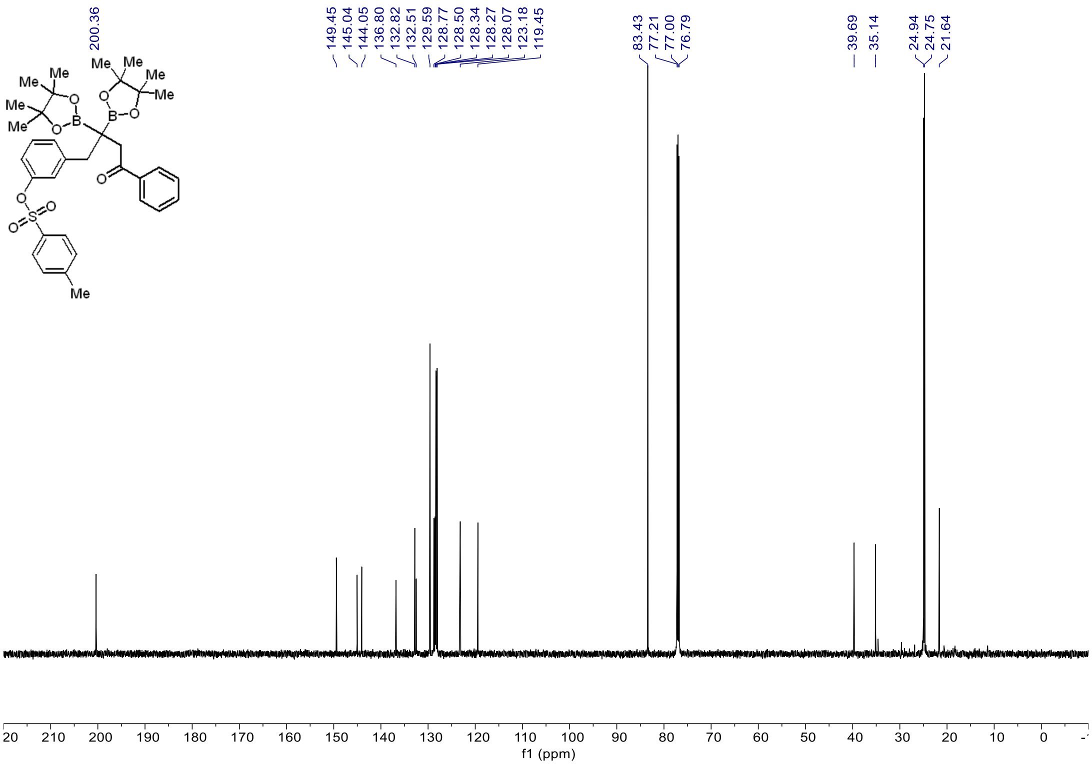

^{13}C NMR (150 MHz, $CDCl_3$) spectrum of compound **4s**

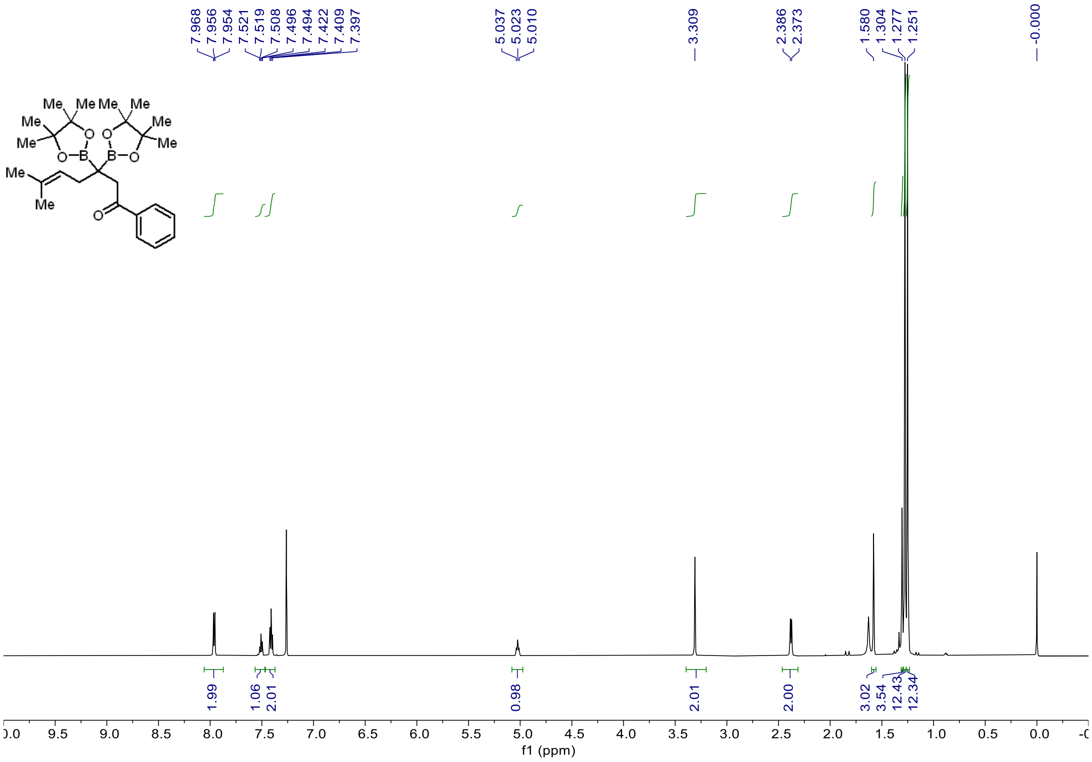

^{1}H NMR (600 MHz, $CDCl_3$) spectrum of compound **4t**

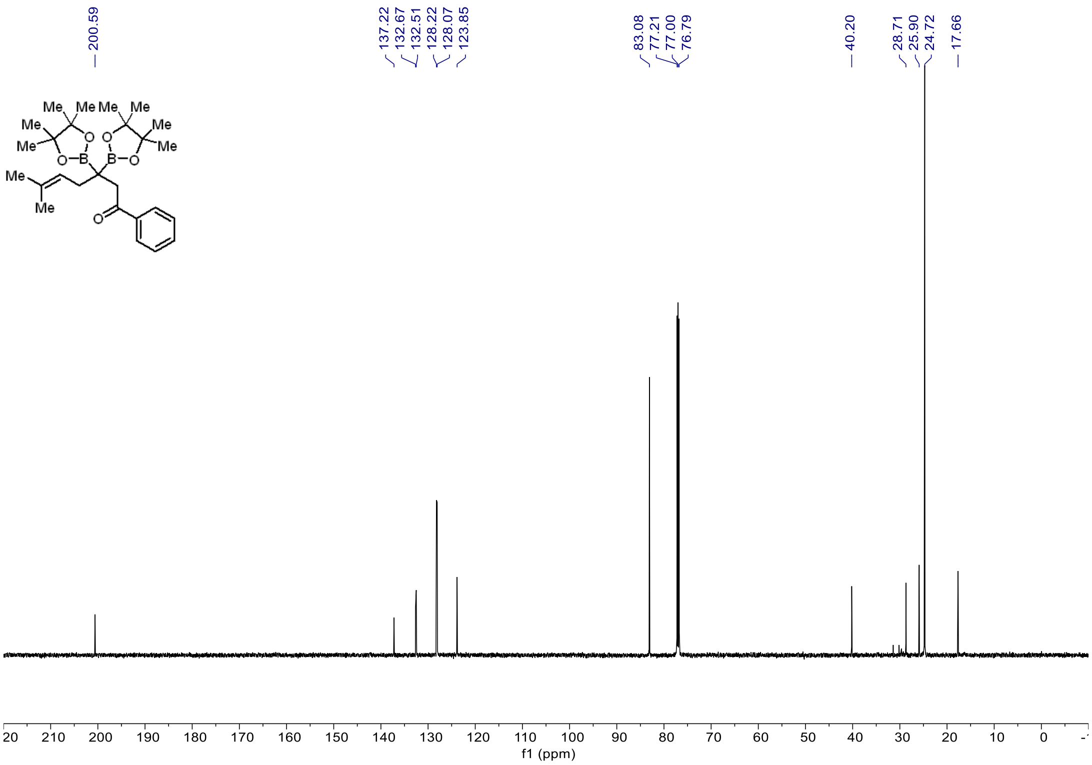

^{13}C NMR (150 MHz, $CDCl_3$) spectrum of compound **4t**

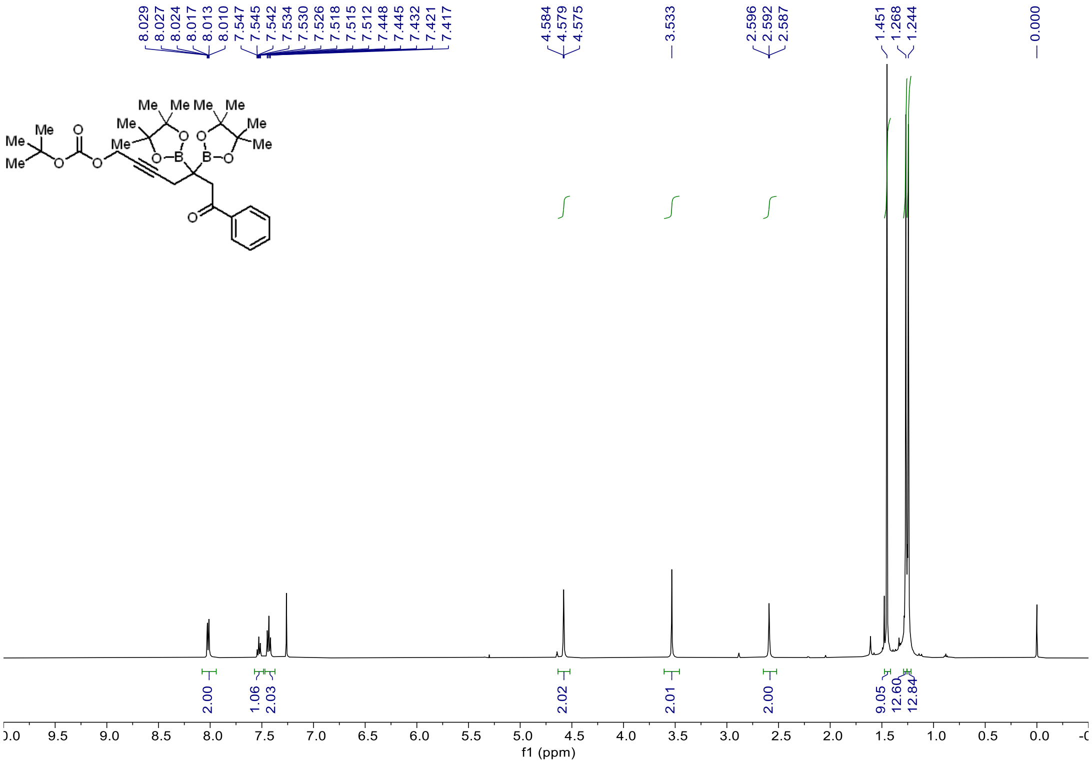

^{1}H NMR (500 MHz, $CDCl_3$) spectrum of compound **4u**

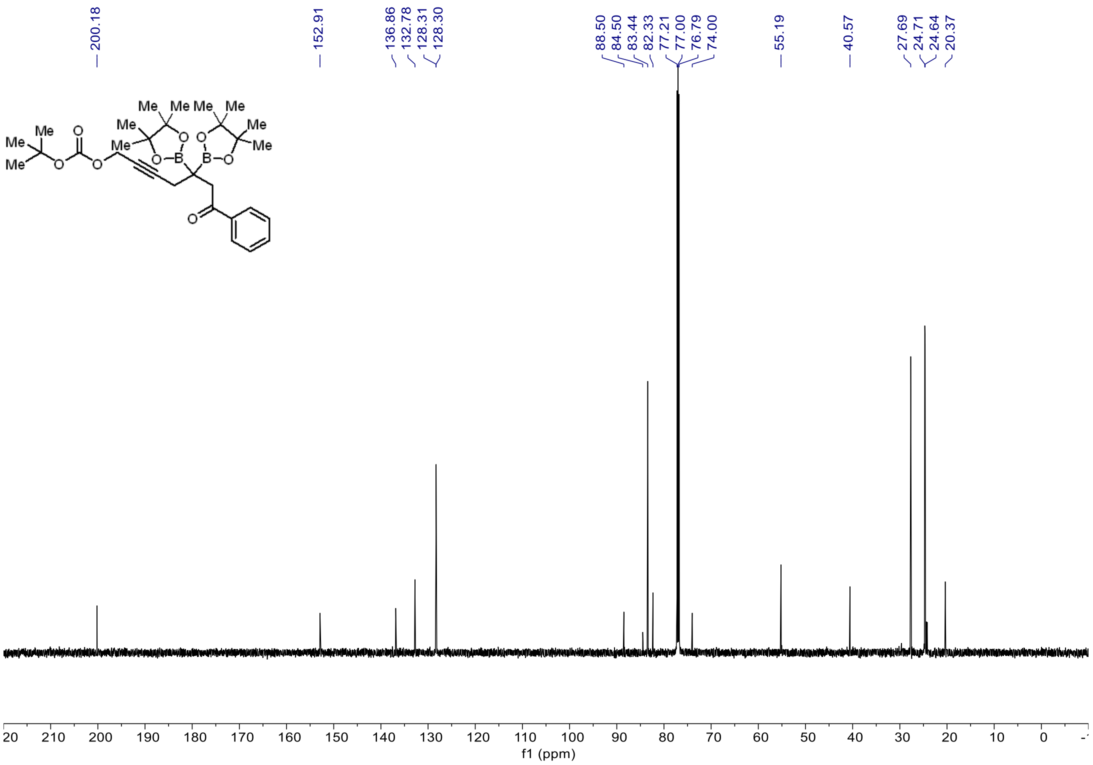

^{13}C NMR (150 MHz, $CDCl_3$) spectrum of compound **4u**

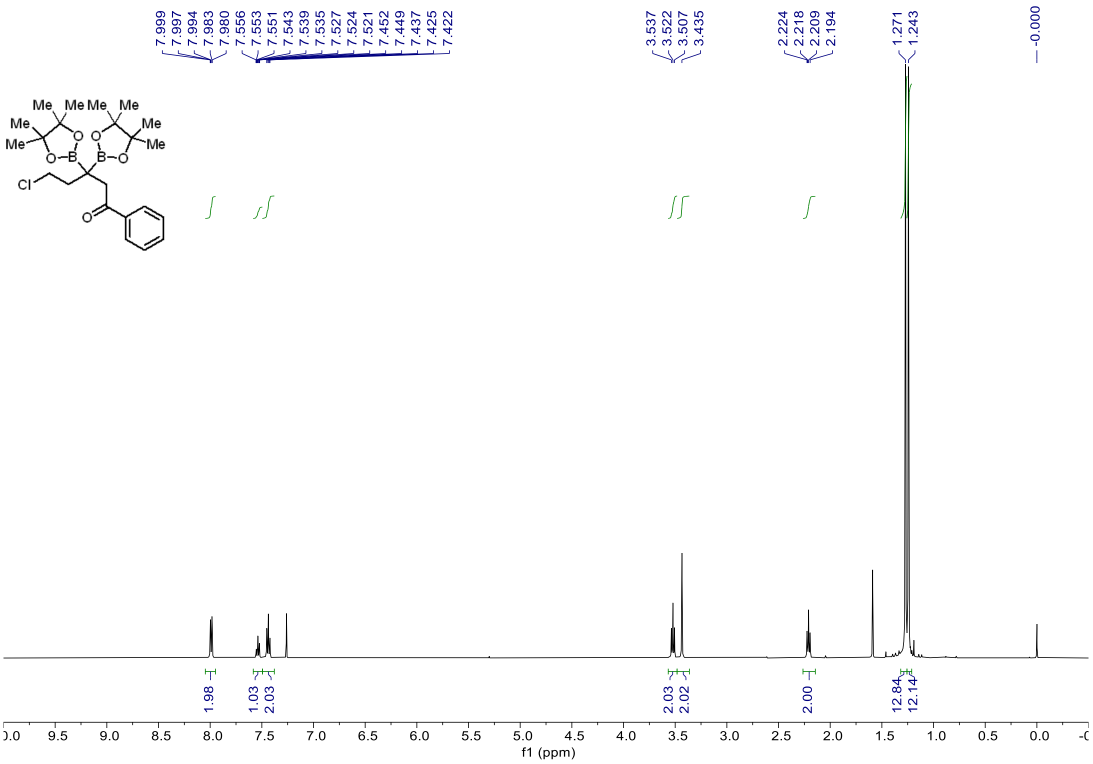

^{1}H NMR (500 MHz, $CDCl_3$) spectrum of compound **4v**

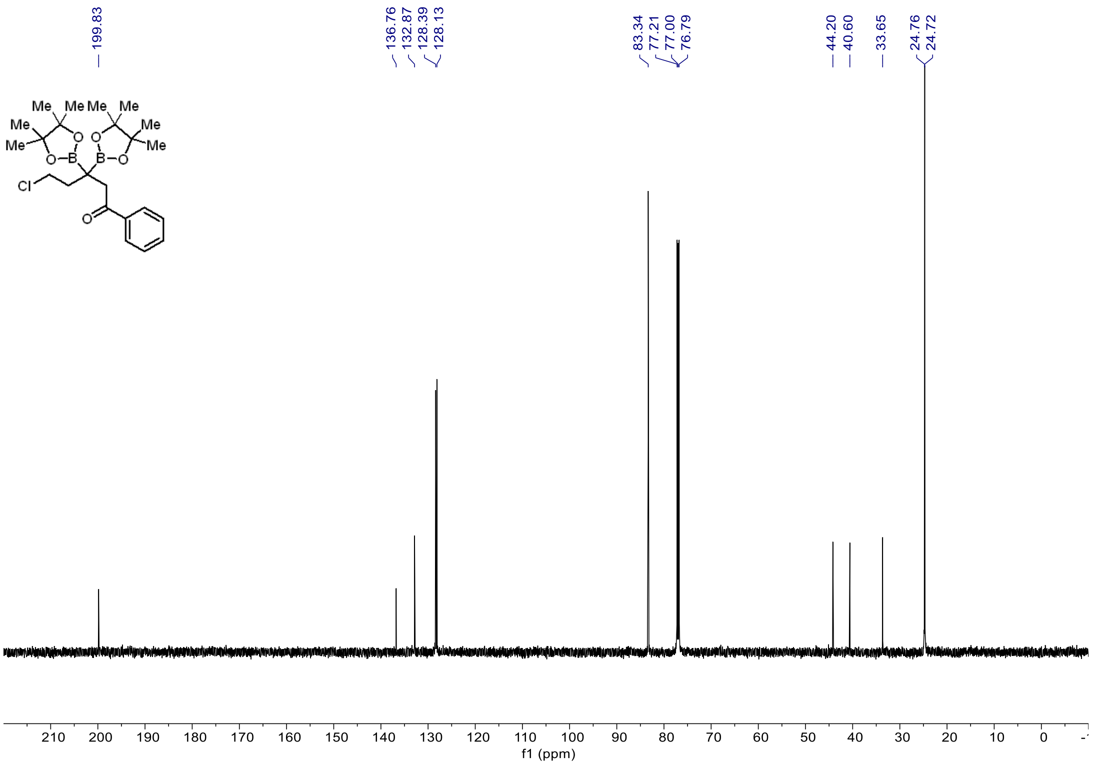

^{13}C NMR (150 MHz, CDCl$_3$) spectrum of compound **4v**

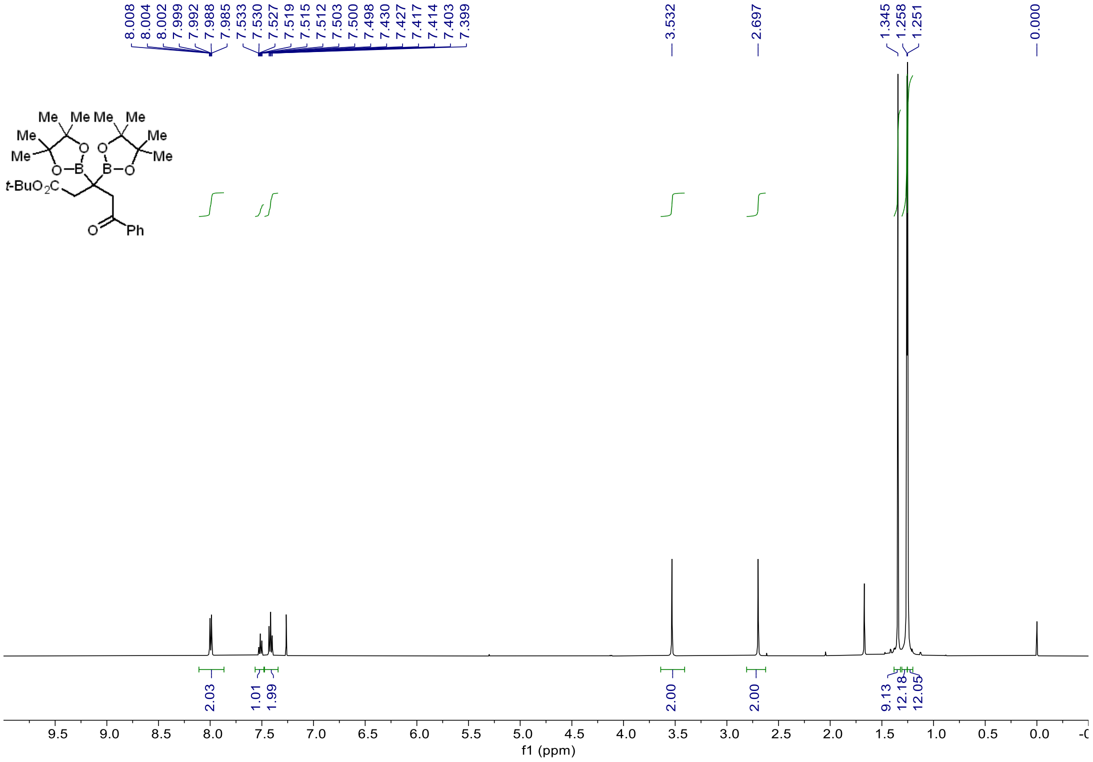

^{1}H NMR (500 MHz, CDCl$_3$) spectrum of compound **4w**

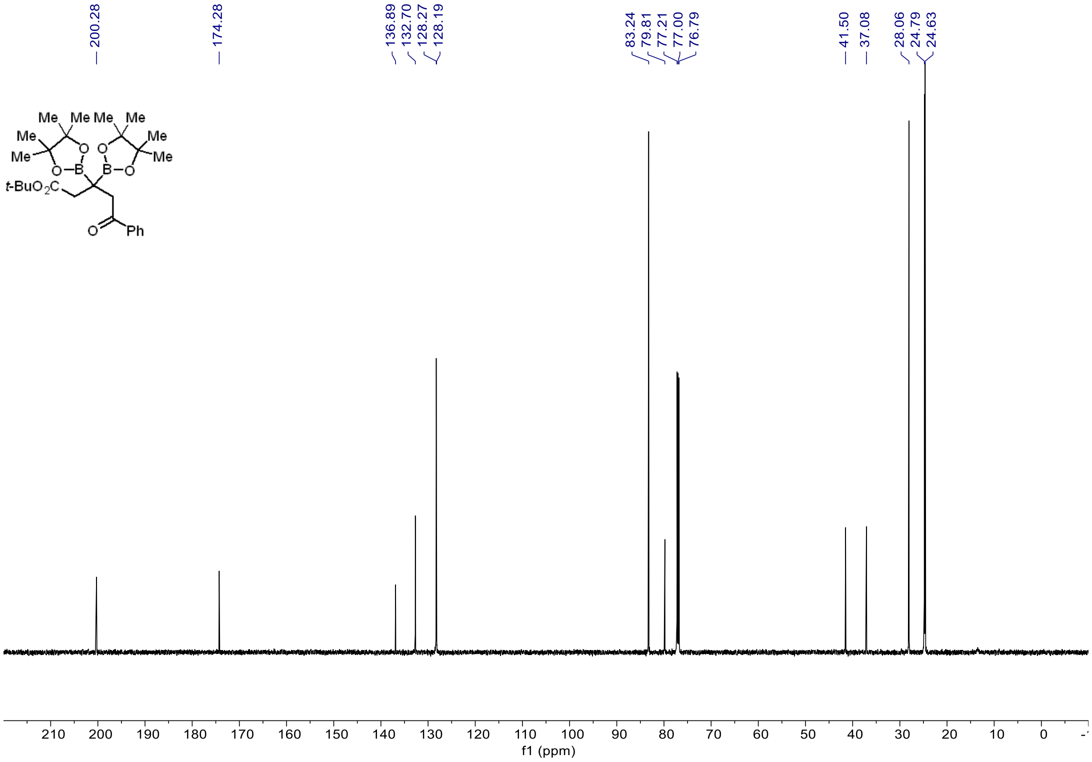

^{13}C NMR (150 MHz, CDCl$_3$) spectrum of compound **4w**

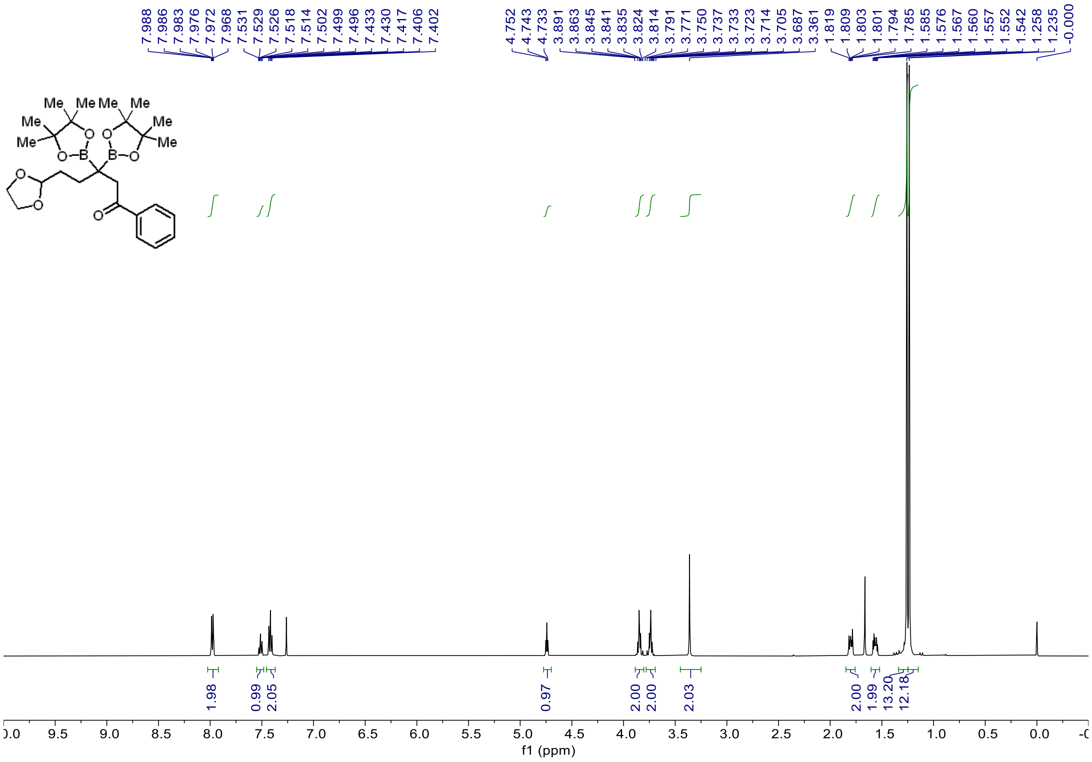

^{1}H NMR (500 MHz, CDCl$_3$) spectrum of compound **4x**

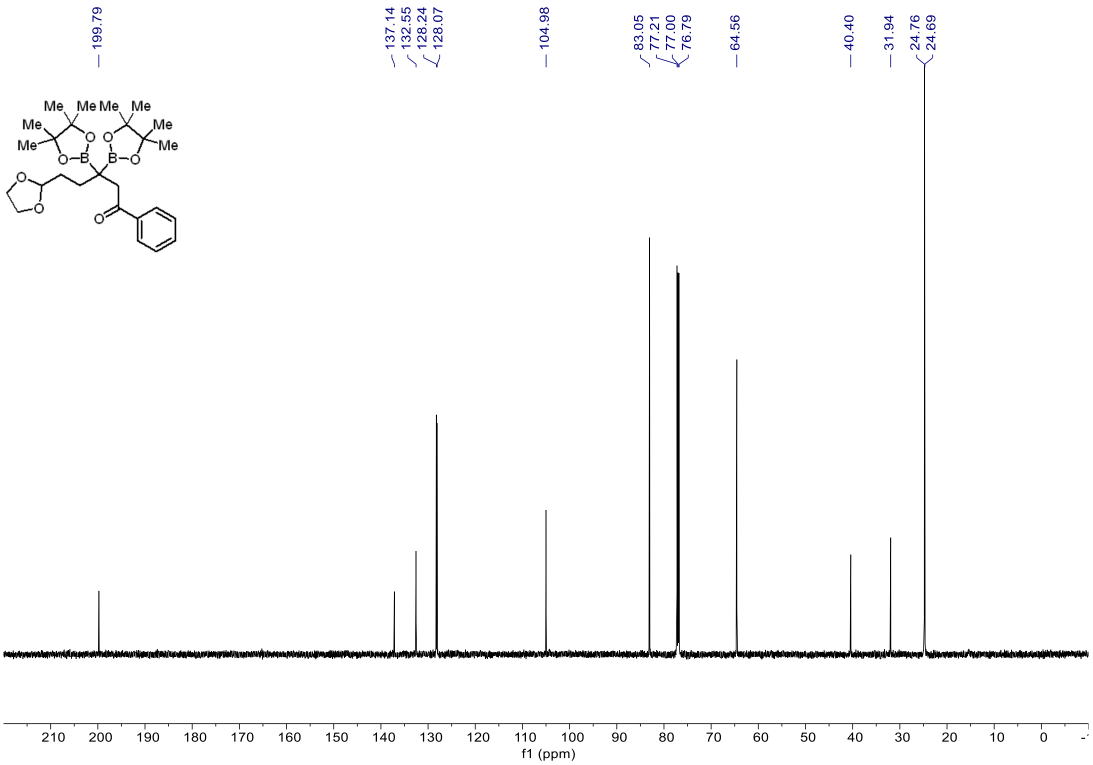

^{13}C NMR (150 MHz, $CDCl_3$) spectrum of compound **4x**

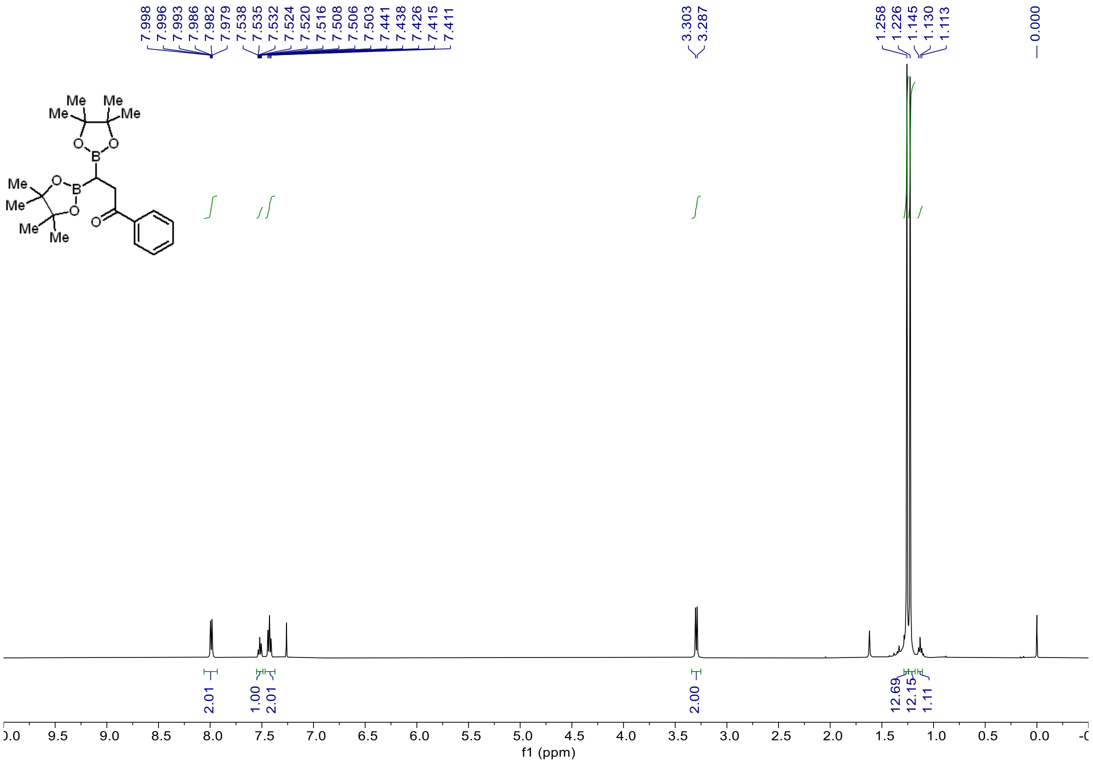

^{1}H NMR (500 MHz, $CDCl_3$) spectrum of compound **4y**

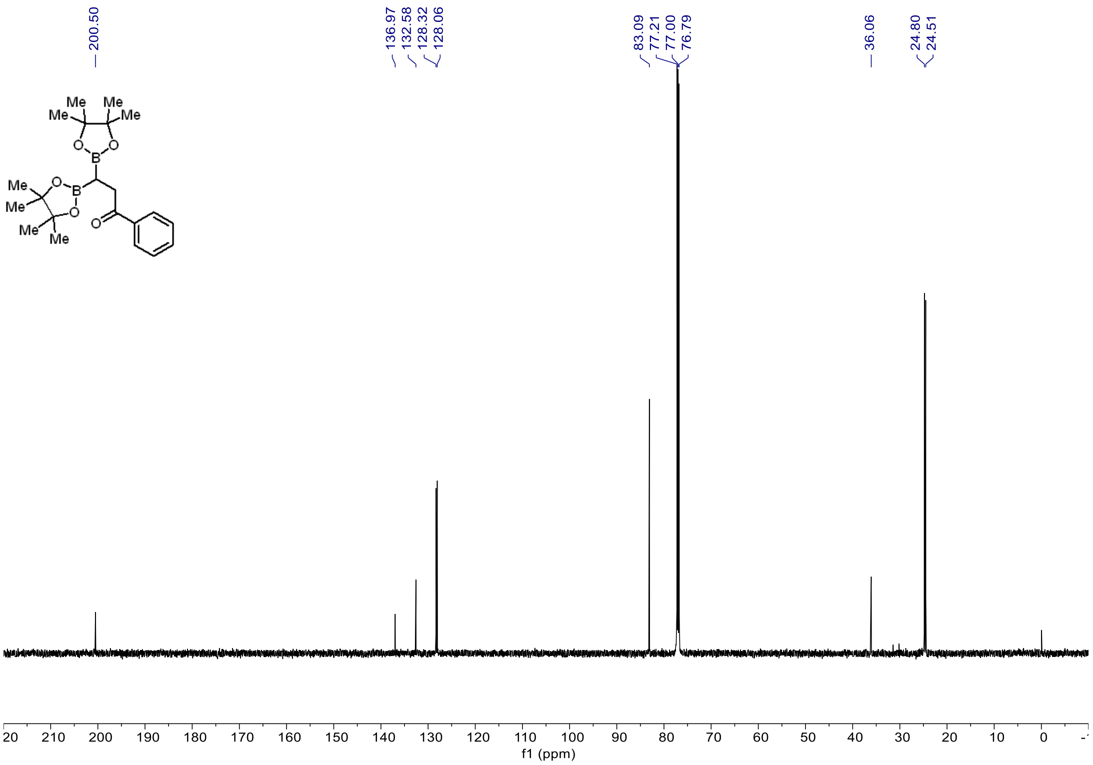

^{13}C NMR (150 MHz, $CDCl_3$) spectrum of compound **4y**

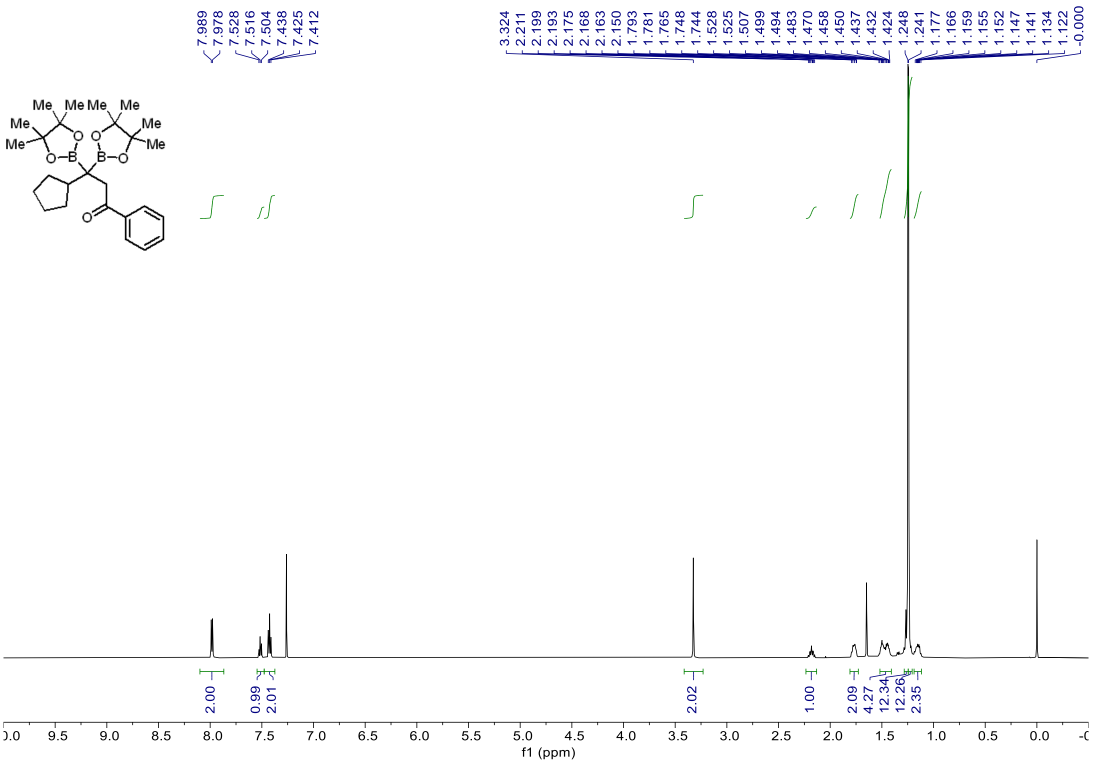

^{1}H NMR (600 MHz, $CDCl_3$) spectrum of compound **4z**

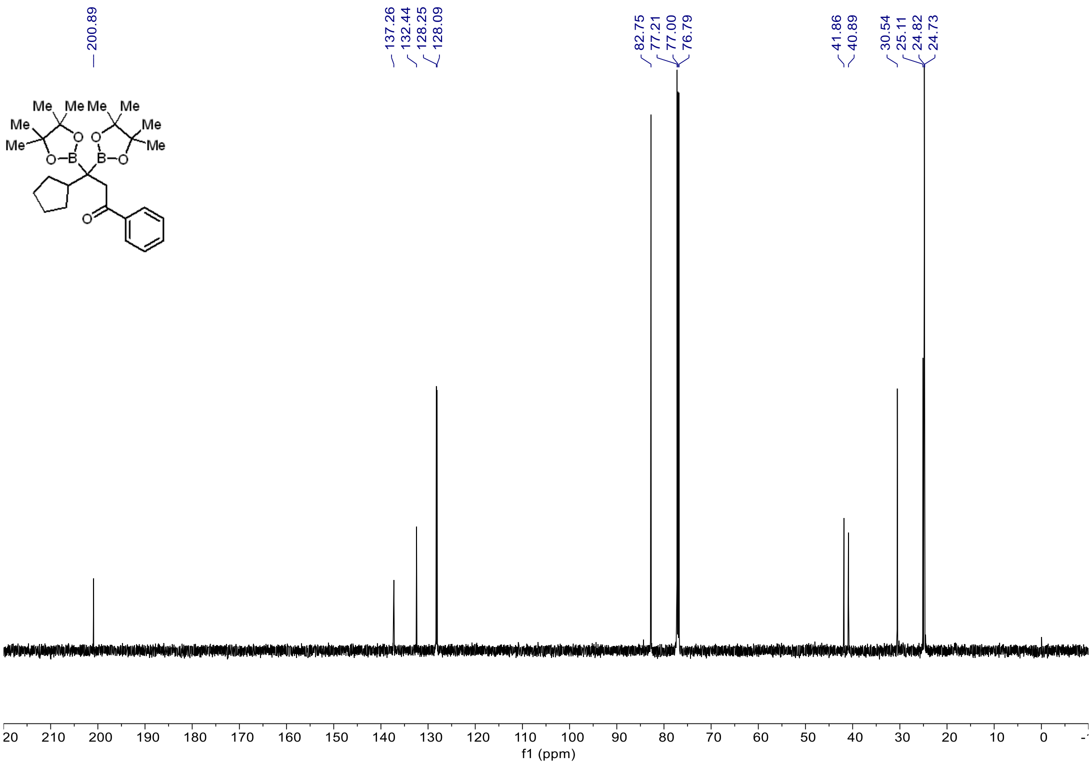

^{13}C NMR (150 MHz, $CDCl_3$) spectrum of compound **4z**

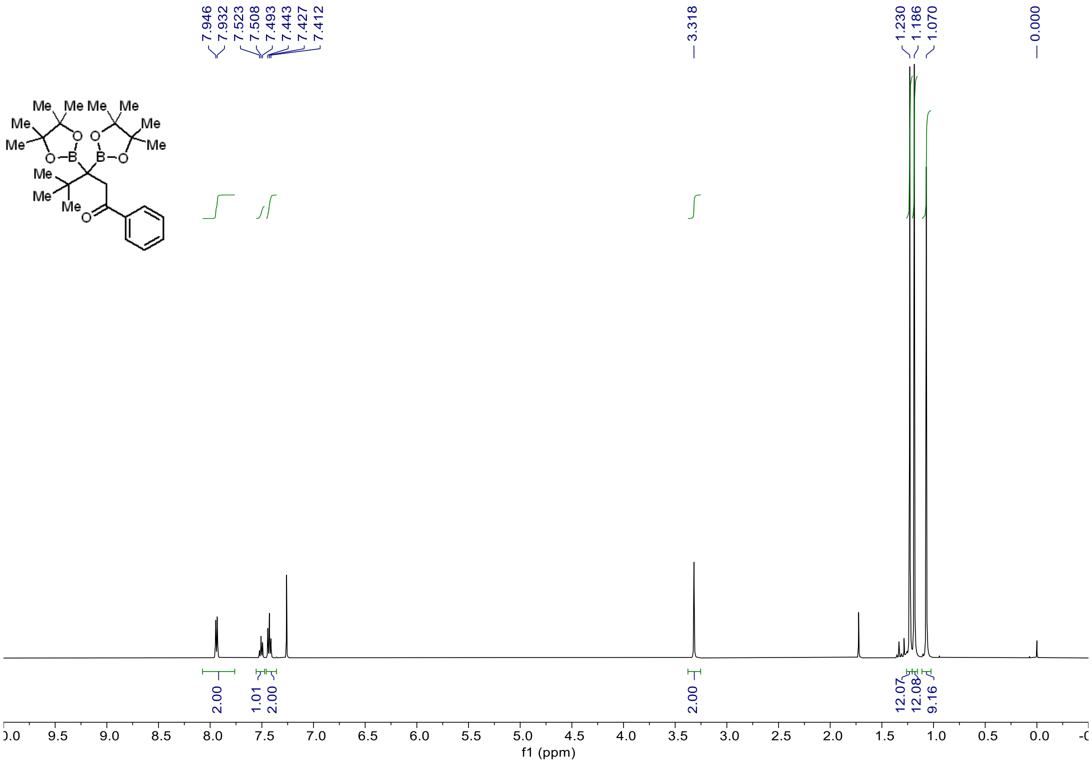

^{1}H NMR (500 MHz, $CDCl_3$) spectrum of compound **4aa**

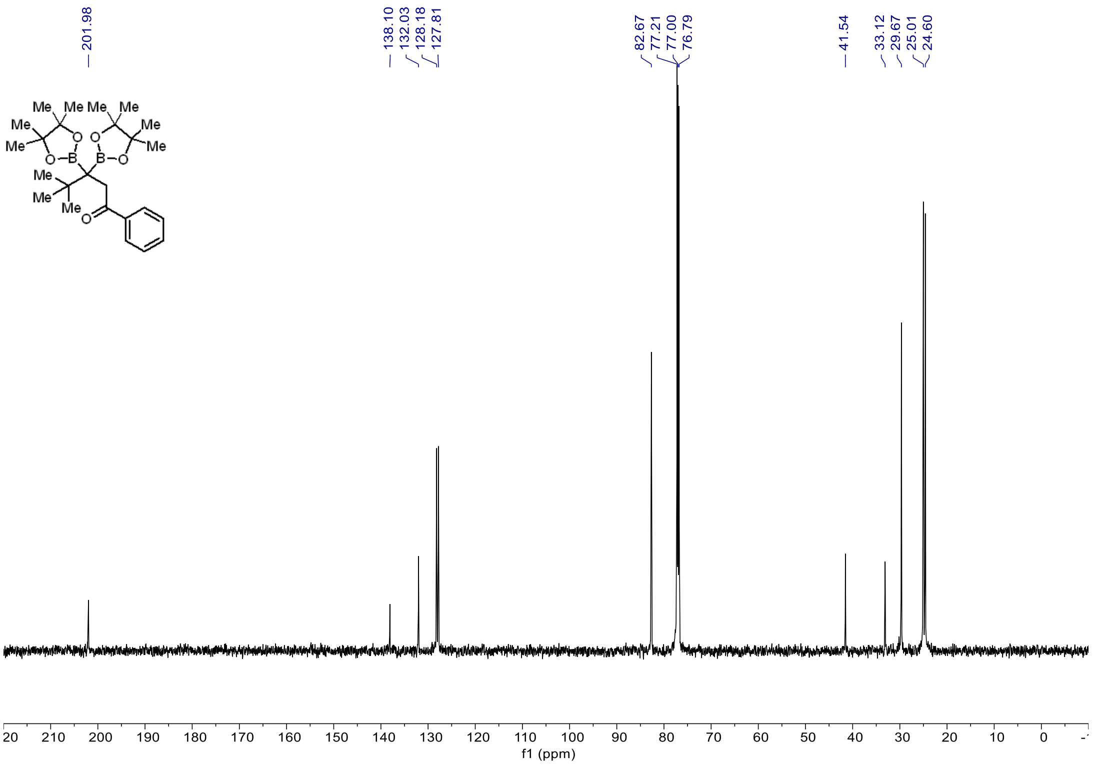

^{13}C NMR (150 MHz, $CDCl_3$) spectrum of compound **4aa**

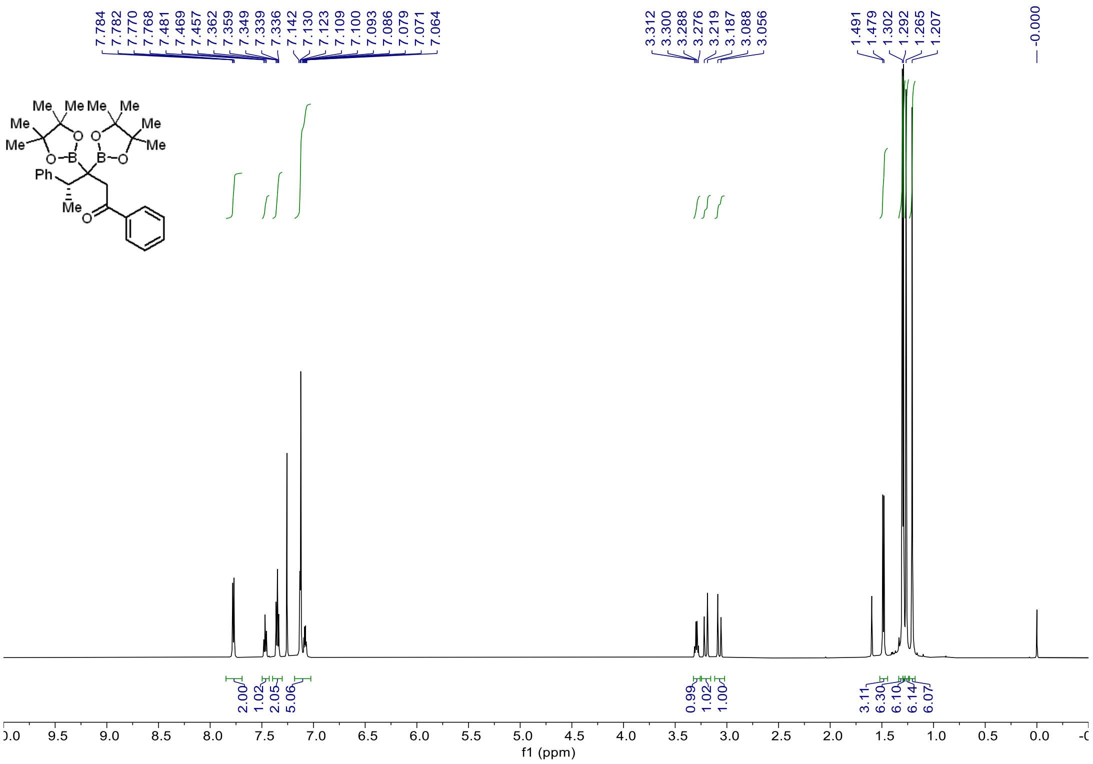

^{1}H NMR (600 MHz, $CDCl_3$) spectrum of compound **4ab**

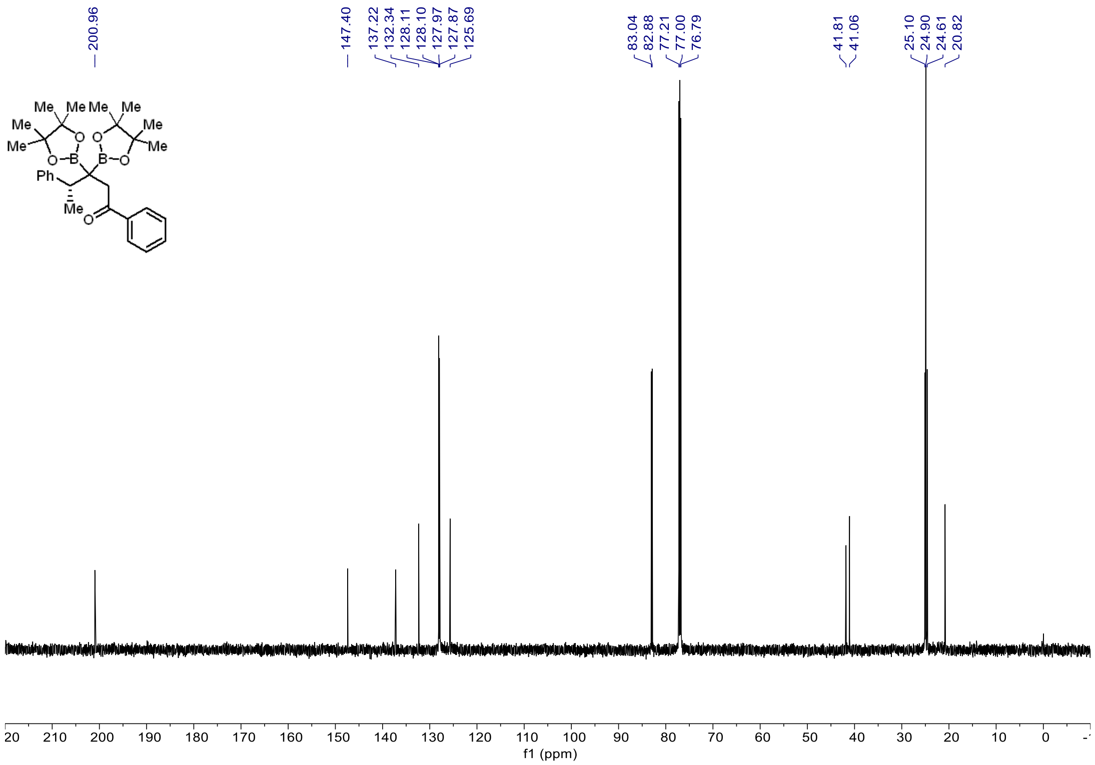

^{13}C NMR (150 MHz, $CDCl_3$) spectrum of compound **4ab**

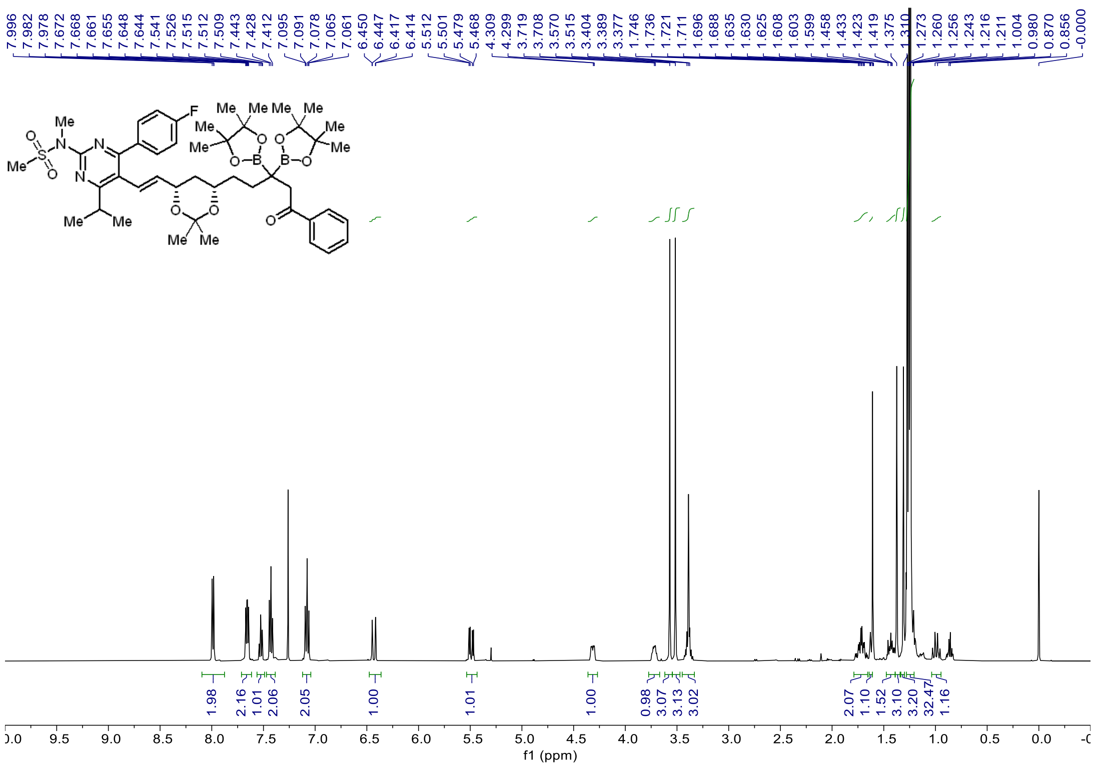

^{1}H NMR (500 MHz, $CDCl_3$) spectrum of compound **4ac**

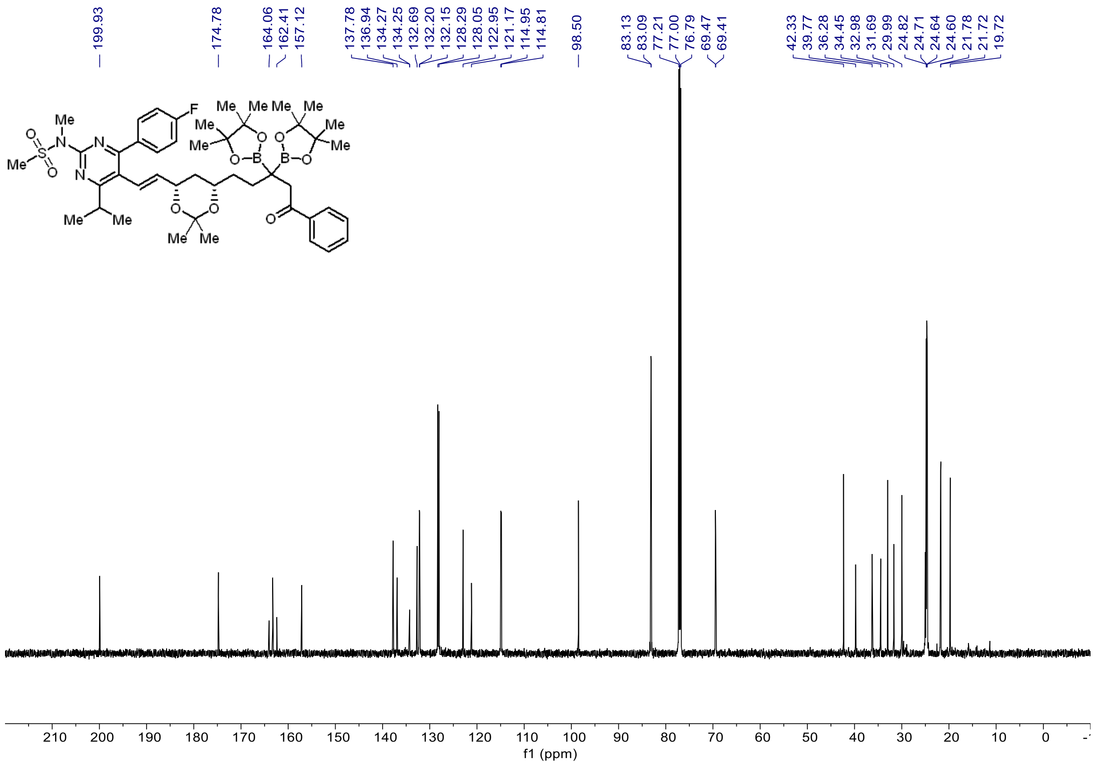

^{13}C NMR (150 MHz, $CDCl_3$) spectrum of compound **4ac**

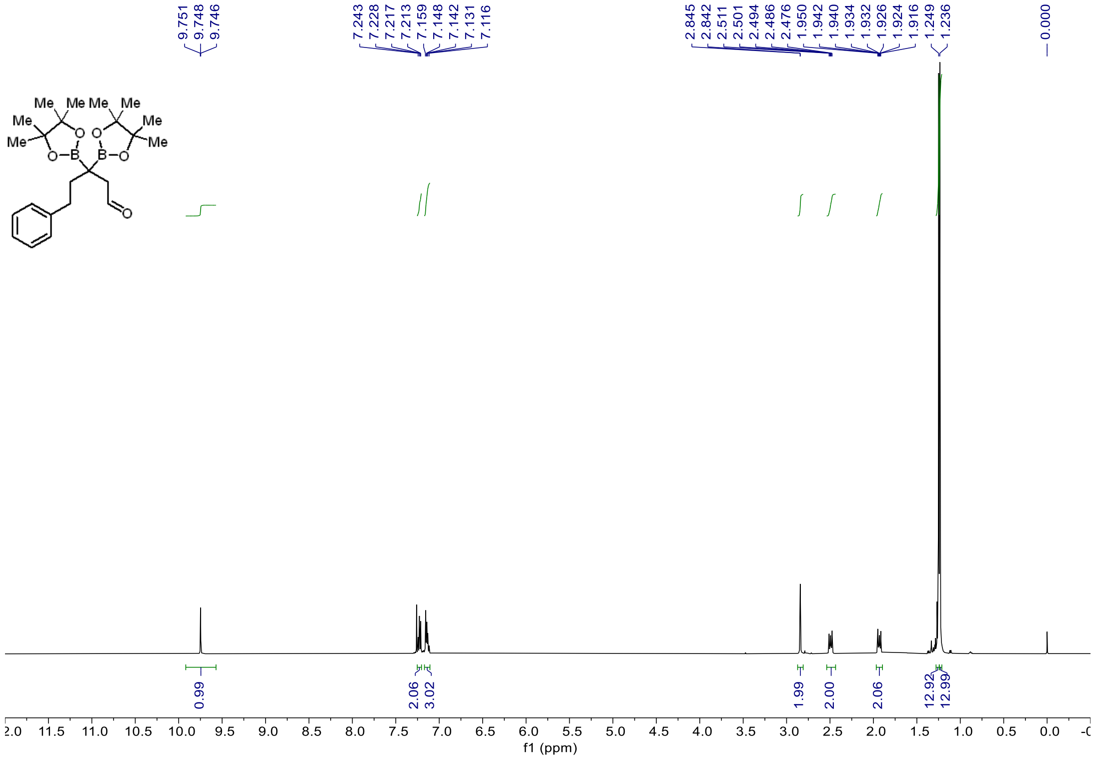

^{1}H NMR (500 MHz, $CDCl_3$) spectrum of compound **5a**

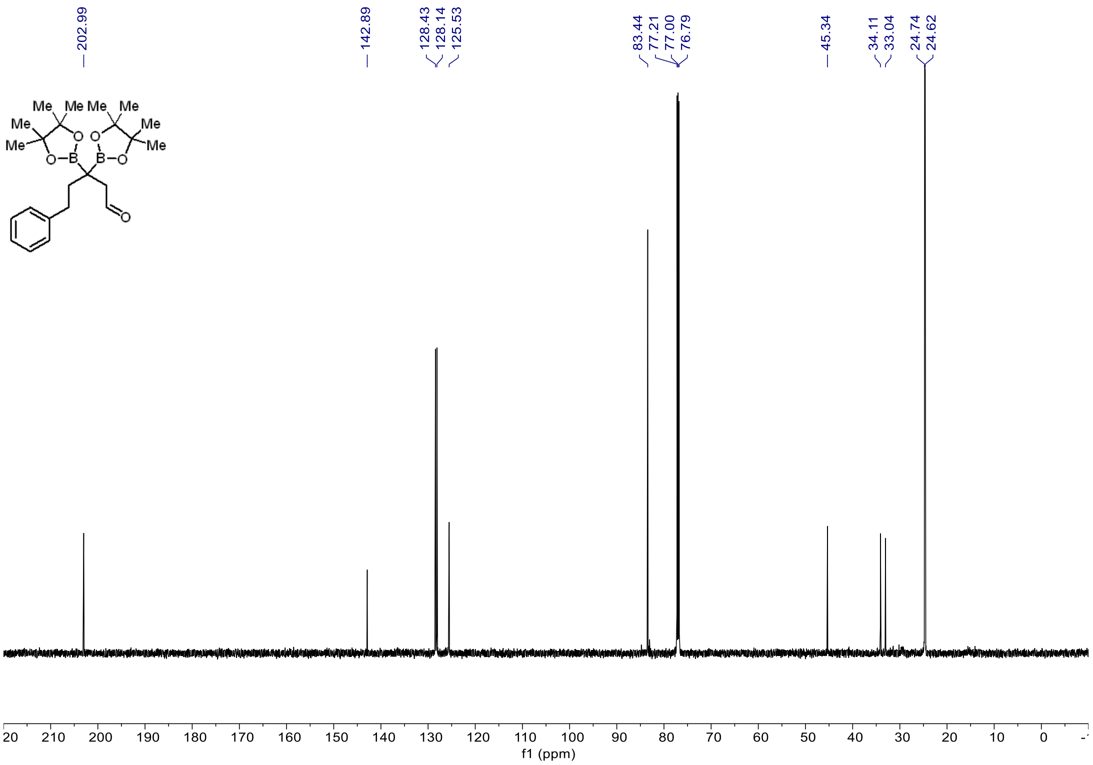

^{13}C NMR (150 MHz, $CDCl_3$) spectrum of compound **5a**

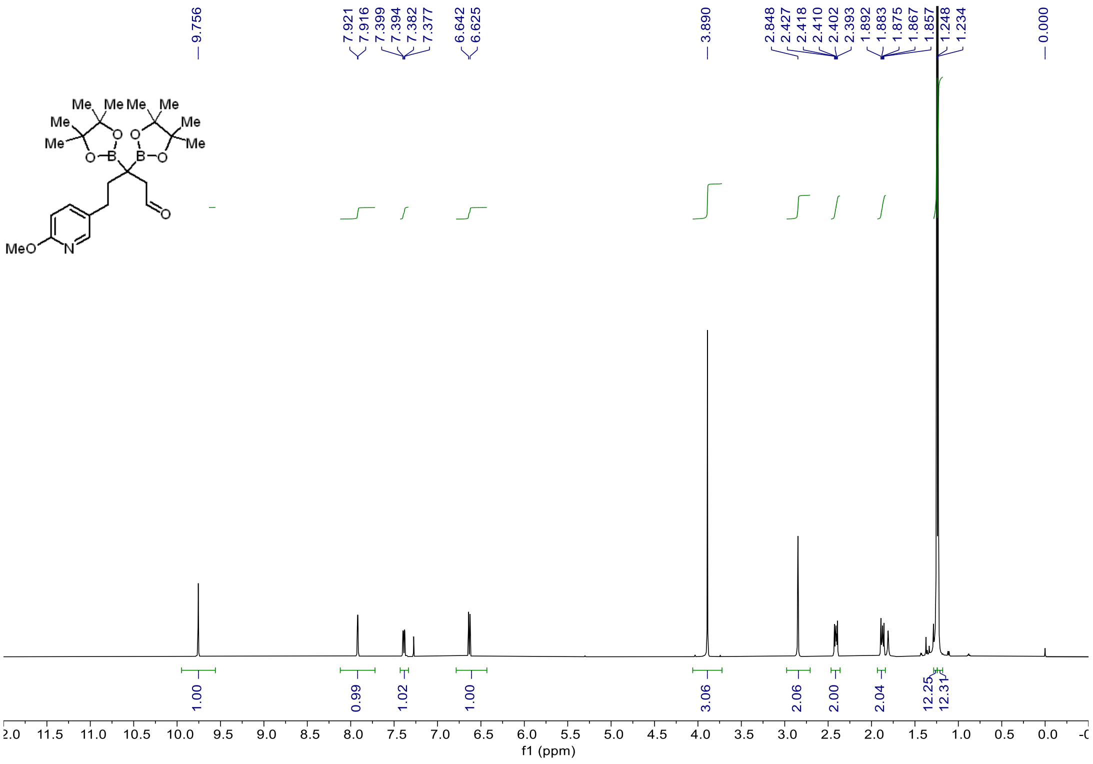

^{1}H NMR (500 MHz, $CDCl_3$) spectrum of compound **5b**

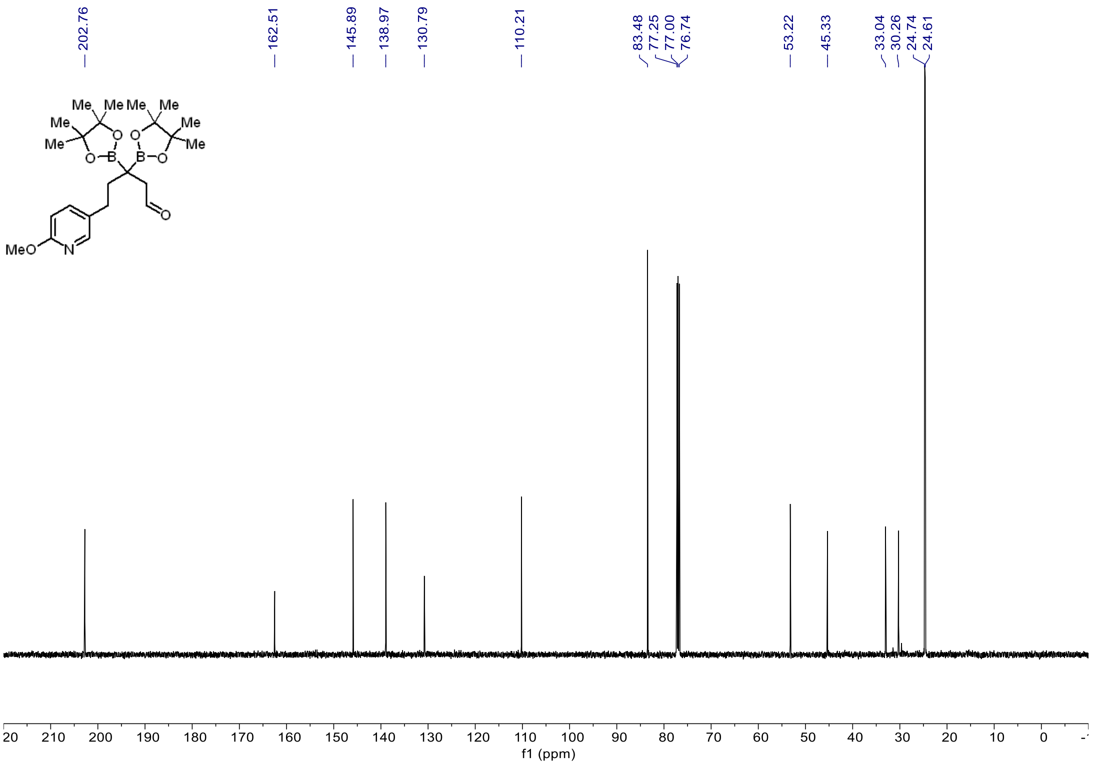

^{13}C NMR (125 MHz, CDCl$_3$) spectrum of compound **5b**

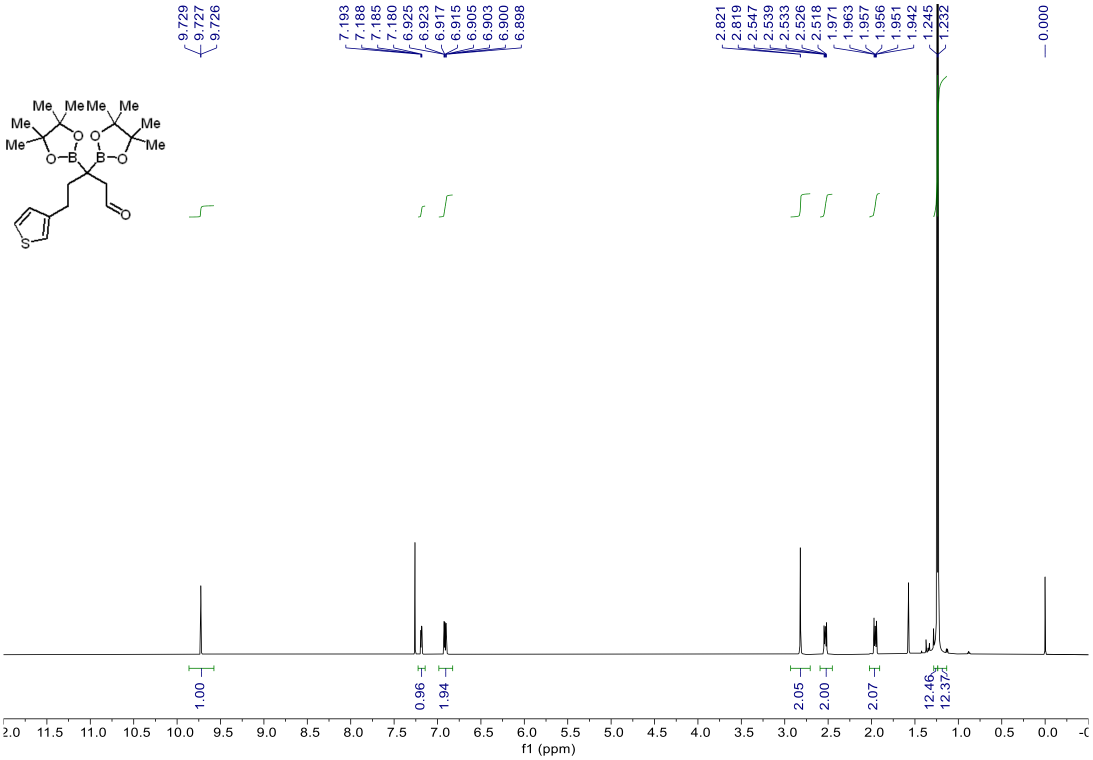

^{1}H NMR (600 MHz, CDCl$_3$) spectrum of compound **5c**

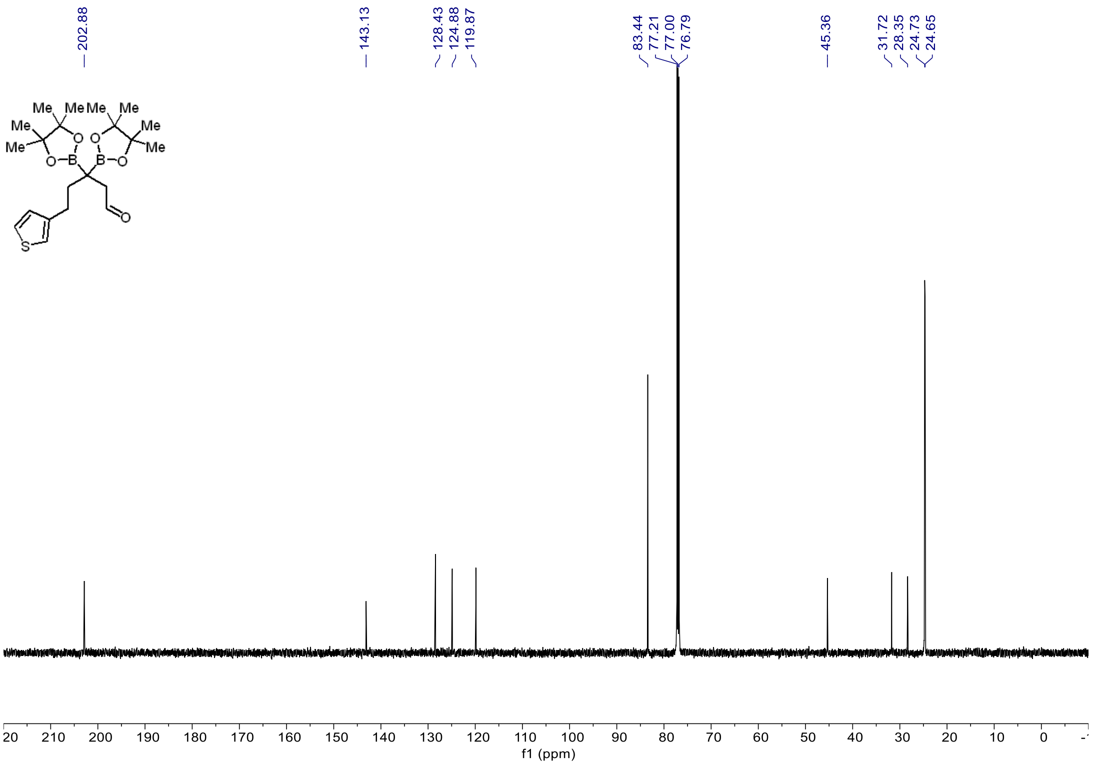

^{13}C NMR (150 MHz, $CDCl_3$) spectrum of compound **5c**

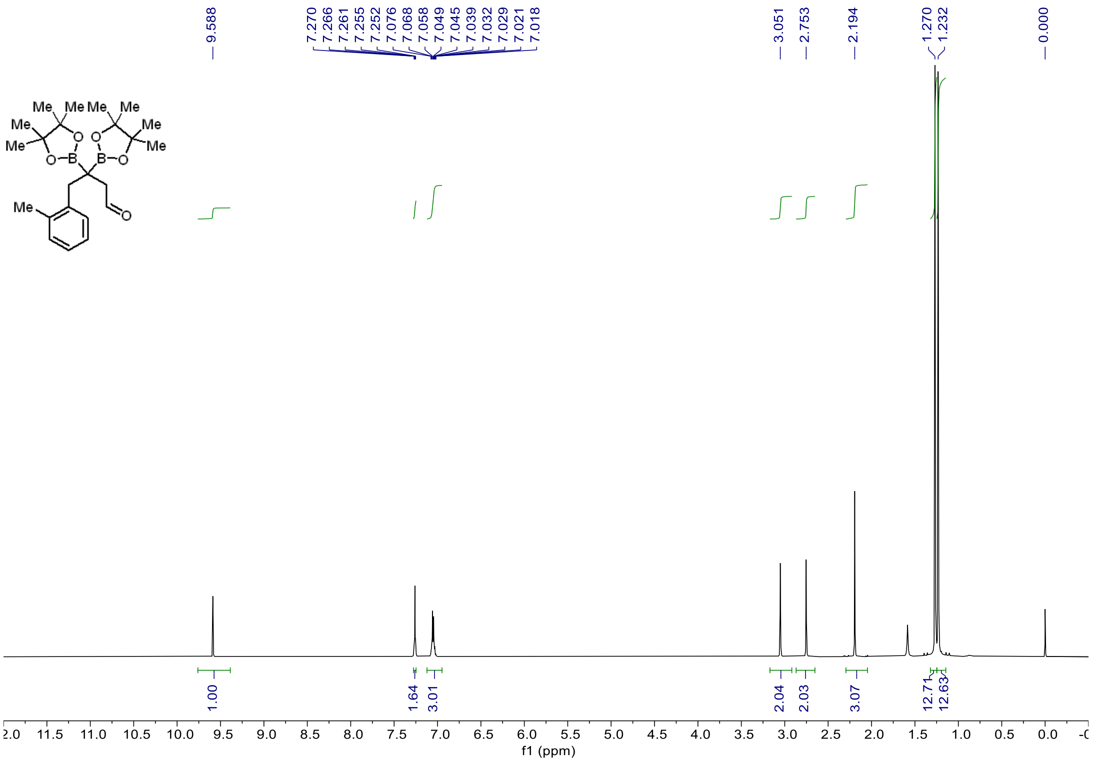

^{1}H NMR (500 MHz, $CDCl_3$) spectrum of compound **5d**

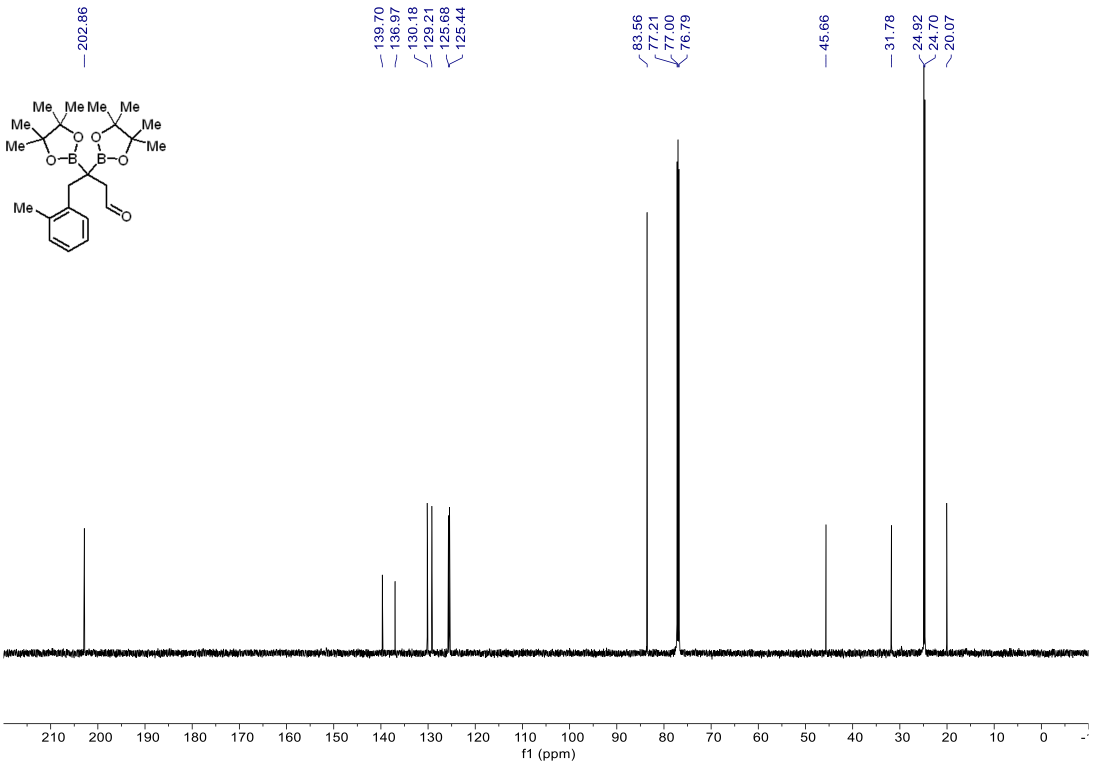

^{13}C NMR (150 MHz, CDCl$_3$) spectrum of compound **5d**

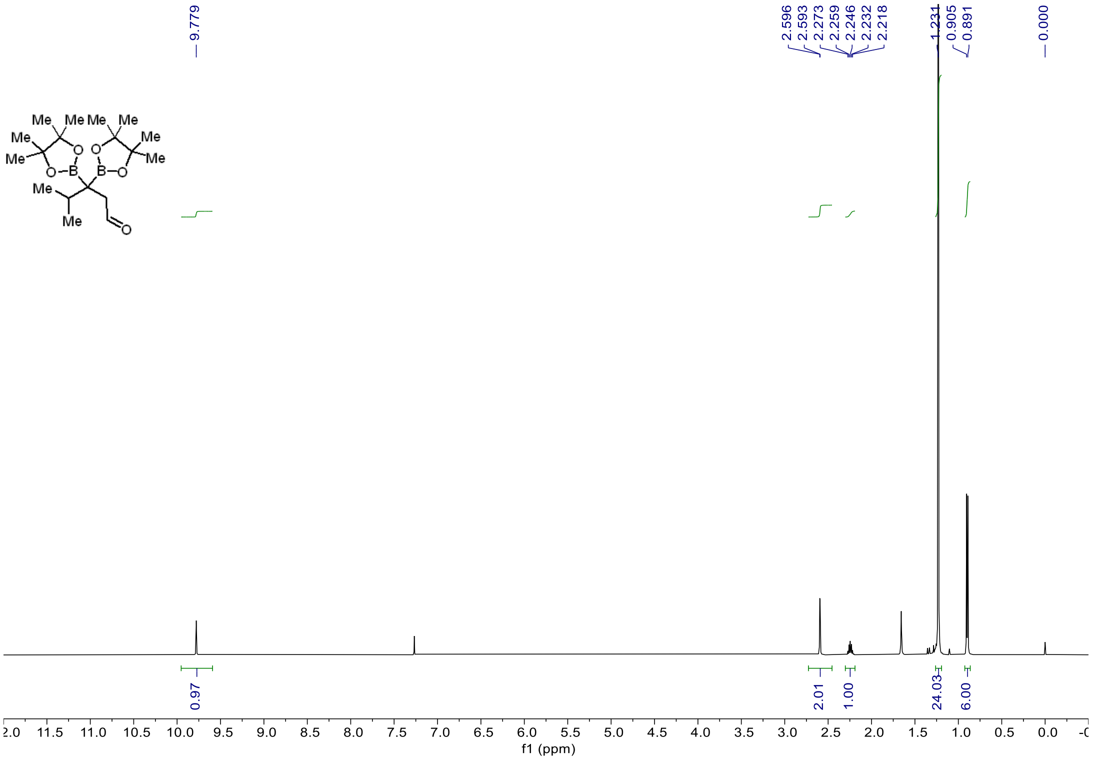

^{1}H NMR (500 MHz, CDCl$_3$) spectrum of compound **5e**

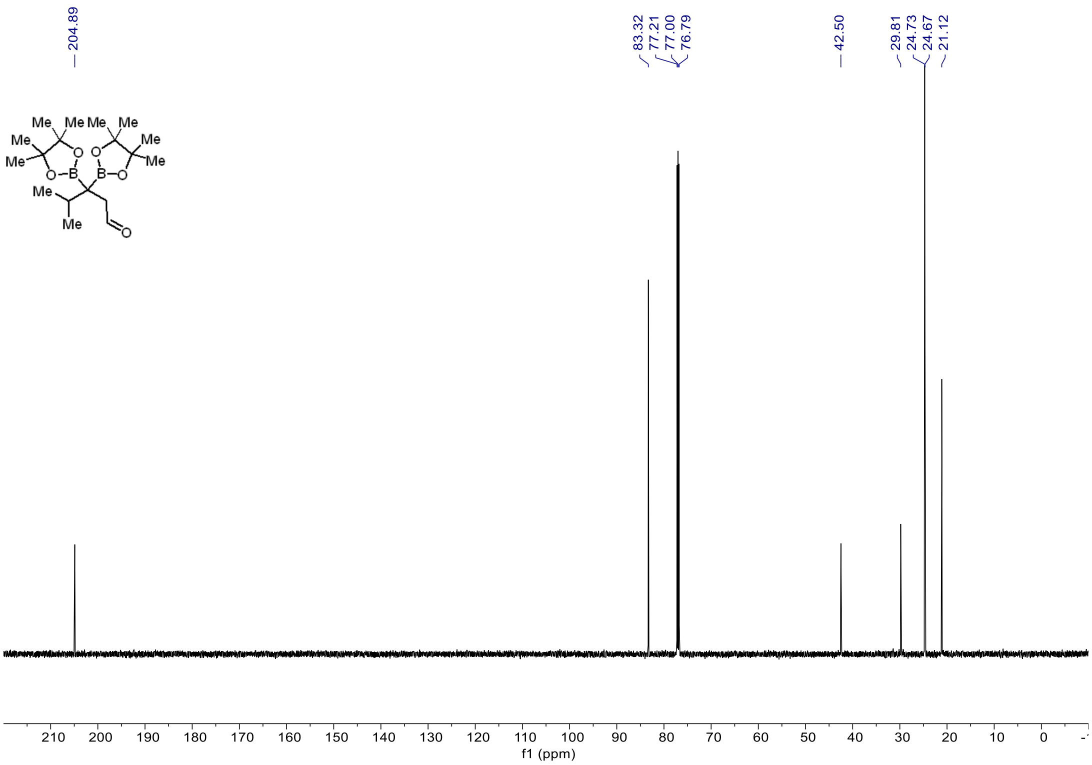

^{13}C NMR (150 MHz, $CDCl_3$) spectrum of compound **5e**

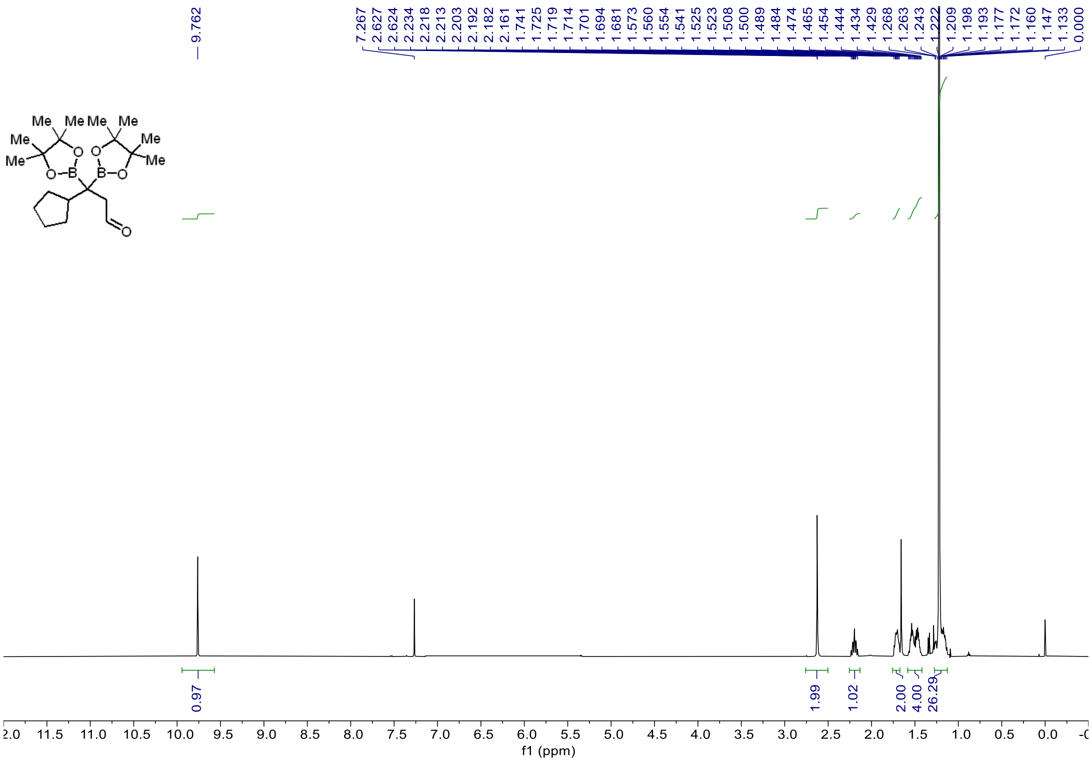

^{1}H NMR (500 MHz, $CDCl_3$) spectrum of compound **5f**

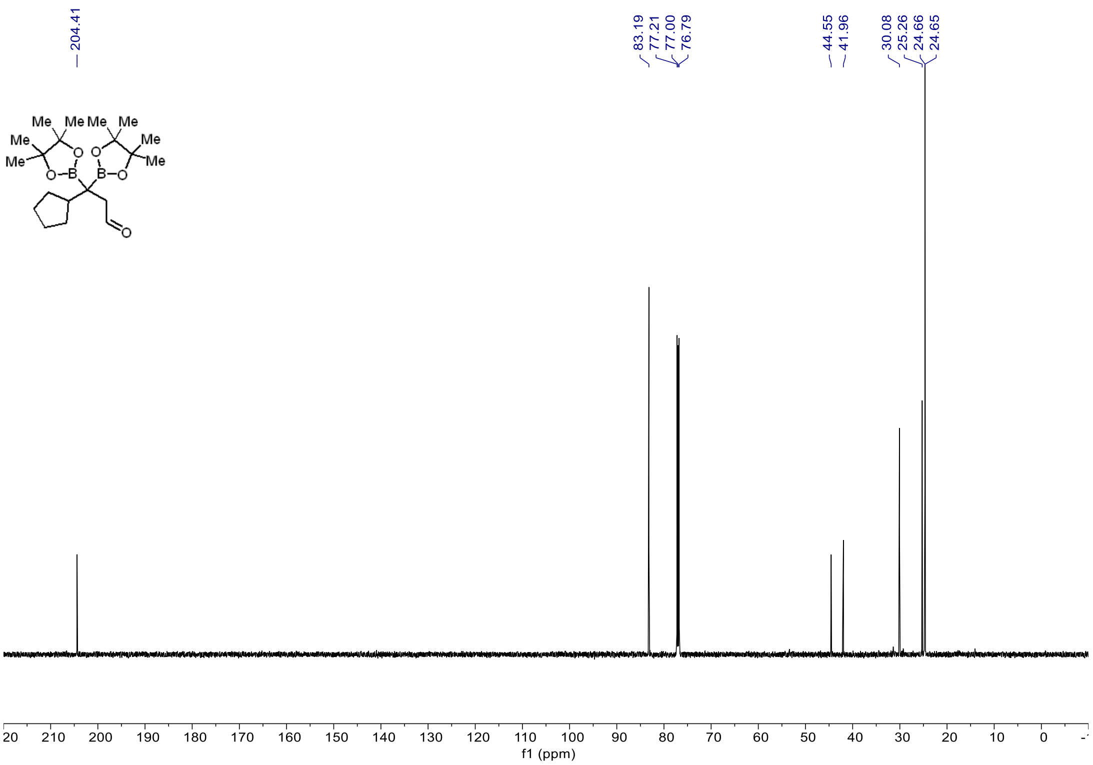

^{13}C NMR (150 MHz, $CDCl_3$) spectrum of compound **5f**

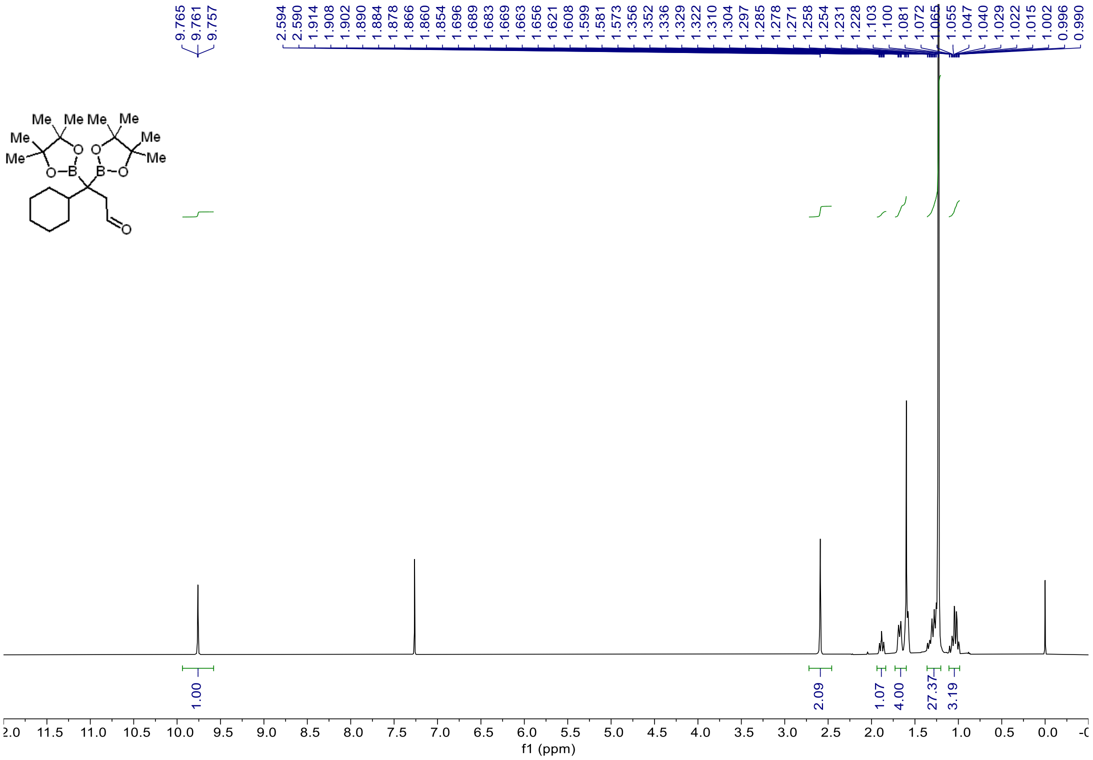

^{1}H NMR (500 MHz, $CDCl_3$) spectrum of compound **5g**

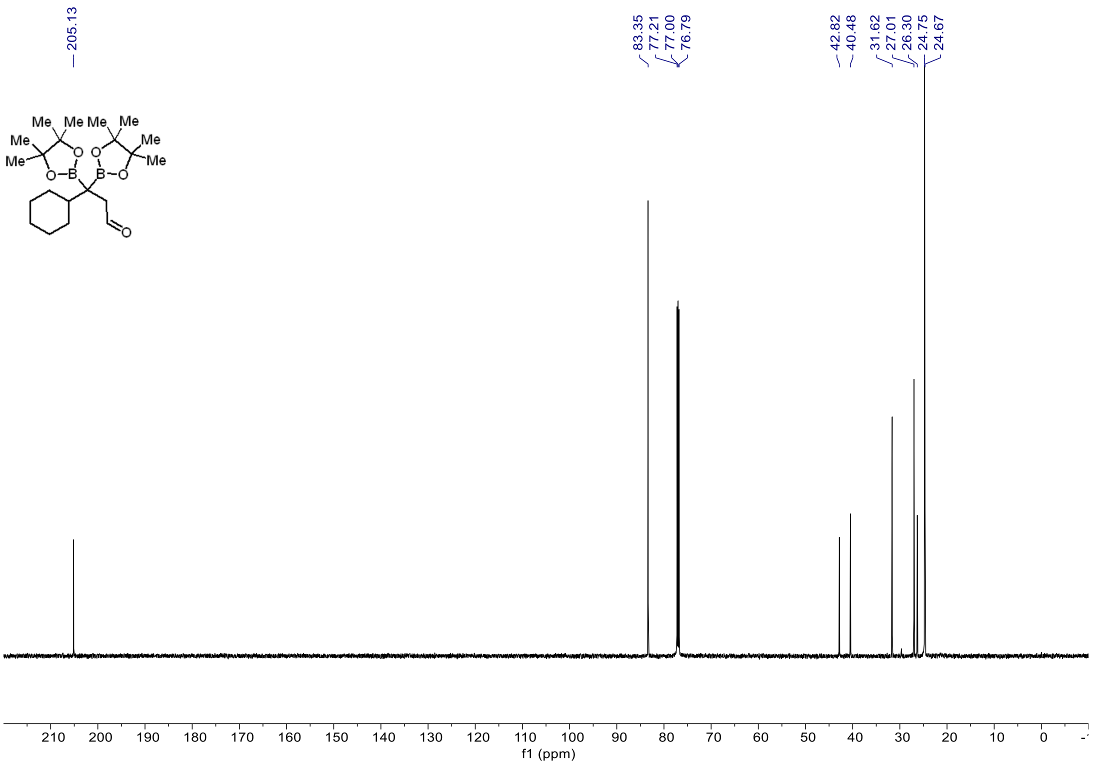

^{13}C NMR (150 MHz, $CDCl_3$) spectrum of compound **5g**

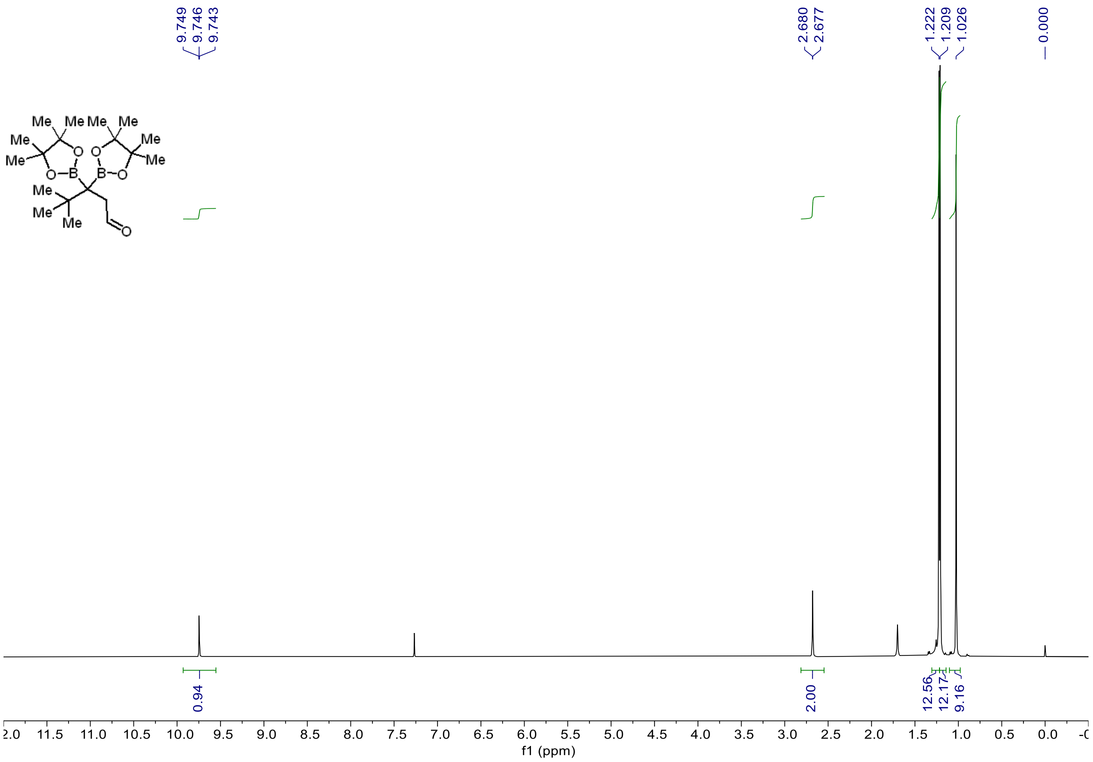

^{1}H NMR (500 MHz, $CDCl_3$) spectrum of compound **5h**

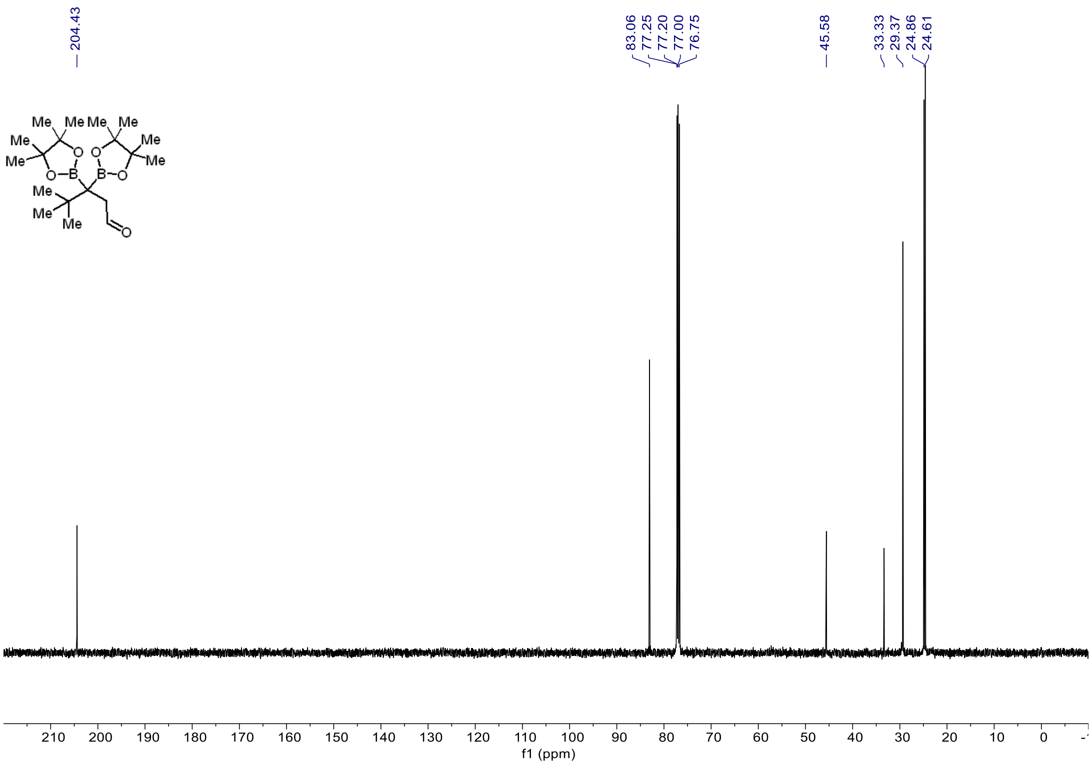

^{13}C NMR (125 MHz, $CDCl_3$) spectrum of compound **5h**

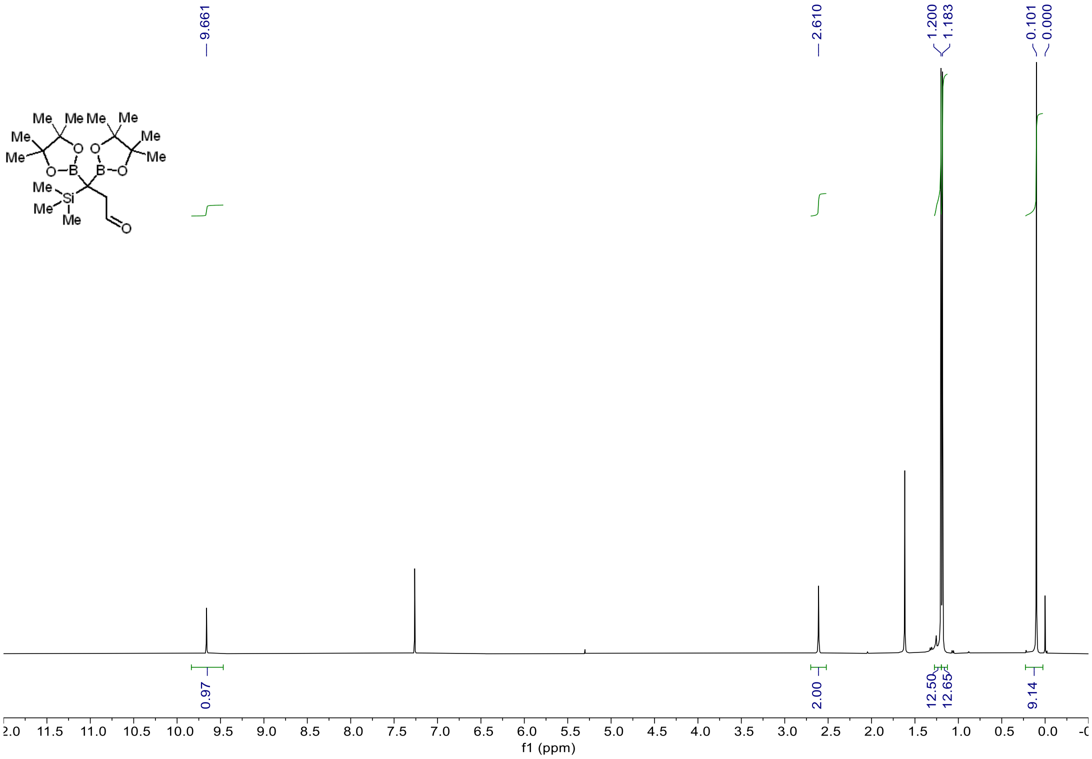

^{1}H NMR (500 MHz, $CDCl_3$) spectrum of compound **5i**

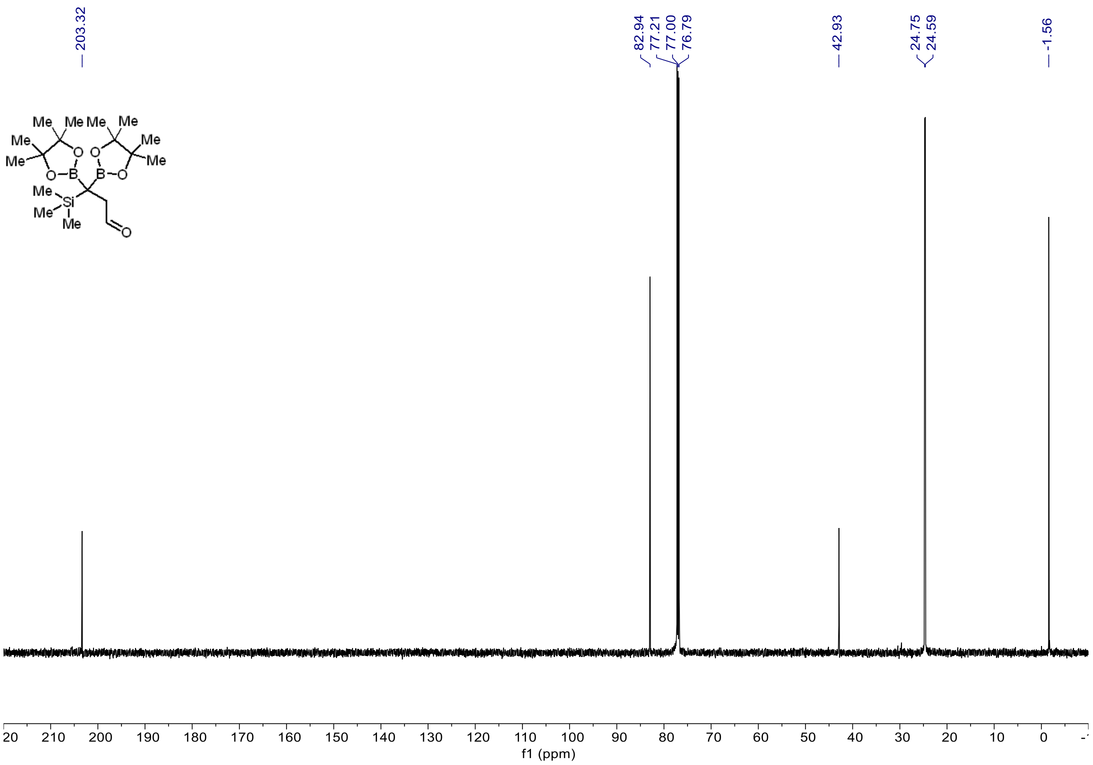

^{13}C NMR (150 MHz, $CDCl_3$) spectrum of compound **5i**

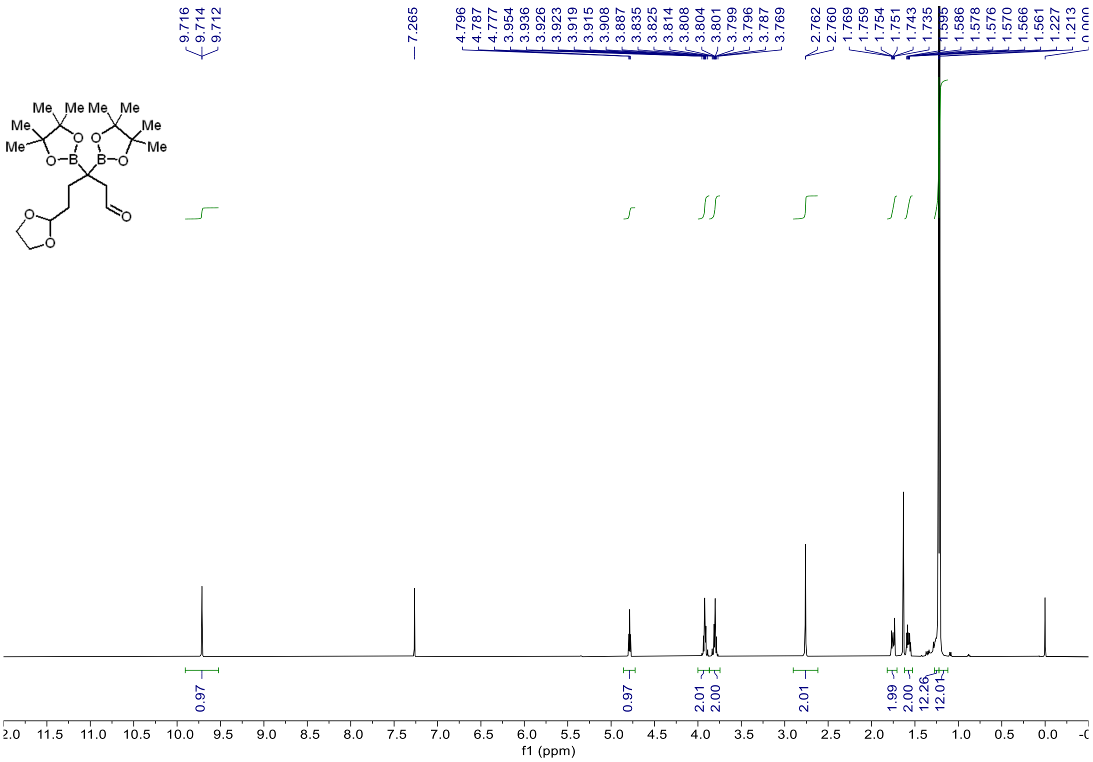

^{1}H NMR (500 MHz, $CDCl_3$) spectrum of compound **5j**

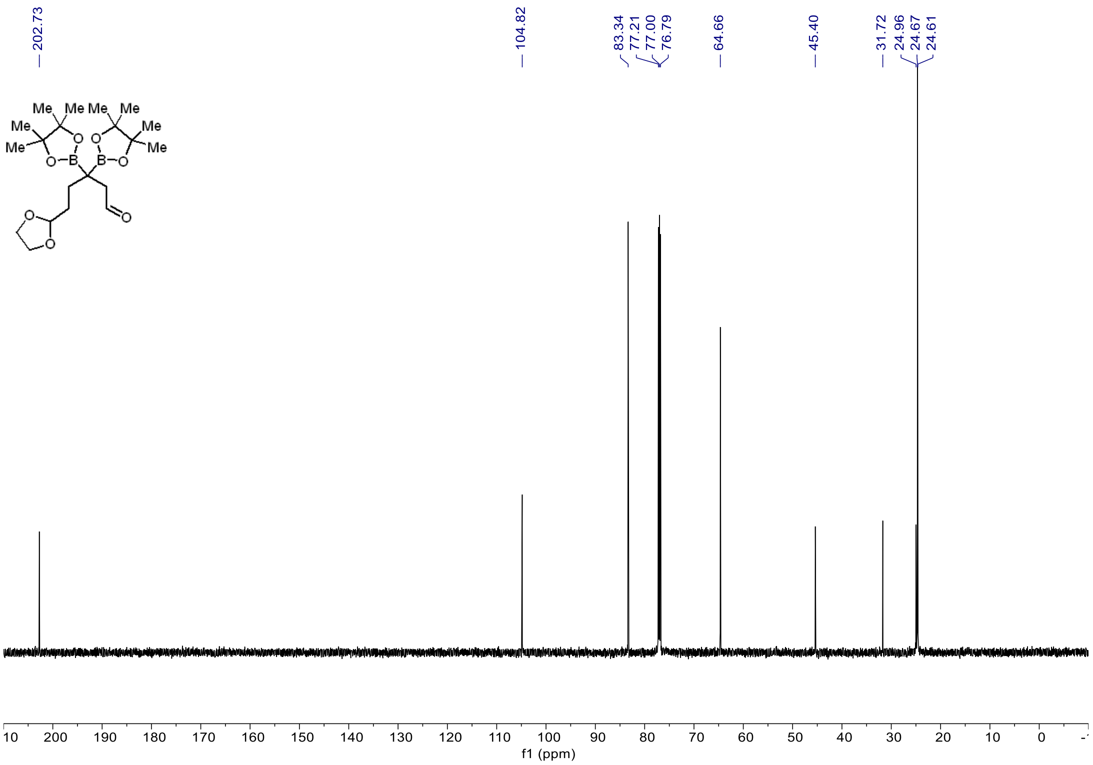

^{13}C NMR (150 MHz, $CDCl_3$) spectrum of compound **5j**

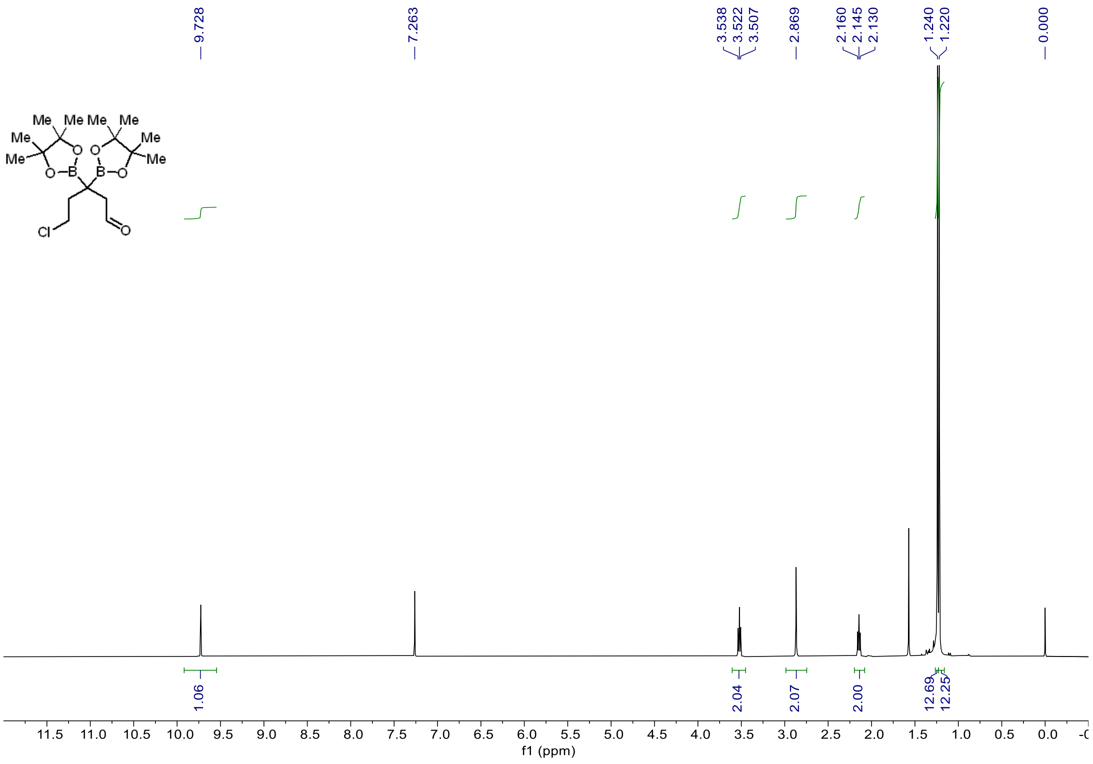

^{1}H NMR (500 MHz, $CDCl_3$) spectrum of compound **5k**

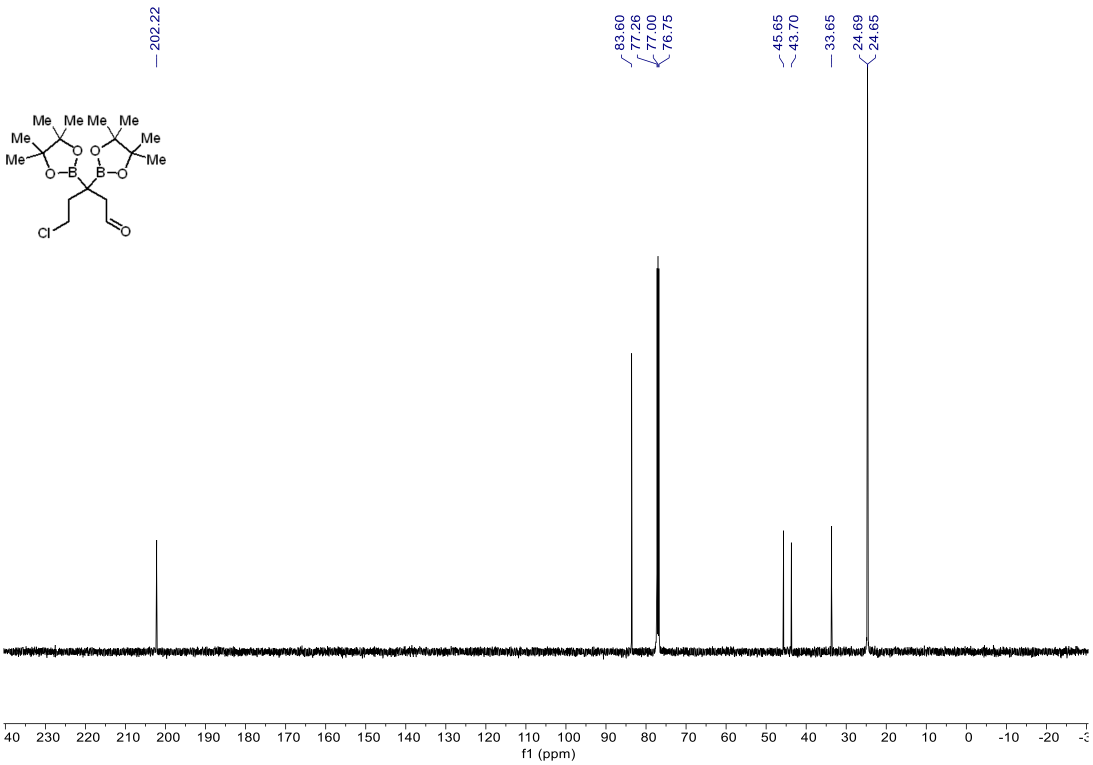

^{13}C NMR (150 MHz, $CDCl_3$) spectrum of compound **5k**

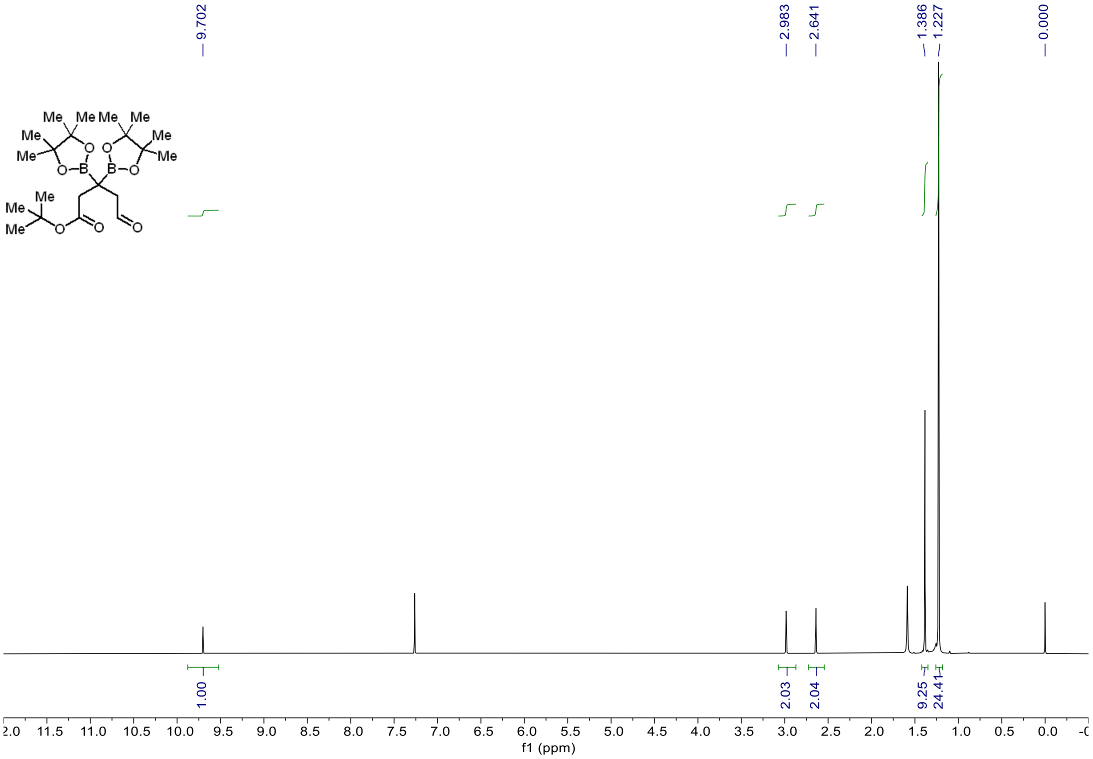

^{1}H NMR (500 MHz, $CDCl_3$) spectrum of compound **5l**

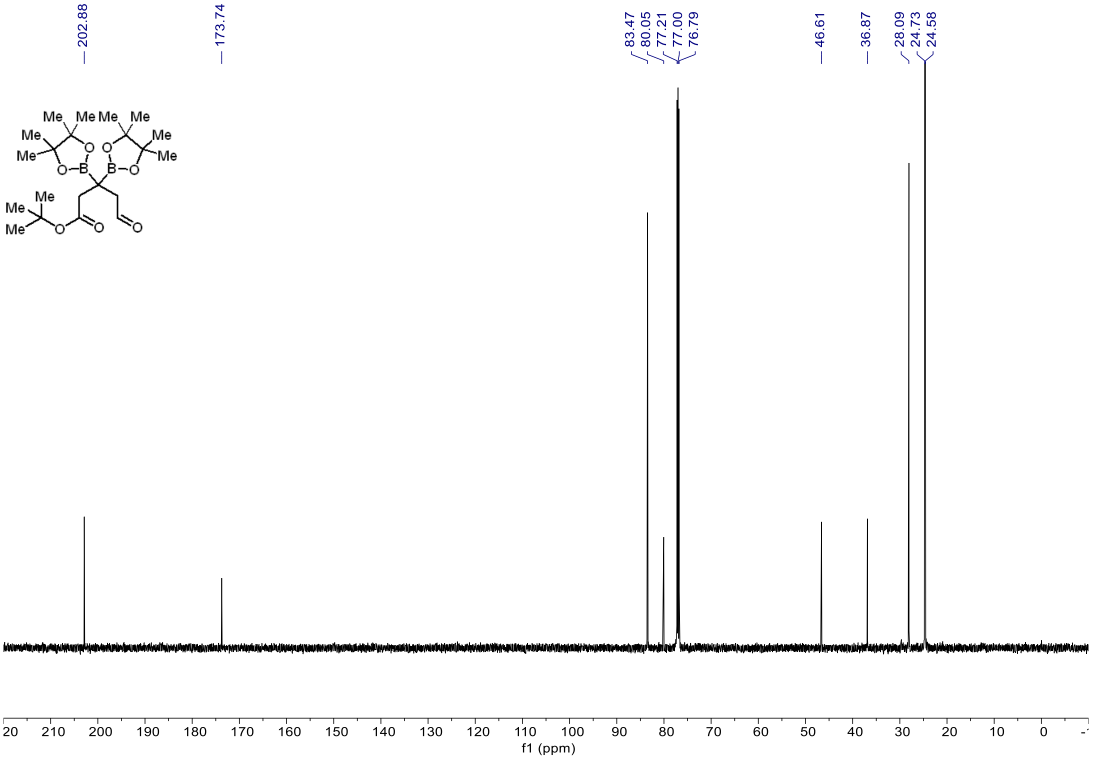

^{13}C NMR (150 MHz, $CDCl_3$) spectrum of compound **5l**

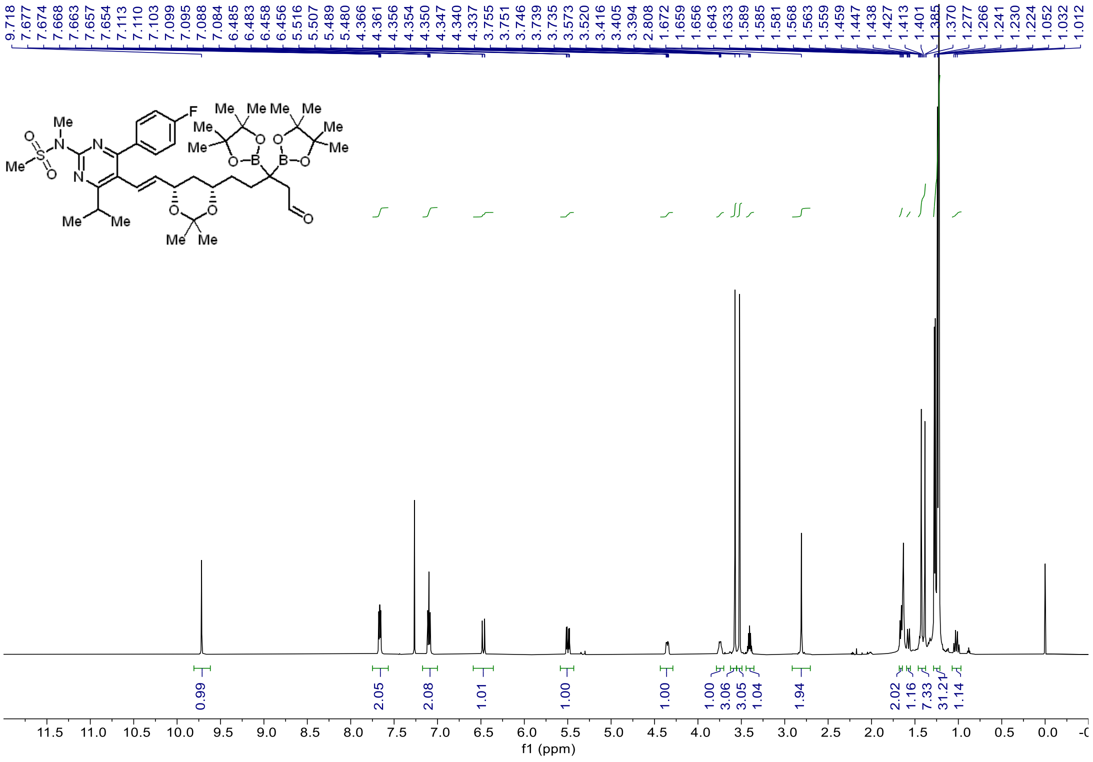

^{1}H NMR (600 MHz, $CDCl_3$) spectrum of compound **5m**

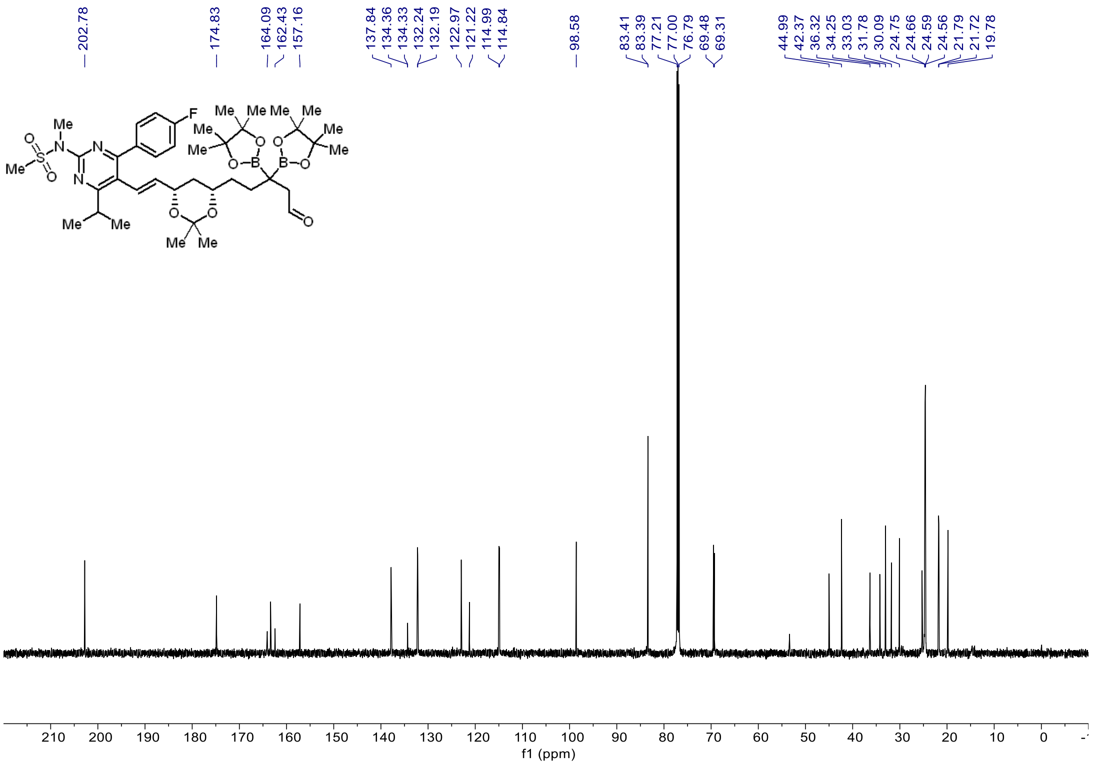

^{13}C NMR (150 MHz, $CDCl_3$) spectrum of compound **5m**

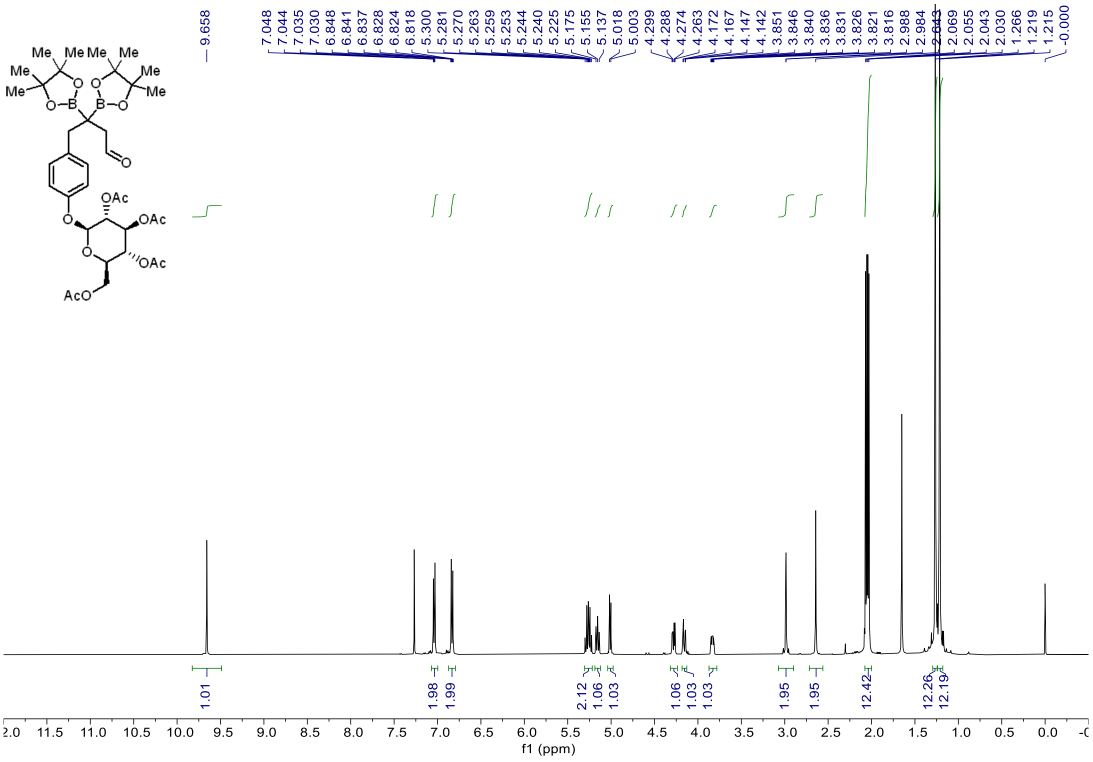

^{1}H NMR (500 MHz, $CDCl_3$) spectrum of compound **5n**

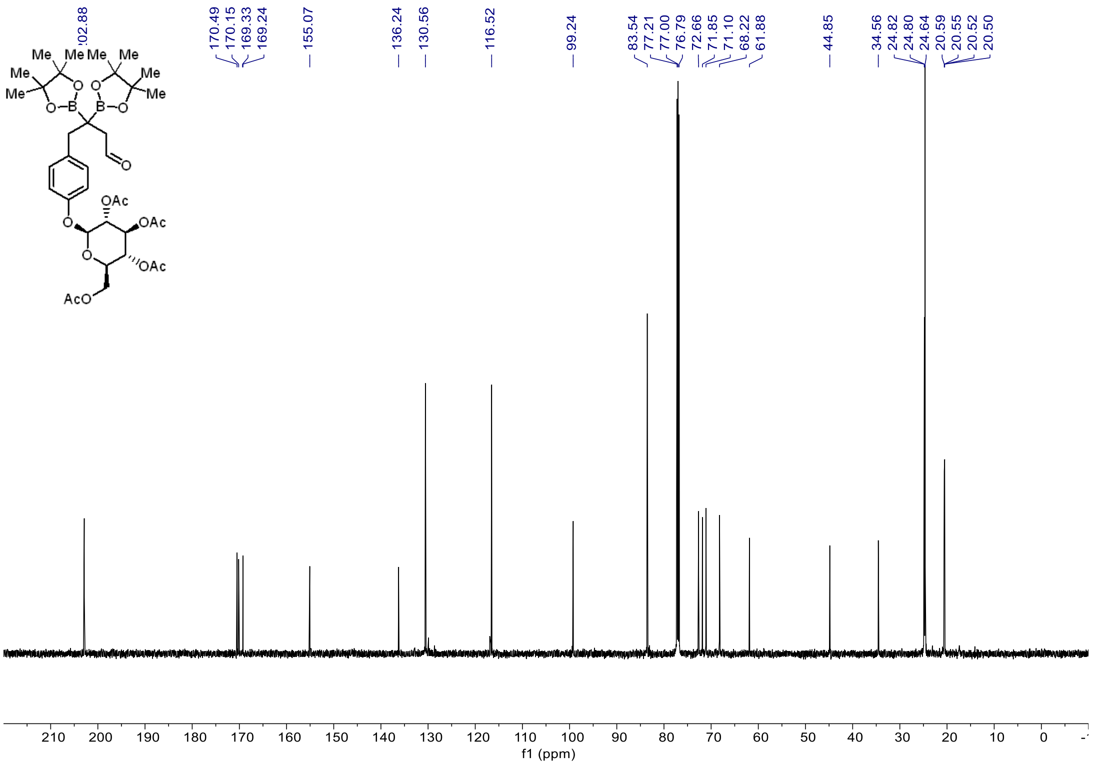

^{13}C NMR (150 MHz, CDCl$_3$) spectrum of compound **5n**

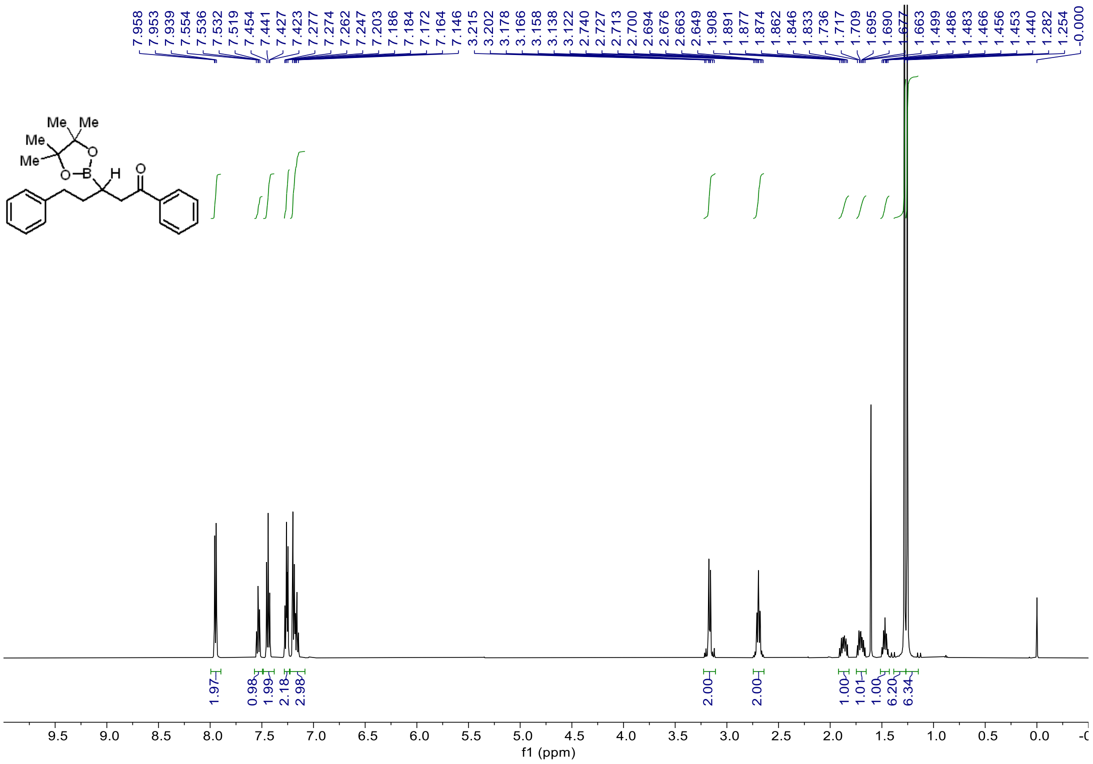

^{1}H NMR (500 MHz, CDCl$_3$) spectrum of compound **6**

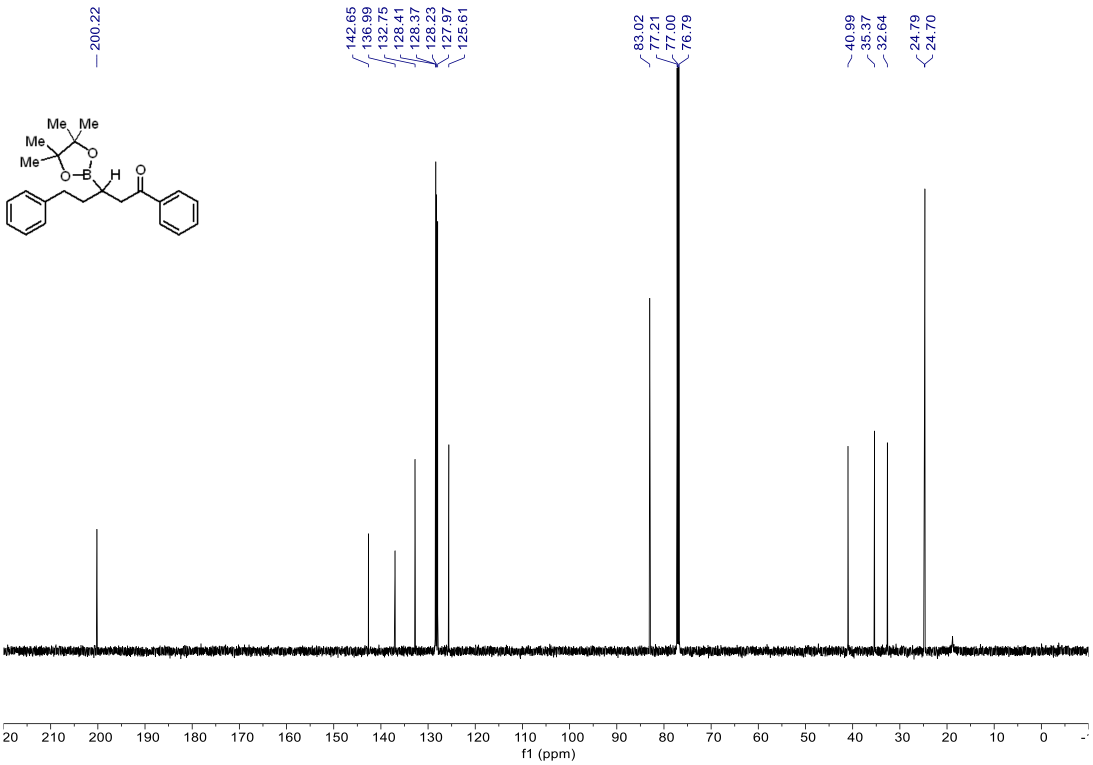

^{13}C NMR (150 MHz, $CDCl_3$) spectrum of compound **6**

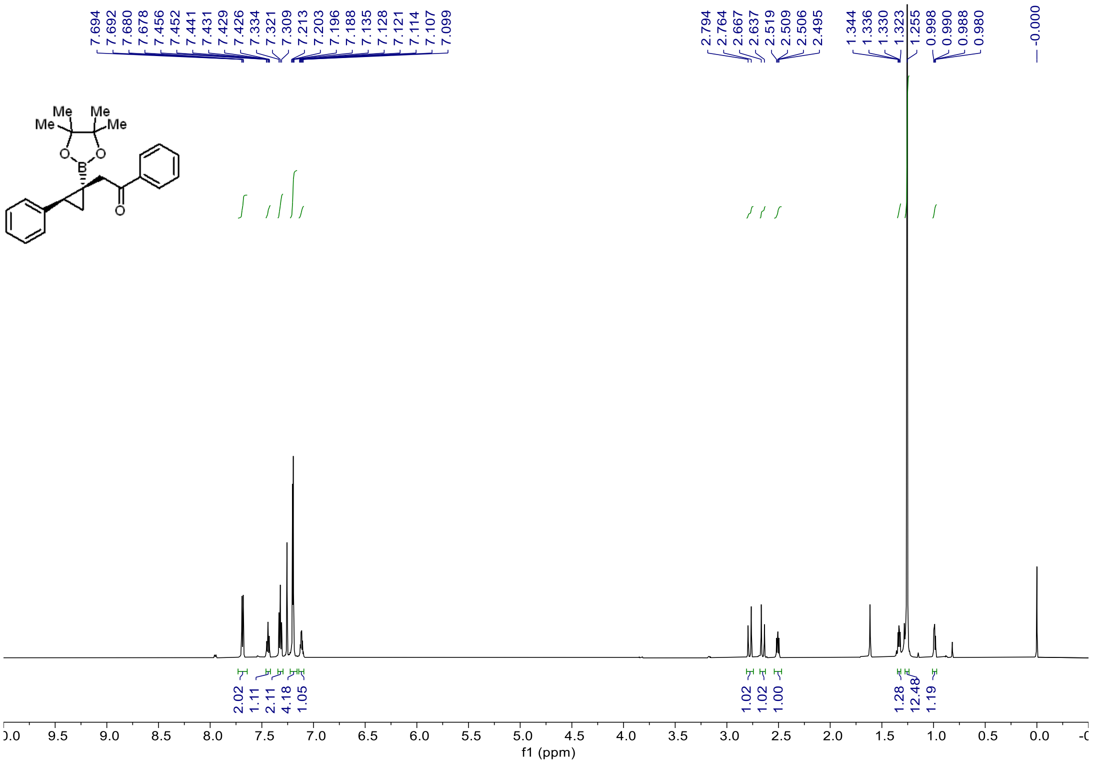

^{1}H NMR (600 MHz, $CDCl_3$) spectrum of compound **7**

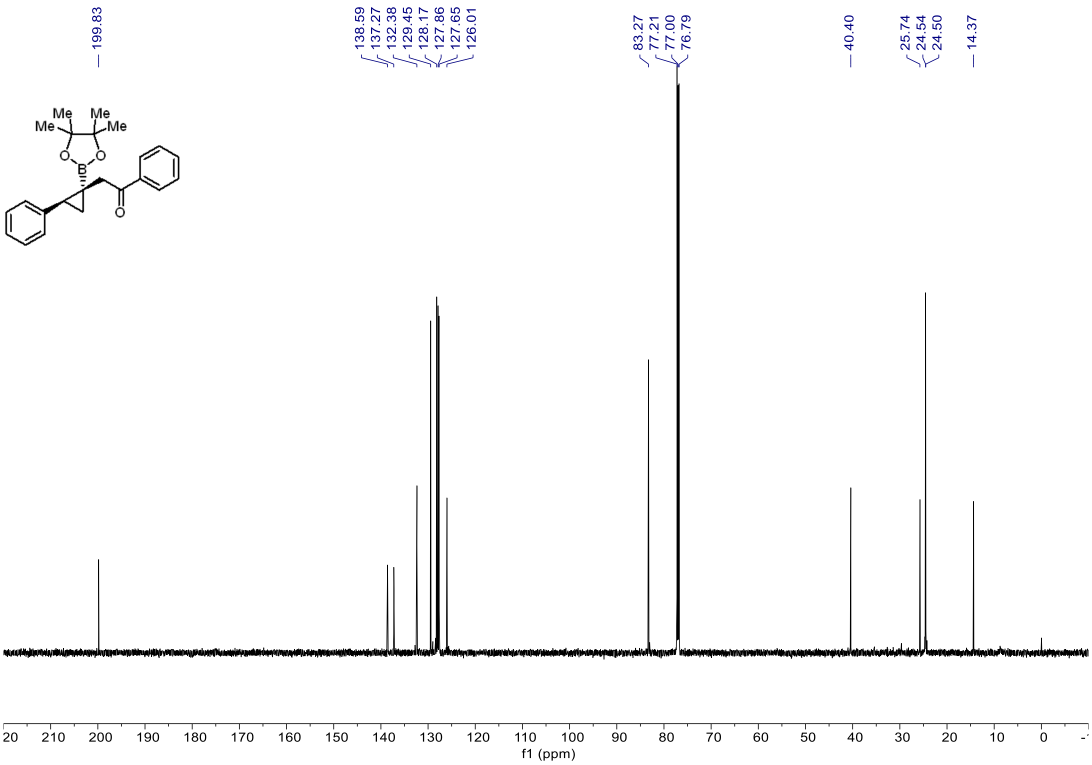

^{13}C NMR (150 MHz, $CDCl_3$) spectrum of compound **7**

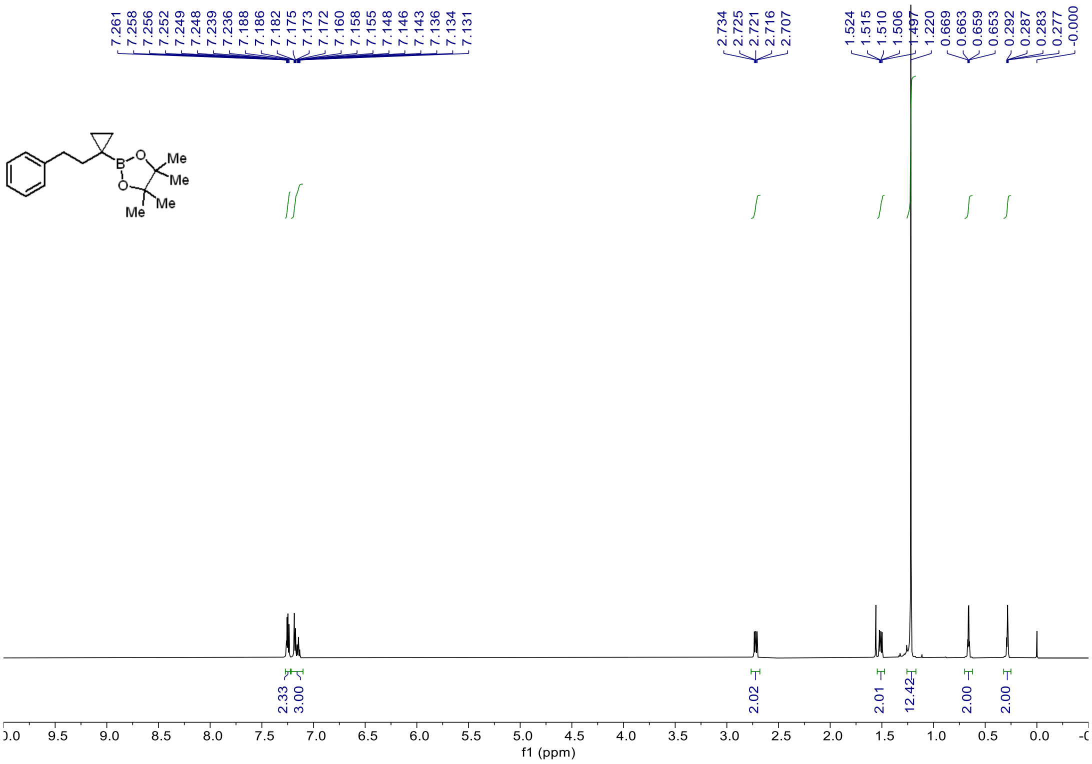

^{1}H NMR (600 MHz, $CDCl_3$) spectrum of compound **8**

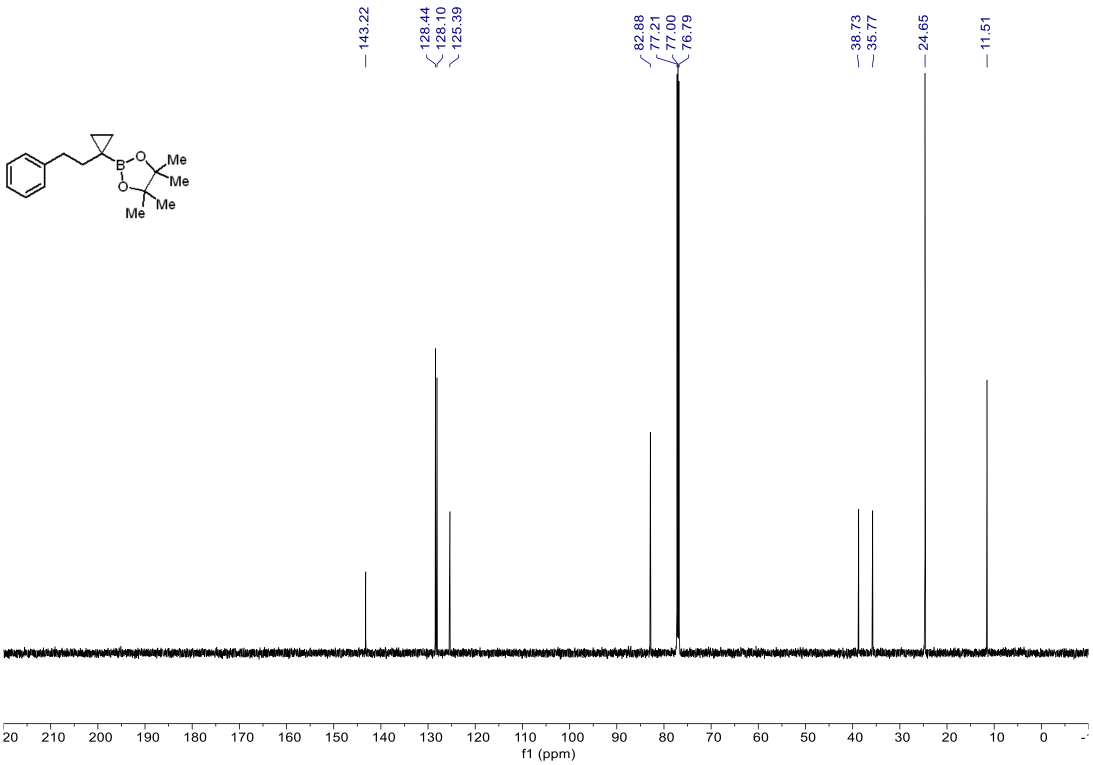

^{13}C NMR (150 MHz, $CDCl_3$) spectrum of compound **8**

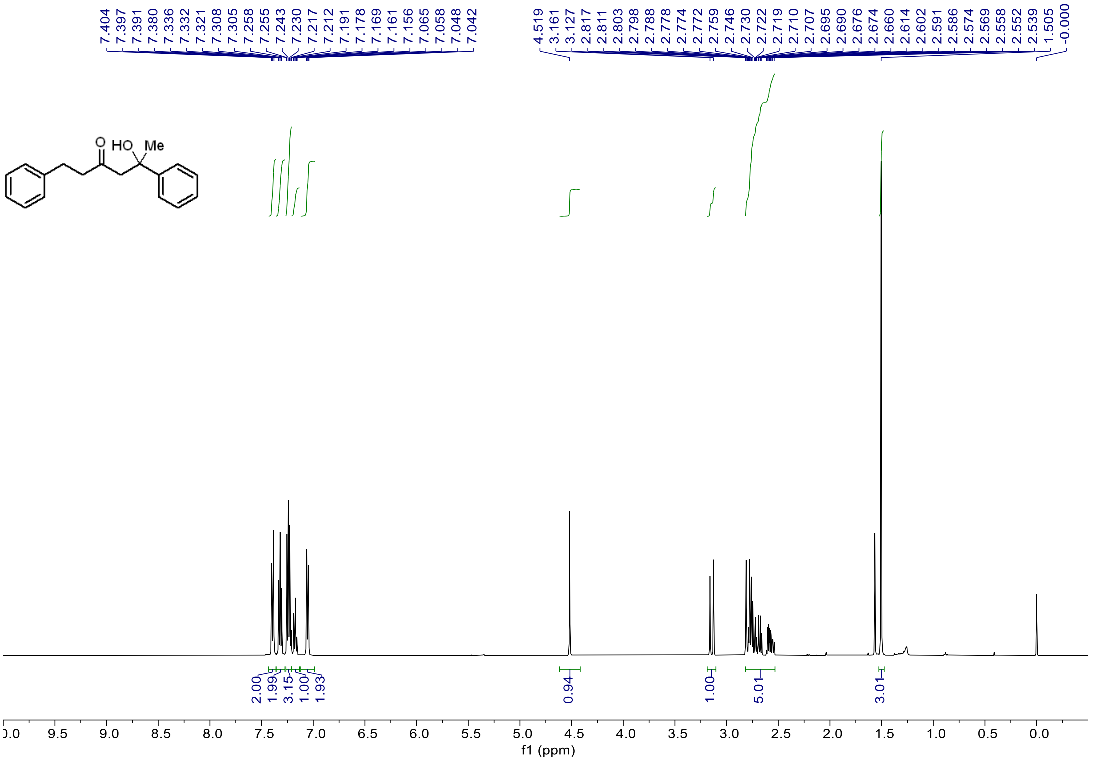

^{1}H NMR (500 MHz, $CDCl_3$) spectrum of compound **9**

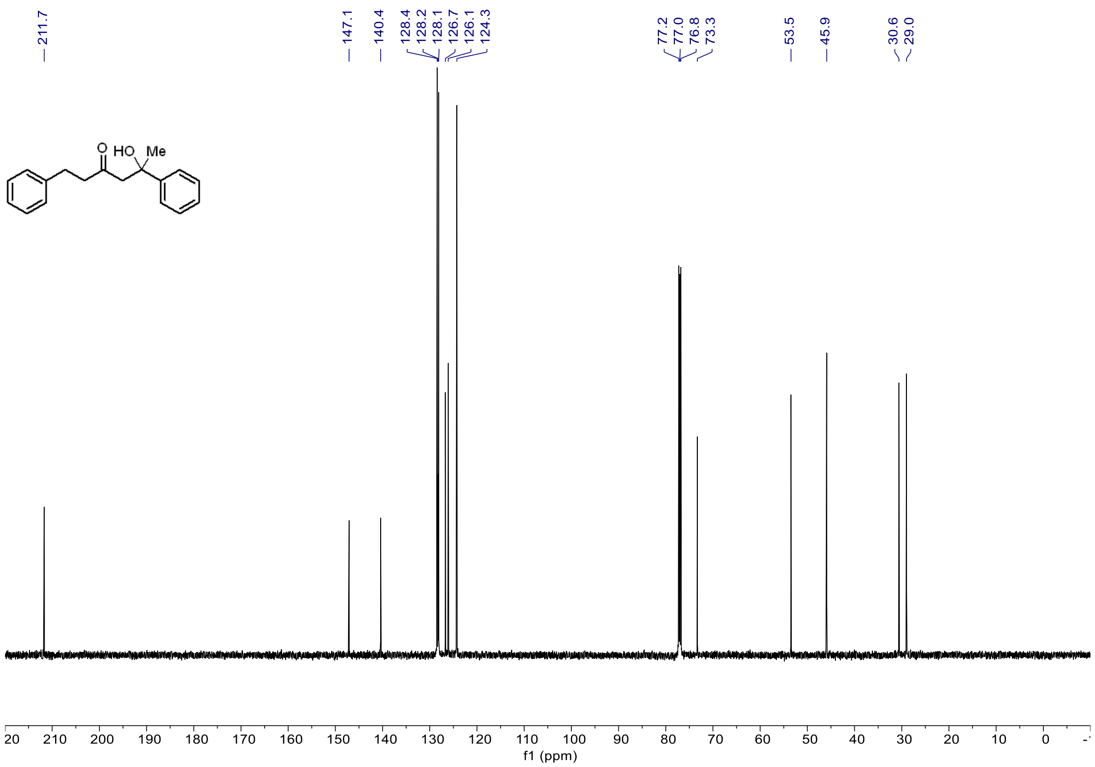

^{13}C NMR (150 MHz, $CDCl_3$) spectrum of compound **9**

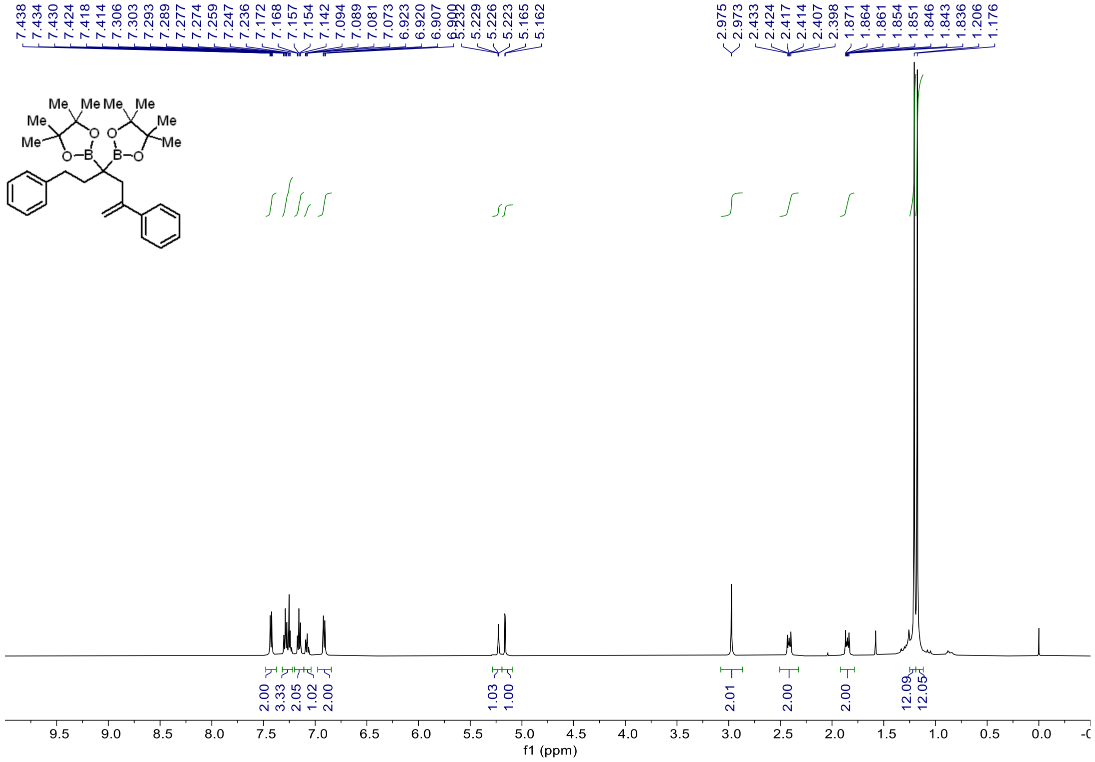

^{1}H NMR (500 MHz, $CDCl_3$) spectrum of compound **10**

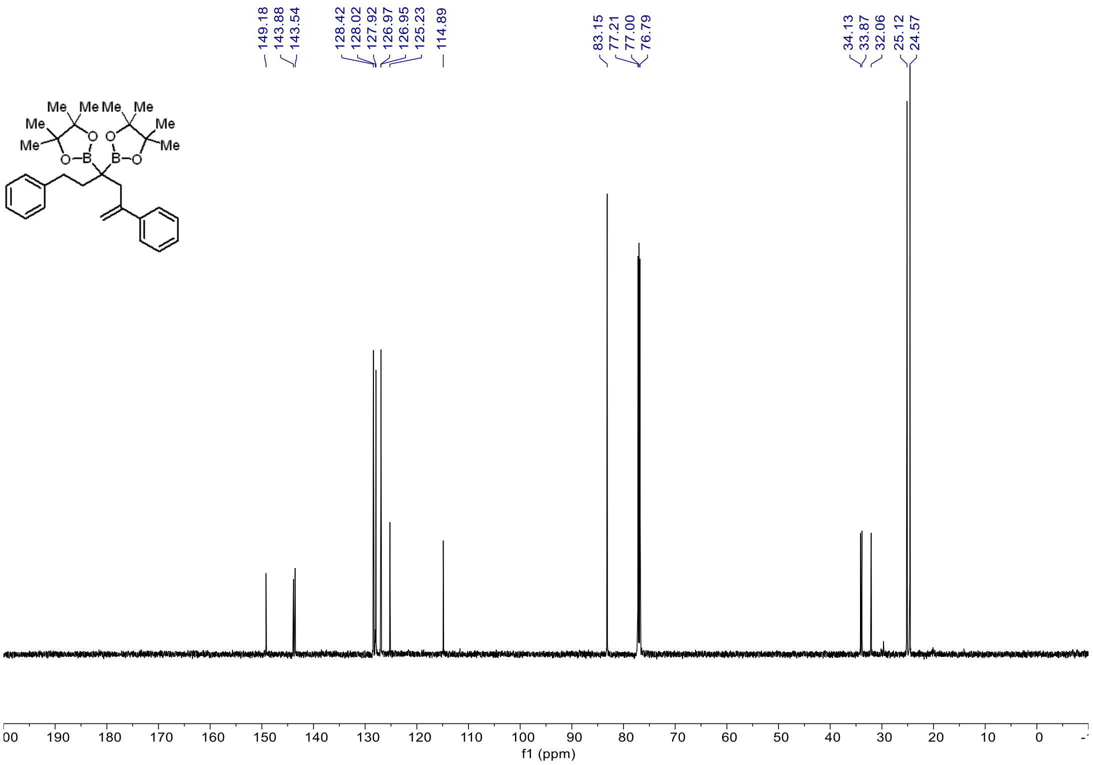

^{13}C NMR (150 MHz, $CDCl_3$) spectrum of compound **10**

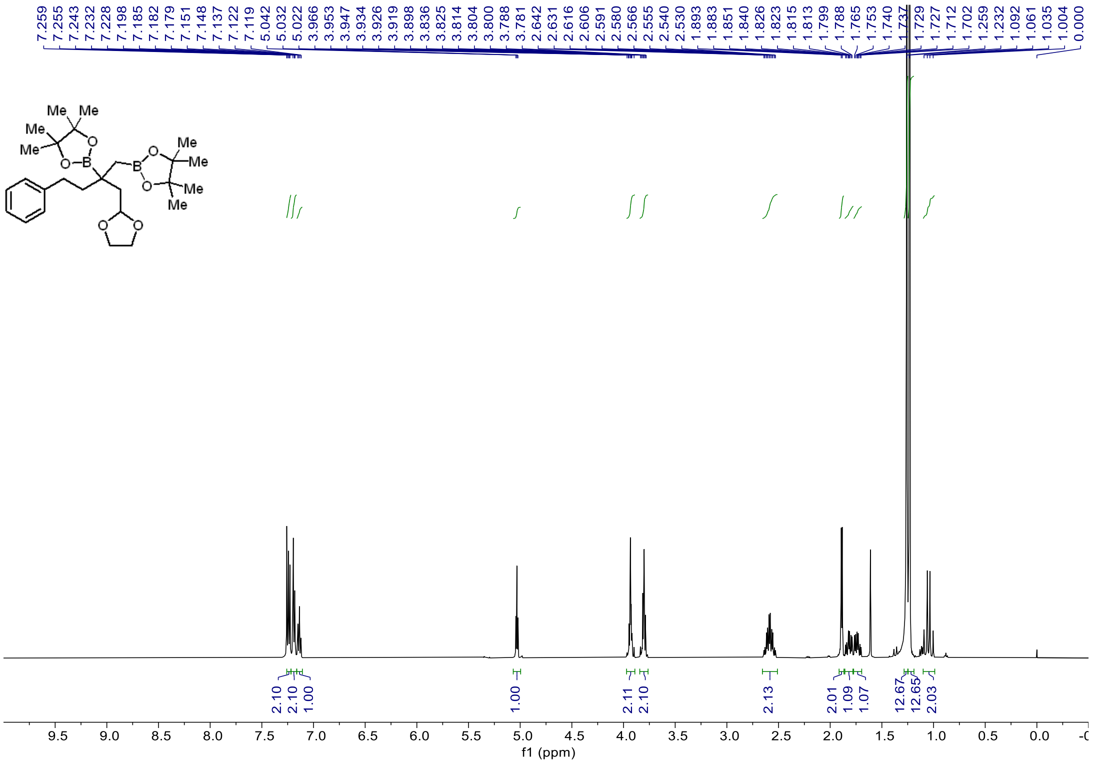

^{1}H NMR (500 MHz, $CDCl_3$) spectrum of compound **12**

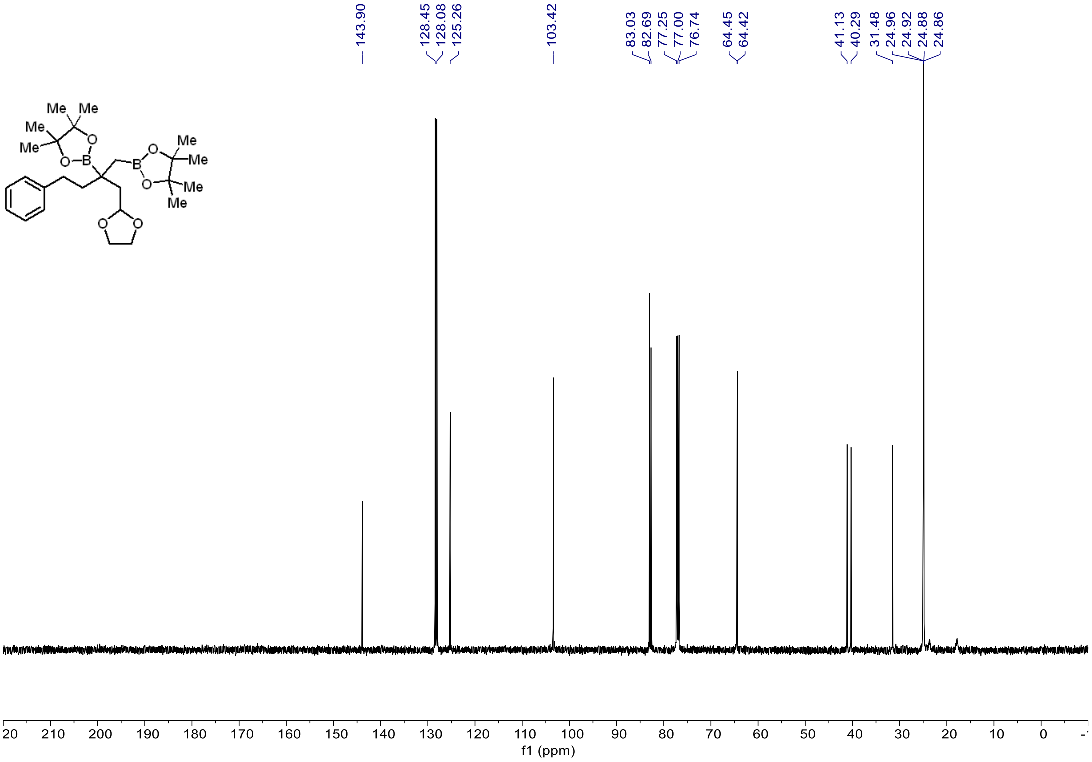

^{13}C NMR (125 MHz, $CDCl_3$) spectrum of compound **12**

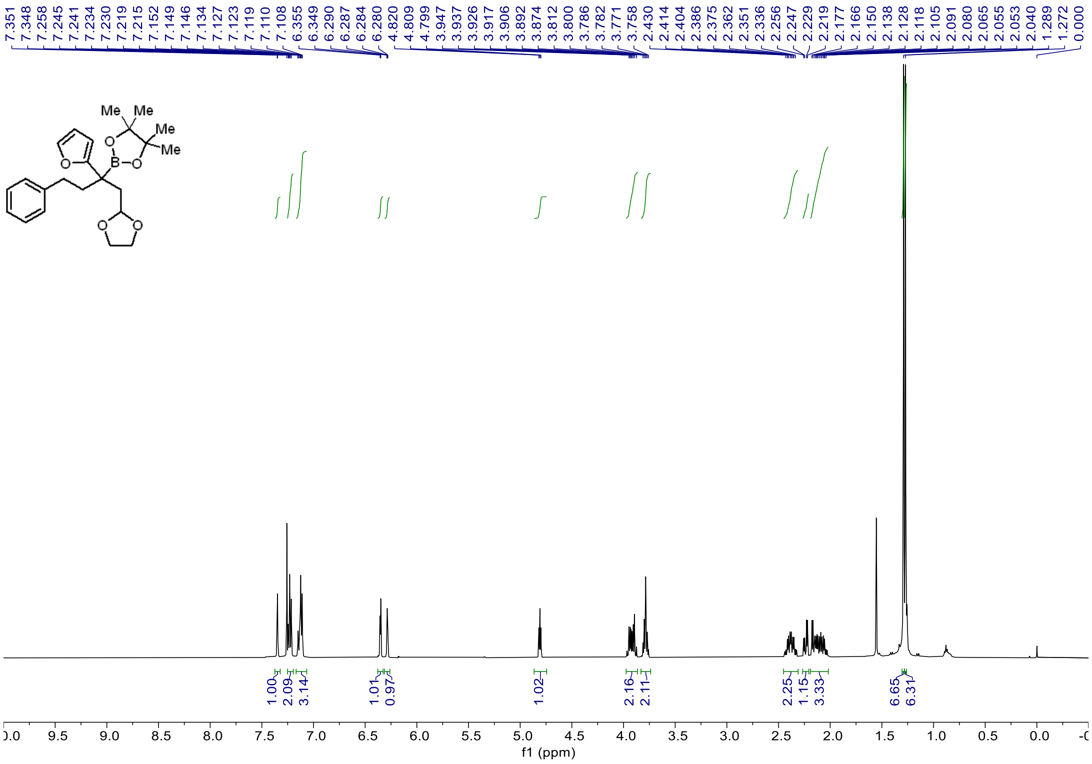

^{1}H NMR (500 MHz, $CDCl_3$) spectrum of compound **13**

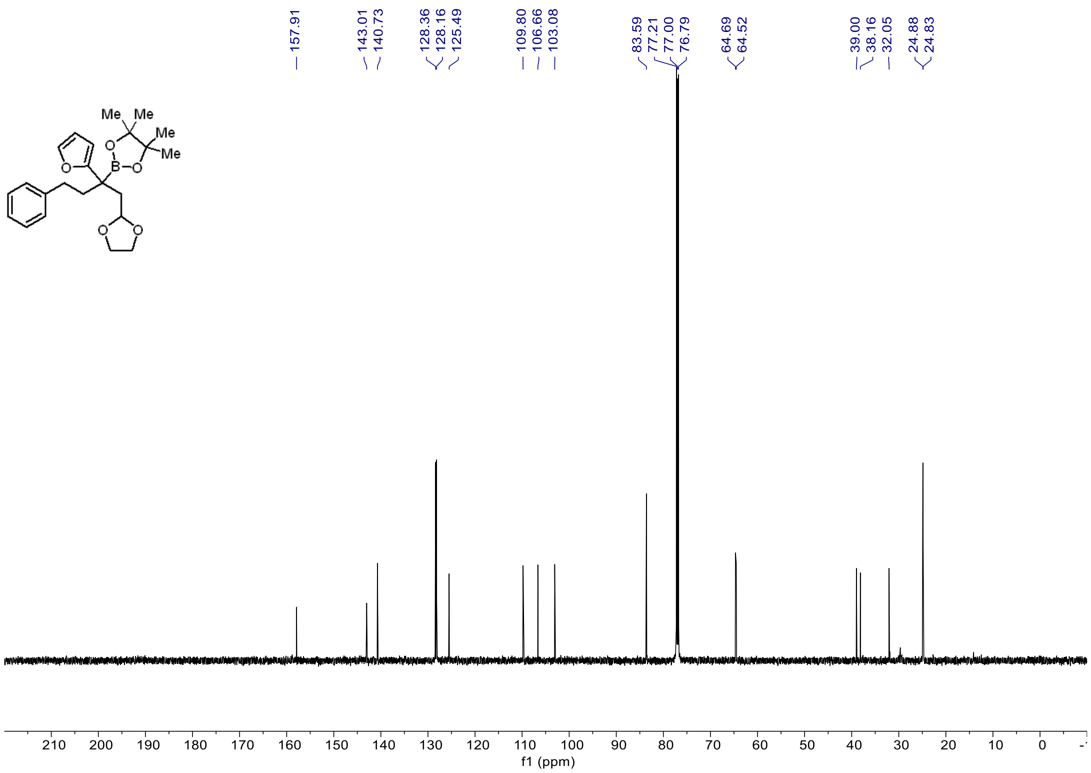

^{13}C NMR (150 MHz, $CDCl_3$) spectrum of compound **13**

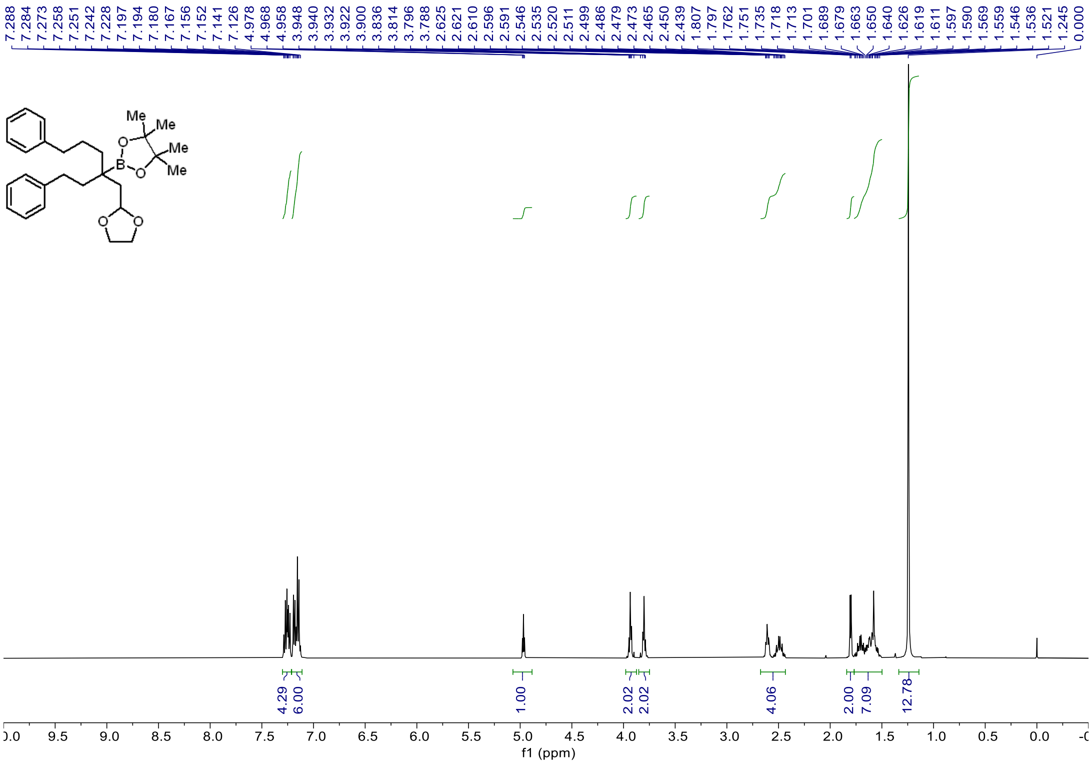

^{1}H NMR (500 MHz, $CDCl_3$) spectrum of compound **14**

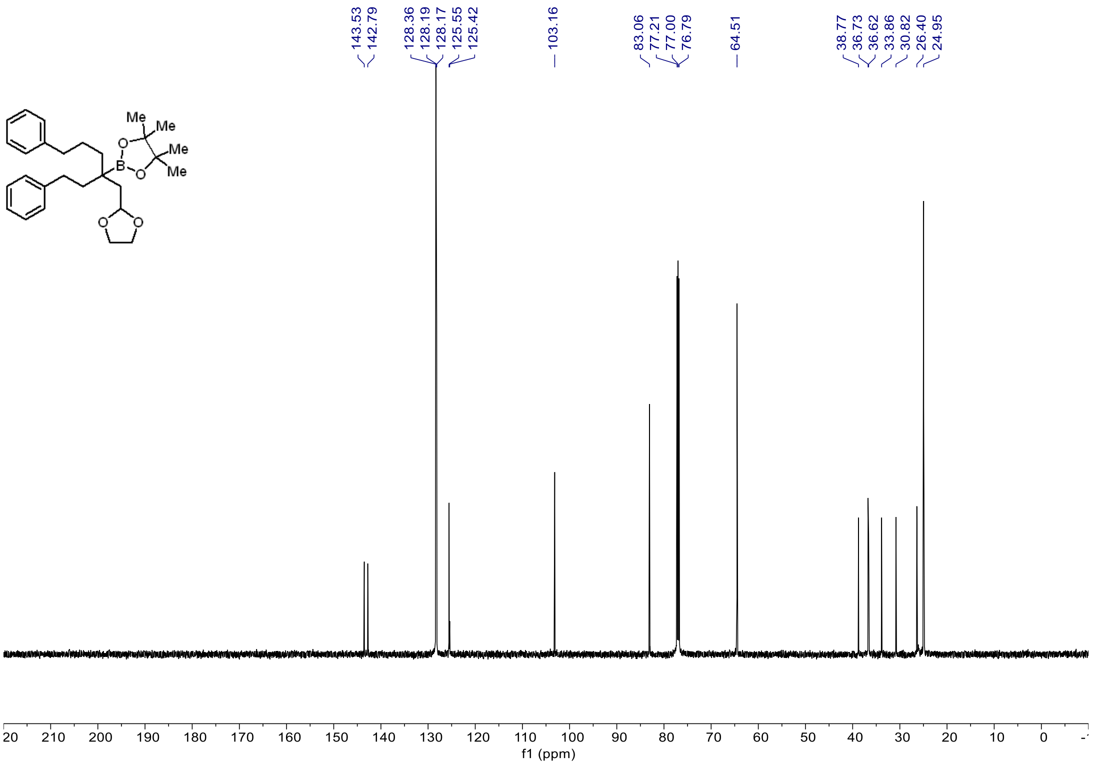

^{13}C NMR (150 MHz, CDCl$_3$) spectrum of compound **14**

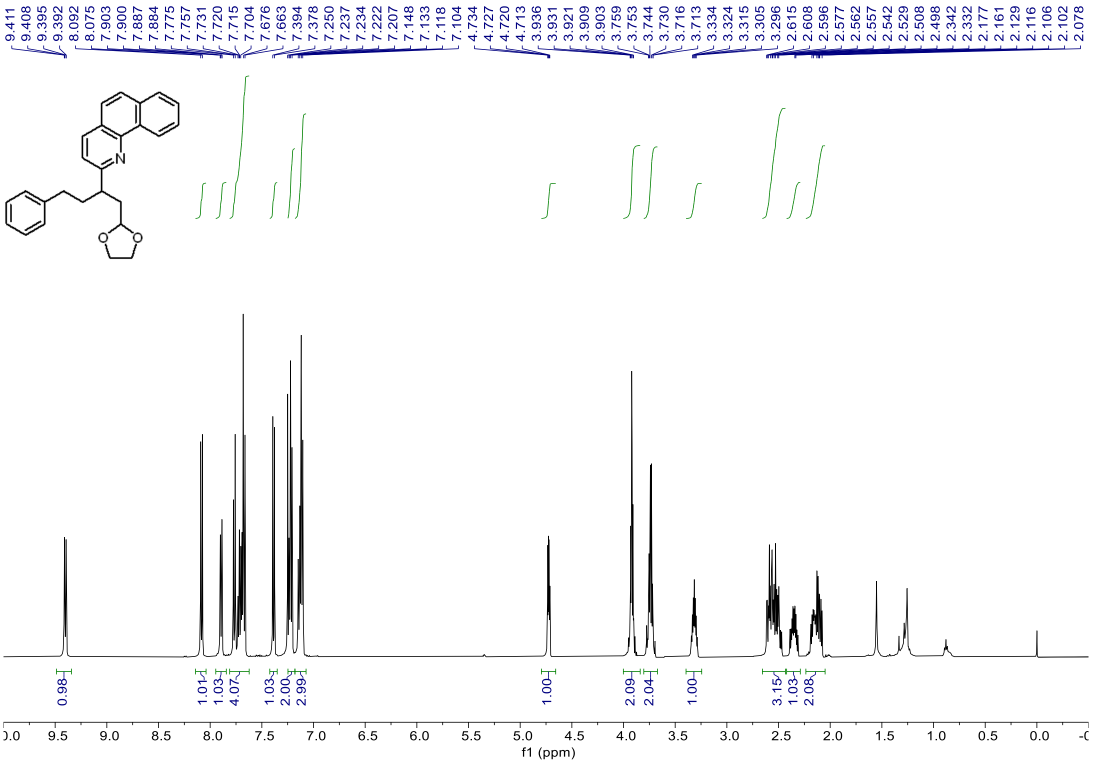

^{1}H NMR (500 MHz, CDCl$_3$) spectrum of compound **15**

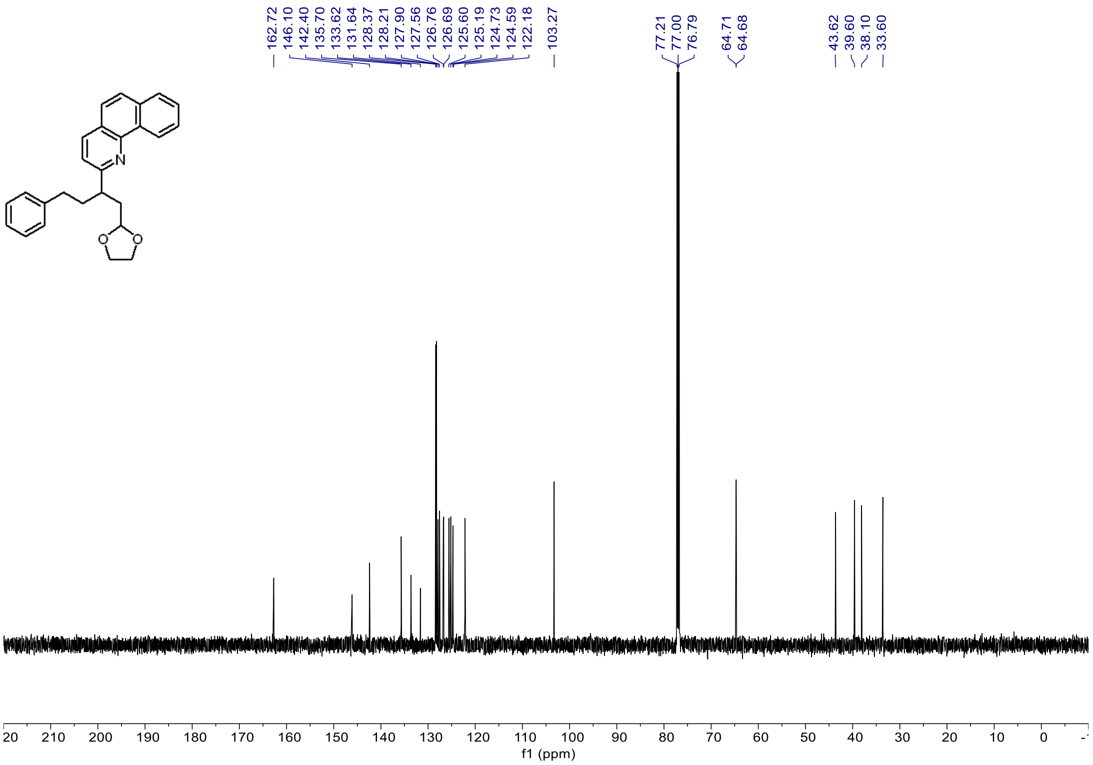

^{13}C NMR (150 MHz, $CDCl_3$) spectrum of compound **15**

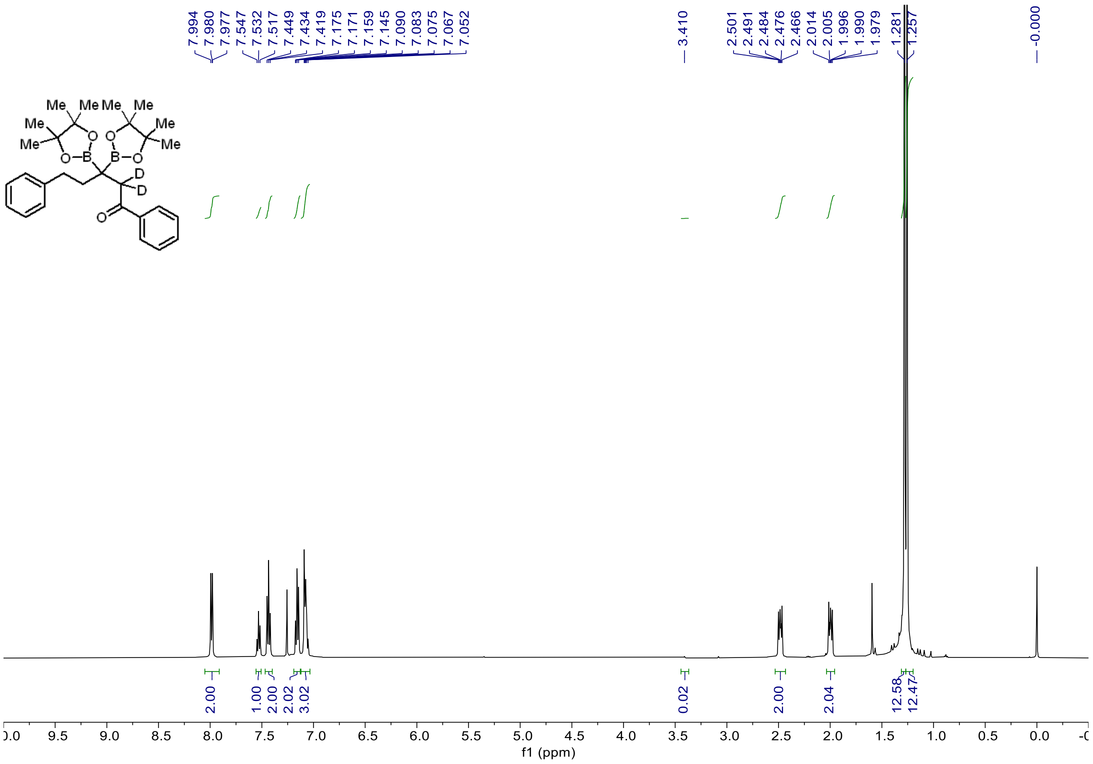

^{1}H NMR (500 MHz, $CDCl_3$) spectrum of compound **D_2-4a**

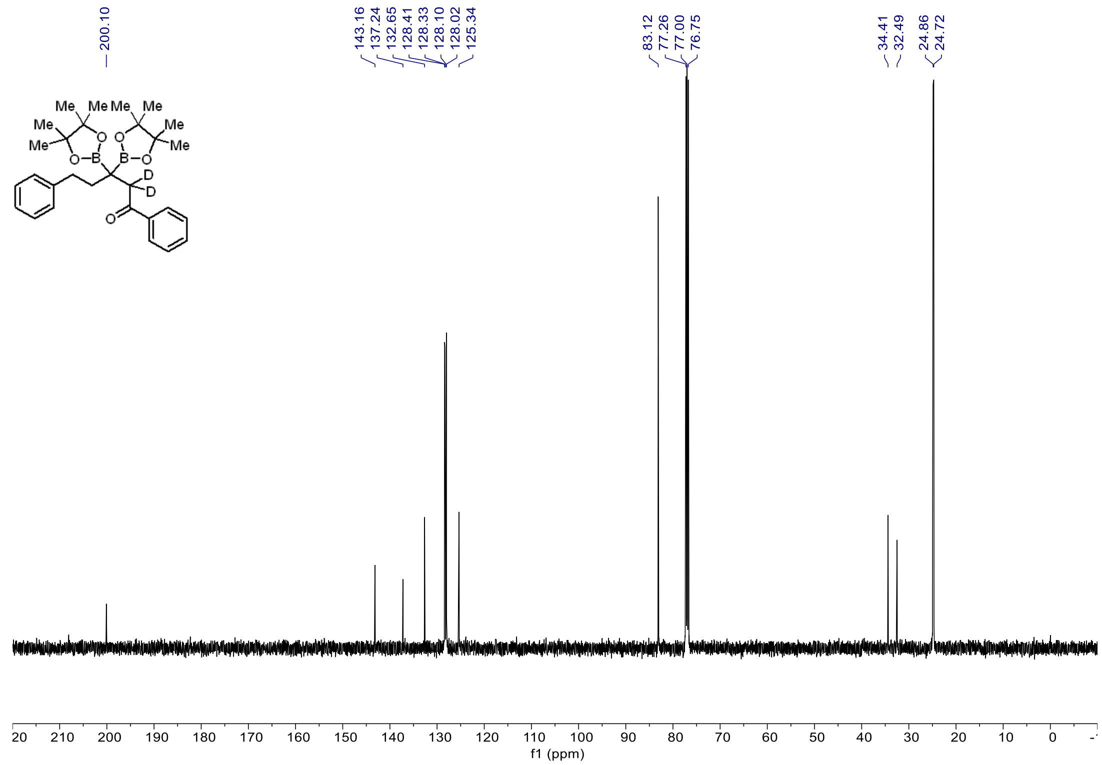

^{13}C NMR (125 MHz, $CDCl_3$) spectrum of compound **D$_2$-4a**
